# Supplementary material for: Synthesis and Characterization of Novel Heterocyclic Chalcones from 1-Phenyl-1H-pyrazol-3-ol
Source: Molecules. 2022 Jun 10;27(12):3752. doi: 10.3390/molecules27123752 (PMC9227189; doi:10.3390/molecules27123752)
Supplement: Supplementary file 1 [file molecules-27-03752-s001.zip › molecules-1742615-new-supplementary.pdf]

Supplementary Material

Synthesis and Characterization of Novel Heterocyclic Chalcones from 1-Phenyl-1*H*-pyrazol-3-ol

Arminas Urbonavičius[1,3], Graziana Fortunato[1,2], Emilija Ambrazaitytė[1], Elena Plytninkienė[1,3], Aurimas Bieliauskas[3], Vaida Milišiūnaitė[1,3], Renzo Luisi[2], Eglė Arbačiauskienė[1*], Sonata Krikštolaitytė[1], Algirdas Šačkus[1,3*]

[1]Department of Organic Chemistry, Kaunas University of Technology, Radvilėnų pl. 19, LT-50254 Kaunas, Lithuania

[2]Department of Pharmacy - Drug Sciences, University of Bari "Aldo Moro", Via E. Orabona 4, I- 70125 Bari, Italy

[3]Institute of Synthetic Chemistry, Kaunas University of Technology, K. Baršausko g. 59, LT-51423 Kaunas, Lithuania

* Corresponding authors

E-mail address: algirdas.sackus@ktu.lt (A. Šačkus), egle.arbaciauskiene@ktu.lt (E. Arbačiauskienė)

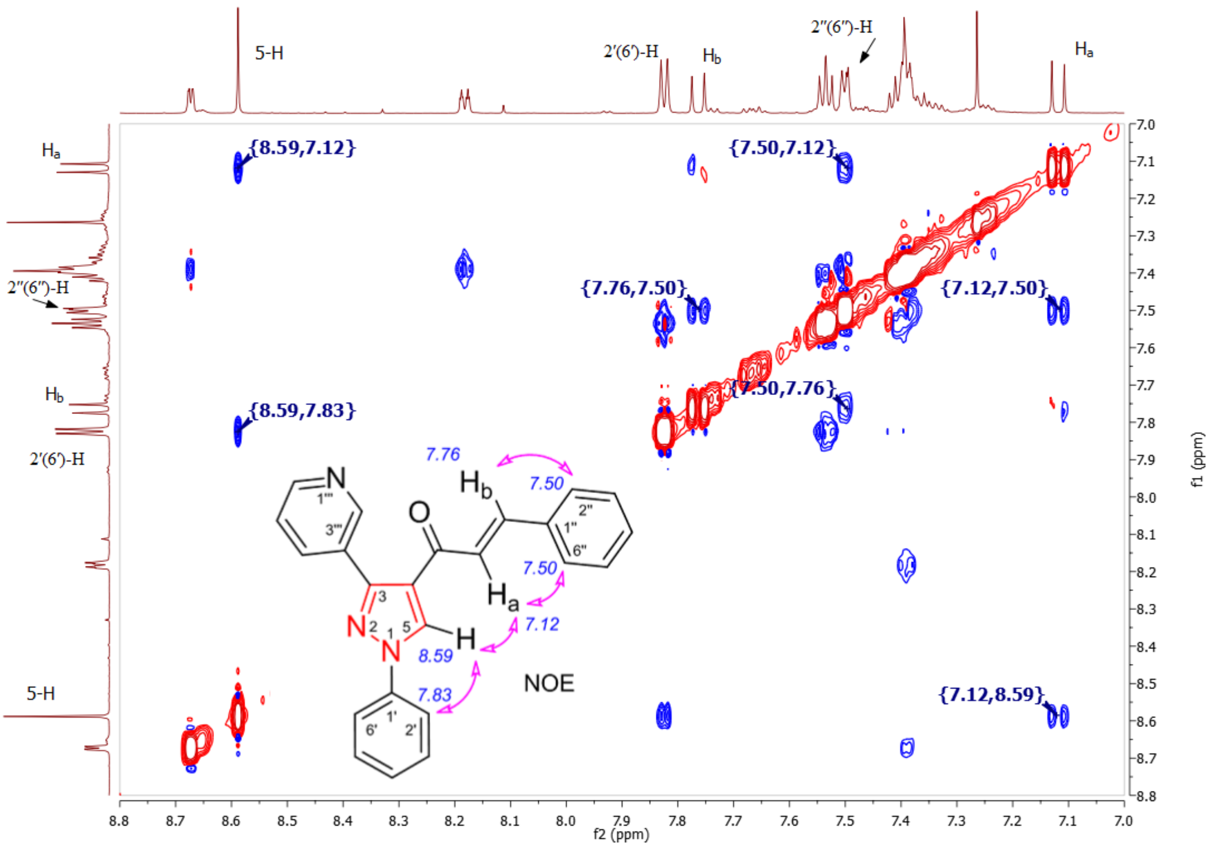

Figure S1. ^{1}H-^{1}H **NOESY NMR spectrum (*E*-12a, *Z*-13a) (700 MHz, CDCl$_3$)**

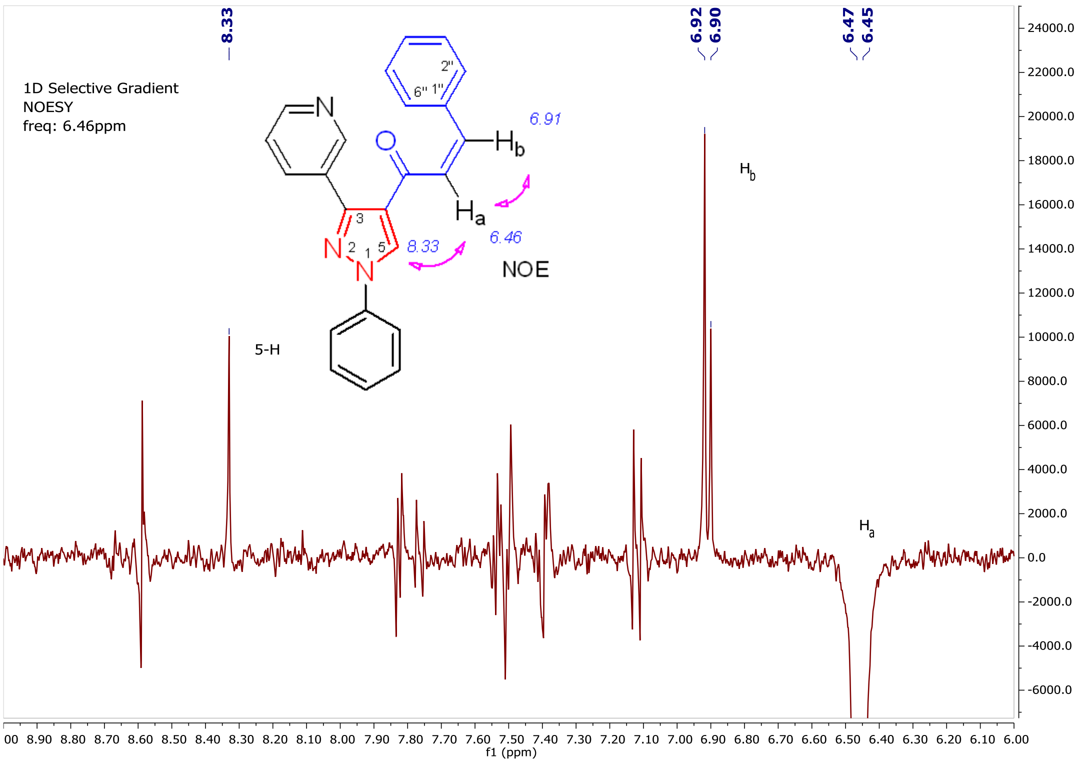

Figure S2. 1D Selective Gradient NOESY spectrum (*E*-12a, *Z*-13a) (700 MHz, CDCl$_3$)

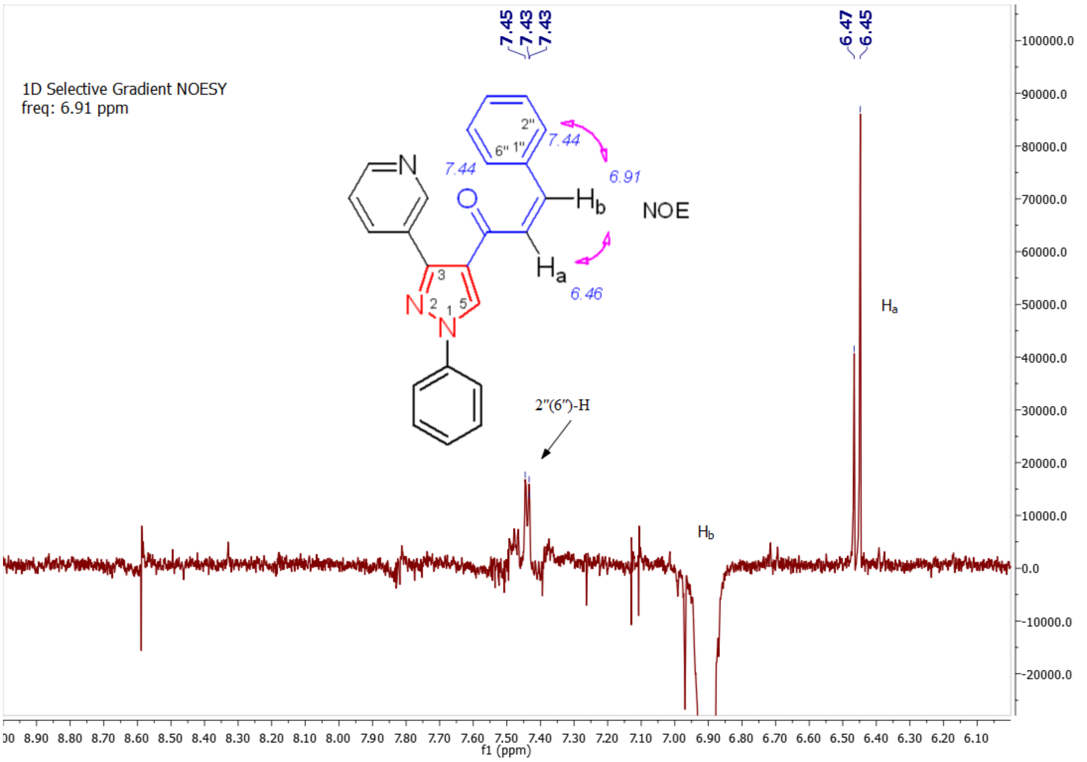

Figure S3. 1D Selective Gradient NOESY spectrum (*E*-12a, *Z*-13a) (700 MHz, $CDCl_3$)

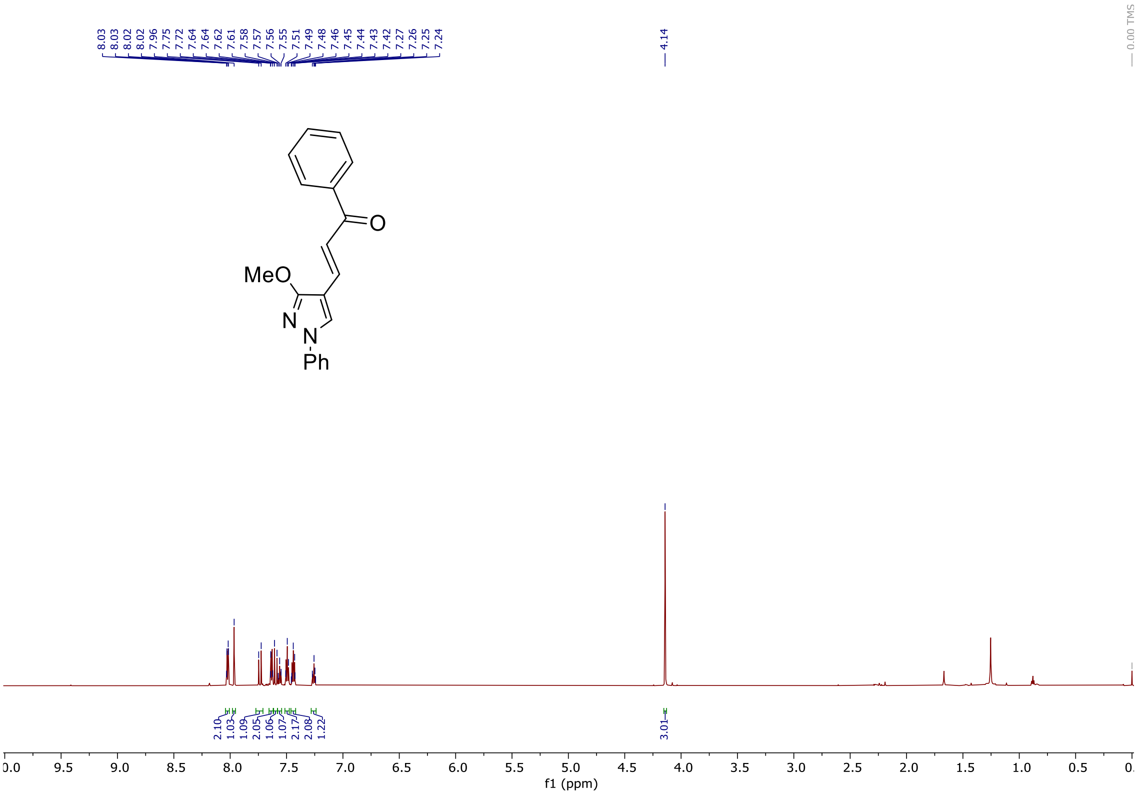

Figure S4. (2*E*)-3-[3-(Methoxy)-1-phenyl-1*H*-pyrazol-4-yl]-1-phenylprop-2-en-1-one (4a). ^{1}H NMR spectrum (700 MHz, $CDCl_3$)

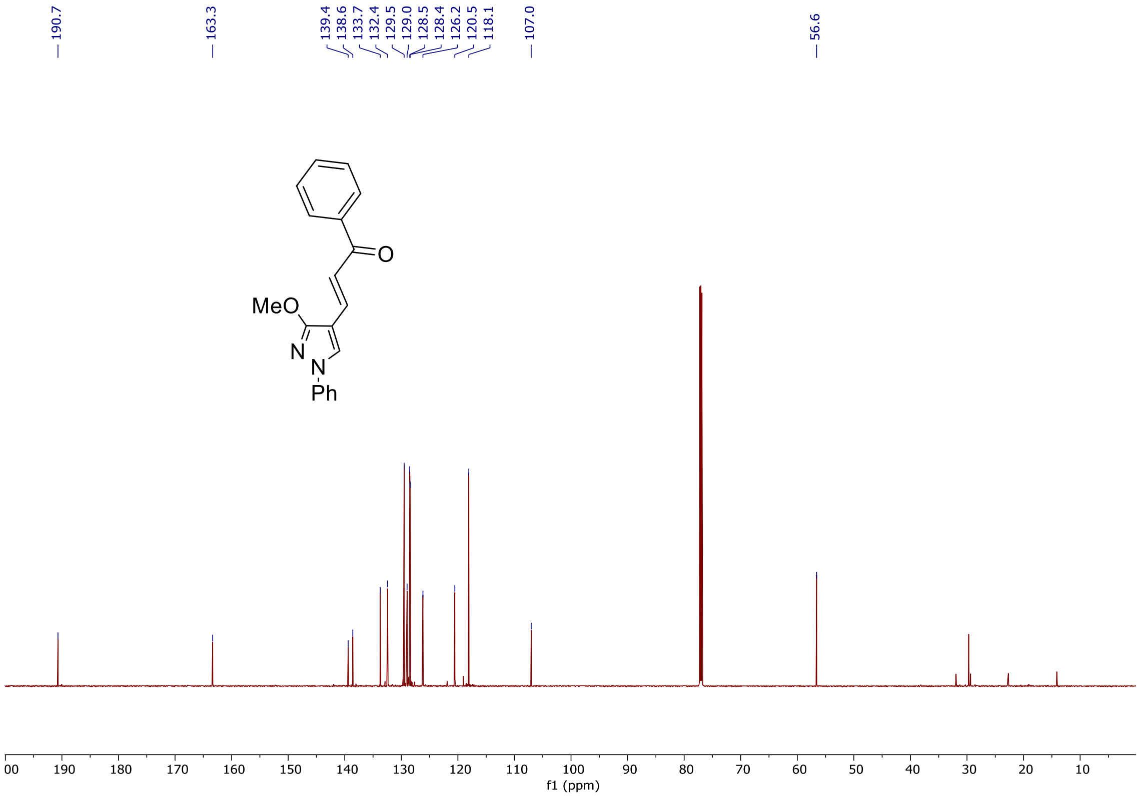

Figure S5. (2*E*)-3-[3-(Methoxy)-1-phenyl-1*H*-pyrazol-4-yl]-1-phenylprop-2-en-1-one (4a). ^{13}**C NMR spectrum (176 MHz, CDCl$_3$)**

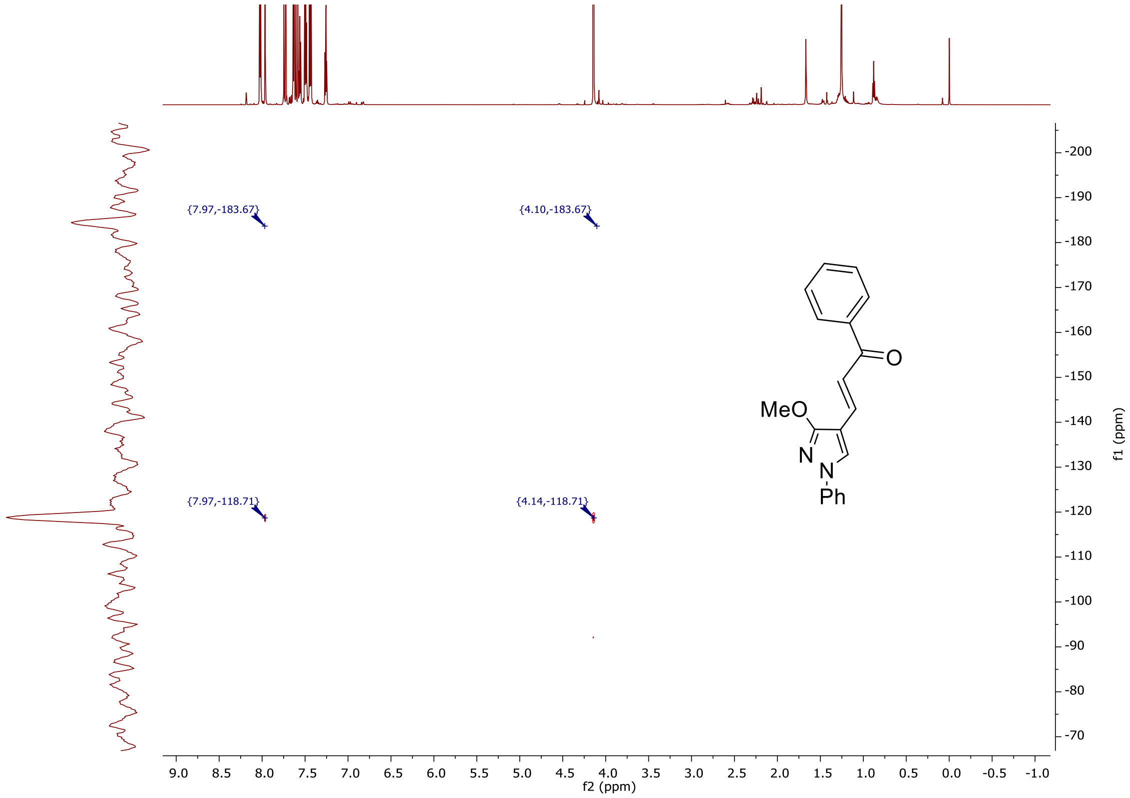

Figure S6. (2*E*)-3-[3-(Methoxy)-1-phenyl-1*H*-pyrazol-4-yl]-1-phenylprop-2-en-1-one (4a). ^{1}**H-**^{15}**N HMBC NMR spectrum (71 MHz, CDCl$_3$)**

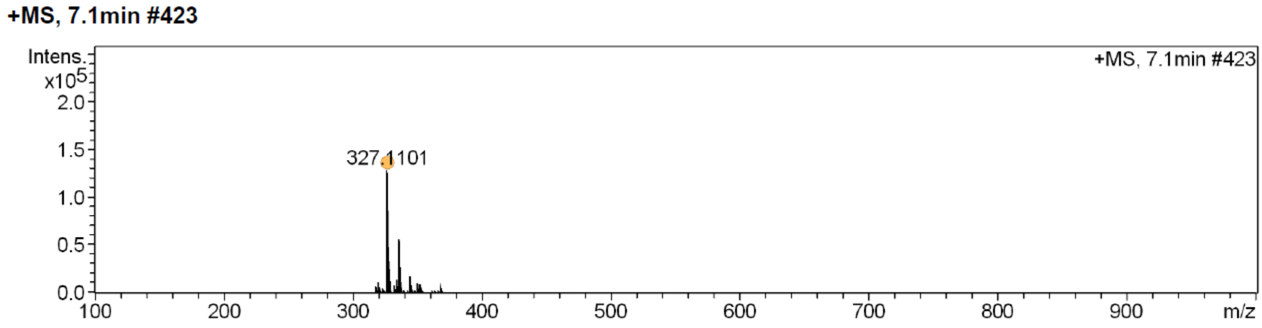

Meas. m/z	#	Ion Formula	m/z	err [ppm]	mSigma	# Sigma	Score	rdb	e⁻ Conf	N-Rule
327.1101	1	C19H16N2NaO2	327.1104	1.0	7.3	2	100.00	12.5	even	ok

Figure S7. (2*E*)-3-[3-(Methoxy)-1-phenyl-1*H*-pyrazol-4-yl]-1-phenylprop-2-en-1-one (4a). HRMS (ESI-TOF).

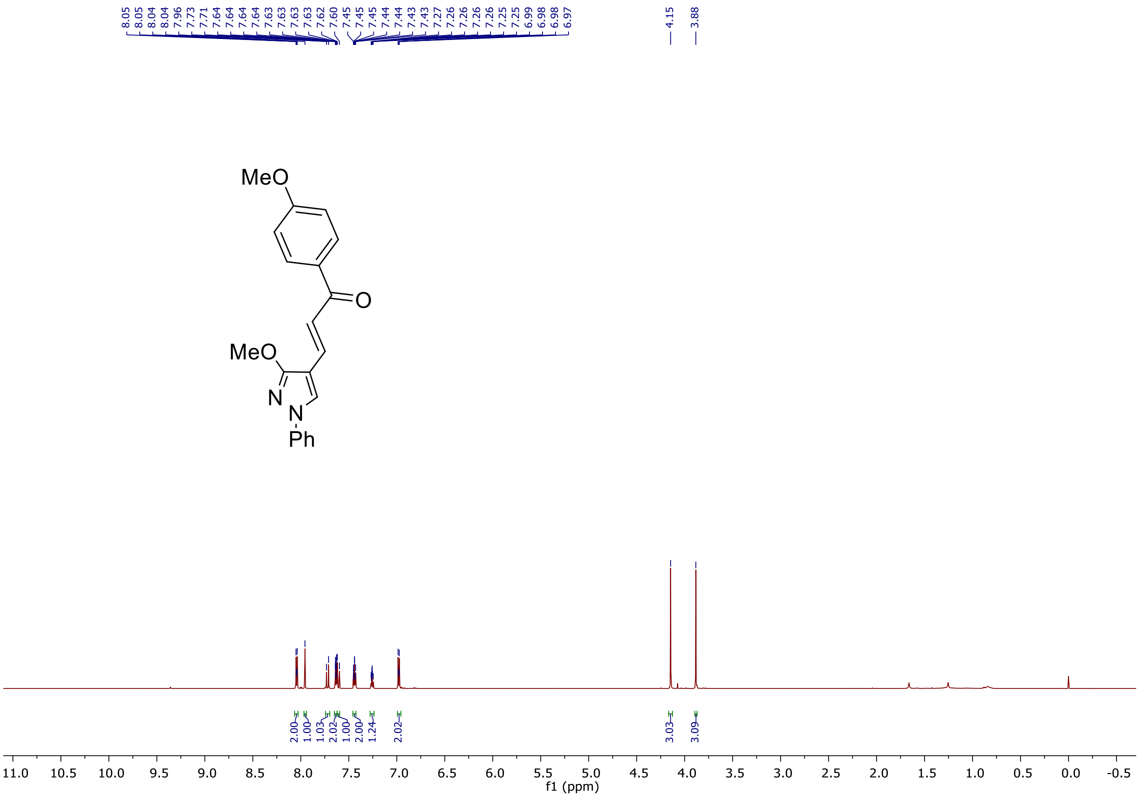

Figure S8. (2*E*)-1-(4-Methoxyphenyl)-3-(3-methoxy-1-phenyl-1*H*-pyrazol-4-yl)prop-2-en-1-one (4b). ^{1}H NMR spectrum (700 MHz, $CDCl_3$)

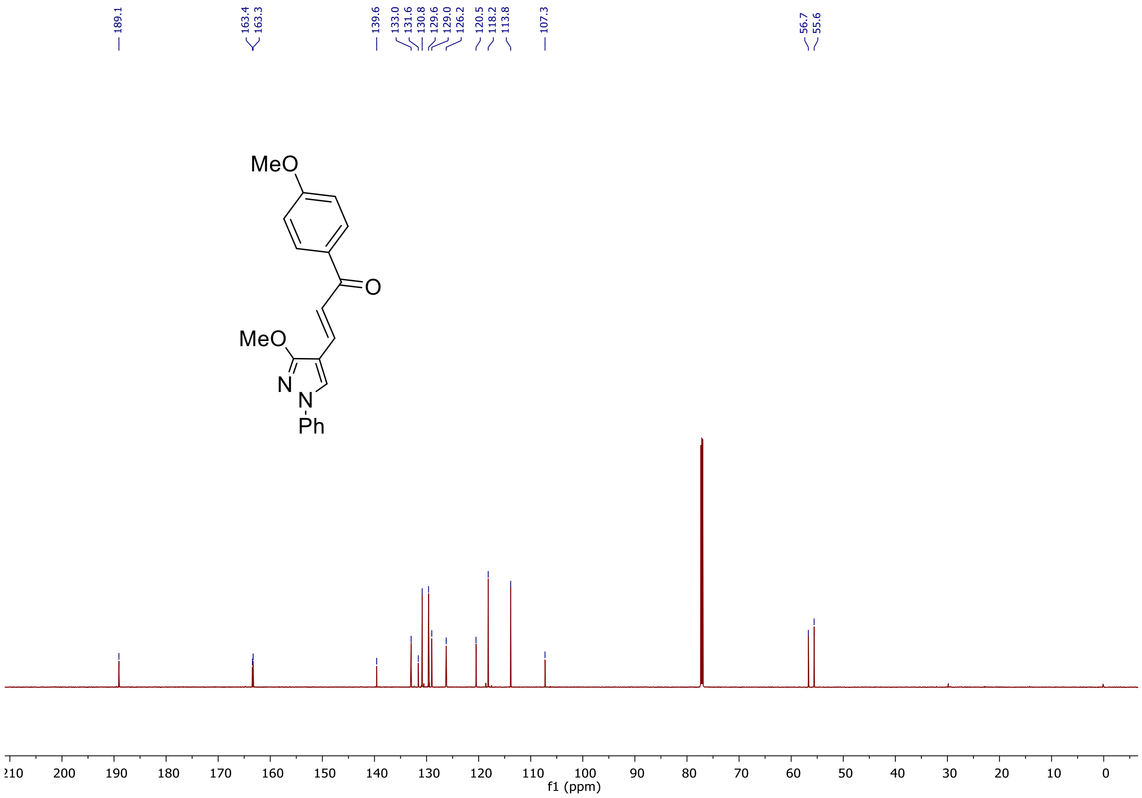

Figure S9. (2*E*)-1-(4-Methoxyphenyl)-3-(3-methoxy-1-phenyl-1*H*-pyrazol-4-yl)prop-2-en-1-one (4b). ^{13}C NMR spectrum (176 MHz, CDCl$_3$)

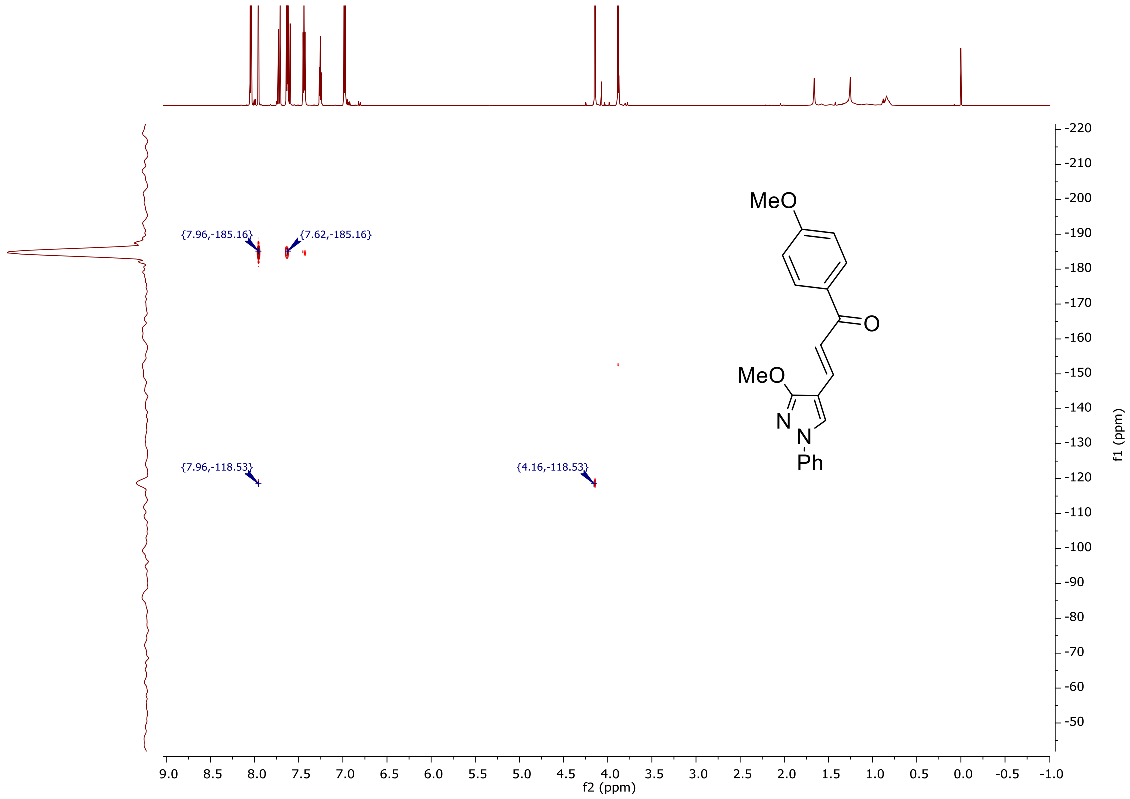

Figure S10. (2*E*)-1-(4-Methoxyphenyl)-3-(3-methoxy-1-phenyl-1*H*-pyrazol-4-yl)prop-2-en-1-one (4b). ^{1}H-^{15}N HMBC NMR spectrum (71 MHz, CDCl$_3$)

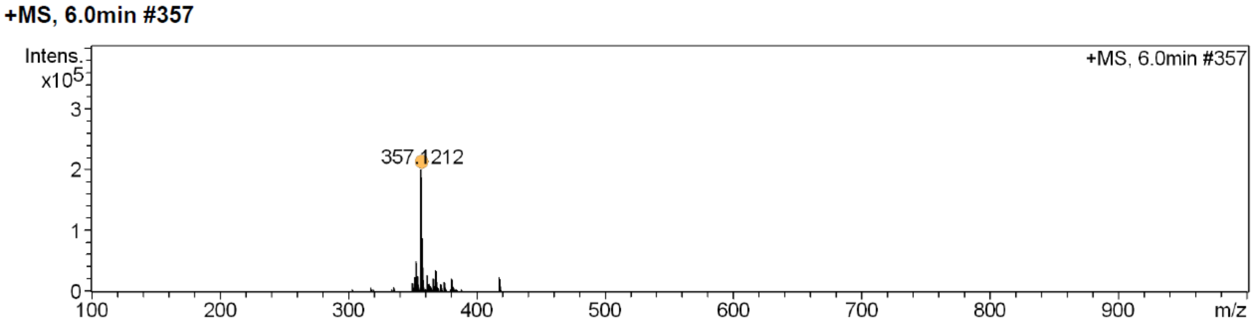

Meas. m/z	#	Ion Formula	m/z	err [ppm]	mSigma	# Sigma	Score	rdb	e¯ Conf	N-Rule
357.1212	1	C20H18N2NaO3	357.1210	-0.5	10.9	2	100.00	12.5	even	ok

Figure S11. (2*E*)-1-(4-Methoxyphenyl)-3-(3-methoxy-1-phenyl-1*H*-pyrazol-4-yl)prop-2-en-1-one (4b). HRMS (ESI-TOF).

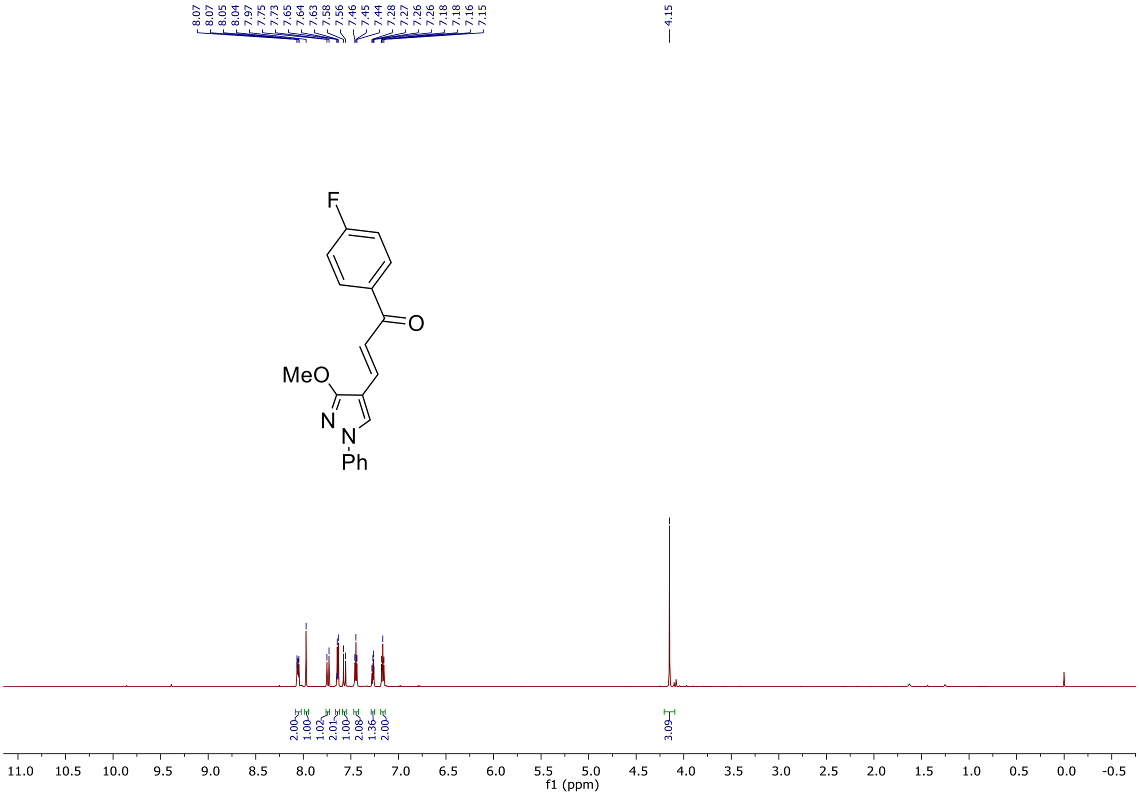

Figure S12. (2*E*)-1-(4-Fluorophenyl)-3-(3-methoxy-1-phenyl-1*H*-pyrazol-4-yl)prop-2-en-1-one (4c). ^{1}H NMR spectrum (700 MHz, $CDCl_3$)

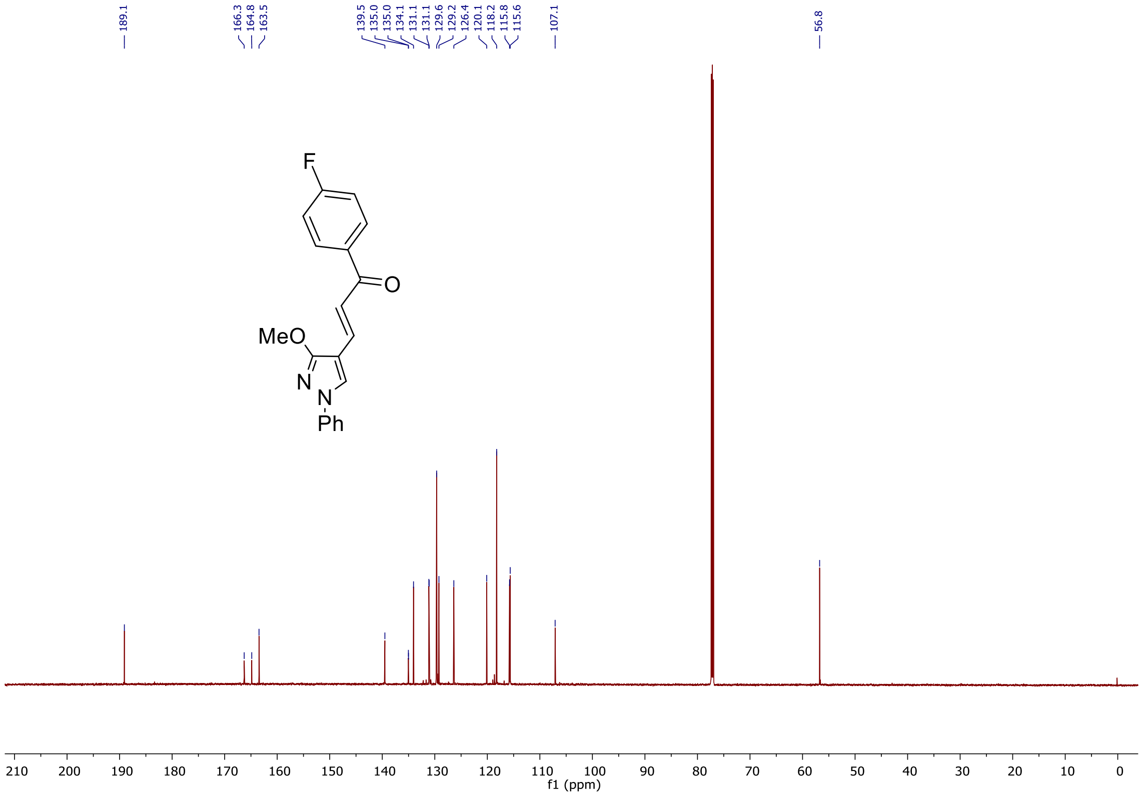

Figure S13. (2*E*)-1-(4-Fluorophenyl)-3-(3-methoxy-1-phenyl-1*H*-pyrazol-4-yl)prop-2-en-1-one (4c). ^{13}C NMR spectrum (176 MHz, $CDCl_3$)

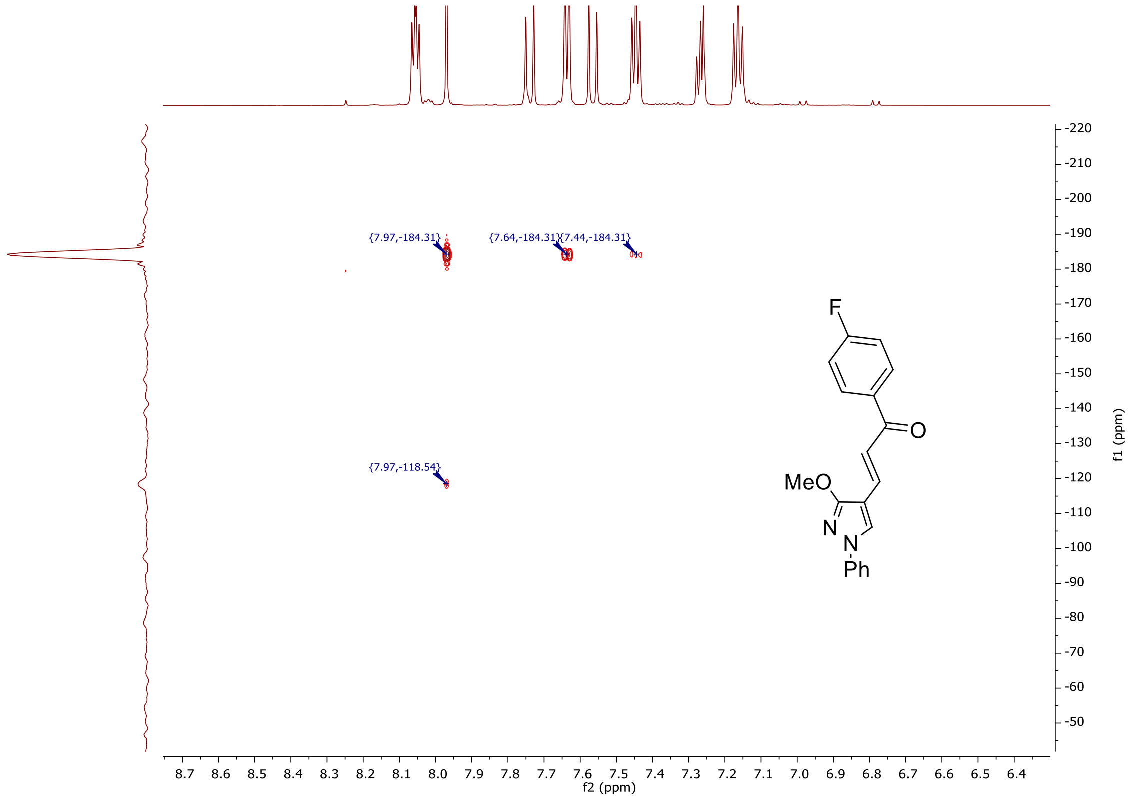

Figure S14. (2*E*)-1-(4-Fluorophenyl)-3-(3-methoxy-1-phenyl-1*H*-pyrazol-4-yl)prop-2-en-1-one (4c). ^{1}H-^{15}N HMBC NMR spectrum (71 MHz, $CDCl_3$)

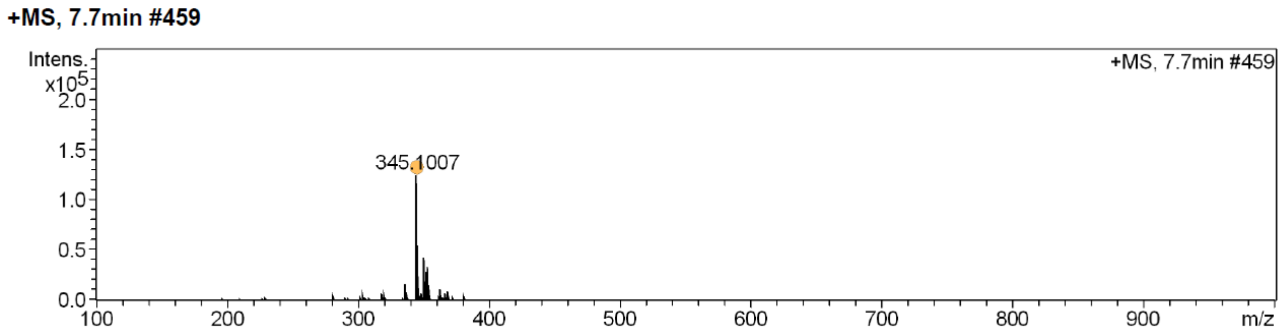

Meas. m/z	#	Ion Formula	m/z	err [ppm]	mSigma	# Sigma	Score	rdb	e⁻ Conf	N-Rule
345.1007	1	C19H15FN2NaO2	345.1010	0.9	10.2	4	100.00	12.5	even	ok

Figure S15. (2*E*)-1-(4-Fluorophenyl)-3-(3-methoxy-1-phenyl-1*H*-pyrazol-4-yl)prop-2-en-1-one (4c). HRMS (ESI-TOF).

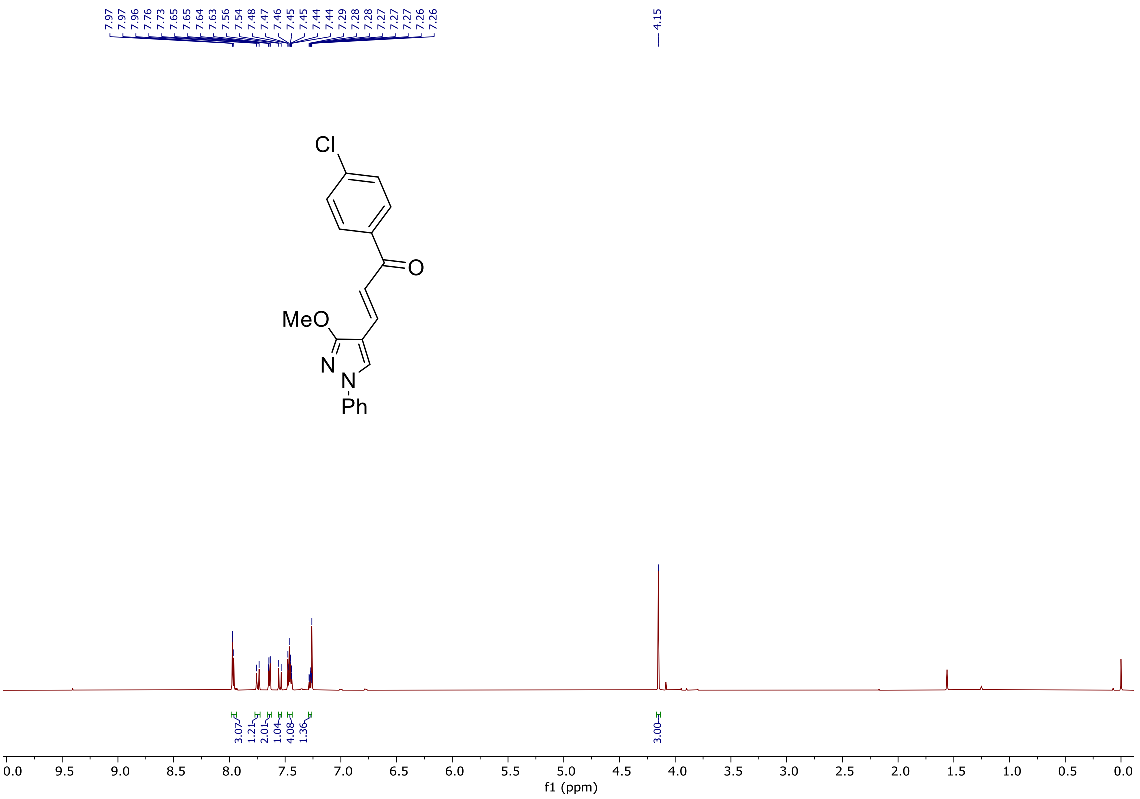

Figure S16. (2*E*)-1-(4-Chlorophenyl)-3-(3-methoxy-1-phenyl-1*H*-pyrazol-4-yl)prop-2-en-1-one (4d). ^{1}H NMR spectrum (700 MHz, $CDCl_3$)

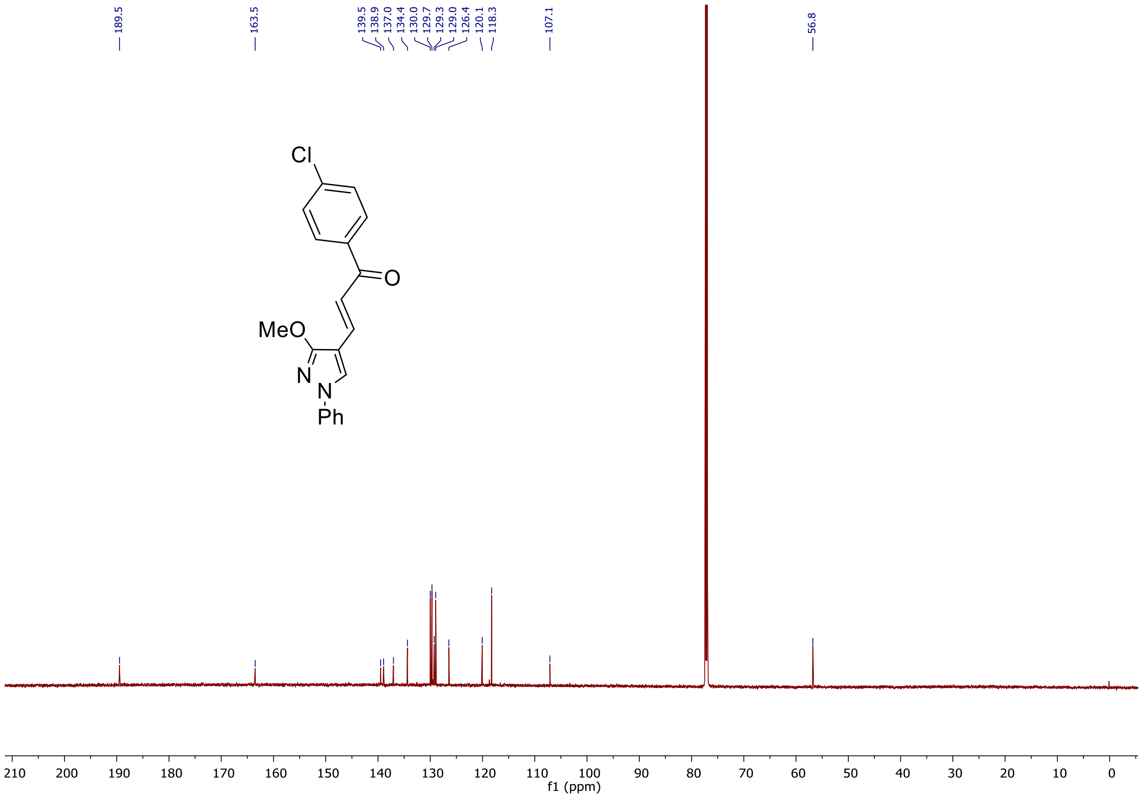

Figure S17. (2*E*)-1-(4-Chlorophenyl)-3-(3-methoxy-1-phenyl-1*H*-pyrazol-4-yl)prop-2-en-1-one (4d). ^{13}**C NMR spectrum (176 MHz, CDCl$_3$)**

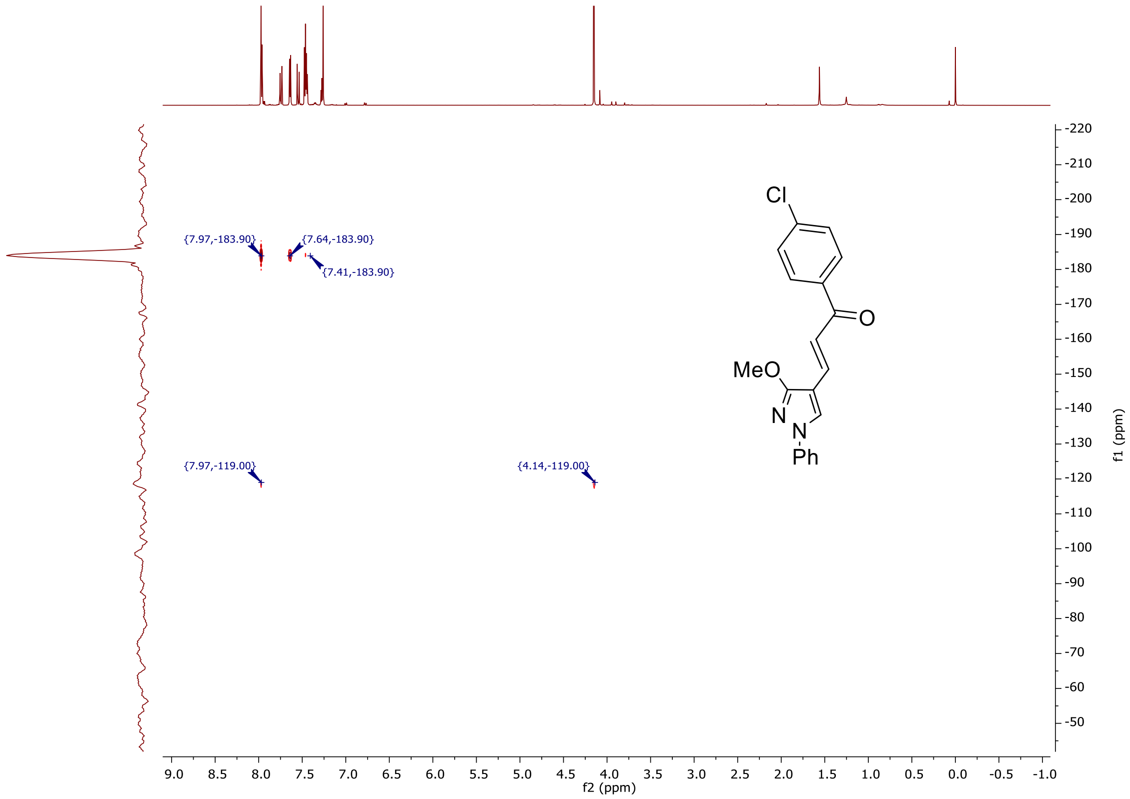

Figure S18. (2*E*)-1-(4-Chlorophenyl)-3-(3-methoxy-1-phenyl-1*H*-pyrazol-4-yl)prop-2-en-1-one (4d). ^{1}**H-**^{15}**N HMBC NMR spectrum (71 MHz, CDCl$_3$)**

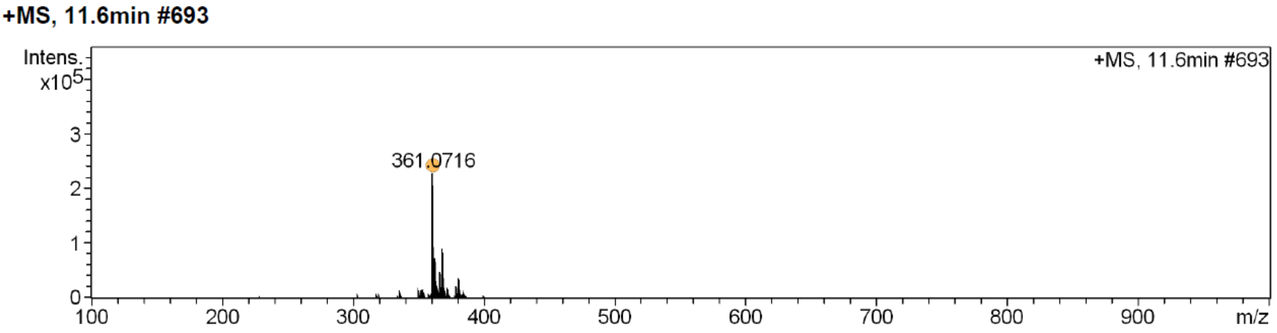

Meas. m/z	#	Ion Formula	m/z	err [ppm]	mSigma	# Sigma	Score	rdb	e⁻ Conf	N-Rule
361.0716	1	C19H15ClN2NaO2	361.0714	0.4	12.0	1	100.00	12.5	even	ok

Figure S19. (2*E*)-1-(4-Chlorophenyl)-3-(3-methoxy-1-phenyl-1*H*-pyrazol-4-yl)prop-2-en-1-one (4d). HRMS (ESI-TOF).

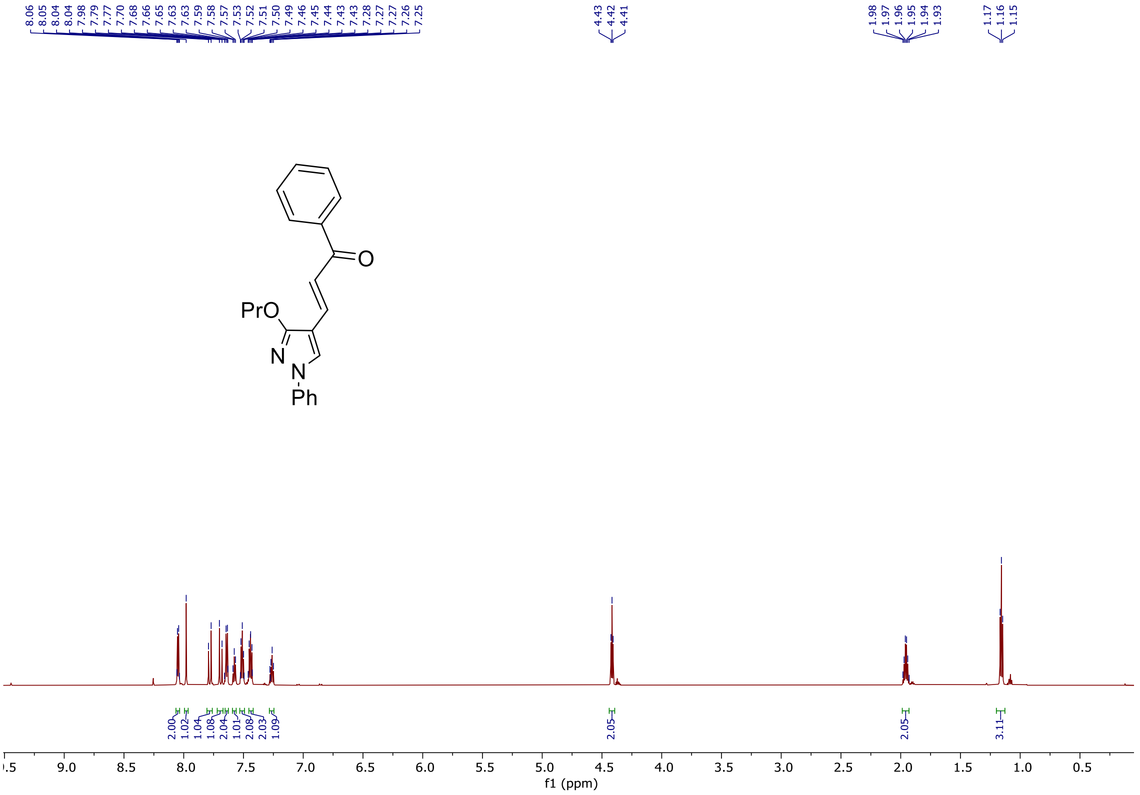

Figure S20. (2*E*)-1-Phenyl-3-(1-phenyl-3-propoxy-1*H*-pyrazol-4-yl)prop-2-en-1-one (4e). ^{1}H NMR spectrum (700 MHz, $CDCl_3$)

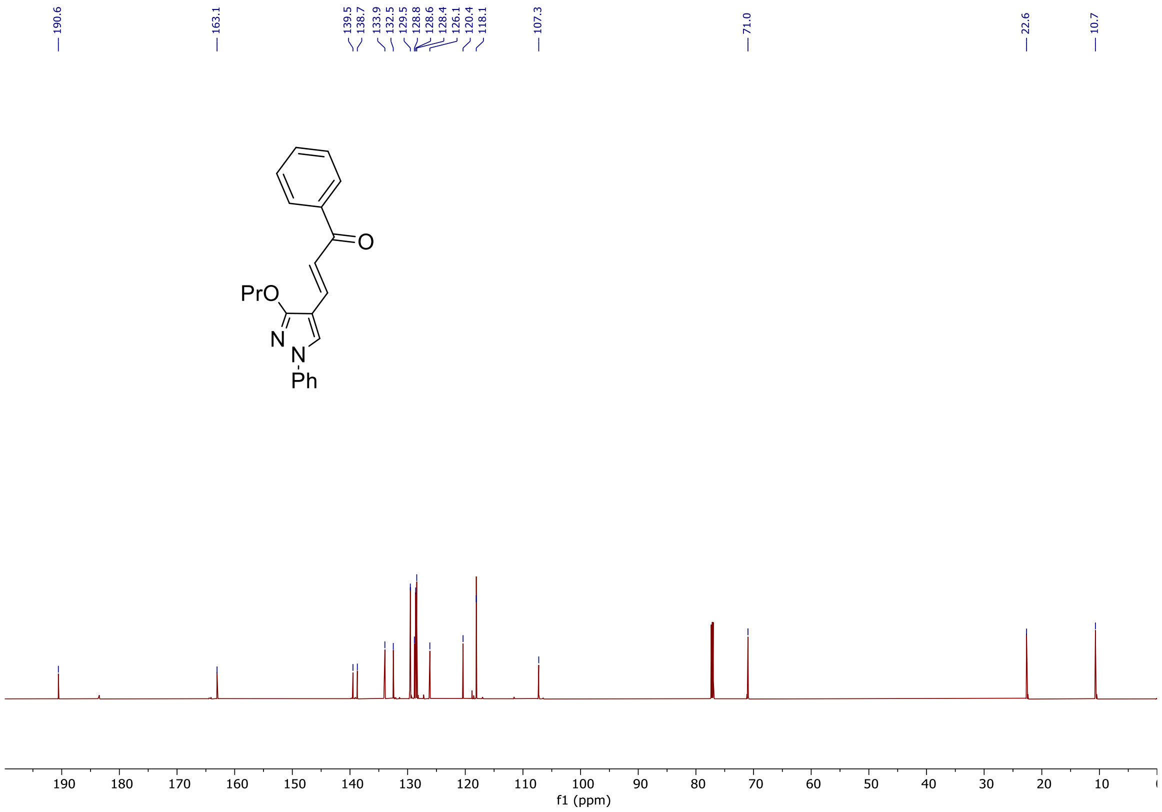

Figure S21. (2*E*)-1-Phenyl-3-(1-phenyl-3-propoxy-1*H*-pyrazol-4-yl)prop-2-en-1-one (4e). ^{13}C NMR spectrum (176 MHz, $CDCl_3$)

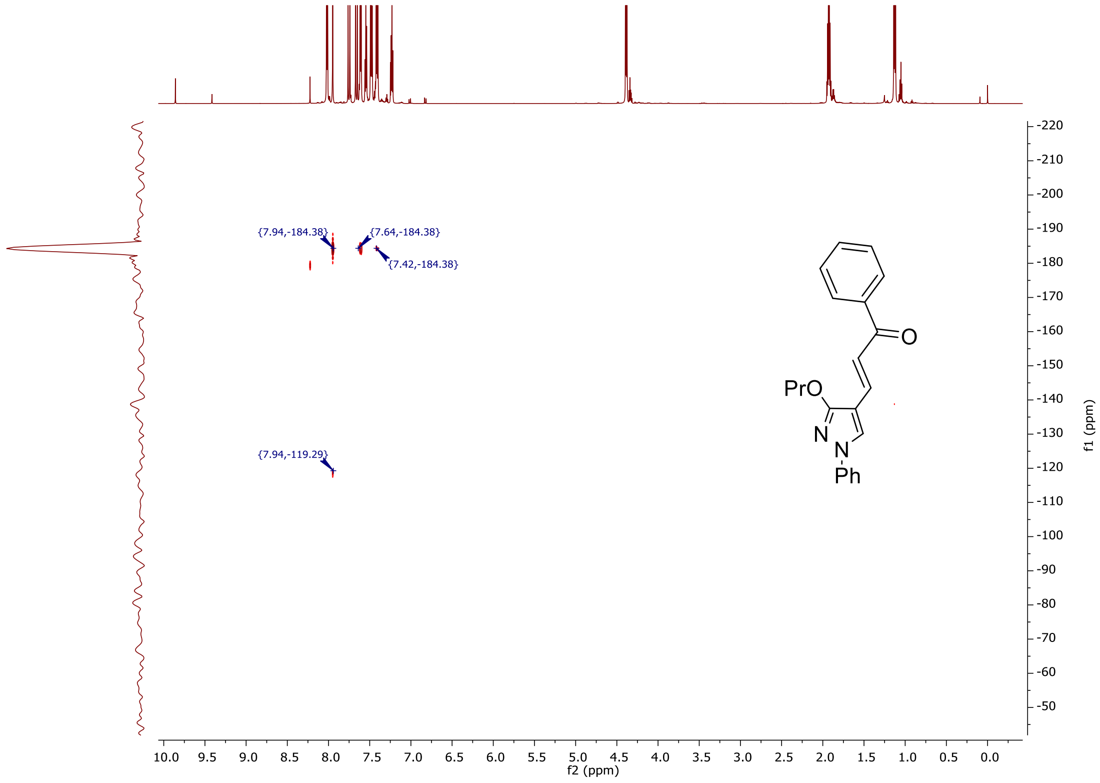

Figure S22. (2*E*)-1-Phenyl-3-(1-phenyl-3-propoxy-1*H*-pyrazol-4-yl)prop-2-en-1-one (4e). ^{1}H-^{15}N HMBC NMR spectrum (71 MHz, $CDCl_3$)

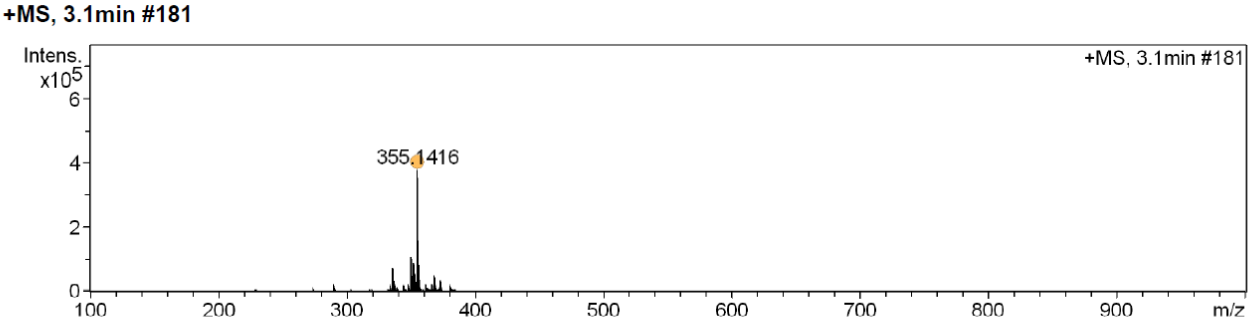

Meas. m/z	#	Ion Formula	m/z	err [ppm]	mSigma	# Sigma	Score	rdb	e⁻ Conf	N-Rule
355.1416	1	C21H20N2NaO2	355.1417	0.3	4.2	1	100.00	12.5	even	ok

Figure S23. (2*E*)-1-Phenyl-3-(1-phenyl-3-propoxy-1*H*-pyrazol-4-yl)prop-2-en-1-one (4e). HRMS (ESI-TOF).

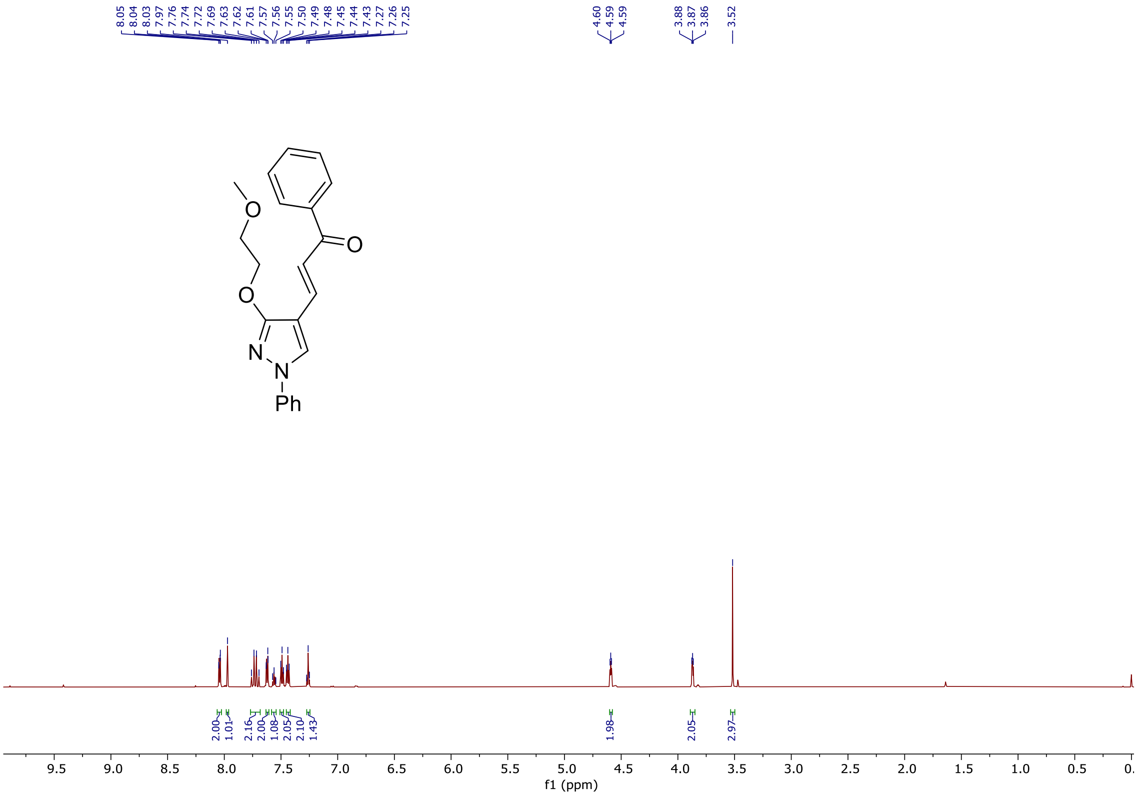

Figure S24. (2*E*)-3-[3-(2-Methoxyethoxy)-1-phenyl-1*H*-pyrazol-4-yl]-1-phenylprop-2-en-1-one (4f). ¹H NMR spectrum (700 MHz, $CDCl_3$)

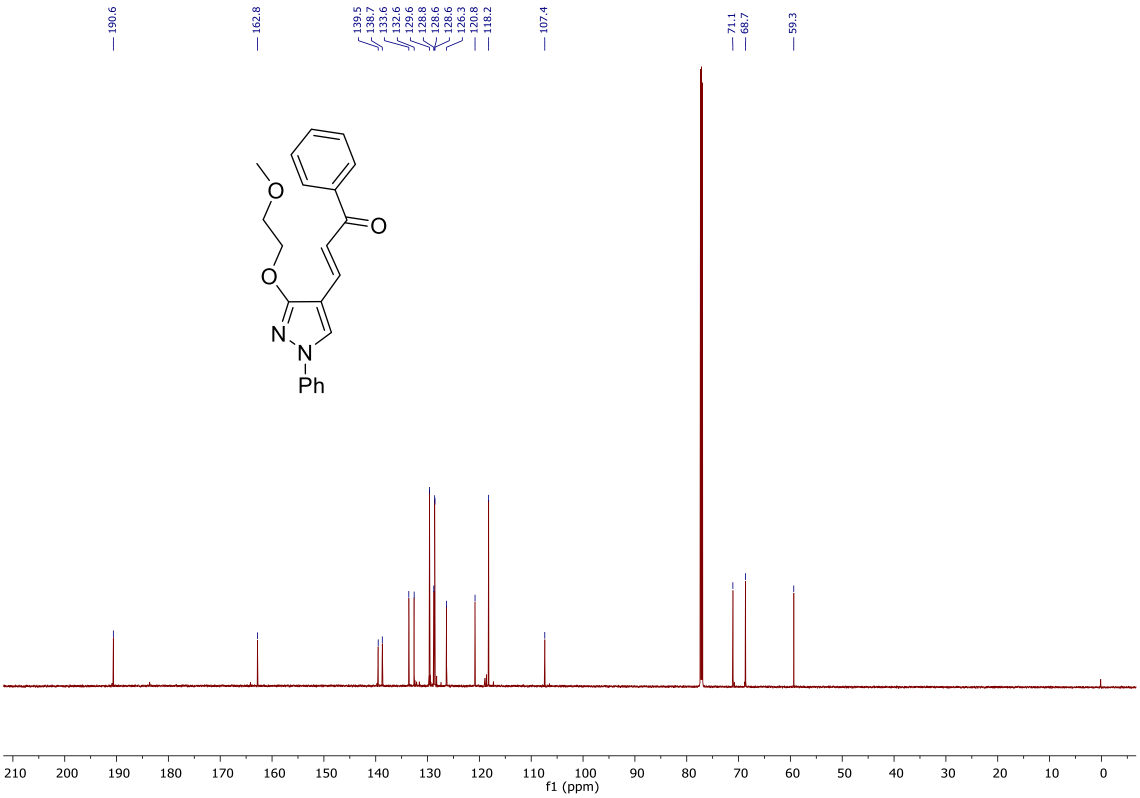

Figure S25. (2*E*)-3-[3-(2-Methoxyethoxy)-1-phenyl-1*H*-pyrazol-4-yl]-1-phenylprop-2-en-1-one (4f). ^{13}C NMR spectrum (CDCl$_3$)

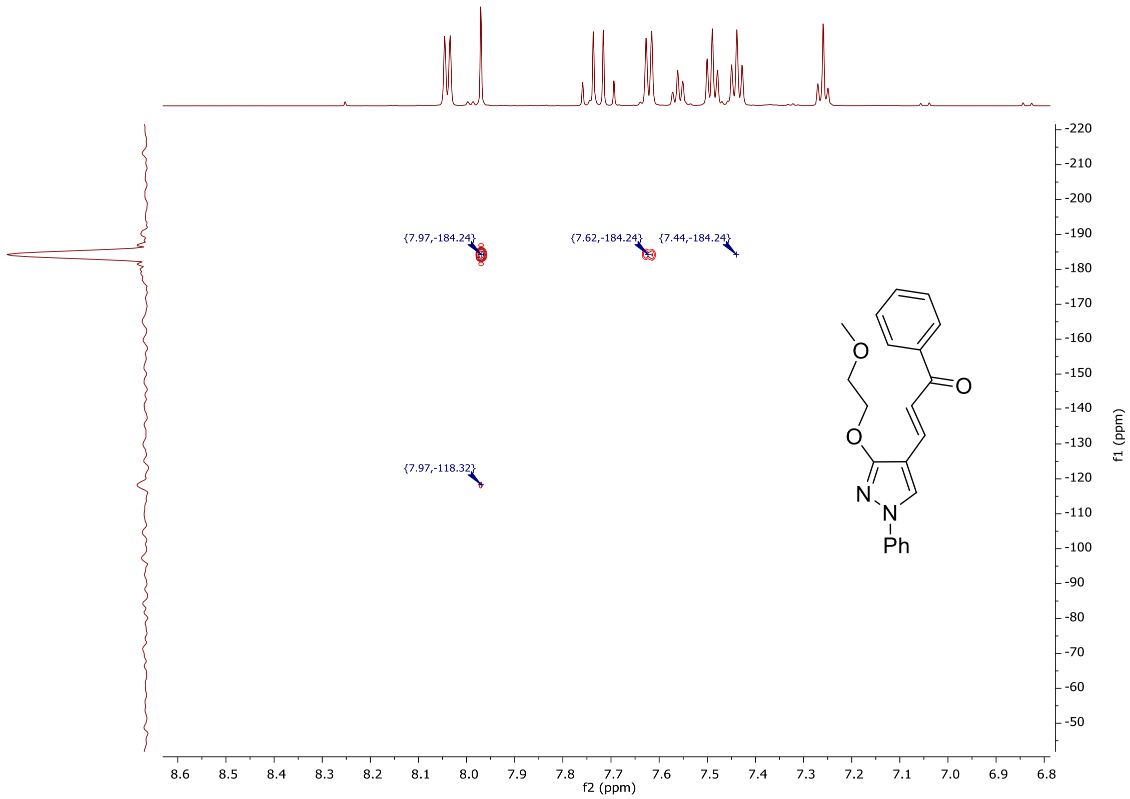

Figure S26. (2*E*)-3-[3-(2-Methoxyethoxy)-1-phenyl-1*H*-pyrazol-4-yl]-1-phenylprop-2-en-1-one (4f). ^{1}H-^{15}N HMBC NMR spectrum (71 MHz, CDCl$_3$)

+MS, 7.3min #436

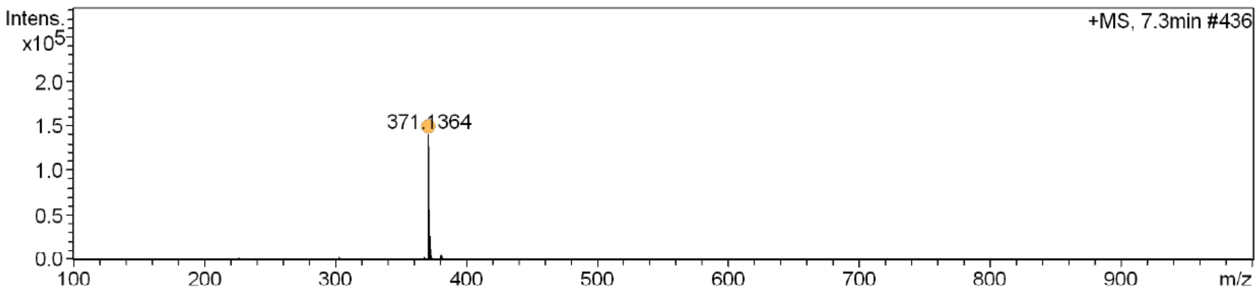

Meas. m/z	#	Ion Formula	m/z	err [ppm]	mSigma	# Sigma	Score	rdb	e¯ Conf	N-Rule
371.1364	1	C21H20N2NaO3	371.1366	-0.6	15.7	3	100.00	12.5	even	ok

Figure S27. (2*E*)-3-[3-(2-Methoxyethoxy)-1-phenyl-1*H*-pyrazol-4-yl]-1-phenylprop-2-en-1-one (4f). HRMS (ESI-TOF).

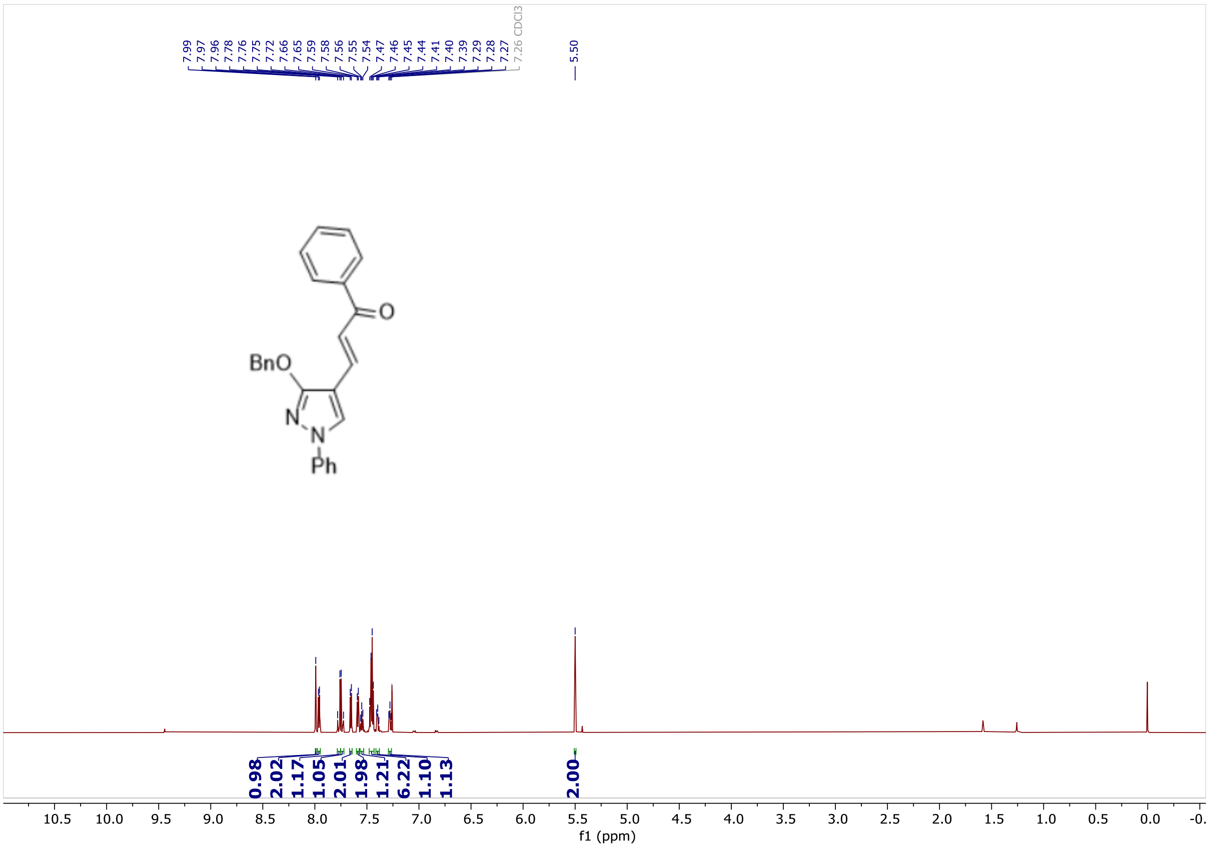

Figure S28. (2*E*)-3-[3-(Benzyloxy)-1-phenyl-1*H*-pyrazol-4-yl]-1-phenylprop-2-en-1-one (4g). ^{1}H NMR spectrum (700 MHz, $CDCl_3$)

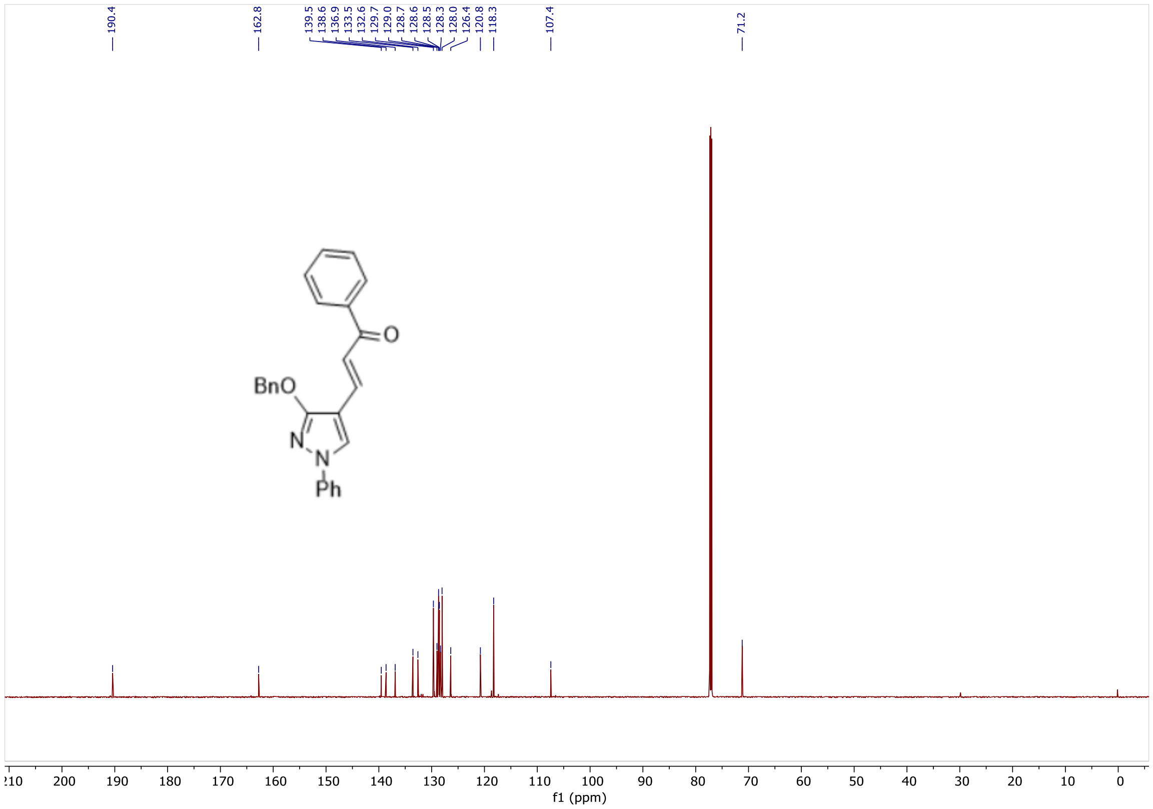

Figure S29. (2*E*)-3-[3-(Benzyloxy)-1-phenyl-1*H*-pyrazol-4-yl]-1-phenylprop-2-en-1-one (4g). ^{13}C NMR spectrum (176 MHz, $CDCl_3$)

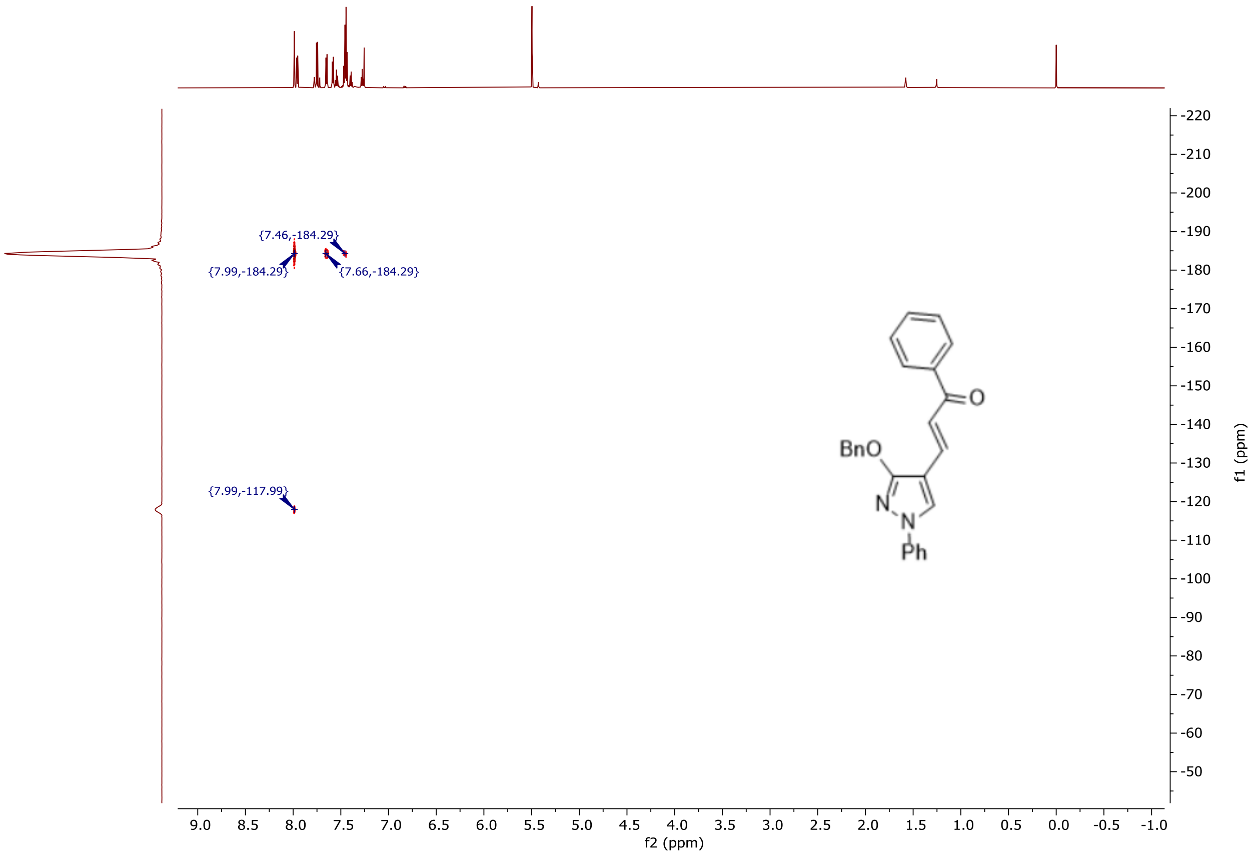

Figure S30. (2*E*)-3-[3-(Benzyloxy)-1-phenyl-1*H*-pyrazol-4-yl]-1-phenylprop-2-en-1-one (4g). ^{1}H-^{15}N HMBC NMR spectrum (71 MHz, $CDCl_3$)

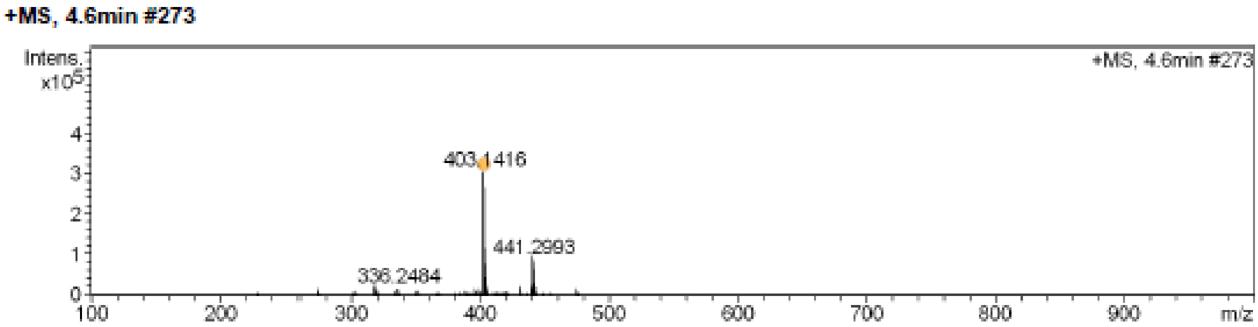

Meas. m/z	#	Ion Formula	m/z	err [ppm]	mSigma	# Sigma	Score	rdb	e⁻ Conf	N-Rule
403.1416	1	C25H20N2NaO2	403.1417	0.3	2.0	1	100.00	16.5	even	ok

Figure S31. (2*E*)-3-[3-(Benzyloxy)-1-phenyl-1*H*-pyrazol-4-yl]-1-phenylprop-2-en-1-one (4g). HRMS (ESI-TOF).

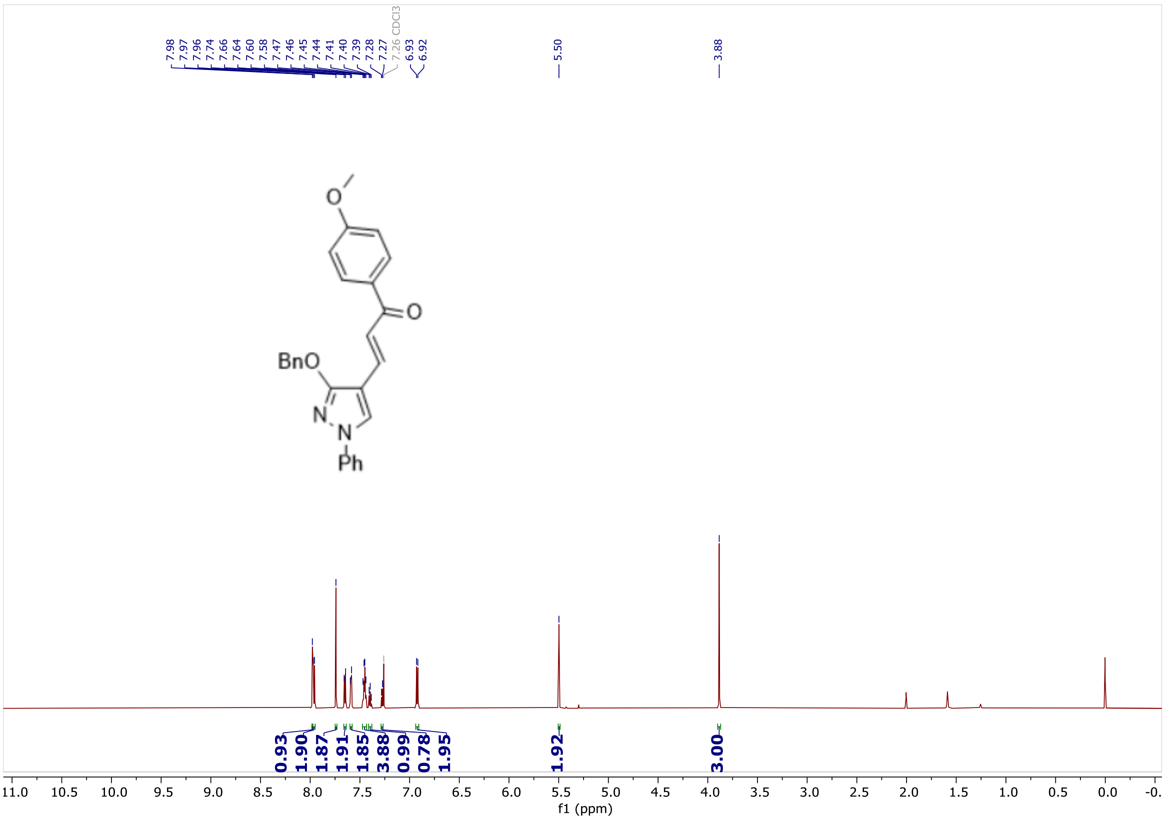

Figure S32. (2*E*)-3-[3-(Benzyloxy)-1-phenyl-1*H*-pyrazol-4-yl]-1-(4-methoxyphenyl)prop-2-en-1-one (4h). ¹H NMR spectrum (700 MHz, CDCl$_3$)

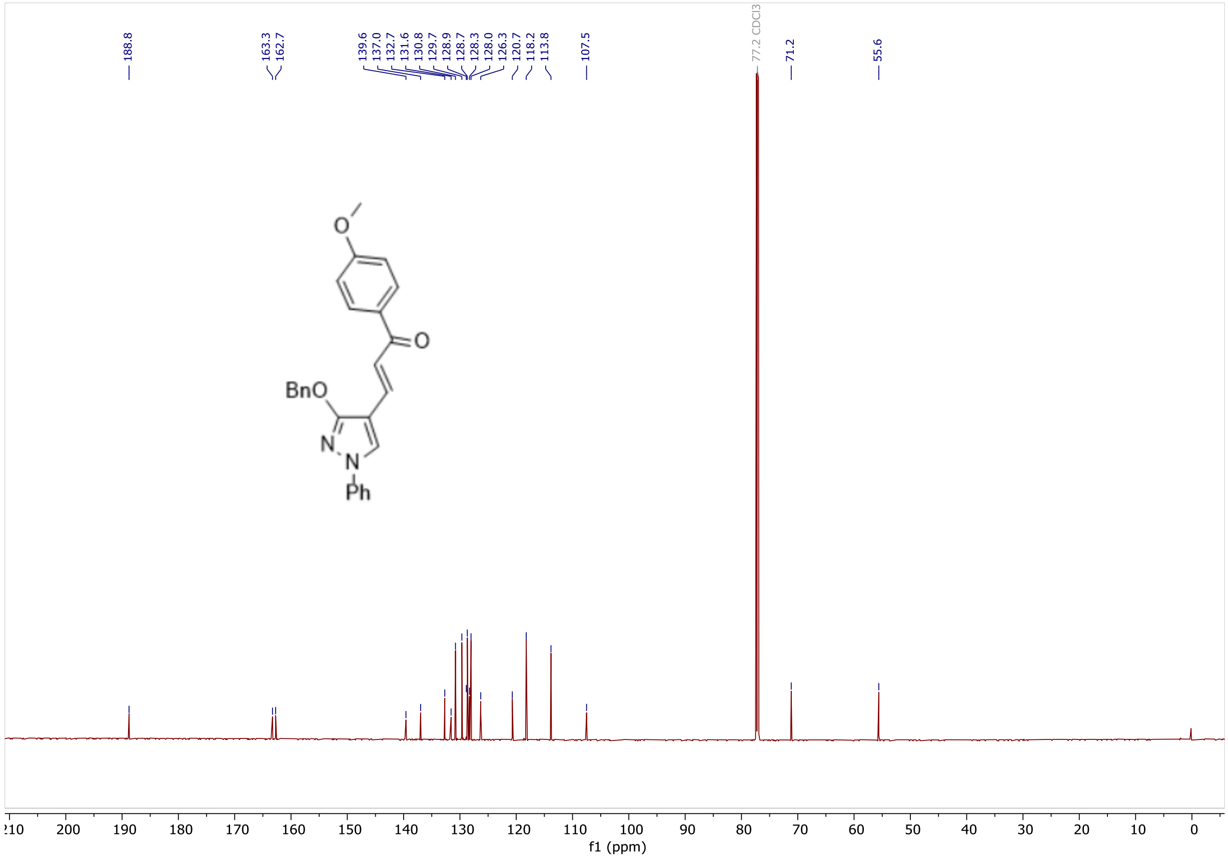

Figure S33. (2*E*)-3-[3-(Benzyloxy)-1-phenyl-1*H*-pyrazol-4-yl]-1-(4-methoxyphenyl)prop-2-en-1-one (4h). ^{13}C NMR spectrum (176 MHz, $CDCl_3$)

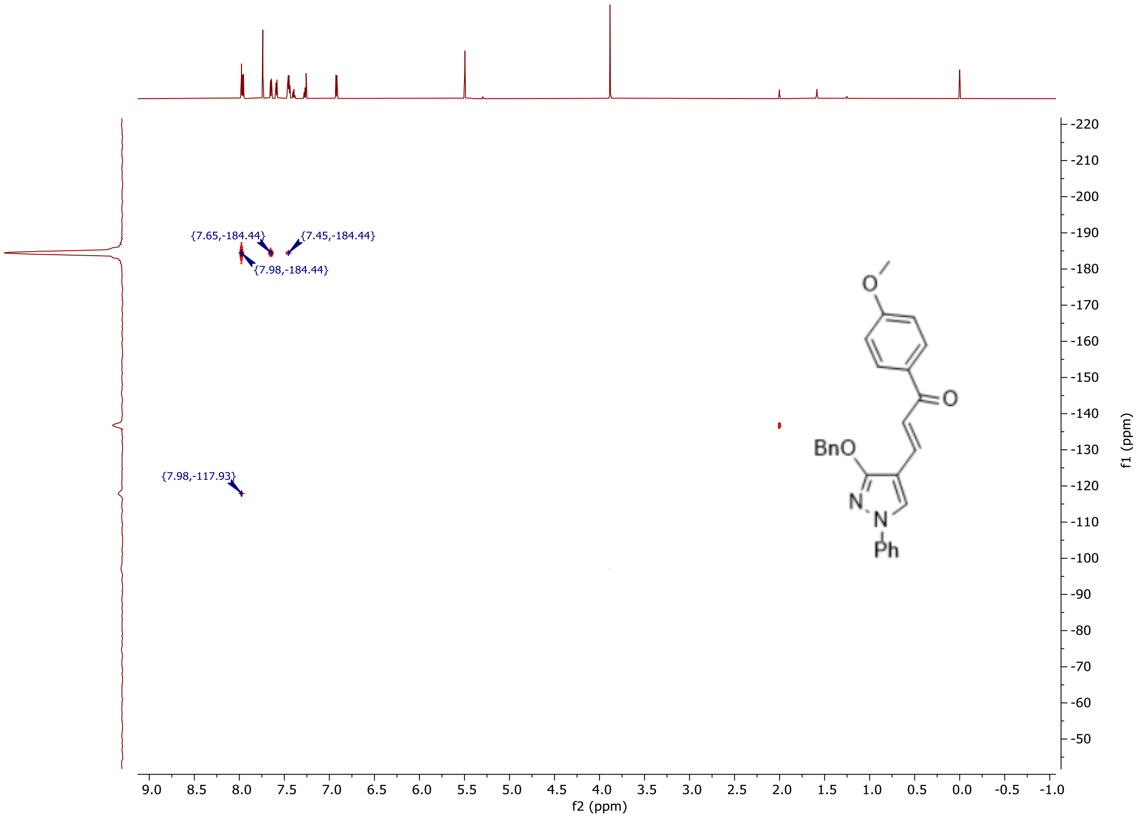

Figure S34. (*E*)-3-[3-(Benzyloxy)-1-phenyl-1*H*-pyrazol-4-yl]-1-(4-methoxyphenyl)prop-2-en-1-one (4h). ^{1}H-^{15}N HMBC NMR spectrum (71 MHz, $CDCl_3$)

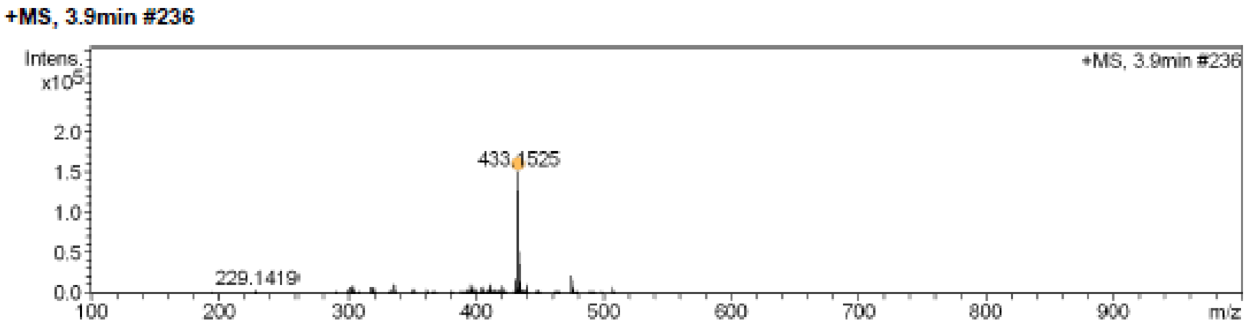

Meas. m/z	#	Ion Formula	m/z	err [ppm]	mSigma	# Sigma	Score	rdb	e⁻ Conf	N-Rule
433.1525	1	C26H22N2NaO3	433.1523	0.6	6.8	1	100.00	16.5	even	ok

Figure S35. (*E*)-3-[3-(Benzyloxy)-1-phenyl-1*H*-pyrazol-4-yl]-1-(4-methoxyphenyl)prop-2-en-1-one (4h). HRMS (ESI-TOF).

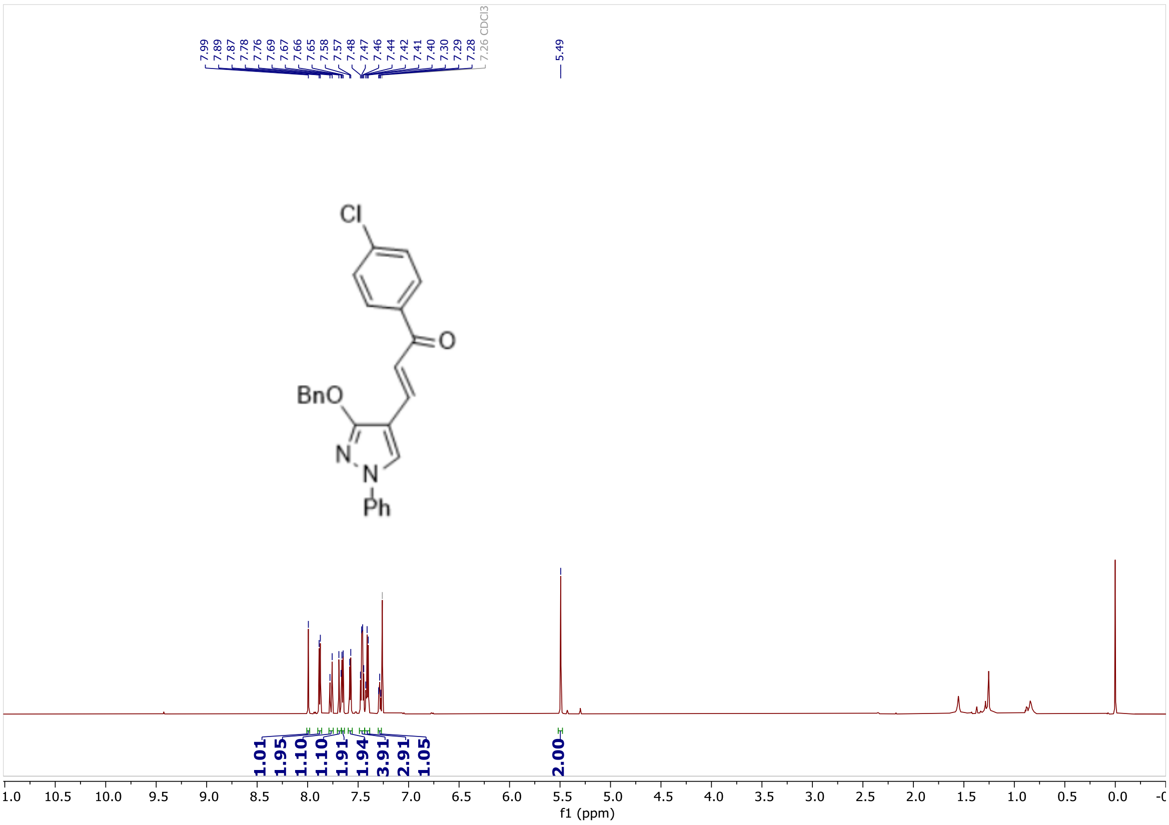

Figure S36. (2*E*)-3-[3-(Benzyloxy)-1-phenyl-1*H*-pyrazol-4-yl]-1-(4-chlorophenyl)prop-2-en-1-one (4i). ^{1}H NMR spectrum (700 MHz, $CDCl_3$)

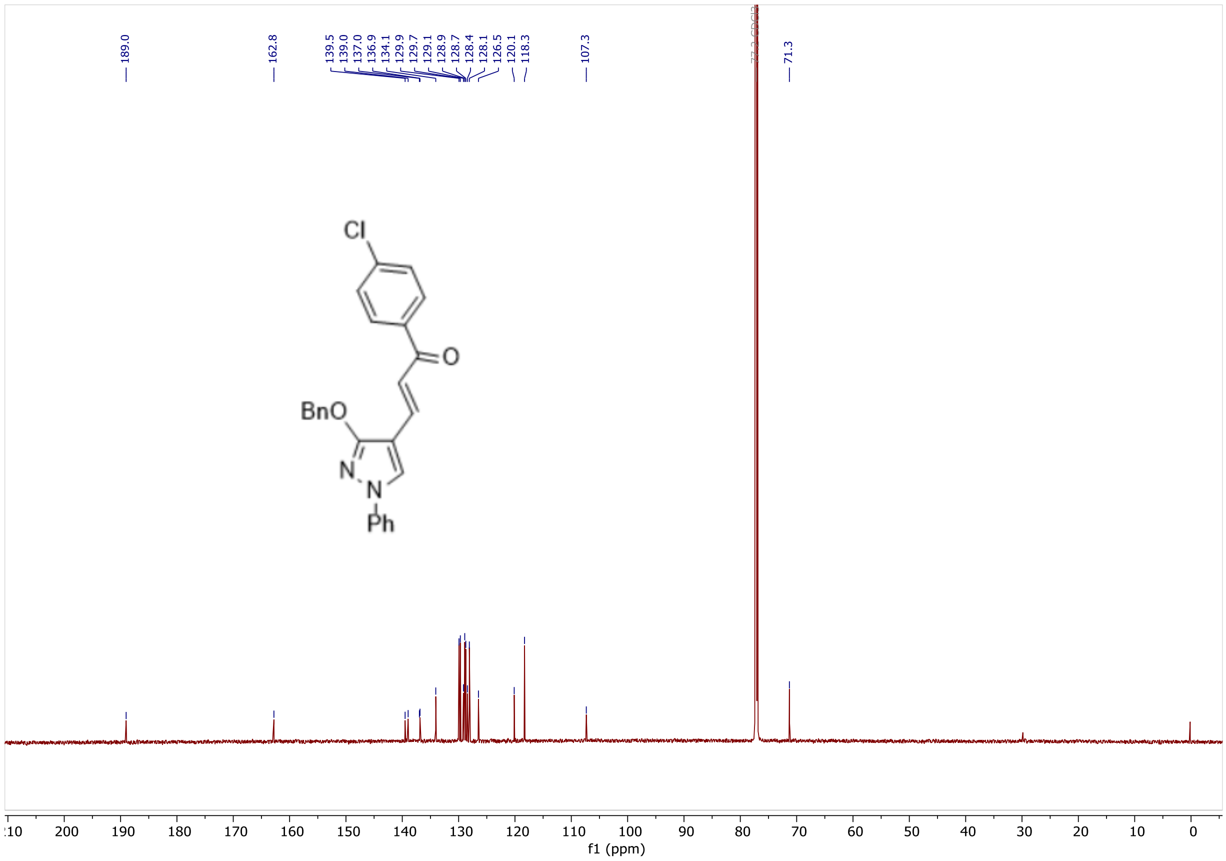

Figure S37. (2*E*)-3-[3-(Benzyloxy)-1-phenyl-1*H*-pyrazol-4-yl]-1-(4-chlorophenyl)prop-2-en-1-one (4i). ^{13}C NMR spectrum (176 MHz, CDCl$_3$)

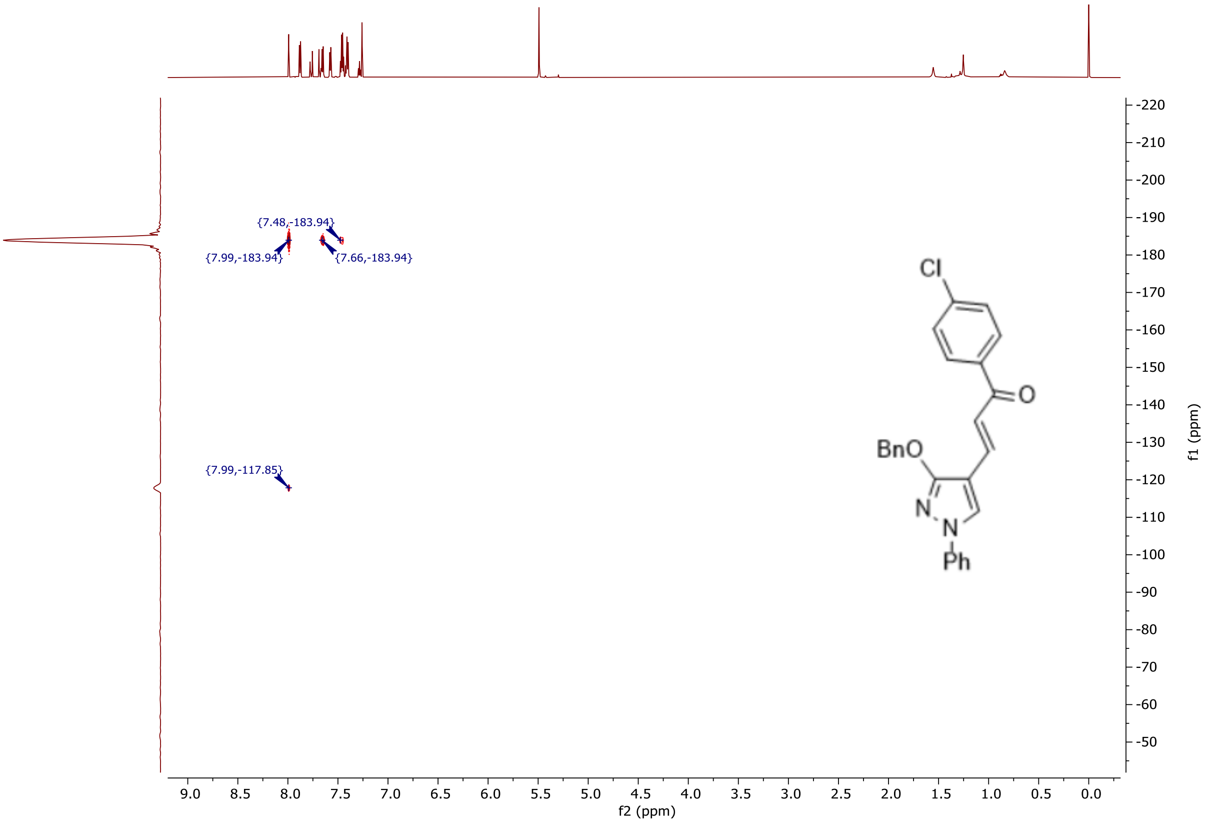

Figure S38. (2*E*)-3-[3-(Benzyloxy)-1-phenyl-1*H*-pyrazol-4-yl]-1-(4-chlorophenyl)prop-2-en-1-one (4i). ^{1}H-^{15}N HMBC NMR spectrum (71 MHz, CDCl$_3$)

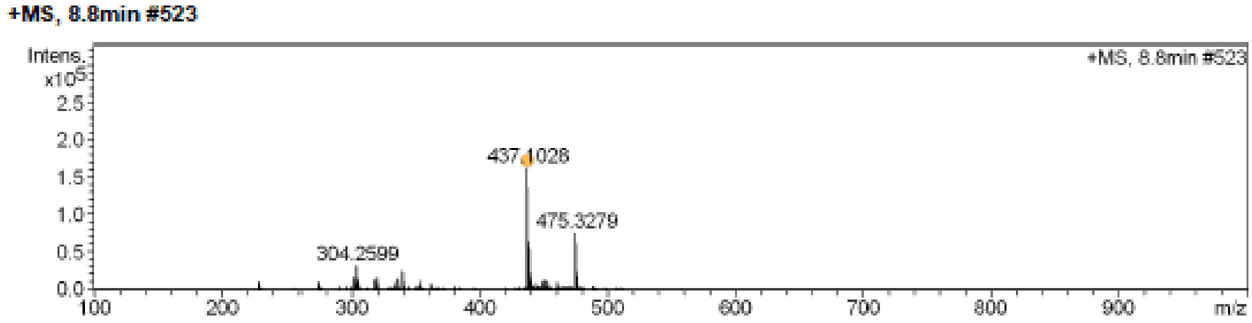

Meas. m/z	#	Ion Formula	m/z	err [ppm]	mSigma	# Sigma	Score	rdb	e⁻ Conf	N-Rule
437.1028	1	C25H19ClN2NaO2	437.1027	-0.1	21.3	2	100.00	16.5	even	ok

Figure S39. (2*E*)-3-[3-(Benzyloxy)-1-phenyl-1*H*-pyrazol-4-yl]-1-(4-chlorophenyl)prop-2-en-1-one (4i). HRMS (ESI-TOF).

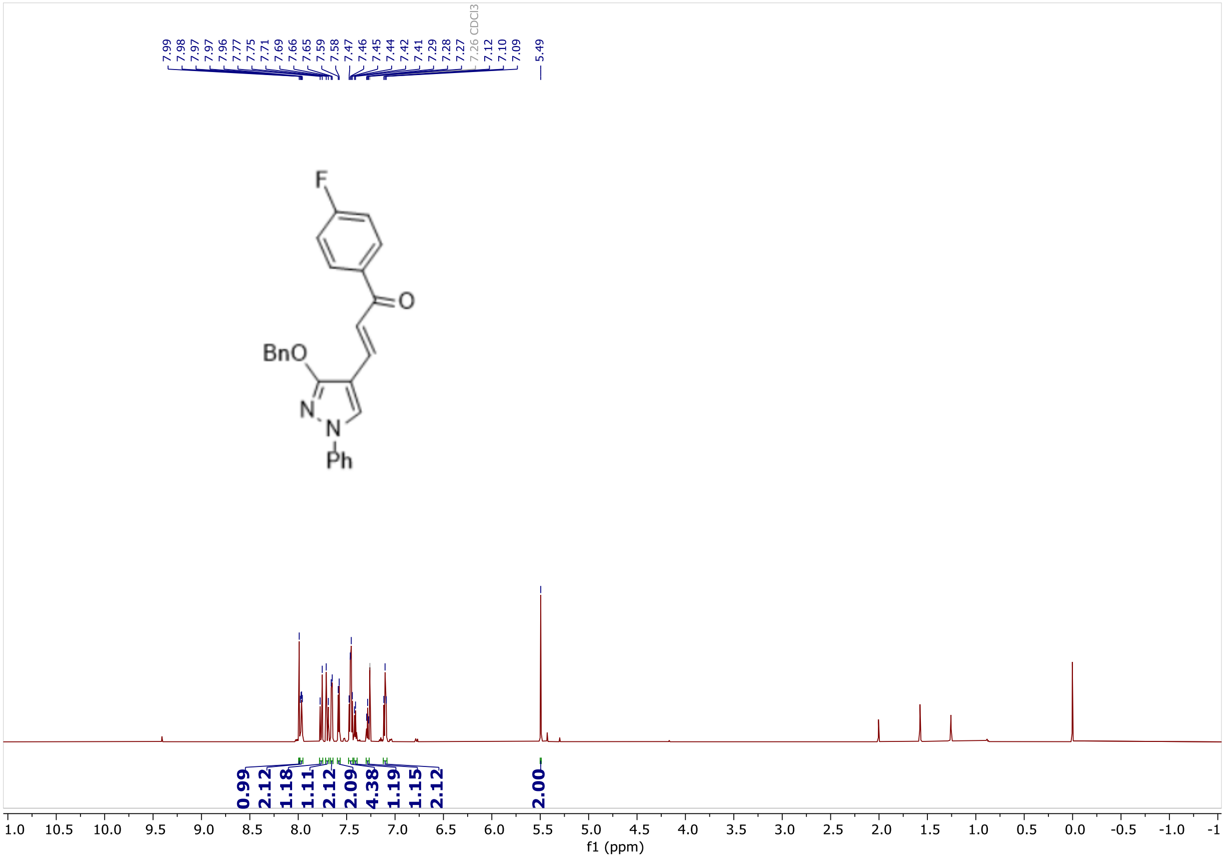

Figure S40. (2*E*)-3-[3-(Benzyloxy)-1-phenyl-1*H*-pyrazol-4-yl]-1-(4-fluorophenyl)prop-2-en-1-one (4j). ^{1}H NMR spectrum (700 MHz, $CDCl_3$)

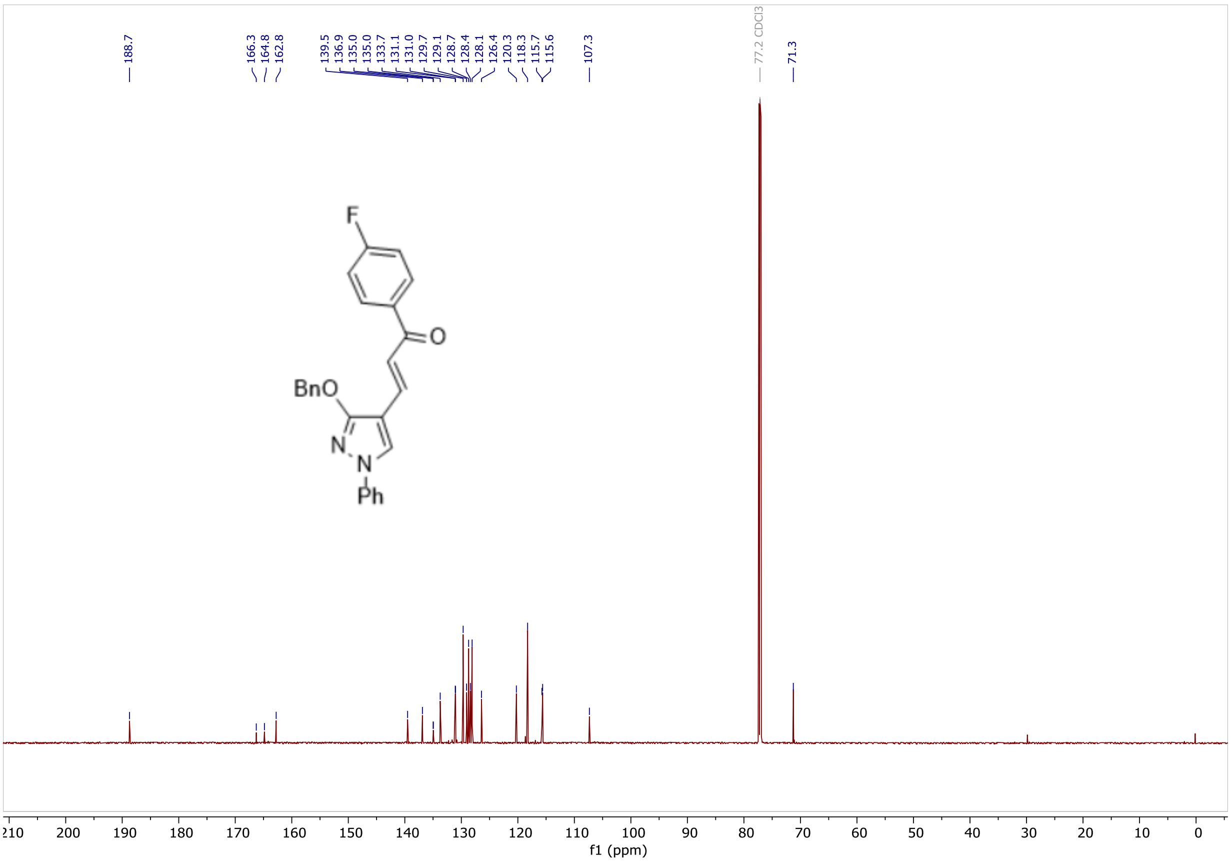

Figure S41. (2*E*)-3-[3-(Benzyloxy)-1-phenyl-1*H*-pyrazol-4-yl]-1-(4-fluorophenyl)prop-2-en-1-one (4j). ^{13}C NMR spectrum (176 MHz, $CDCl_3$)

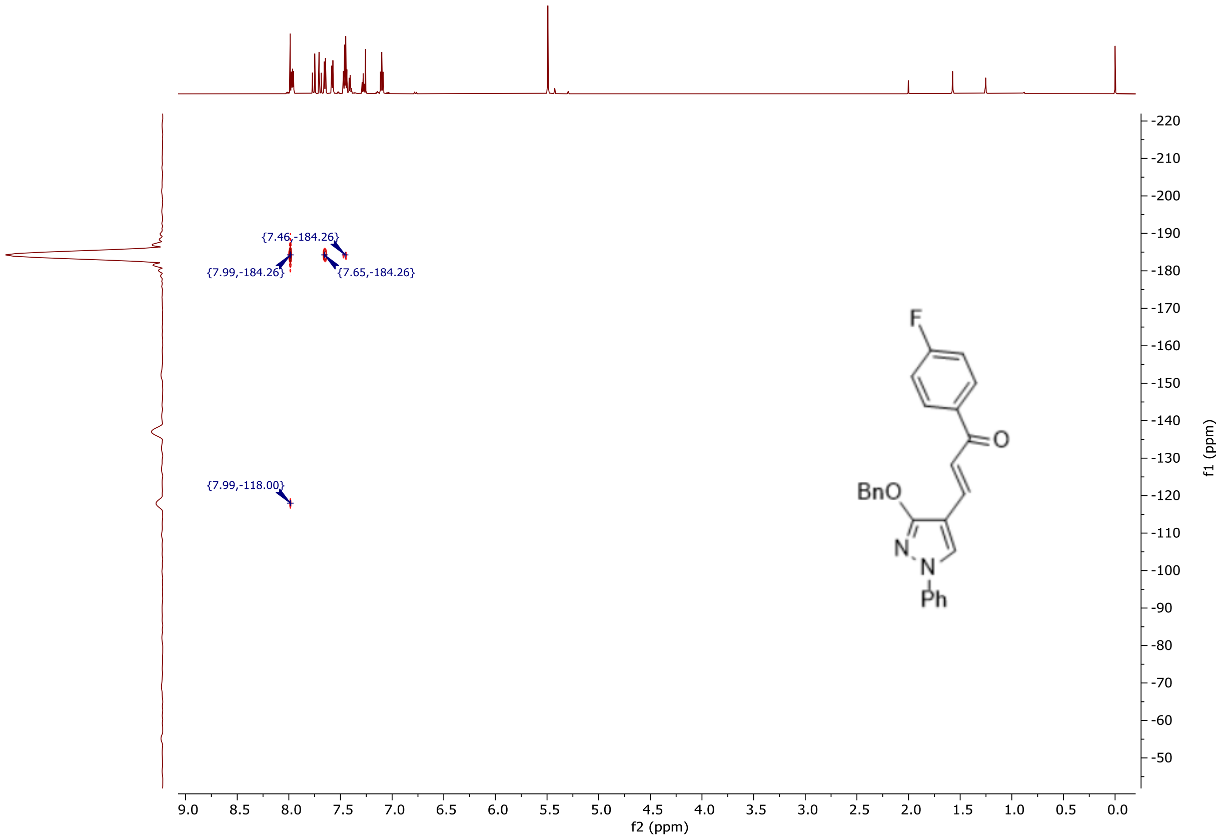

Figure S42. (2*E*)-3-[3-(Benzyloxy)-1-phenyl-1*H*-pyrazol-4-yl]-1-(4-fluorophenyl)prop-2-en-1-one (4j). ^{1}H-^{15}N HMBC NMR spectrum (71 MHz, $CDCl_3$)

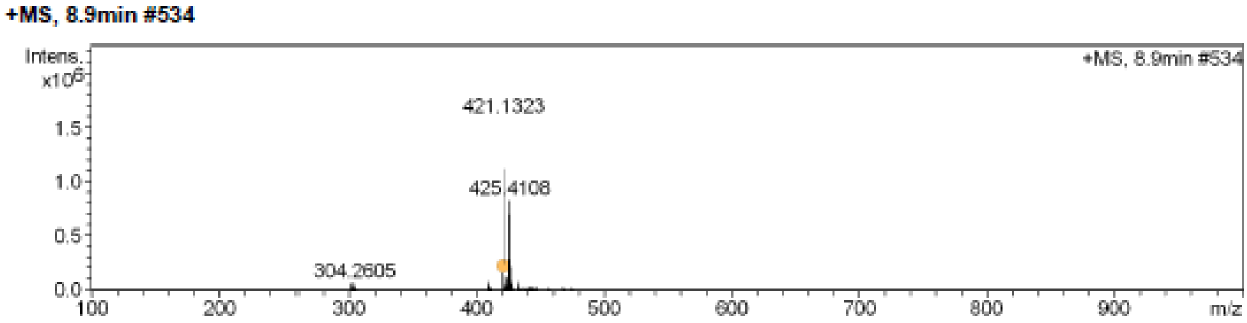

Meas. m/z	#	Ion Formula	m/z	err [ppm]	mSigma	# Sigma	Score	rdb	e⁻ Conf	N-Rule
421.1323	1	C25H19FN2NaO2	421.1323	-0.0	19.1	1	100.00	16.5	even	ok

Figure S43. (2*E*)-3-[3-(Benzyloxy)-1-phenyl-1*H*-pyrazol-4-yl]-1-(4-fluorophenyl)prop-2-en-1-one (4j). HRMS (ESI-TOF).

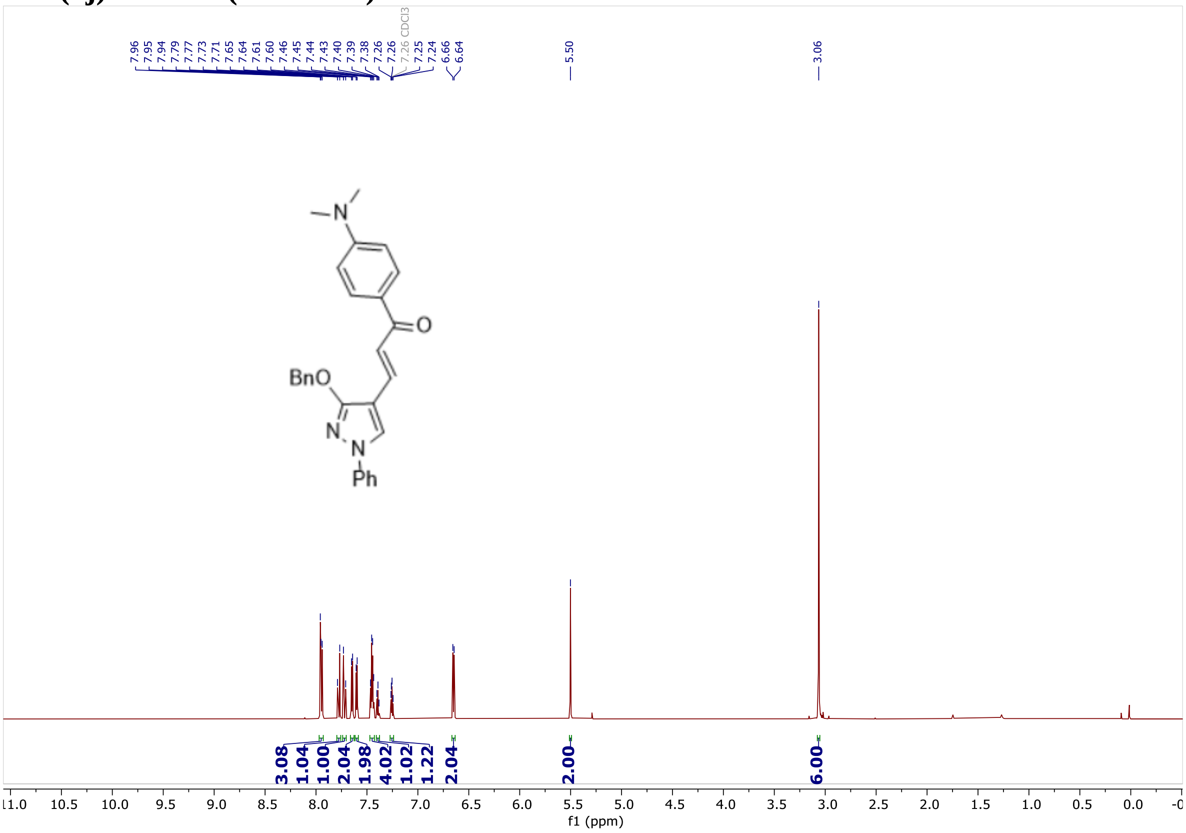

Figure S44. (2*E*)-3-[3-(Benzyloxy)-1-phenyl-1*H*-pyrazol-4-yl]-1-[4-(dimethylamino)phenyl]prop-2-en-1-one (4k). ^{1}H NMR spectrum (700 MHz, $CDCl_3$)

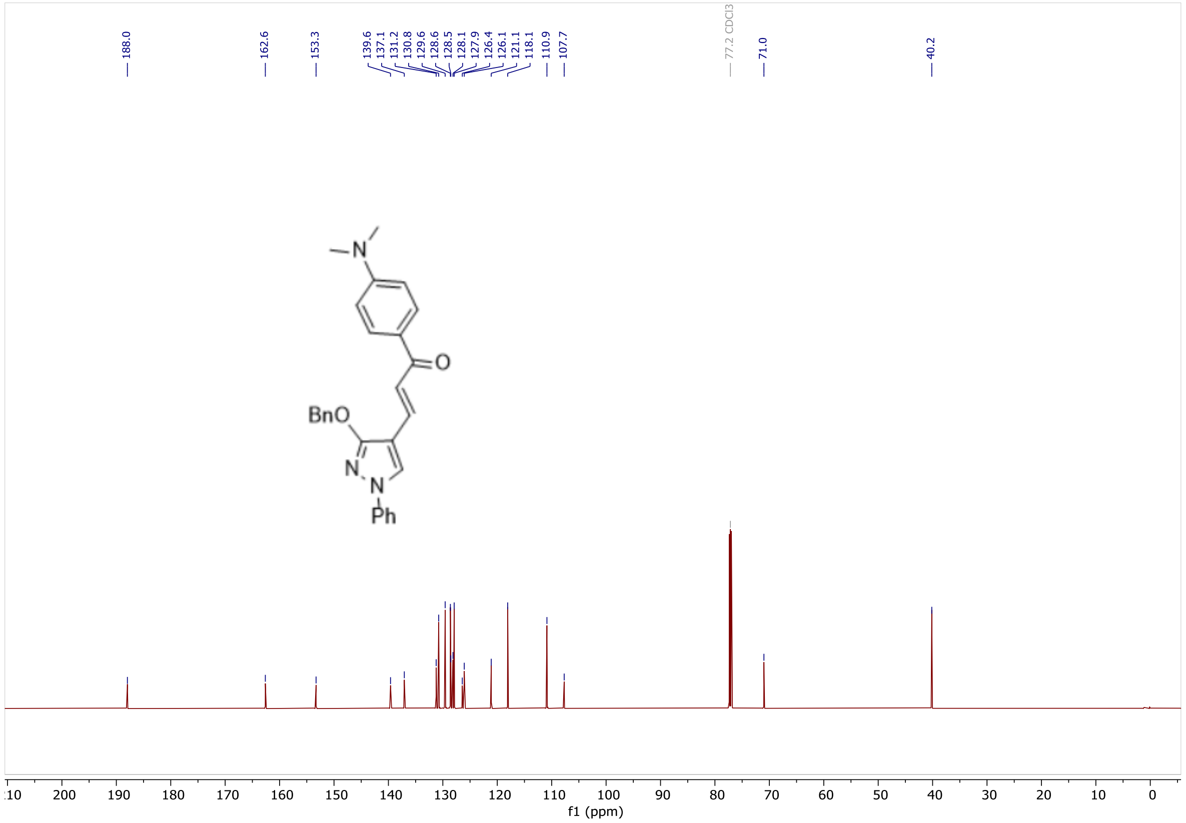

Figure S45. (2*E*)-3-[3-(Benzyloxy)-1-phenyl-1*H*-pyrazol-4-yl]-1-[4-(dimethylamino)phenyl]prop-2-en-1-one (4k). ^{13}C NMR spectrum (176 MHz, $CDCl_3$)

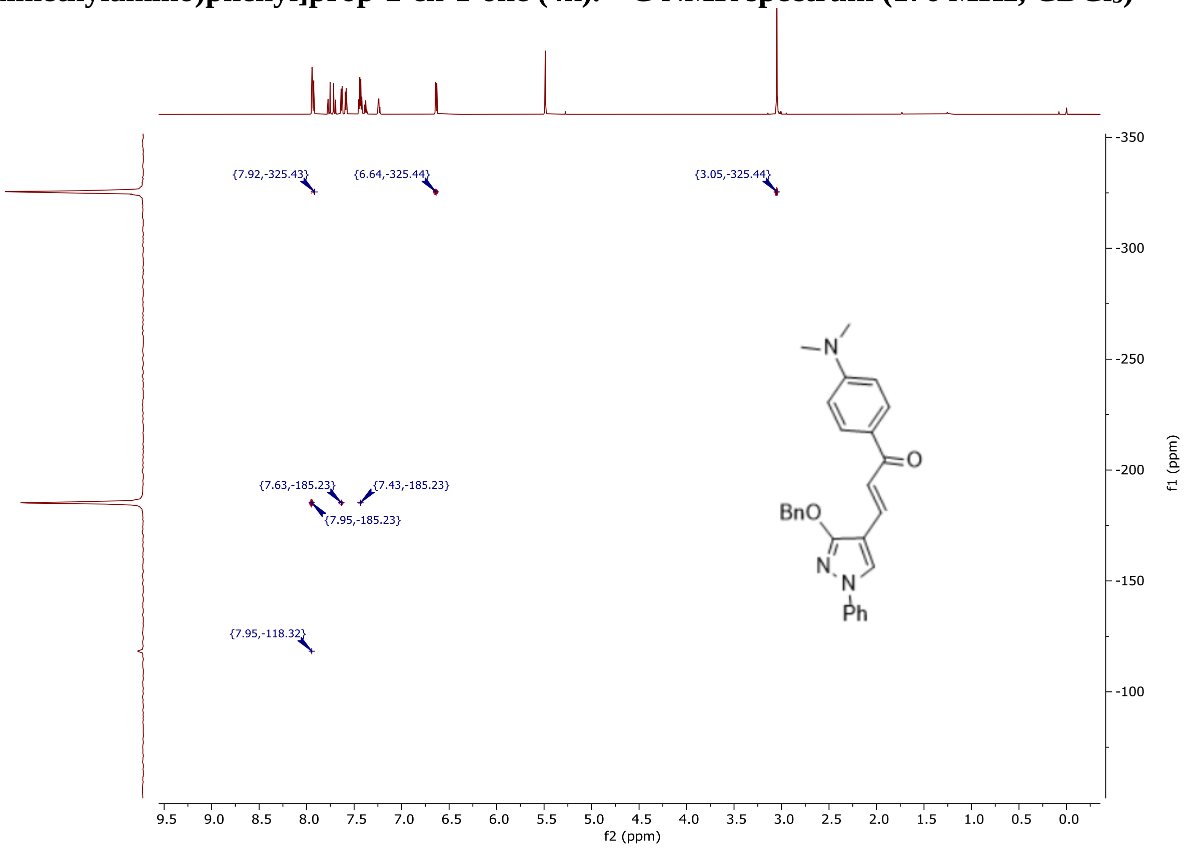

Figure S46. (2*E*)-3-[3-(Benzyloxy)-1-phenyl-1*H*-pyrazol-4-yl]-1-[4-(dimethylamino)phenyl]prop-2-en-1-one (4k). ^{1}H-^{15}N HMBC NMR spectrum (71 MHz, $CDCl_3$)

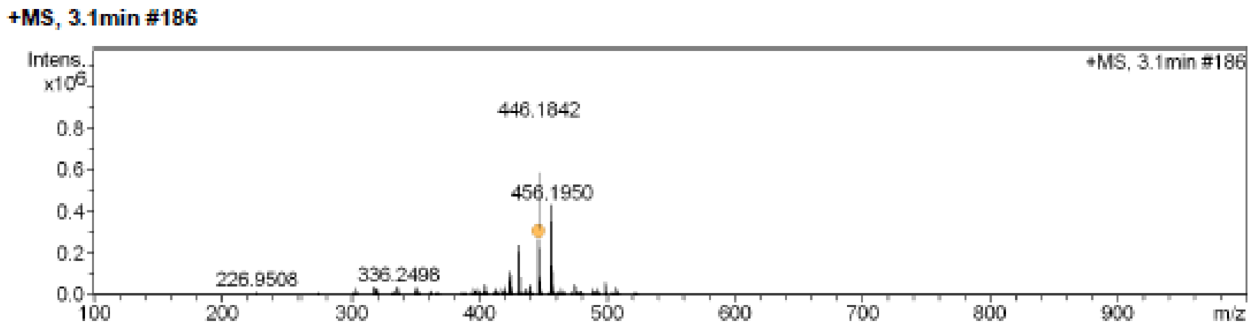

Meas. m/z	#	Ion Formula	m/z	err [ppm]	mSigma	# Sigma	Score	rdb	e⁻ Conf	N-Rule
446.1842	1	C27H25N3NaO2	446.1839	0.7	3.6	1	100.00	16.5	even	ok

Figure S47. (2*E*)-3-[3-(Benzyloxy)-1-phenyl-1*H*-pyrazol-4-yl]-1-[4-(dimethylamino)phenyl]prop-2-en-1-one (4k). HRMS (ESI-TOF).

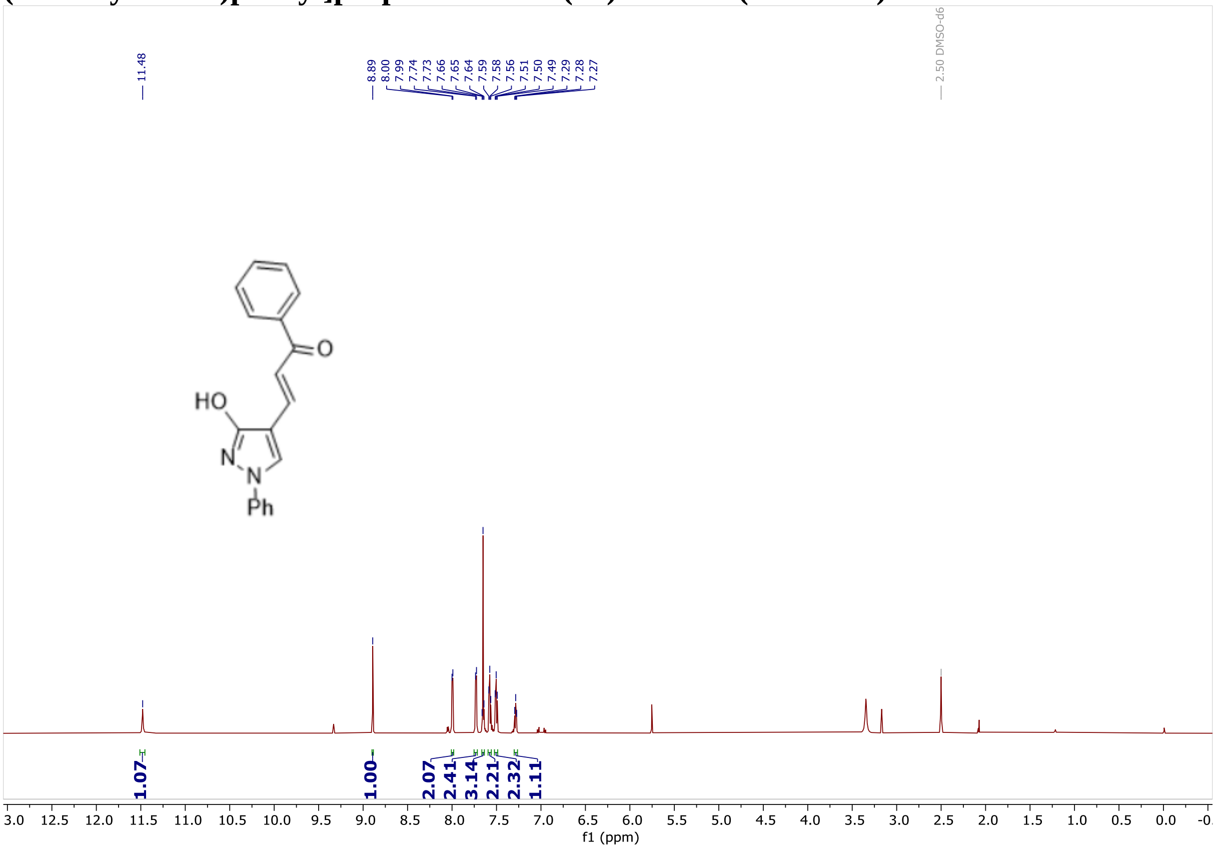

Figure S48. (2*E*)-3-(3-Hydroxy-1-phenyl-1*H*-pyrazol-4-yl)-1-phenylprop-2-en-1-one (5a). ^{1}H NMR spectrum (700 MHz, DMSO-d_6)

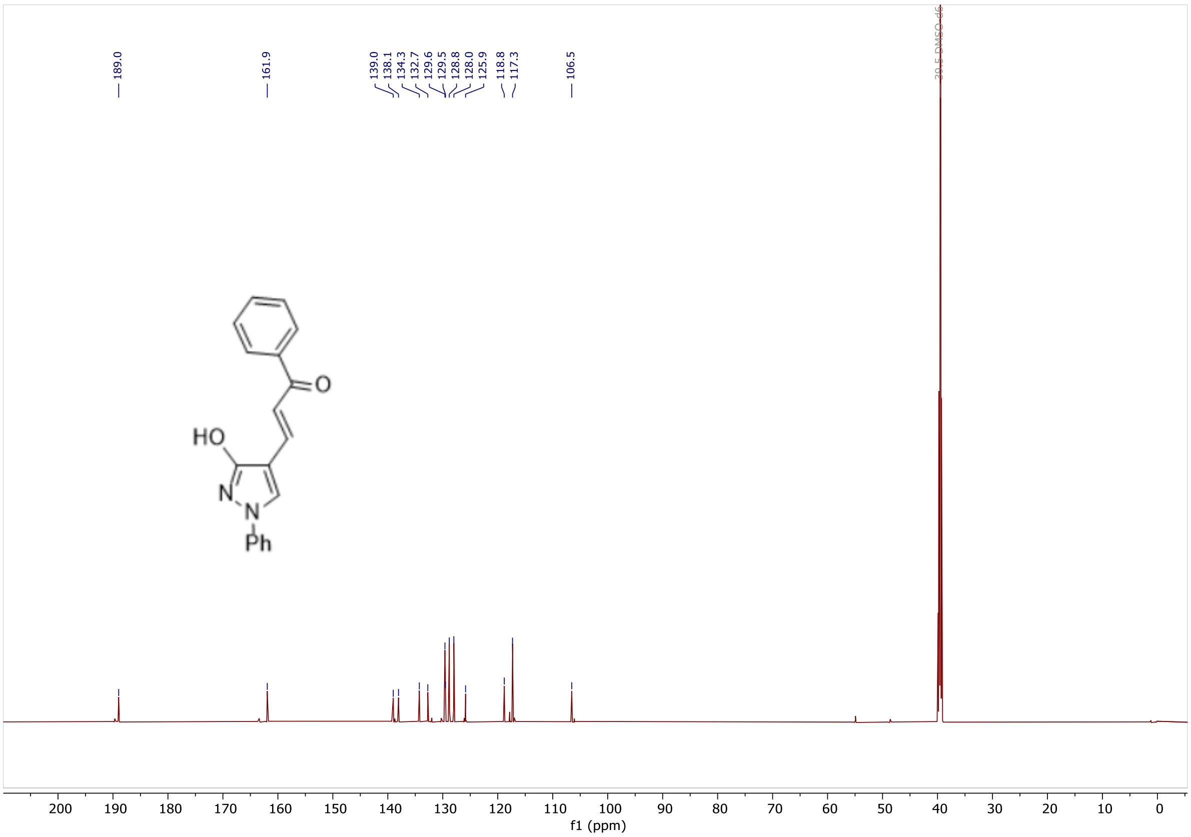

Figure S49. (2*E*)-3-(3-Hydroxy-1-phenyl-1*H*-pyrazol-4-yl)-1-phenylprop-2-en-1-one (5a). ^{13}C NMR spectrum (176 MHz, DMSO-*d*$_6$)

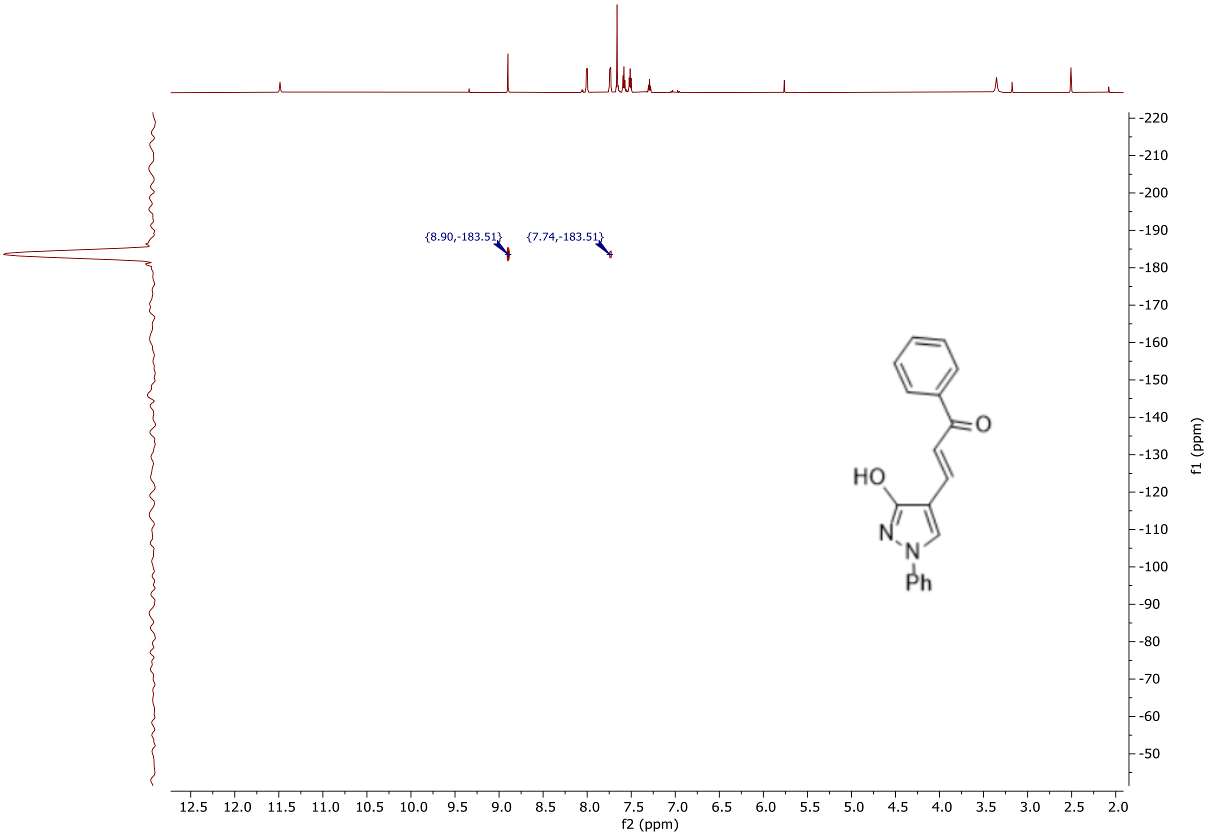

Figure S50. (2*E*)-3-(3-Hydroxy-1-phenyl-1*H*-pyrazol-4-yl)-1-phenylprop-2-en-1-one (5a). ^{1}H-^{15}N HMBC NMR spectrum (71 MHz, DMSO-*d*$_6$)

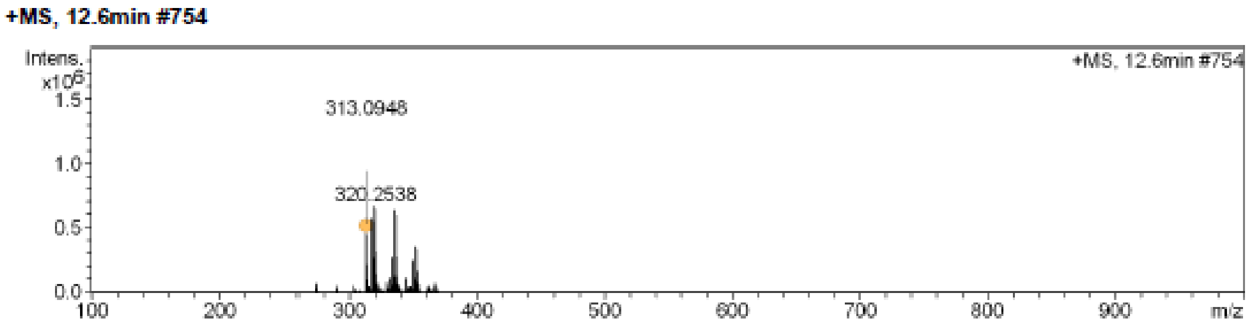

Meas. m/z	#	Ion Formula	m/z	err [ppm]	mSigma	# Sigma	Score	rdb	e⁻ Conf	N-Rule
313.0948	1	C18H14N2NaO2	313.0947	0.1	14.7	1	100.00	12.5	even	ok

Figure S51. (2*E*)-3-(3-Hydroxy-1-phenyl-1*H*-pyrazol-4-yl)-1-phenylprop-2-en-1-one (5a). HRMS (ESI-TOF).

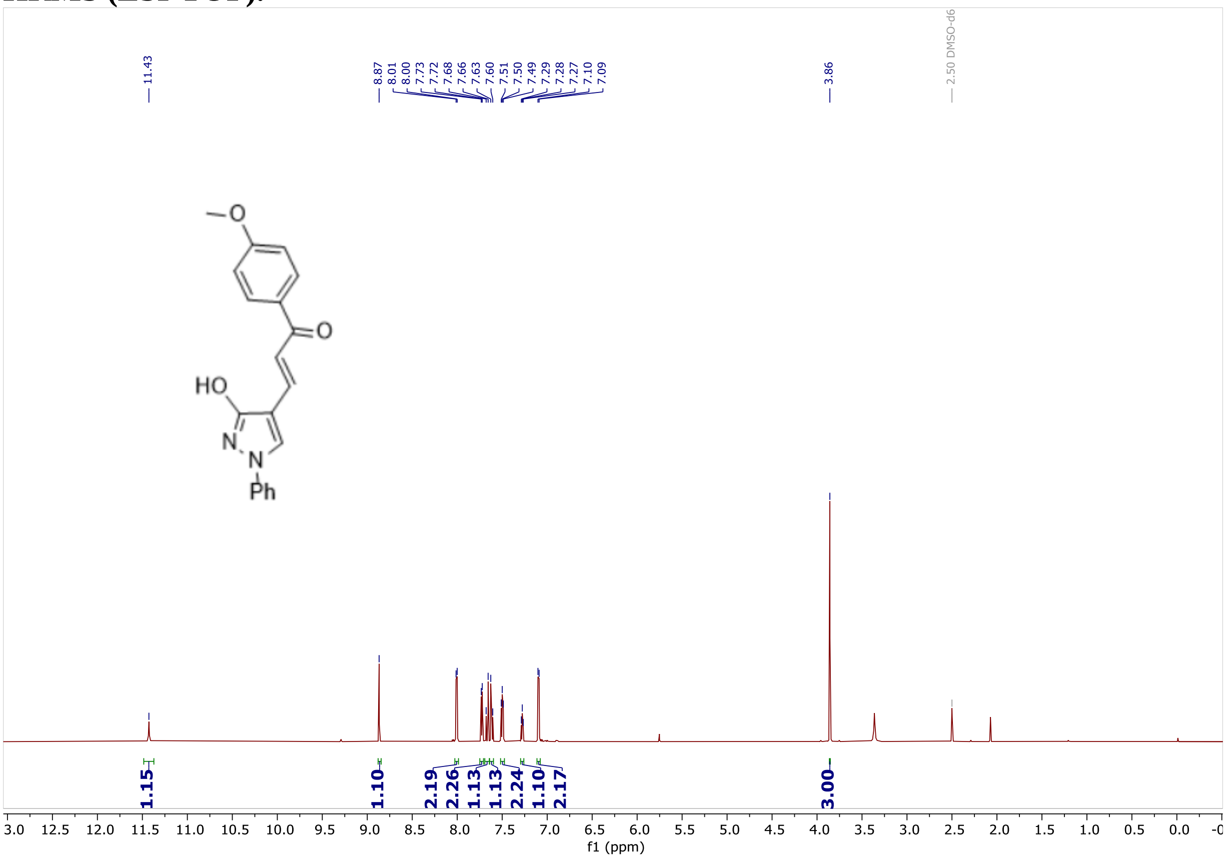

Figure S52. (2*E*)-3-(3-Hydroxy-1-phenyl-1*H*-pyrazol-4-yl)-1-(4-methoxyphenyl)prop-2-en-1-one (5b). ^{1}H NMR spectrum (700 MHz, DMSO-d_6)

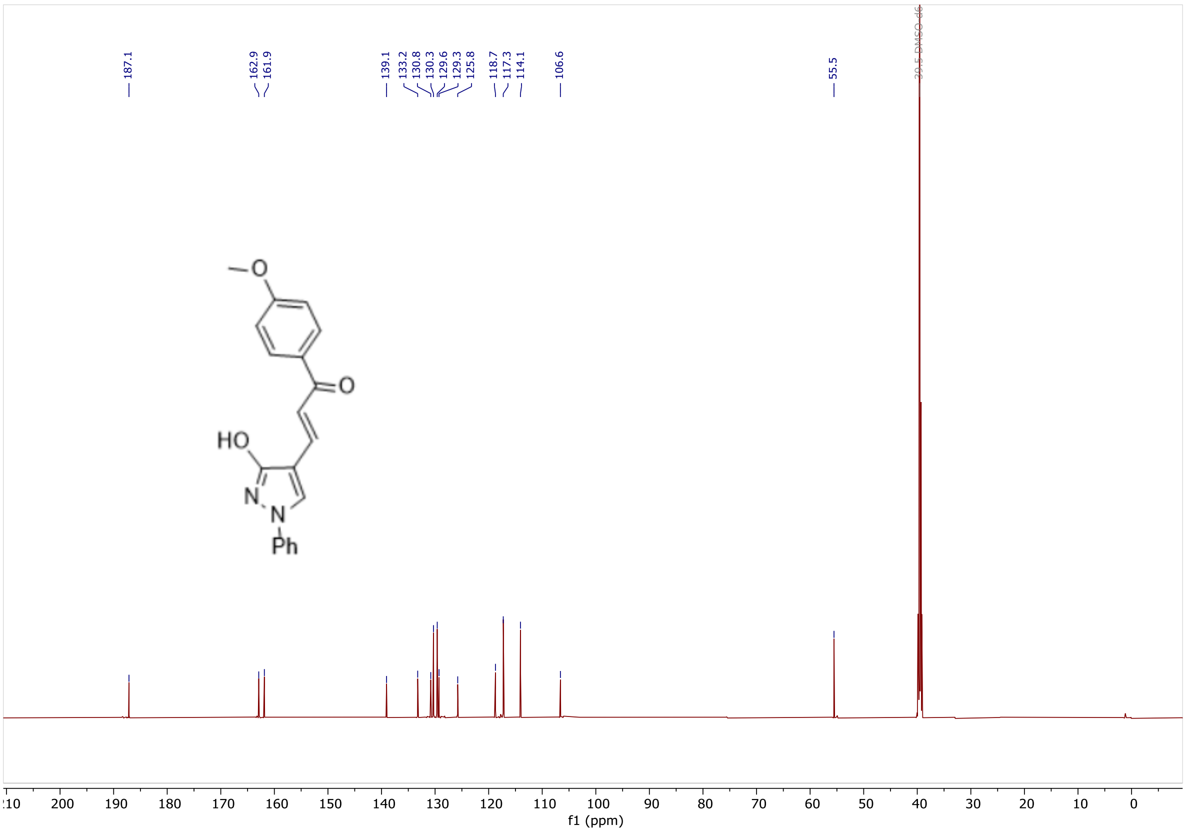

Figure S53. (2*E*)-3-(3-Hydroxy-1-phenyl-1*H*-pyrazol-4-yl)-1-(4-methoxyphenyl)prop-2-en-1-one (5b). ^{13}C NMR spectrum (176 MHz, DMSO-*d*$_6$)

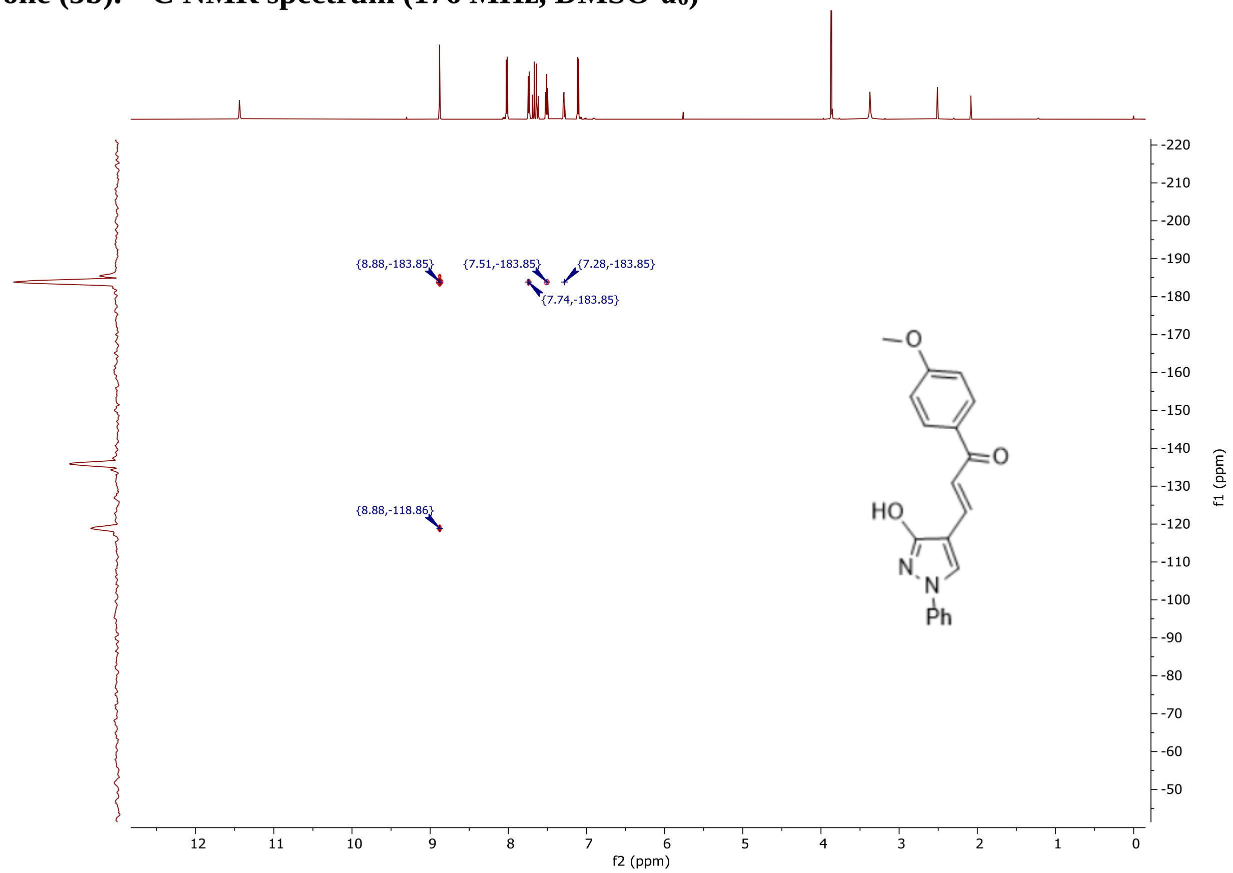

Figure S54. (2*E*)-3-(3-Hydroxy-1-phenyl-1*H*-pyrazol-4-yl)-1-(4-methoxyphenyl)prop-2-en-1-one (5b). ^{1}H-^{15}N HMBC NMR spectrum (71 MHz, DMSO-*d*$_6$)

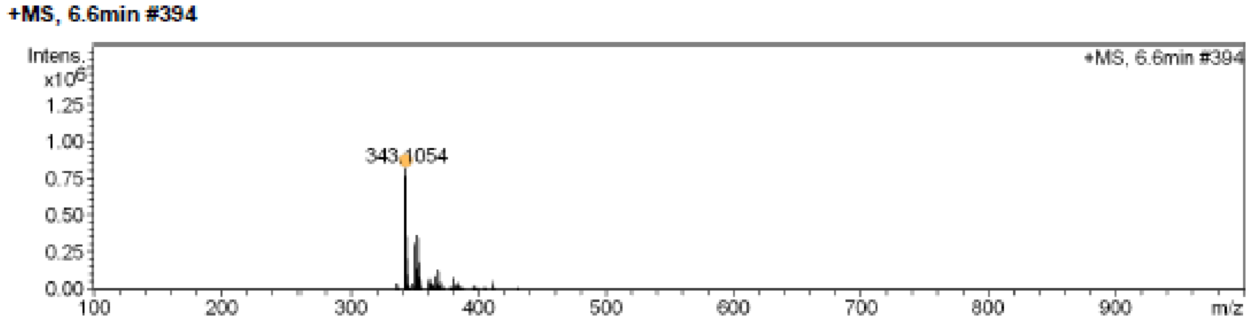

Meas. m/z	#	Ion Formula	m/z	err [ppm]	mSigma	# Sigma	Score	rdb	e⁻ Conf	N-Rule
343.1054	1	C19H16N2NaO3	343.1053	0.3	30.8	2	100.00	12.5	even	ok

Figure S55. (2*E*)-3-(3-Hydroxy-1-phenyl-1*H*-pyrazol-4-yl)-1-(4-methoxyphenyl)prop-2-en-1-one (5b). HRMS (ESI-TOF).

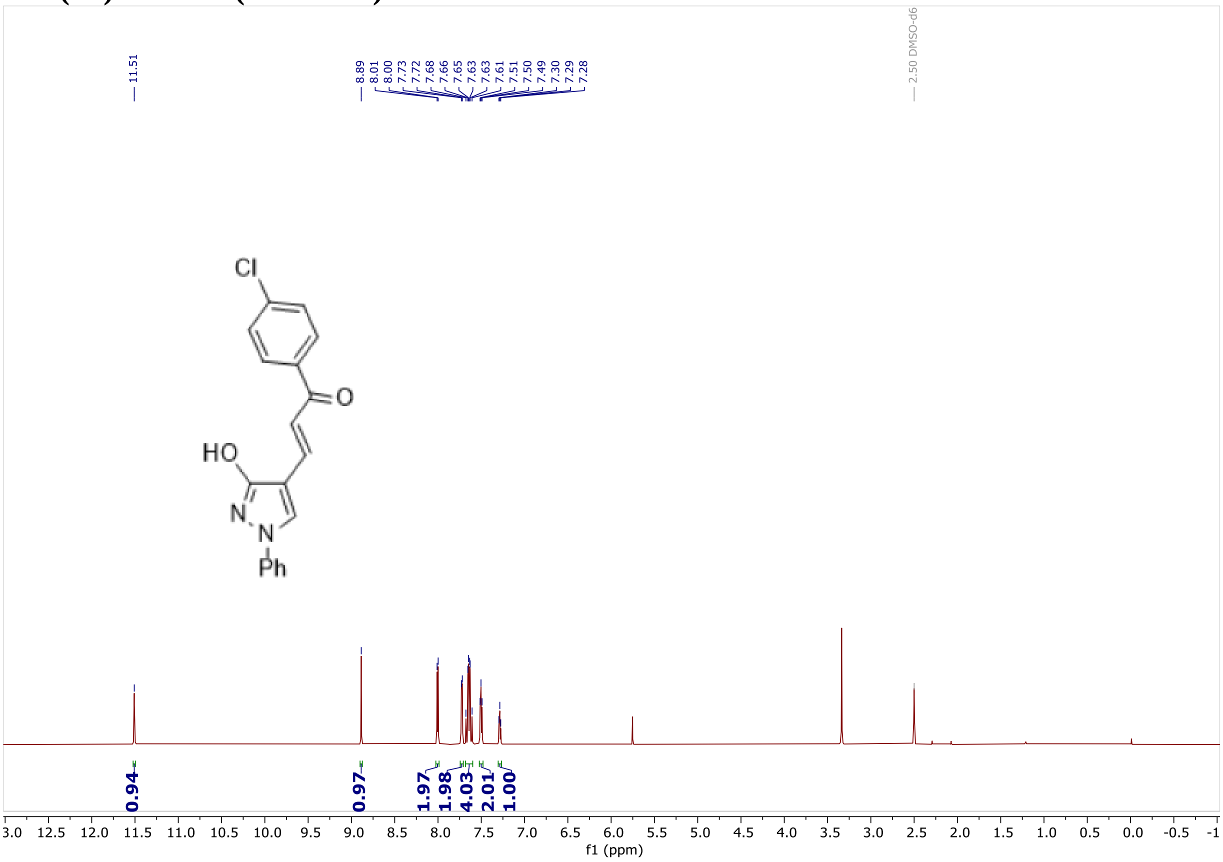

Figure S56. (2*E*)-1-(4-Chlorophenyl)-3-(3-hydroxy-1-phenyl-1*H*-pyrazol-4-yl)prop-2-en-1-one (5c). ^{1}H NMR spectrum (700 MHz, DMSO-*d*$_6$)

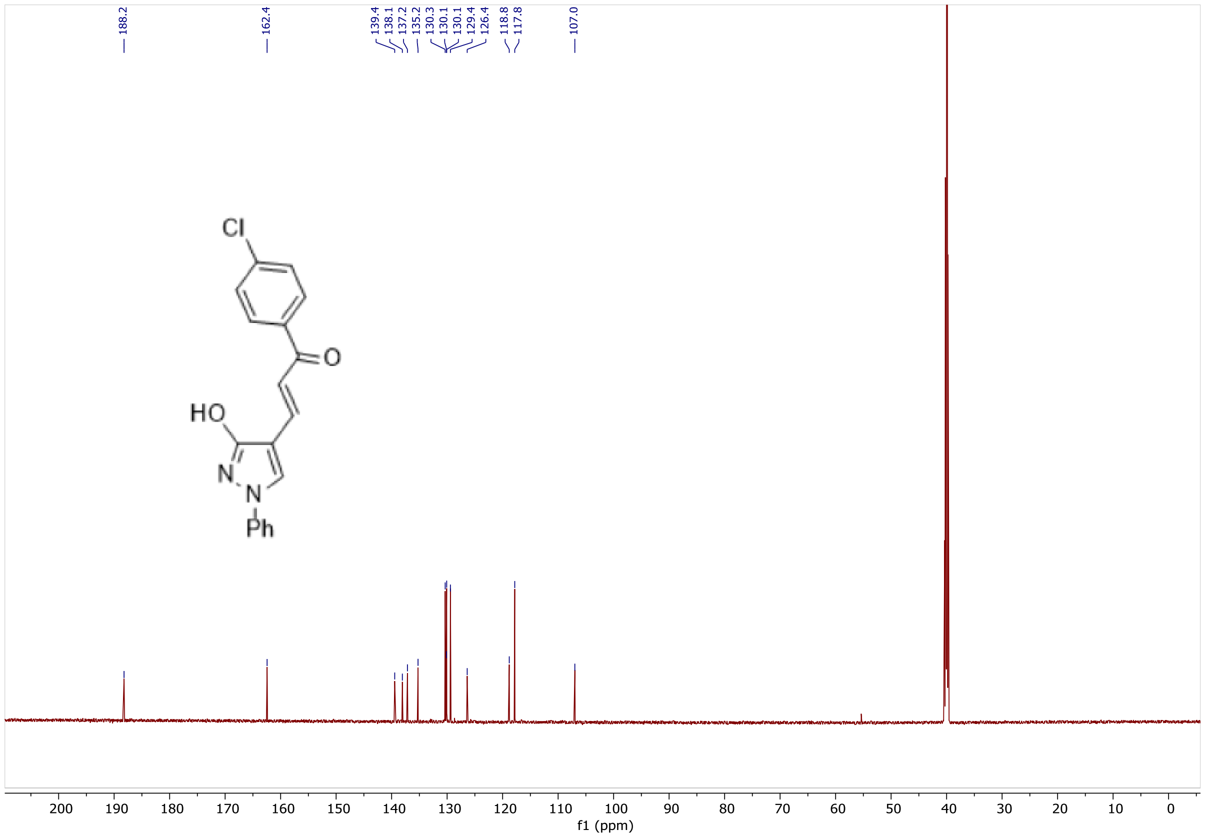

Figure S57. (2*E*)-1-(4-Chlorophenyl)-3-(3-hydroxy-1-phenyl-1*H*-pyrazol-4-yl)prop-2-en-1-one (5c). ^{13}C NMR spectrum (176 MHz, DMSO-d_6)

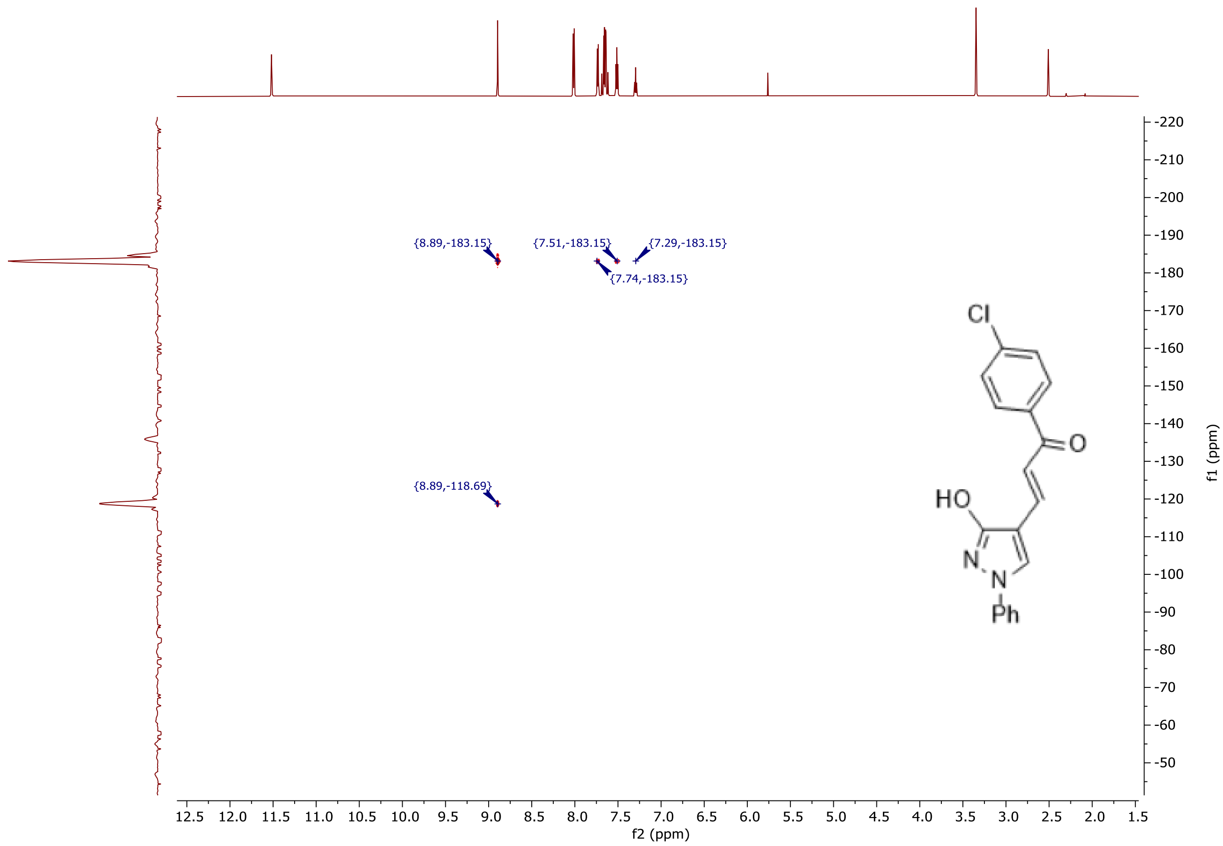

Figure S58. (2*E*)-1-(4-Chlorophenyl)-3-(3-hydroxy-1-phenyl-1*H*-pyrazol-4-yl)prop-2-en-1-one (5c). ^{1}H-^{15}N HMBC NMR spectrum (71 MHz, DMSO-d_6)

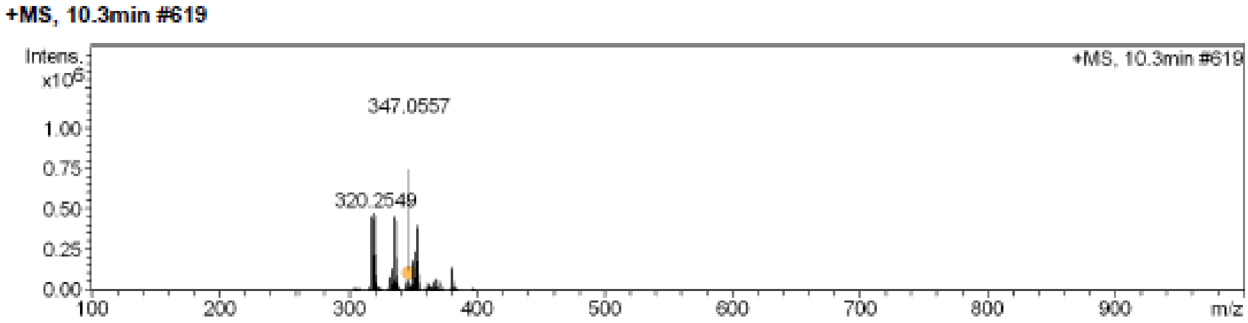

Meas. m/z	#	Ion Formula	m/z	err [ppm]	mSigma	# Sigma	Score	rdb	e¯ Conf	N-Rule
347.0557	1	C18H13ClN2NaO2	347.0558	0.1	26.6	1	100.00	12.5	even	ok

Figure S59. (2*E*)-1-(4-Chlorophenyl)-3-(3-hydroxy-1-phenyl-1*H*-pyrazol-4-yl)prop-2-en-1-one (5c). HRMS (ESI-TOF).

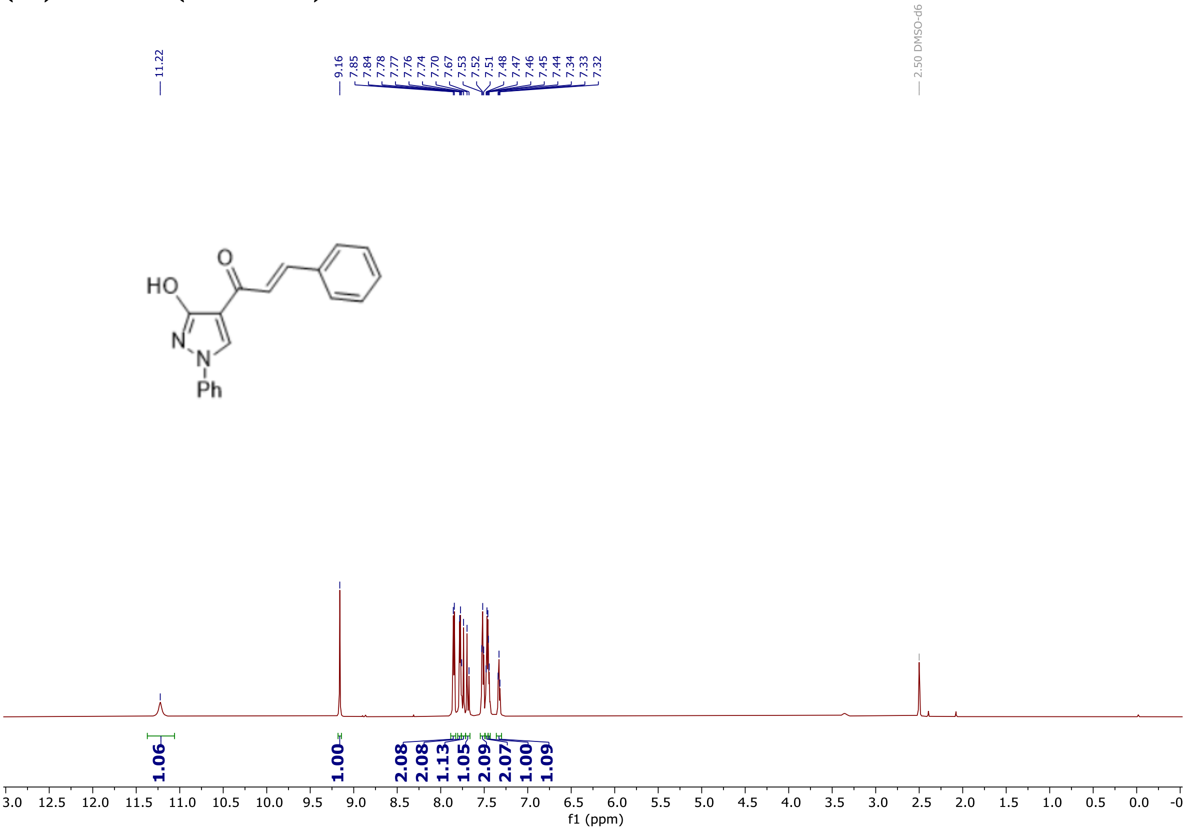

Figure S60. (2*E*)-1-(3-Hydroxy-1-phenyl-1*H*-pyrazol-4-yl)-3-phenylprop-2-en-1-one (8a) ^{1}H NMR spectrum (700 MHz, DMSO-d_6)

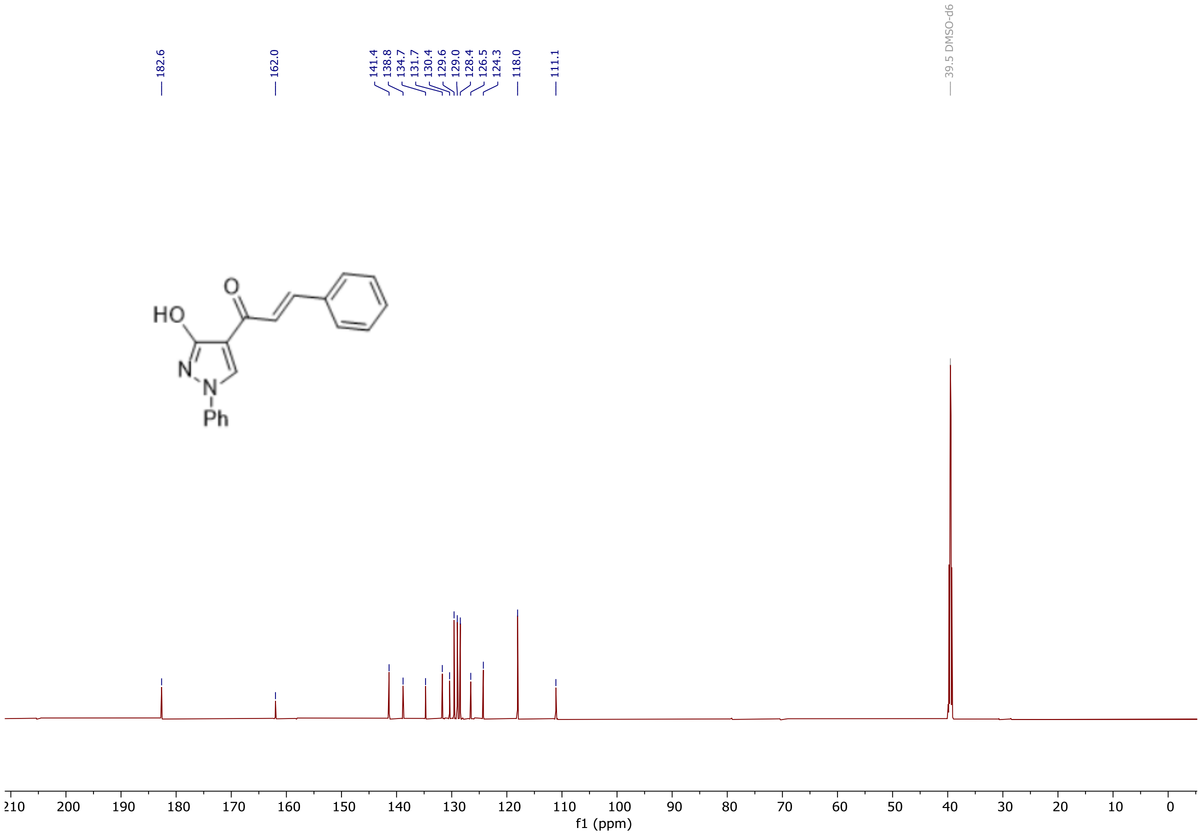

Figure S61. (2*E*)-1-(3-Hydroxy-1-phenyl-1*H*-pyrazol-4-yl)-3-phenylprop-2-en-1-one (8a). ^{13}C NMR spectrum (176 MHz, DMSO-*d*$_6$)

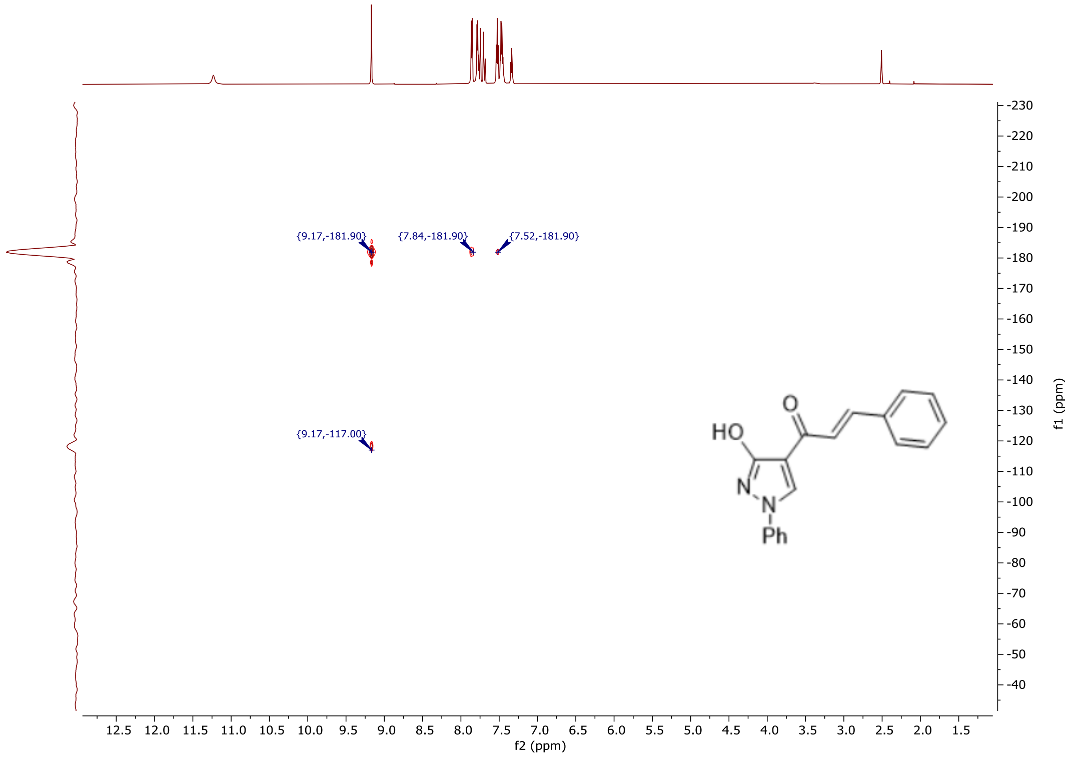

Figure S62. (2*E*)-1-(3-Hydroxy-1-phenyl-1*H*-pyrazol-4-yl)-3-phenylprop-2-en-1-one (8a). ^{1}H-^{15}N HMBC NMR spectrum (71 MHz, DMSO-*d*$_6$)

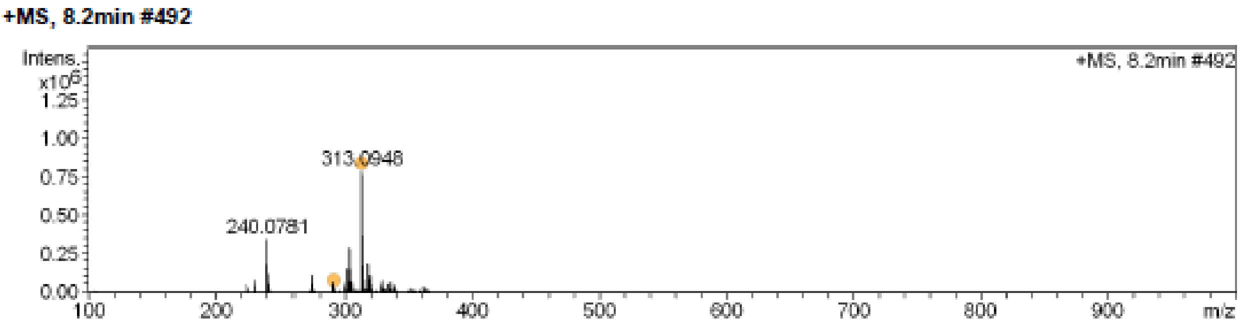

Meas. m/z	#	Ion Formula	m/z	err [ppm]	mSigma	# Sigma	Score	rdb	e⁻ Conf	N-Rule
291.1121	1	C18H15N2O2	291.1128	-2.5	4.9	1	100.00	12.5	even	ok
313.0948	1	C18H14N2NaO2	313.0947	0.3	19.0	1	100.00	12.5	even	ok

Figure S63. (2*E*)-1-(3-Hydroxy-1-phenyl-1*H*-pyrazol-4-yl)-3-phenylprop-2-en-1-one (8a). HRMS (ESI-TOF).

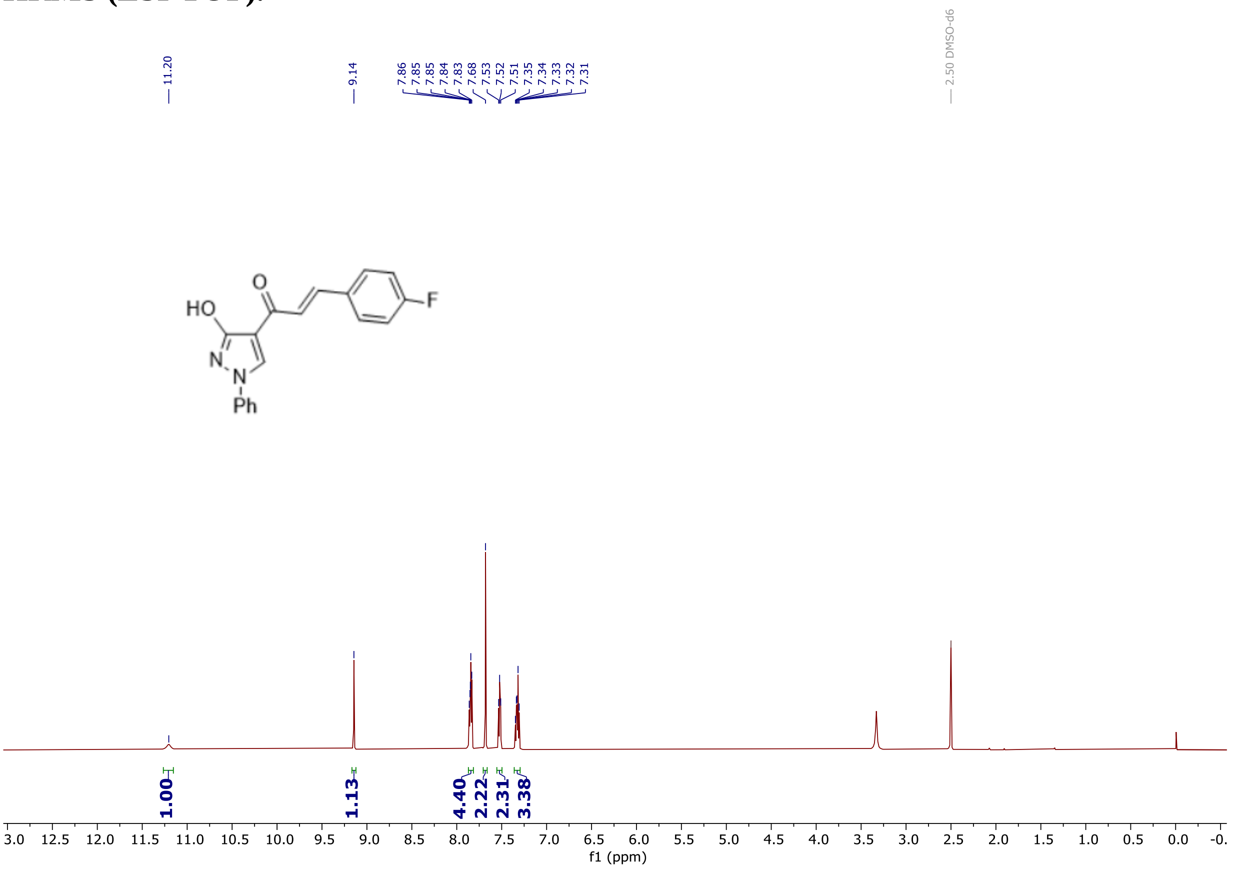

Figure S64. (2*E*)-3-(4-Fluorophenyl)-1-(3-hydroxy-1-phenyl-1*H*-pyrazol-4-yl)prop-2-en-1-one (8b). ^{1}H NMR spectrum (700 MHz, DMSO-*d*$_6$)

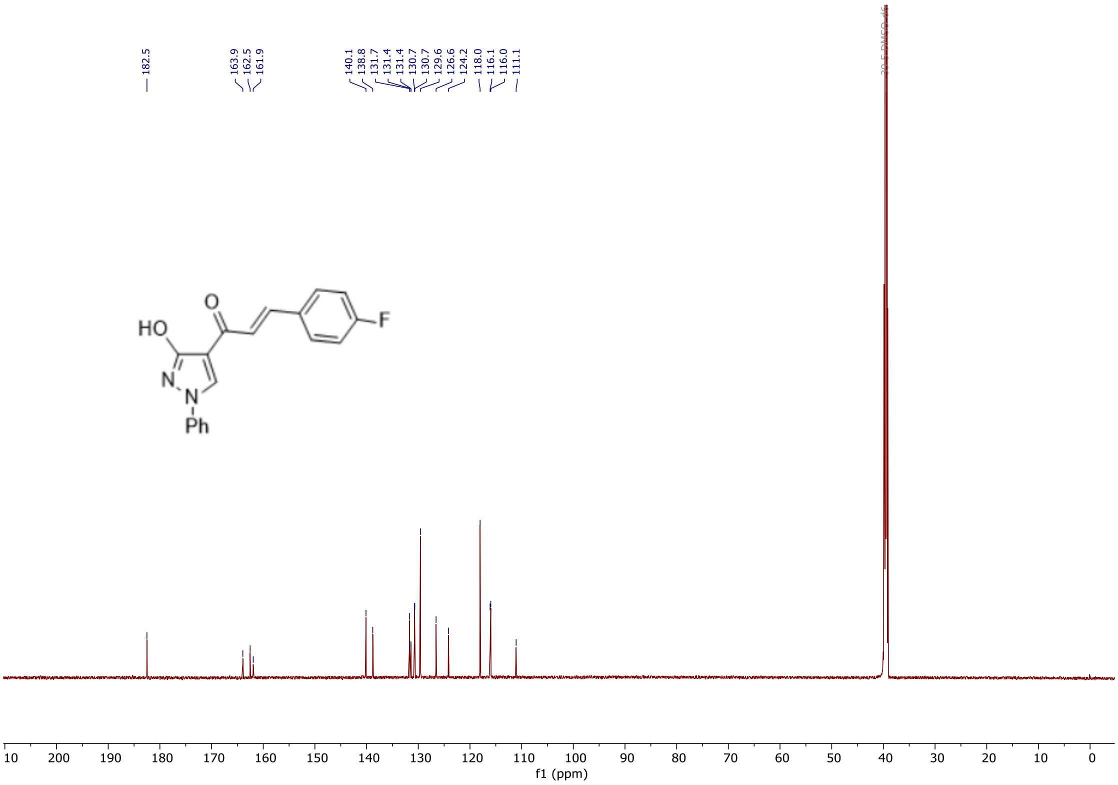

Figure S65. (2*E*)-3-(4-Fluorophenyl)-1-(3-hydroxy-1-phenyl-1*H*-pyrazol-4-yl)prop-2-en-1-one (8b). ^{13}C NMR spectrum (176 MHz, DMSO-d_6)

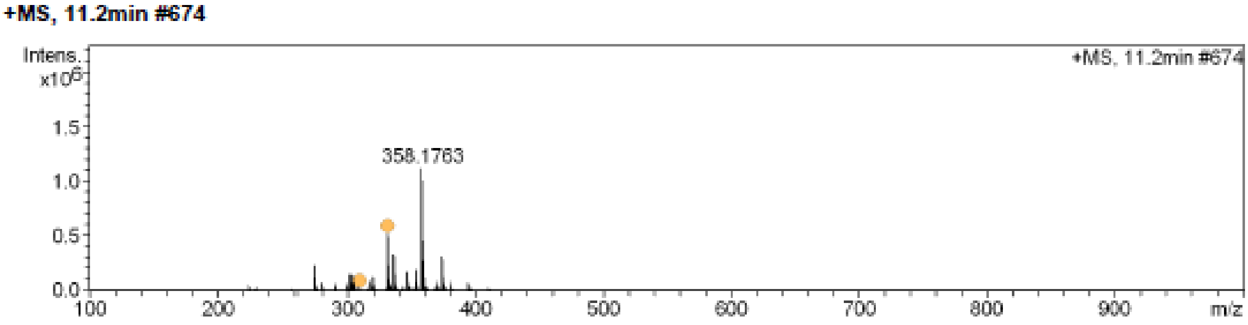

Meas. m/z	#	Ion Formula	m/z	err [ppm]	mSigma	# Sigma	Score	rdb	e⁻ Conf	N-Rule
309.1026	1	C18H14FN2O2	309.1034	-2.7	31.1	1	100.00	12.5	even	ok
331.0853	1	C18H13FN2NaO2	331.0853	0.1	10.0	1	100.00	12.5	even	ok

Figure S66. (2*E*)-3-(4-Fluorophenyl)-1-(3-hydroxy-1-phenyl-1*H*-pyrazol-4-yl)prop-2-en-1-one (8b). HRMS (ESI-TOF).

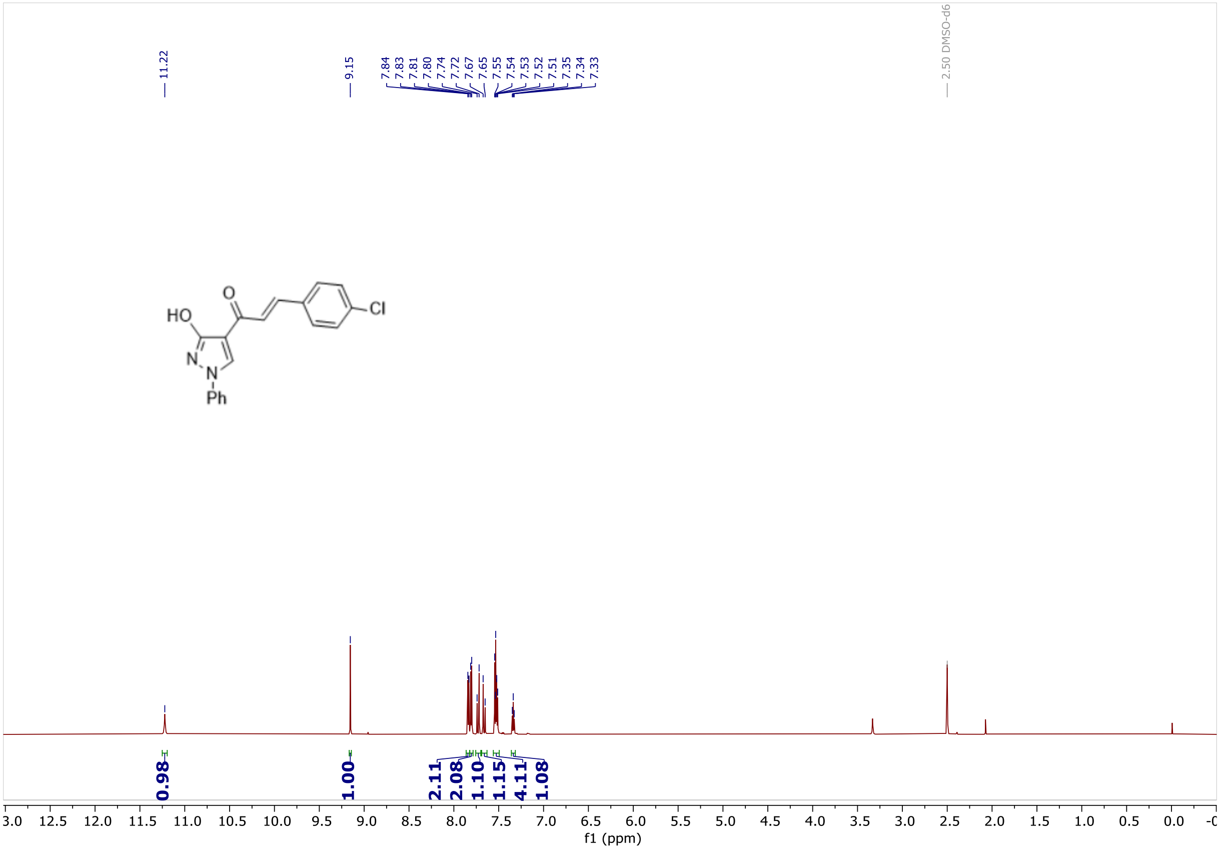

Figure S67. (2*E*)-3-(4-Chlorophenyl)-1-(3-hydroxy-1-phenyl-1*H*-pyrazol-4-yl)prop-2-en-1-one (8c). ^{1}H NMR spectrum (700 MHz, DMSO-*d*$_6$)

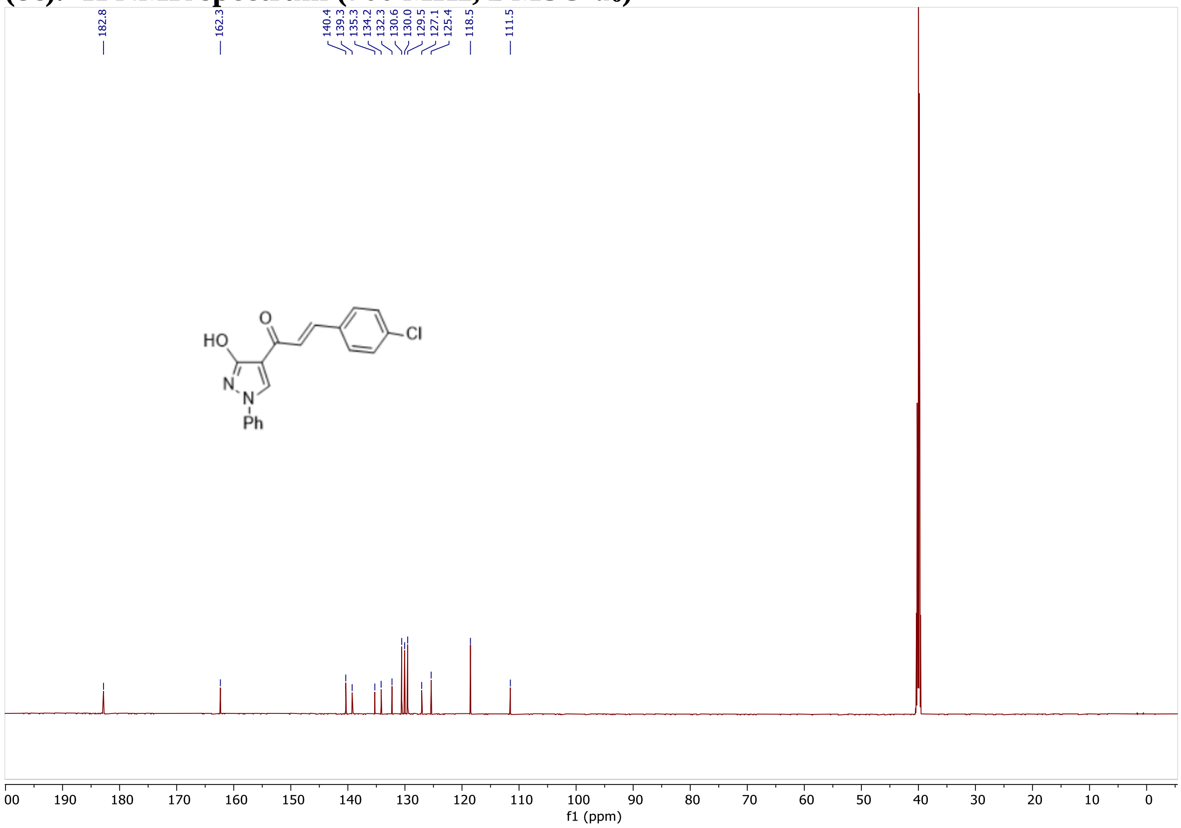

Figure S68. (2*E*)-3-(4-Chlorophenyl)-1-(3-hydroxy-1-phenyl-1*H*-pyrazol-4-yl)prop-2-en-1-one (8c). ^{13}C NMR spectrum (176 MHz, DMSO-*d*$_6$)

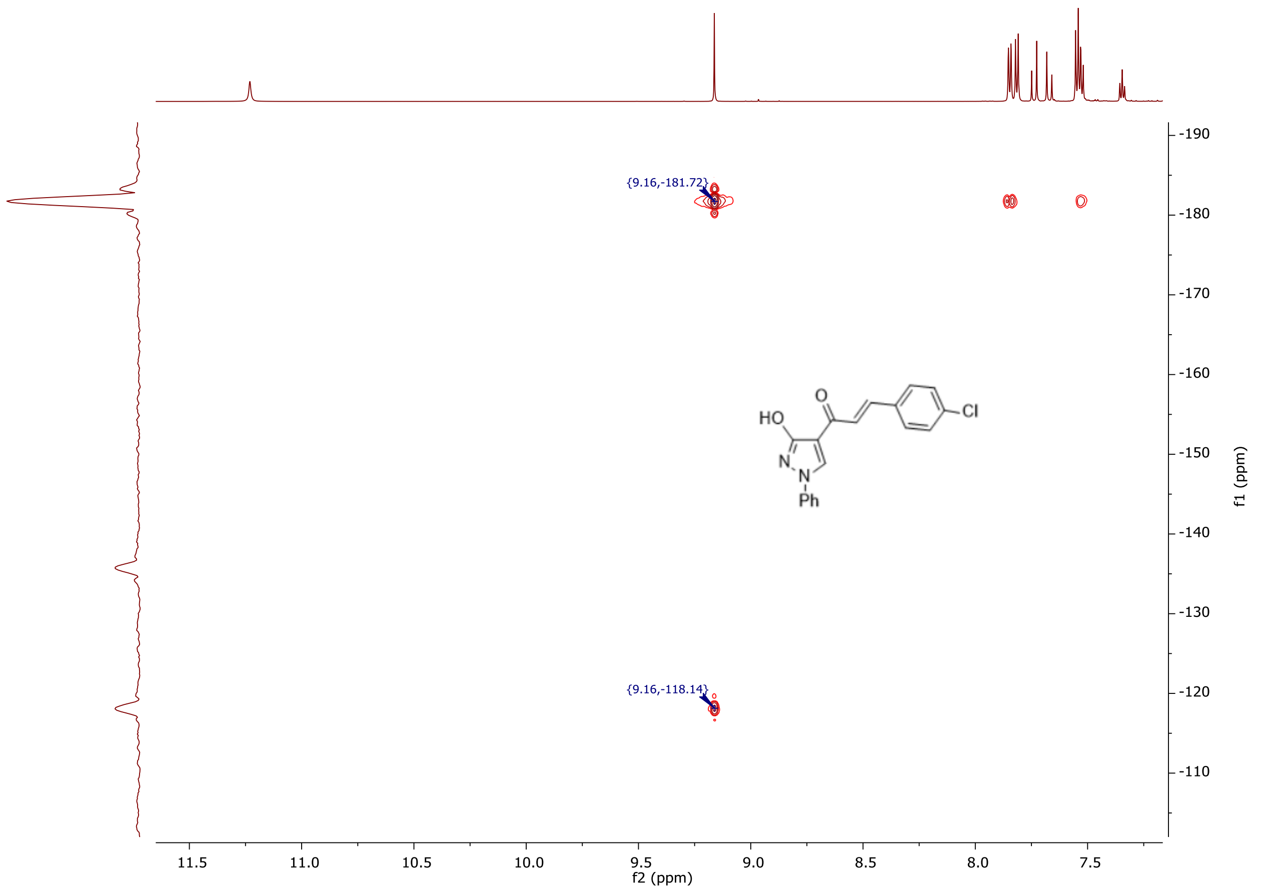

Figure S69. (2*E*)-3-(4-Chlorophenyl)-1-(3-hydroxy-1-phenyl-1*H*-pyrazol-4-yl)prop-2-en-1-one (8c). ^{1}H-^{15}N HMBC NMR spectrum (71 MHz, DMSO-d_6)

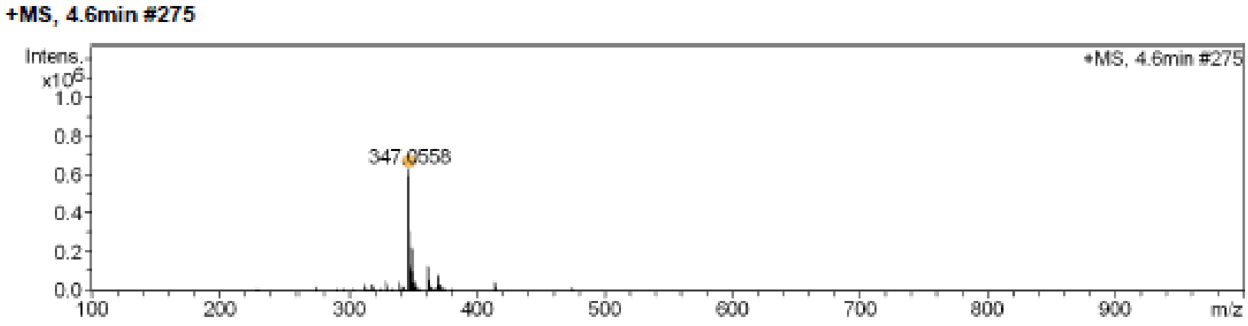

Meas. m/z	#	Ion Formula	m/z	err [ppm]	mSigma	# Sigma	Score	rdb	e¯ Conf	N-Rule
347.0558	1	C18H13ClN2NaO2	347.0558	0.0	11.9	1	100.00	12.5	even	ok

Figure S70. (2*E*)-3-(4-Chlorophenyl)-1-(3-hydroxy-1-phenyl-1*H*-pyrazol-4-yl)prop-2-en-1-one (8c). HRMS (ESI-TOF).

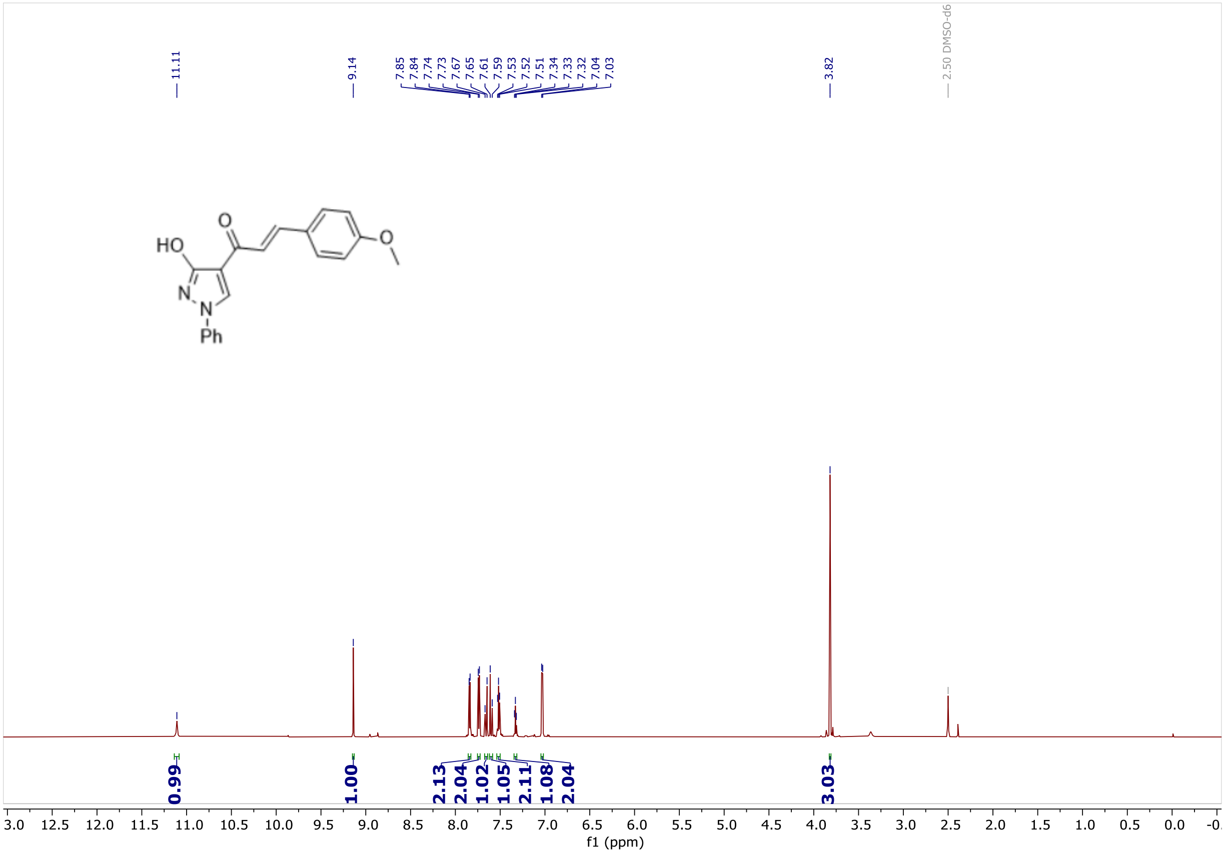

Figure S71. (2*E*)-1-(3-Hydroxy-1-phenyl-1*H*-pyrazol-4-yl)-3-(4-methoxyphenyl)prop-2-en-1-one (8d). ^{1}H NMR spectrum (700 MHz, DMSO-d_6)

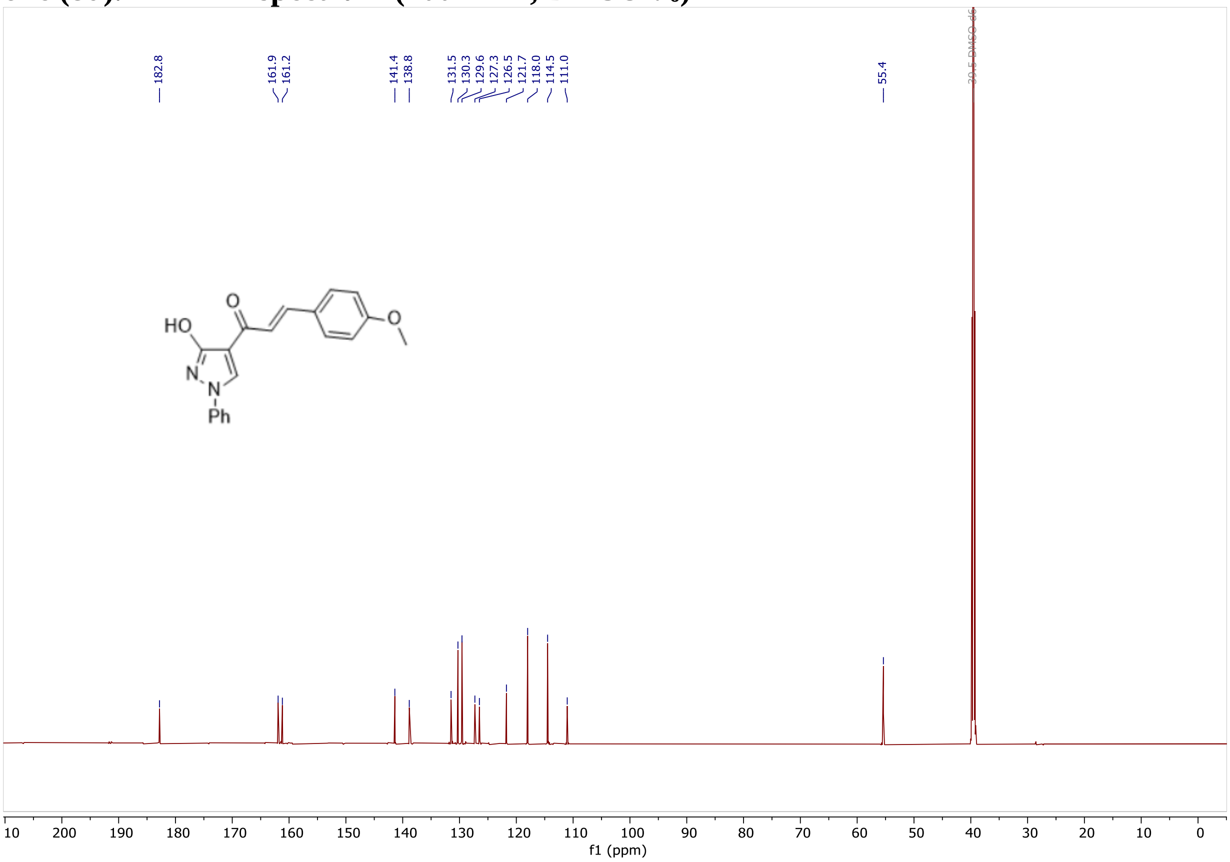

Figure S72. (2*E*)-1-(3-Hydroxy-1-phenyl-1*H*-pyrazol-4-yl)-3-(4-methoxyphenyl)prop-2-en-1-one (8d). ^{13}C NMR spectrum (176 MHz, DMSO-d_6)

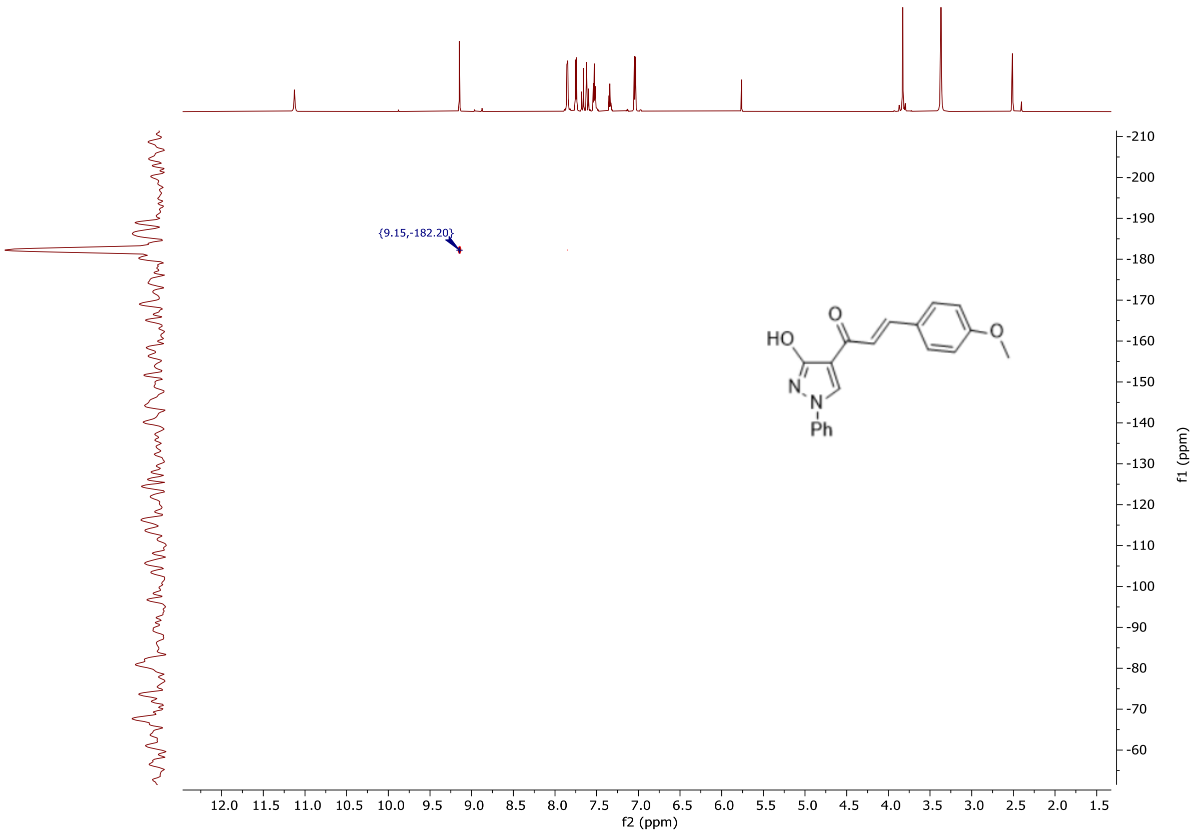

Figure S73. (2*E*)-1-(3-Hydroxy-1-phenyl-1*H*-pyrazol-4-yl)-3-(4-methoxyphenyl)prop-2-en-1-one (8d). ^{1}H-^{15}N HMBC NMR spectrum (71 MHz, DMSO-d_6)

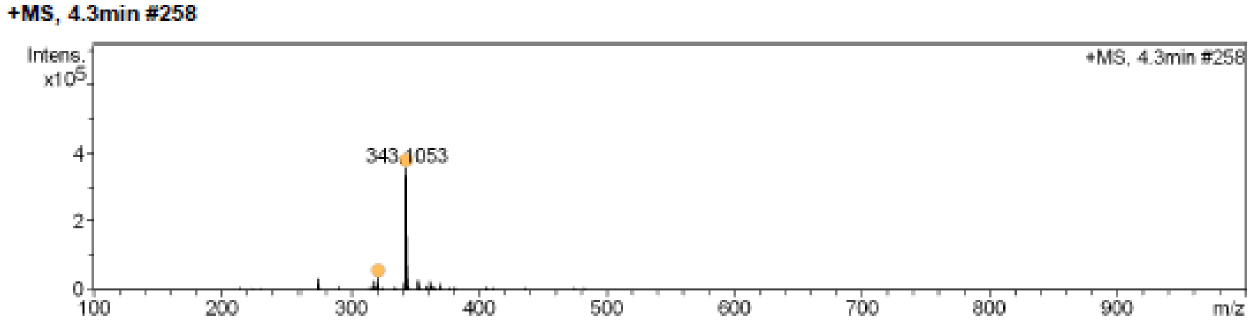

Meas. m/z	#	Ion Formula	m/z	err [ppm]	mSigma	# Sigma	Score	rdb	e¯ Conf	N-Rule
321.1234	1	C19H17N2O3	321.1234	-0.0	5.8	1	100.00	12.5	even	ok
343.1053	1	C19H16N2NaO3	343.1053	0.0	4.9	1	100.00	12.5	even	ok

Figure S74. (2*E*)-1-(3-Hydroxy-1-phenyl-1*H*-pyrazol-4-yl)-3-(4-methoxyphenyl)prop-2-en-1-one (8d). HRMS (ESI-TOF).

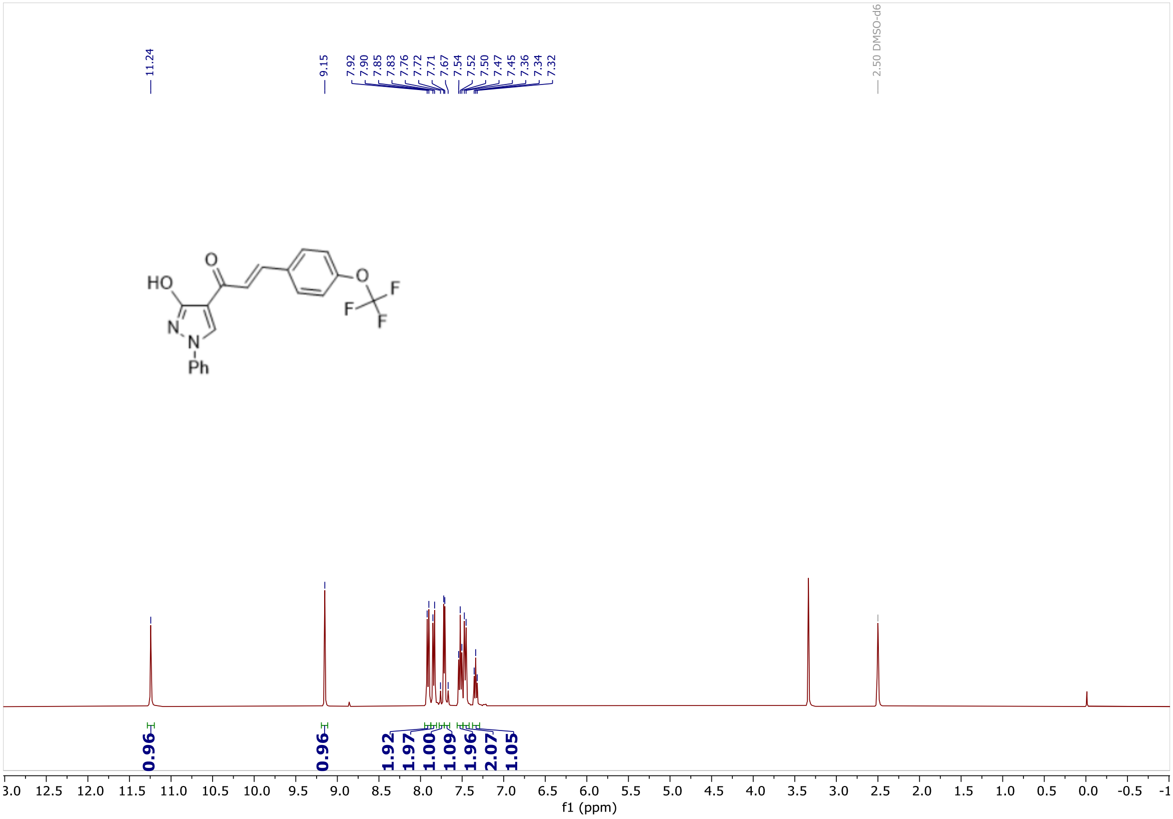

Figure S75. (2*E*)-1-(3-Hydroxy-1-phenyl-1*H*-pyrazol-4-yl)-3-[4-(trifluoromethoxy)phenyl]prop-2-en-1-one (8e). ^{1}H NMR spectrum (400 MHz, DMSO-d_6)

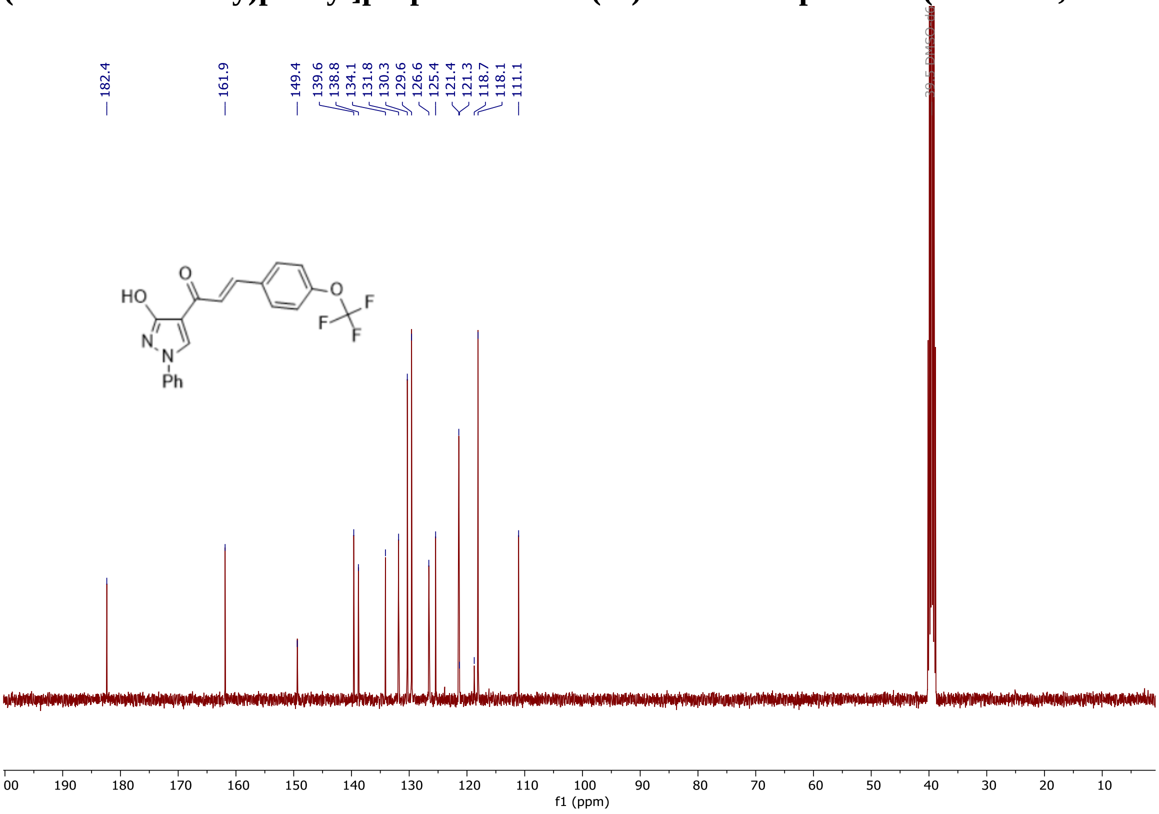

Figure S76.(2*E*)-1-(3-Hydroxy-1-phenyl-1*H*-pyrazol-4-yl)-3-[4-(trifluoromethoxy)phenyl]prop-2-en-1-one (8e). ^{13}C NMR spectrum (101 MHz, DMSO-d_6)

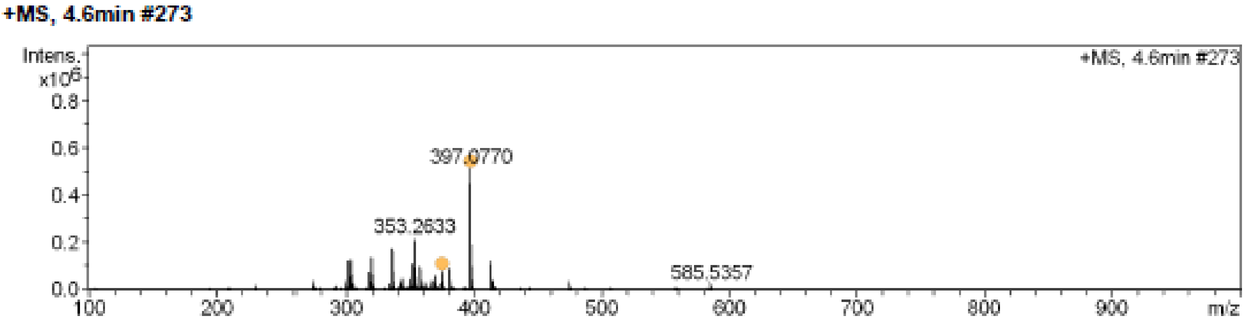

Meas. m/z	#	Ion Formula	m/z	err [ppm]	mSigma	# Sigma	Score	rdb	e⁻ Conf	N-Rule
375.0954	1	C19H14F3N2O3	375.0951	0.8	3.4	1	100.00	12.5	even	ok
397.0770	1	C19H13F3N2NaO3	397.0770	-0.2	3.3	1	100.00	12.5	even	ok

Figure S77.(2*E*)-1-(3-Hydroxy-1-phenyl-1*H*-pyrazol-4-yl)-3-[4-(trifluoromethoxy)phenyl]prop-2-en-1-one (8e). HRMS (ESI-TOF).

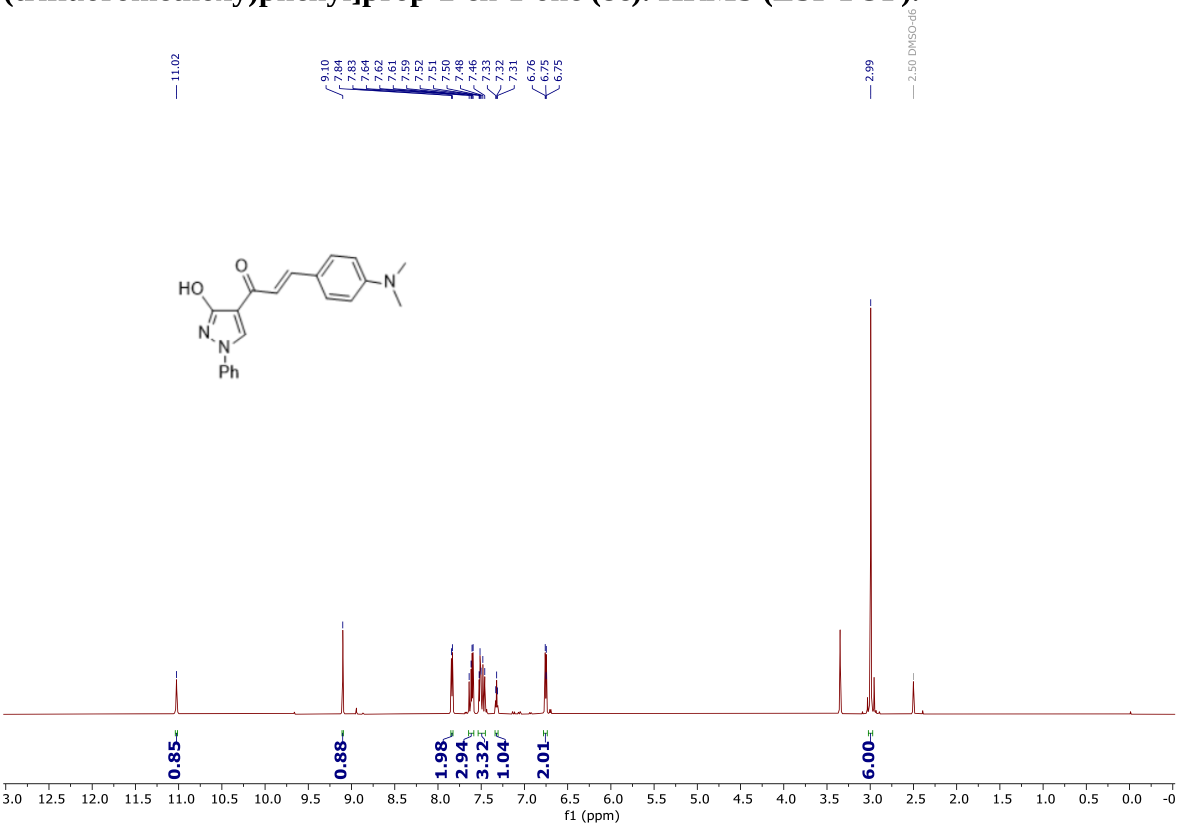

Figure S78. (2*E*)-3-[4-(Dimethylamino)phenyl]-1-(3-hydroxy-1-phenyl-1*H*-pyrazol-4-yl)prop-2-en-1-one (8f). ^{1}H NMR spectrum (700 MHz, DMSO-d_6)

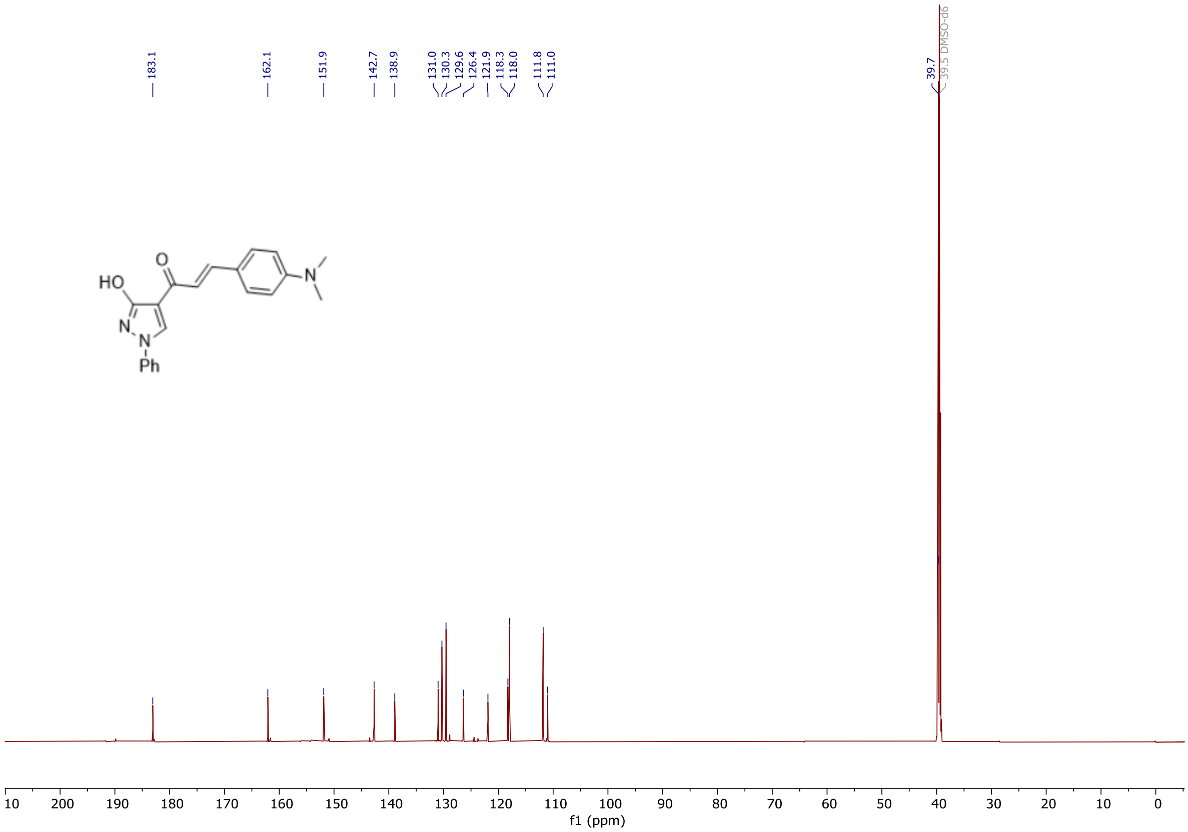

Figure S79. (2*E*)-3-[4-(Dimethylamino)phenyl]-1-(3-hydroxy-1-phenyl-1*H*-pyrazol-4-yl)prop-2-en-1-one (8f). ^{13}C NMR spectrum (176 MHz, DMSO-d_6)

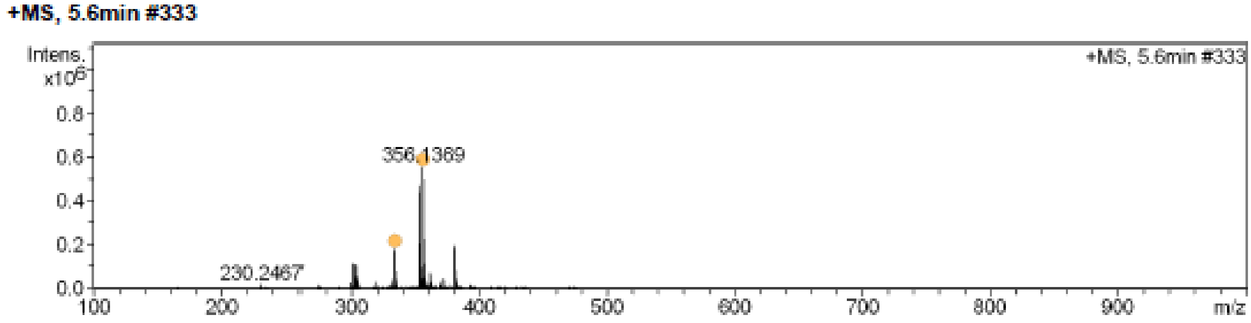

Meas. m/z	#	Ion Formula	m/z	err [ppm]	mSigma	# Sigma	Score	rdb	e⁻ Conf	N-Rule
334.1541	1	C20H20N3O2	334.1550	-2.6	14.2	1	100.00	12.5	even	ok
356.1369	1	C20H19N3NaO2	356.1369	-0.2	2.9	1	100.00	12.5	even	ok

Figure S80. (2*E*)-3-[4-(Dimethylamino)phenyl]-1-(3-hydroxy-1-phenyl-1*H*-pyrazol-4-yl)prop-2-en-1-one (8f). HRMS (ESI-TOF).

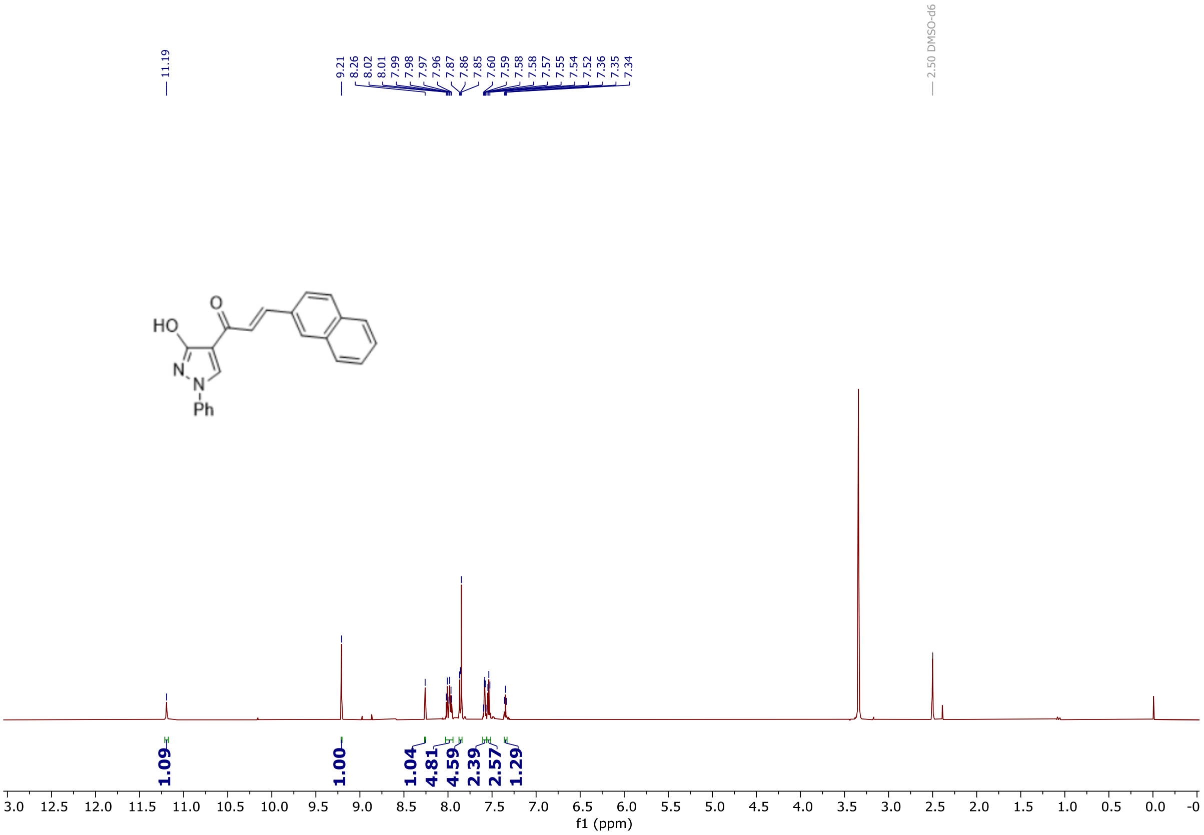

Figure S81. (2*E*)-1-(3-Hydroxy-1-phenyl-1*H*-pyrazol-4-yl)-3-(naphthalen-2-yl)prop-2-en-1-one (8g). ^{1}H NMR spectrum (700 MHz, DMSO-*d*$_6$)

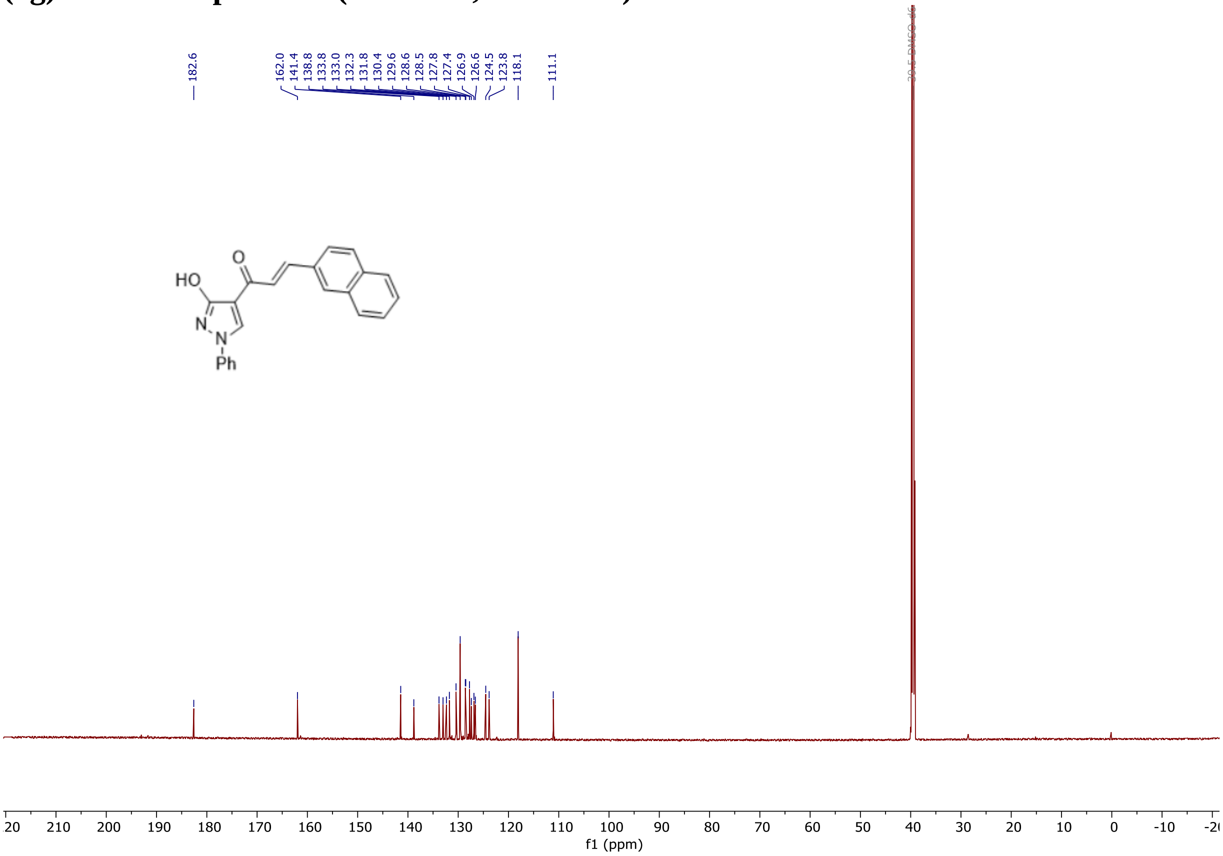

Figure S82. (2*E*)-1-(3-Hydroxy-1-phenyl-1*H*-pyrazol-4-yl)-3-(naphthalen-2-yl)prop-2-en-1-one (8g). ^{13}C NMR spectrum (176 MHz, DMSO-*d*$_6$)

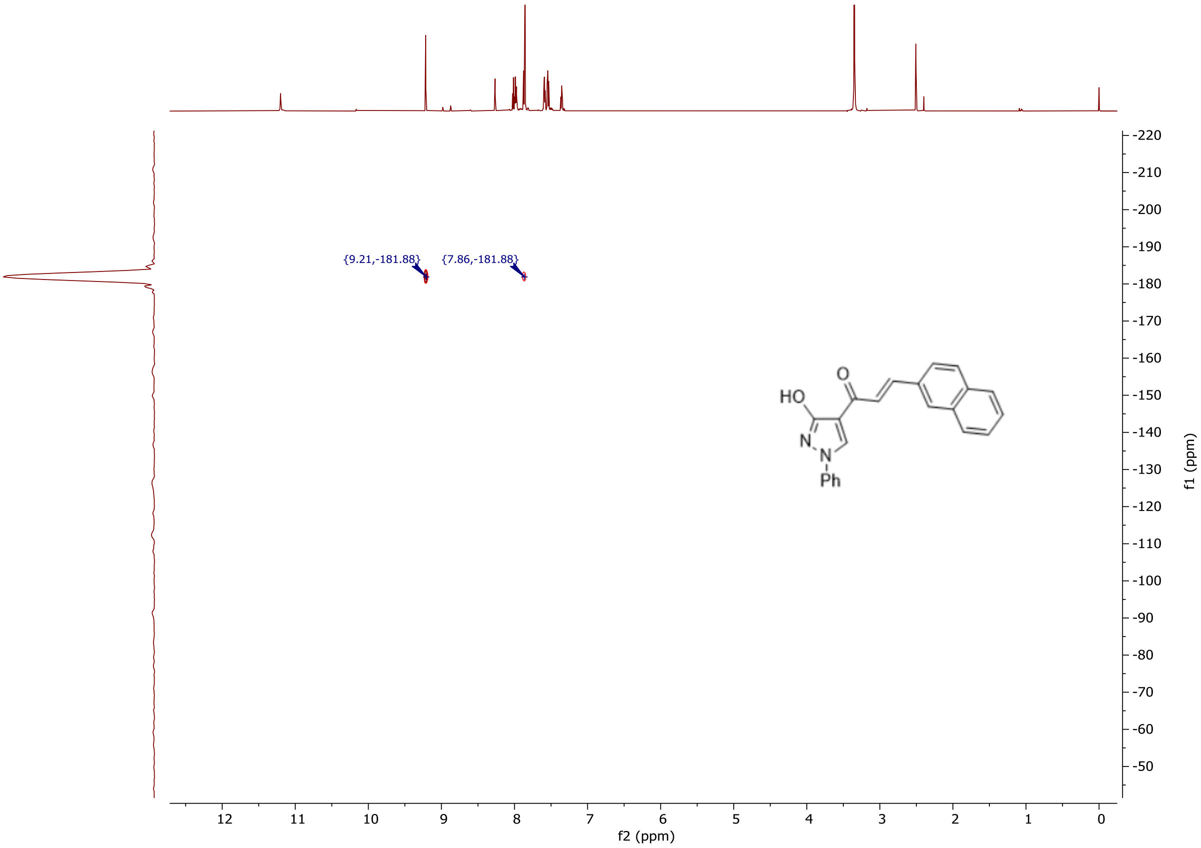

Figure S83. (2*E*)-1-(3-Hydroxy-1-phenyl-1*H*-pyrazol-4-yl)-3-(naphthalen-2-yl)prop-2-en-1-one (8g). ^{1}H-^{15}N HMBC NMR spectrum (71 MHz, DMSO-d_6)

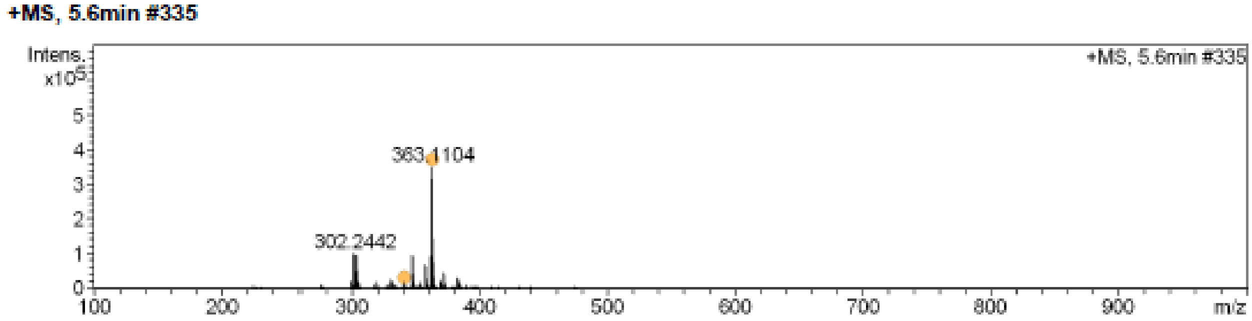

Meas. m/z	#	Ion Formula	m/z	err [ppm]	mSigma	# Sigma	Score	rdb	e⁻ Conf	N-Rule
341.1282	1	C22H17N2O2	341.1285	0.9	37.1	1	100.00	15.5	even	ok
363.1104	1	C22H16N2NaO2	363.1104	-0.1	4.6	1	100.00	15.5	even	ok

Figure S84. (2*E*)-1-(3-Hydroxy-1-phenyl-1*H*-pyrazol-4-yl)-3-(naphthalen-2-yl)prop-2-en-1-one (8g). HRMS (ESI-TOF).

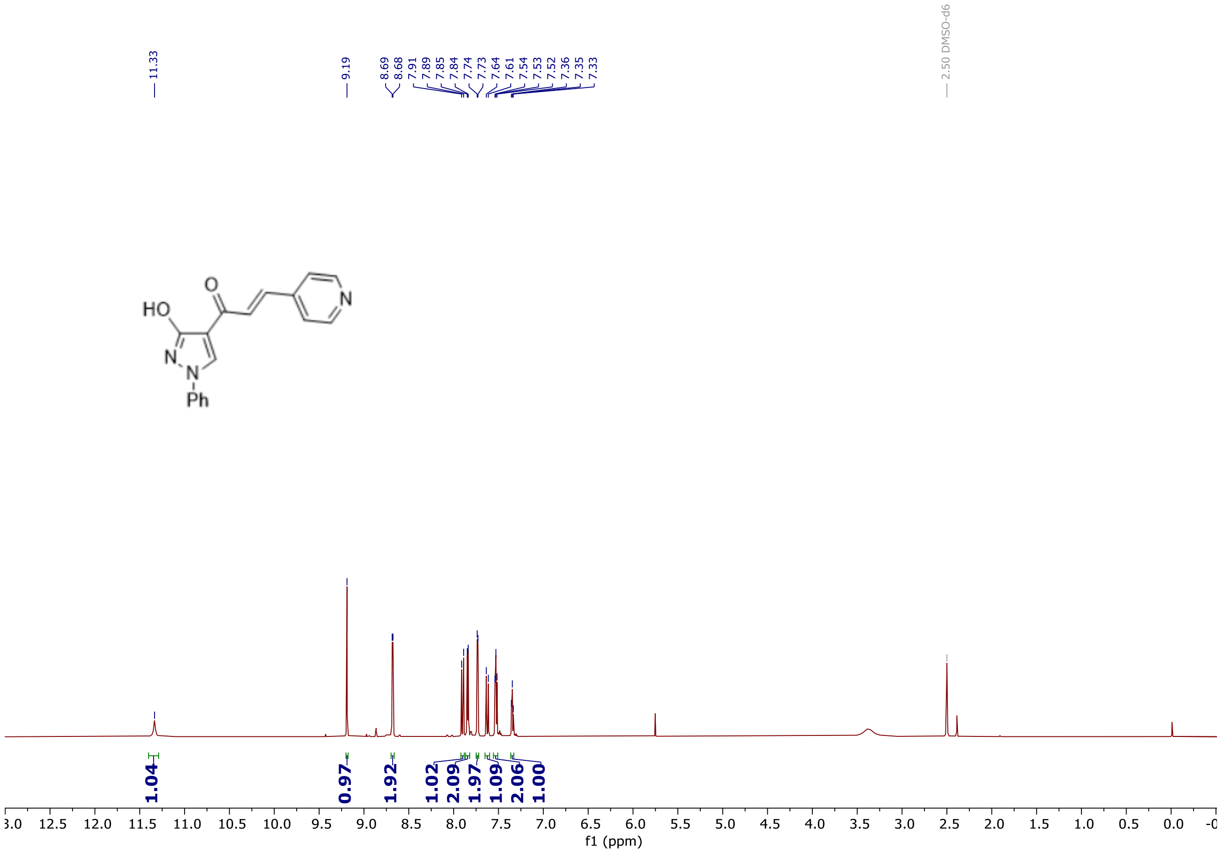

Figure S85. (2*E*)-1-(3-Hydroxy-1-phenyl-1*H*-pyrazol-4-yl)-3-(pyridin-4-yl)prop-2-en-1-one (8h). ^{1}H NMR spectrum (700 MHz, DMSO-d_6)

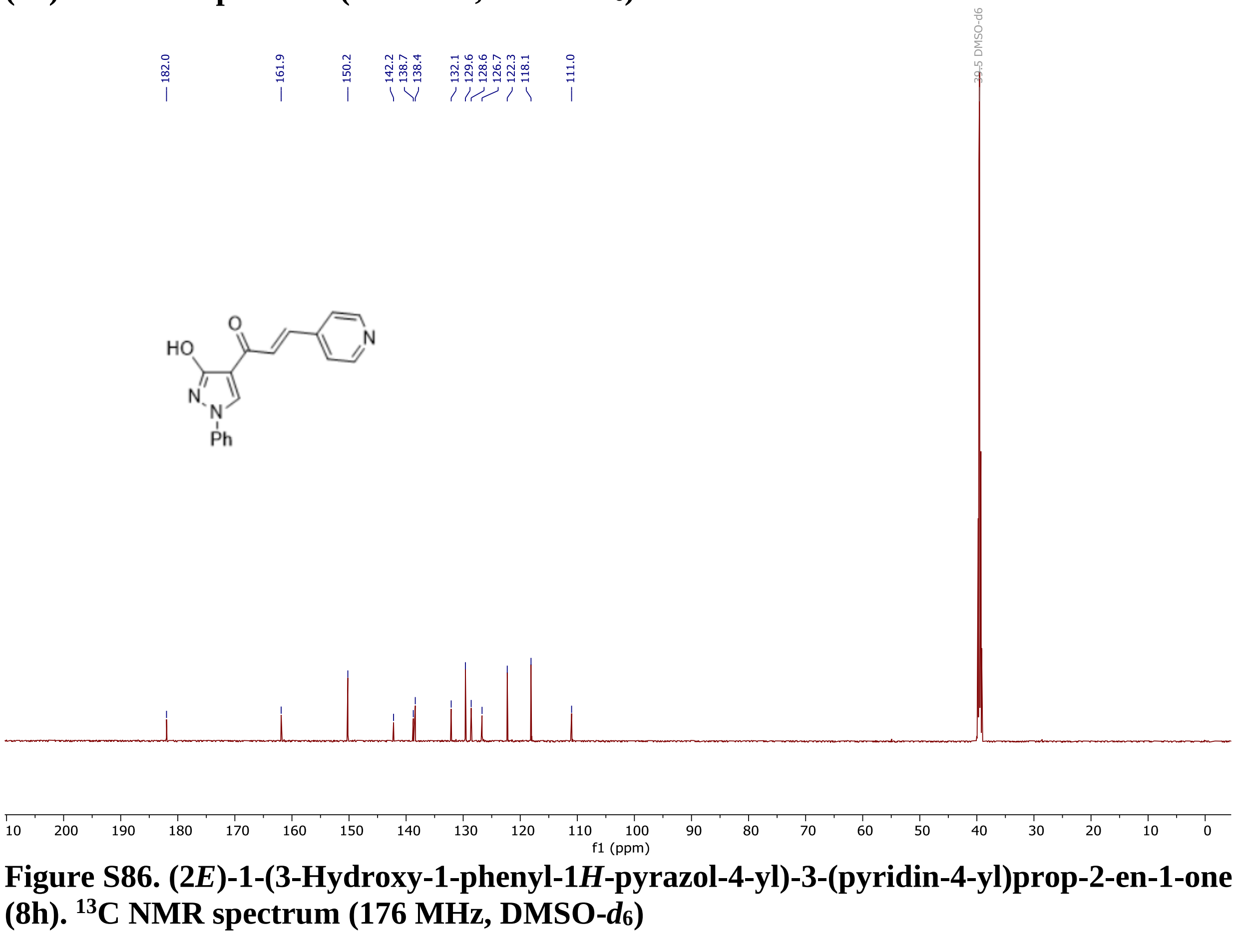

Figure S86. (2*E*)-1-(3-Hydroxy-1-phenyl-1*H*-pyrazol-4-yl)-3-(pyridin-4-yl)prop-2-en-1-one (8h). ^{13}C NMR spectrum (176 MHz, DMSO-d_6)

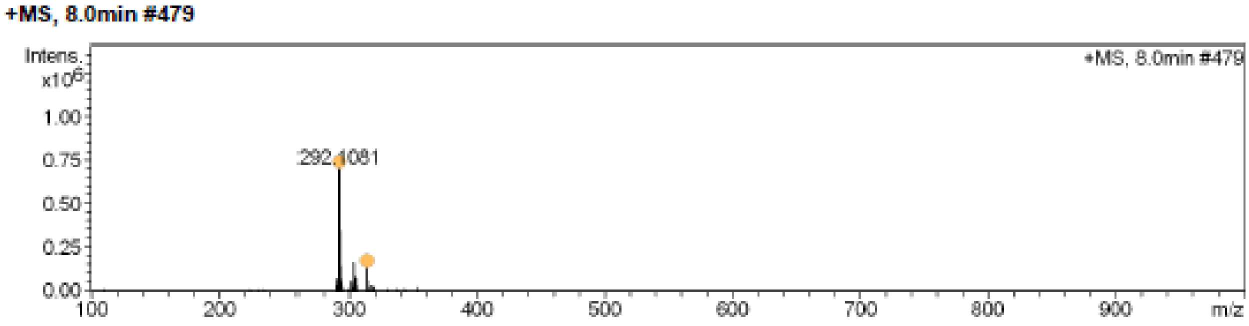

Meas. m/z	#	Ion Formula	m/z	err [ppm]	mSigma	# Sigma	Score	rdb	e⁻ Conf	N-Rule
292.1081	1	C17H14N3O2	292.1081	-0.2	34.7	1	100.00	12.5	even	ok
314.0890	1	C17H13N3NaO2	314.0900	-3.3	3.0	1	100.00	12.5	even	ok

Figure S87. (2*E*)-1-(3-Hydroxy-1-phenyl-1*H*-pyrazol-4-yl)-3-(pyridin-4-yl)prop-2-en-1-one (8h). HRMS (ESI-TOF).

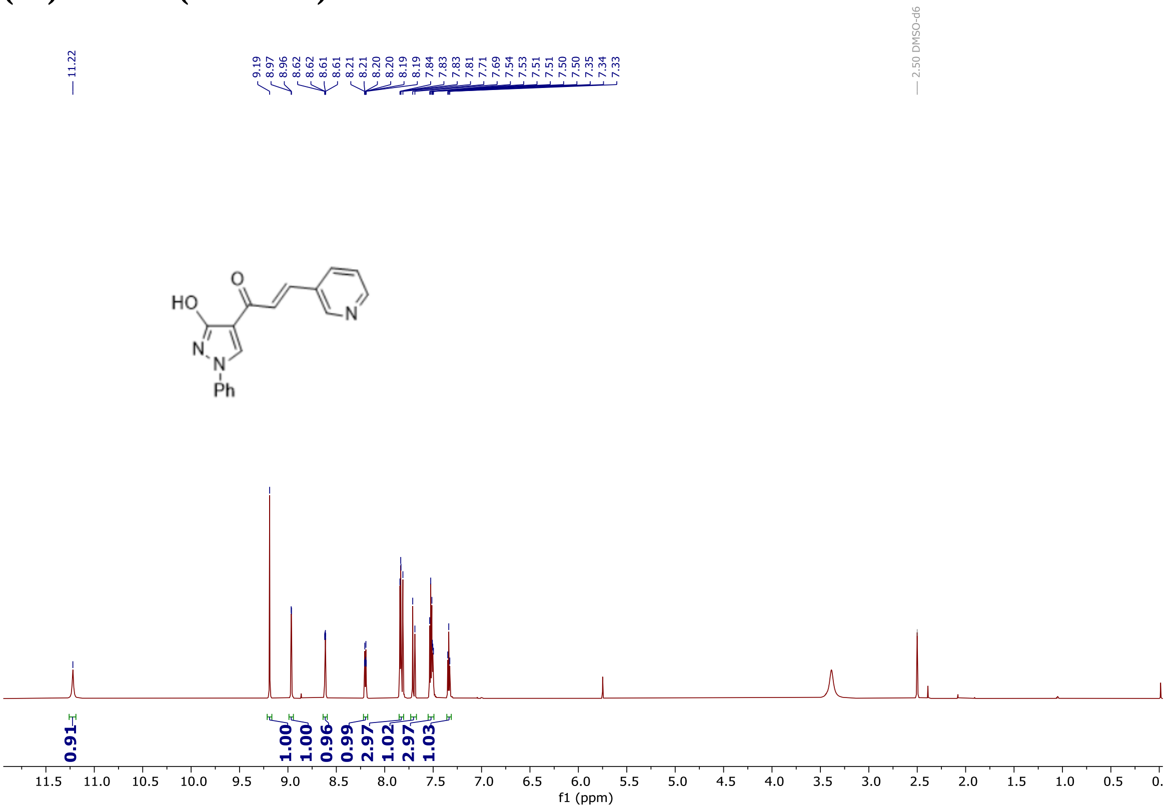

Figure S88. (2*E*)-1-(3-Hydroxy-1-phenyl-1*H*-pyrazol-4-yl)-3-(pyridin-3-yl)prop-2-en-1-one (8i). ^{1}H NMR spectrum (700 MHz, DMSO-d_6)

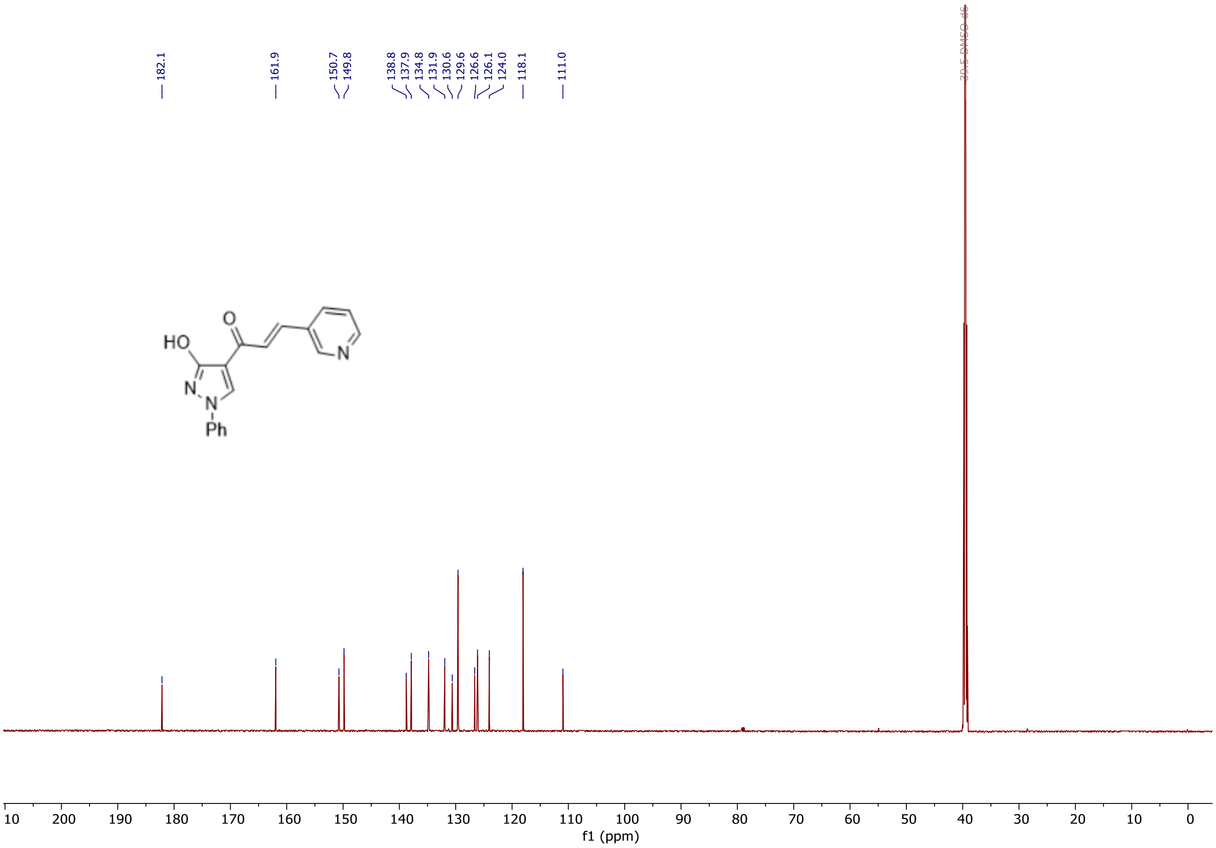

Figure S89. (2*E*)-1-(3-Hydroxy-1-phenyl-1*H*-pyrazol-4-yl)-3-(pyridin-3-yl)prop-2-en-1-one (8i). ^{13}C NMR spectrum (176 MHz, DMSO-d_6)

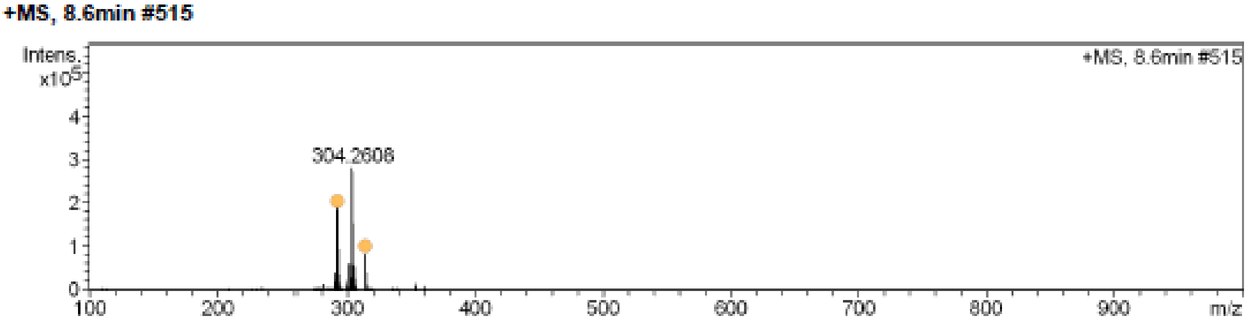

Meas. m/z	#	Ion Formula	m/z	err [ppm]	mSigma	# Sigma	Score	rdb	e⁻ Conf	N-Rule
292.1080	1	C17H14N3O2	292.1081	-0.3	3.0	1	100.00	12.5	even	ok
314.0895	1	C17H13N3NaO2	314.0900	1.6	7.1	1	100.00	12.5	even	ok

Figure S90. (2*E*)-1-(3-Hydroxy-1-phenyl-1*H*-pyrazol-4-yl)-3-(pyridin-3-yl)prop-2-en-1-one (8i). HRMS (ESI-TOF).

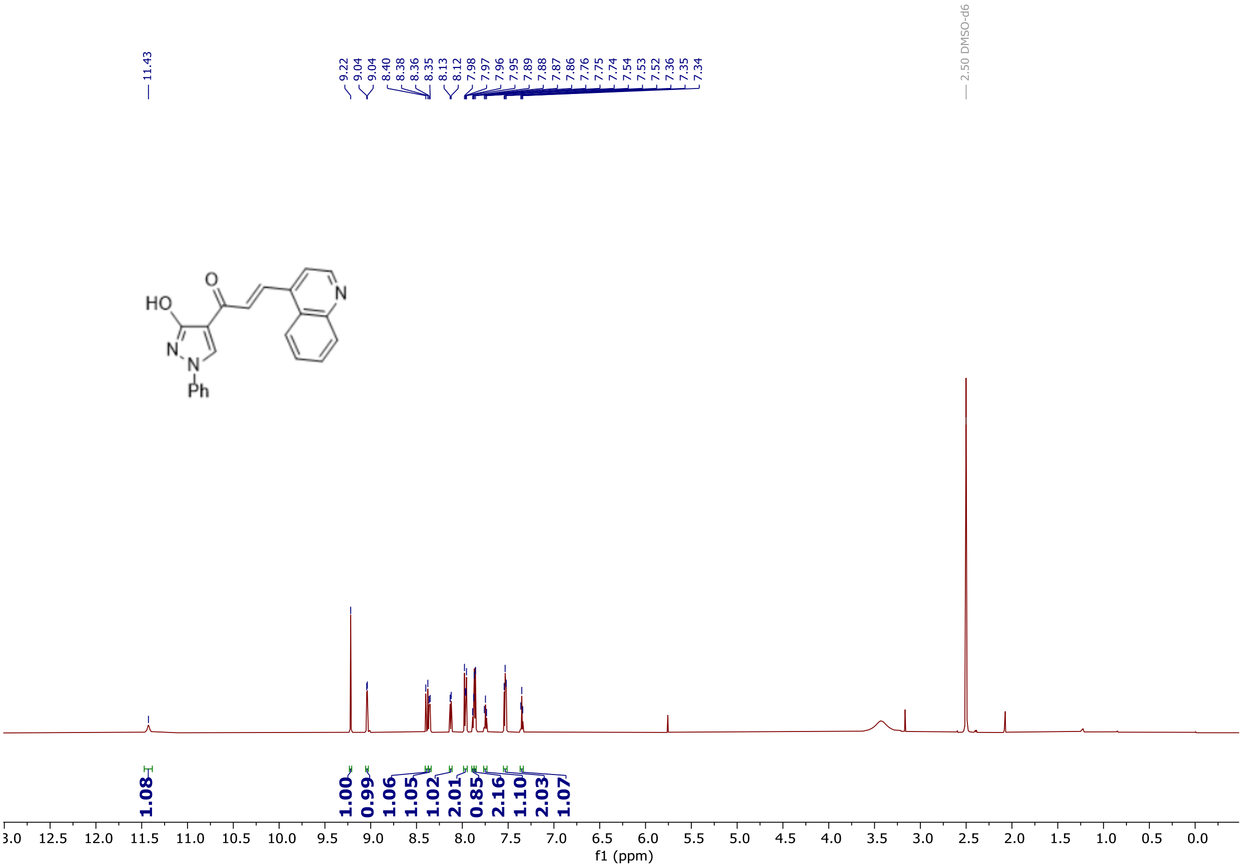

Figure S91. (2*E*)-1-(3-Hydroxy-1-phenyl-1*H*-pyrazol-4-yl)-3-(quinolin-4-yl)prop-2-en-1-one (8j). ^{1}H NMR spectrum (700 MHz, DMSO-*d*$_6$)

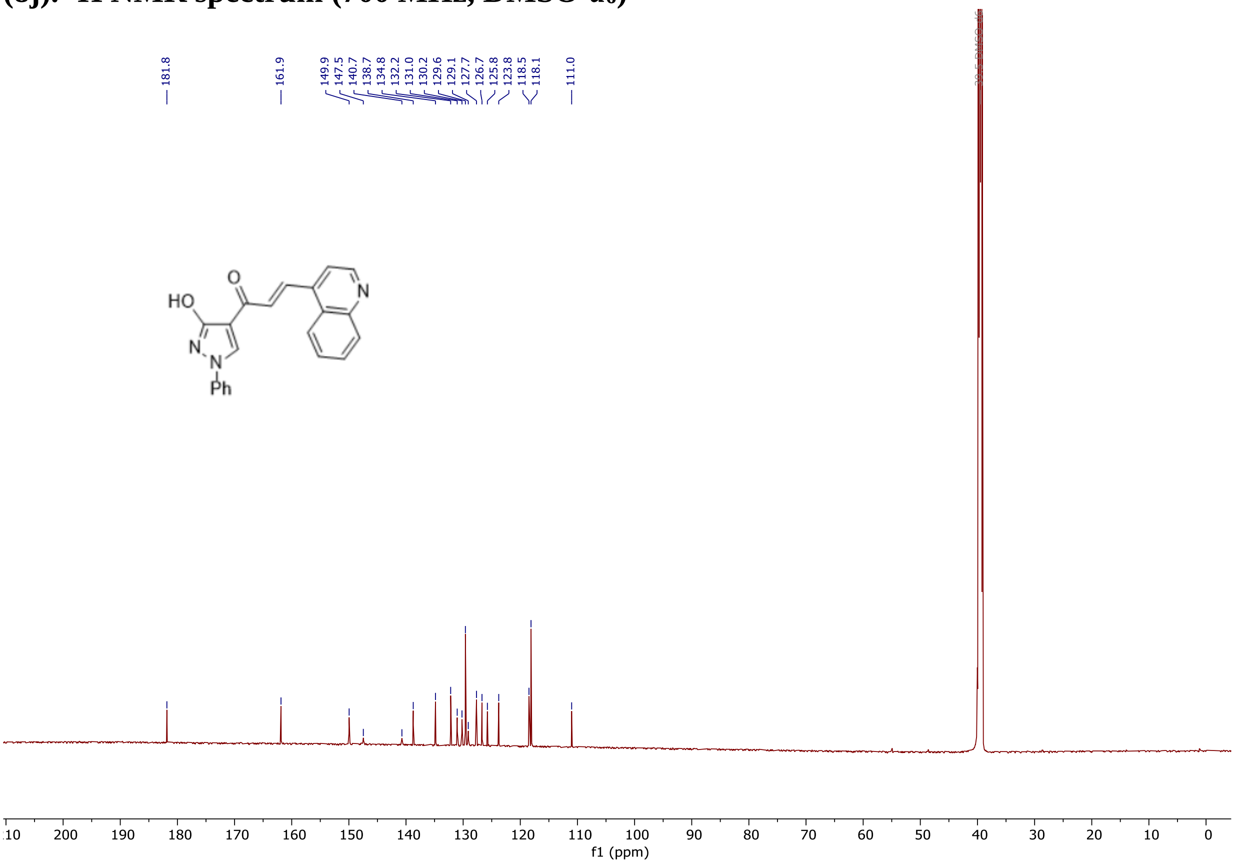

Figure S92. (2*E*)-1-(3-Hydroxy-1-phenyl-1*H*-pyrazol-4-yl)-3-(quinolin-4-yl)prop-2-en-1-one (8j). ^{13}C NMR spectrum (176 MHz, DMSO-*d*$_6$)

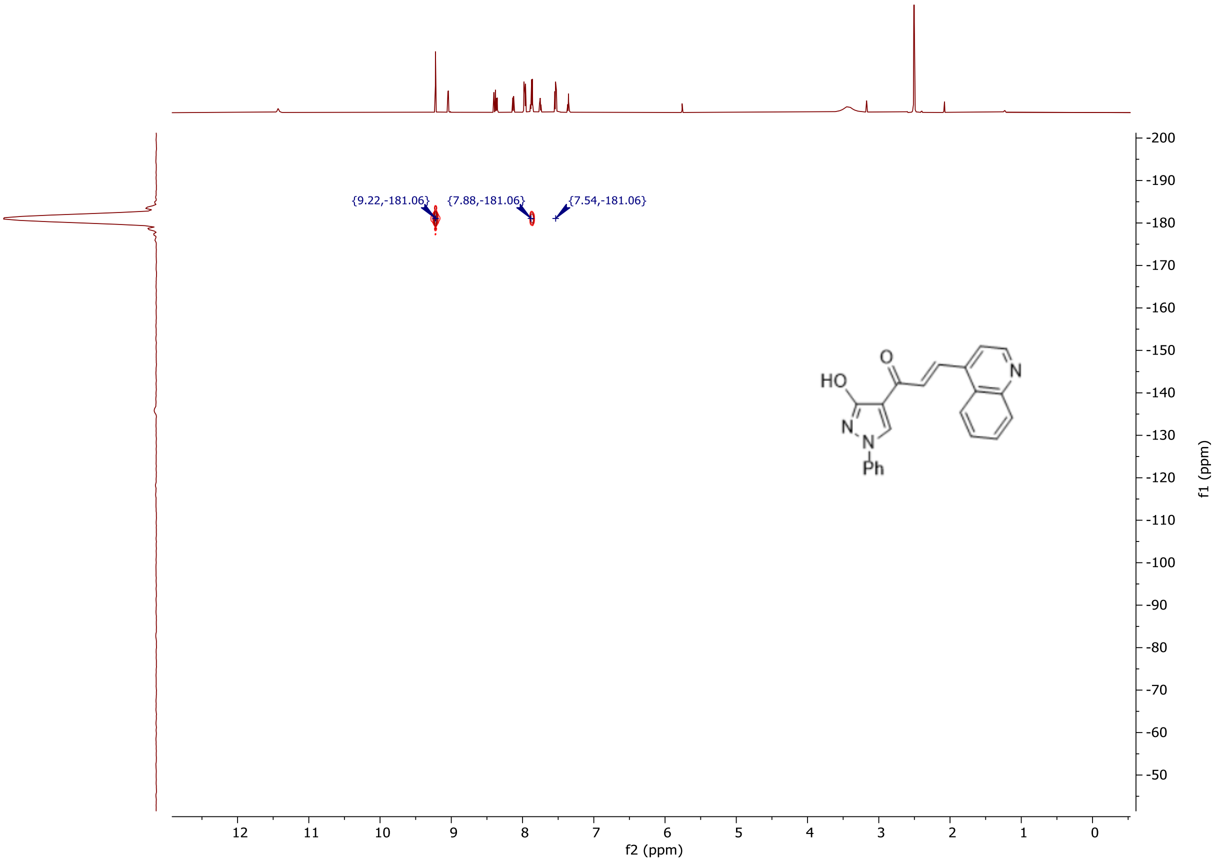

Figure S93. (2*E*)-1-(3-Hydroxy-1-phenyl-1*H*-pyrazol-4-yl)-3-(quinolin-4-yl)prop-2-en-1-one (8j). ^{1}H-^{15}N HMBC NMR spectrum (71 MHz, DMSO-d_6)

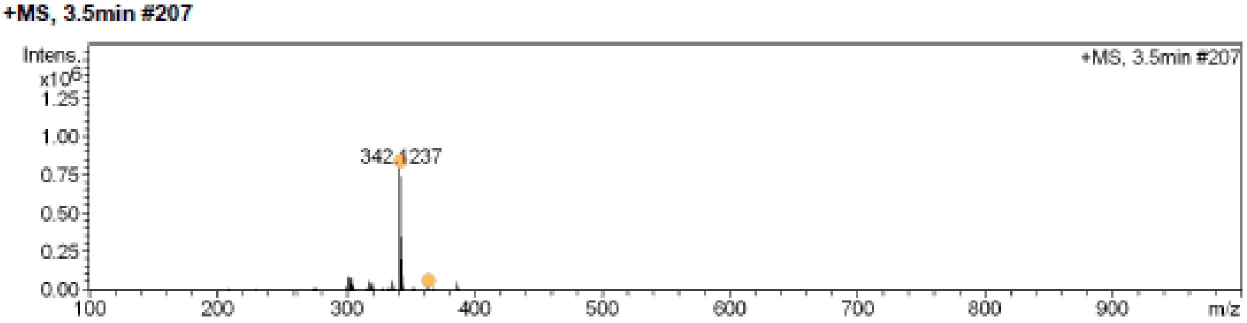

Meas. m/z	#	Ion Formula	m/z	err [ppm]	mSigma	# Sigma	Score	rdb	e⁻ Conf	N-Rule
342.1237	1	C21H16N3O2	342.1237	-0.1	18.1	1	100.00	15.5	even	ok
364.1080	1	C21H15N3NaO2	364.1056	-6.6	53.0	1	100.00	15.5	even	ok

Figure S94. (2*E*)-1-(3-Hydroxy-1-phenyl-1*H*-pyrazol-4-yl)-3-(quinolin-4-yl)prop-2-en-1-one (8j). HRMS (ESI-TOF).

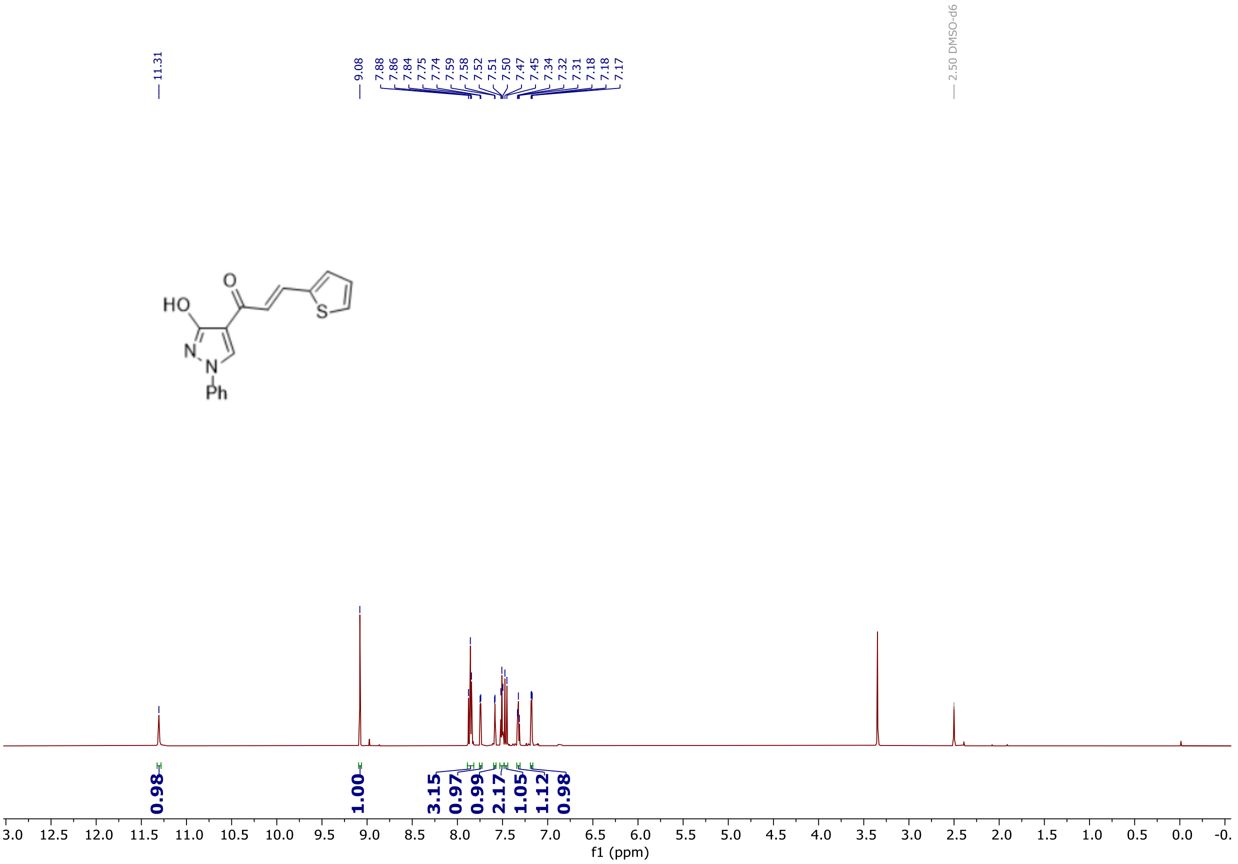

Figure S95. (2*E*)-1-(3-Hydroxy-1-phenyl-1*H*-pyrazol-4-yl)-3-(thiophen-2-yl)prop-2-en-1-one (8k). ^{1}H NMR spectrum (700 MHz, DMSO-d_6)

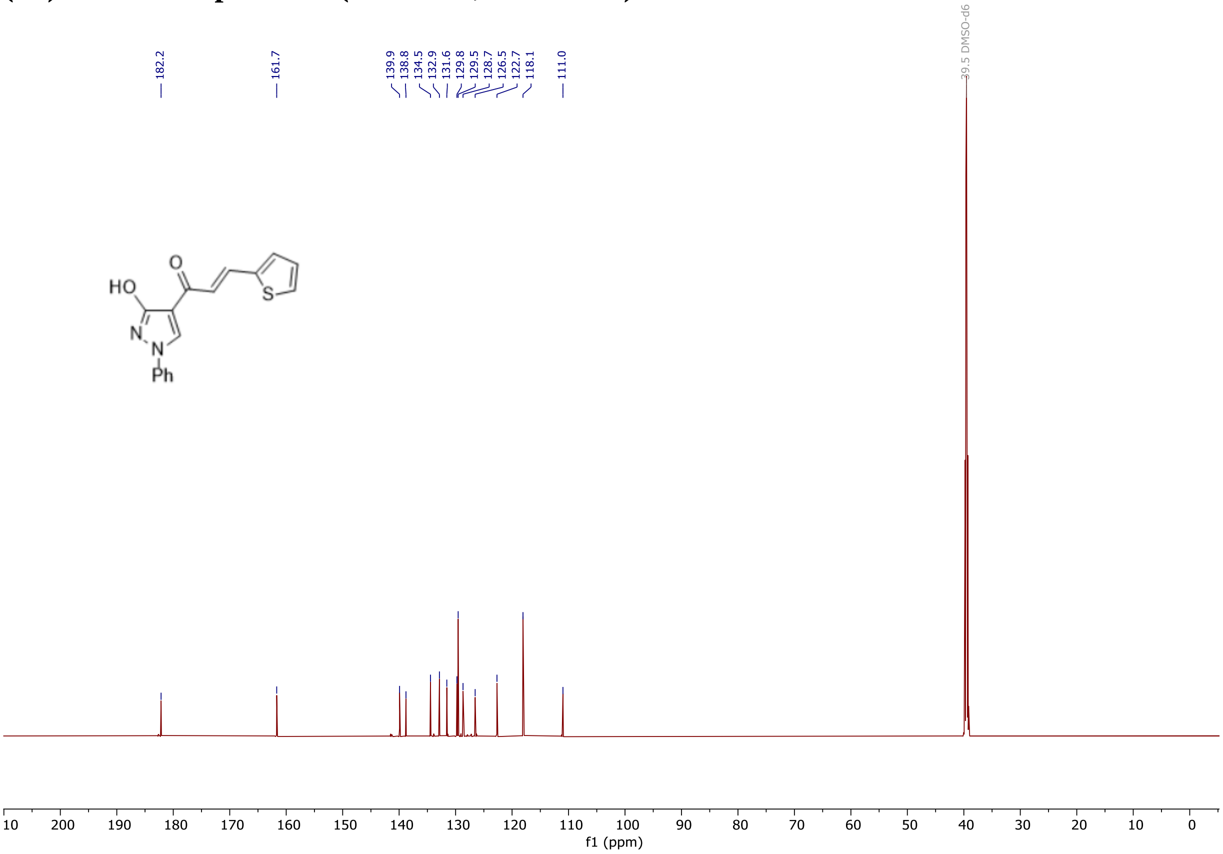

Figure S96. (2*E*)-1-(3-Hydroxy-1-phenyl-1*H*-pyrazol-4-yl)-3-(thiophen-2-yl)prop-2-en-1-one (8k). ^{13}C NMR spectrum (176 MHz, DMSO-d_6)

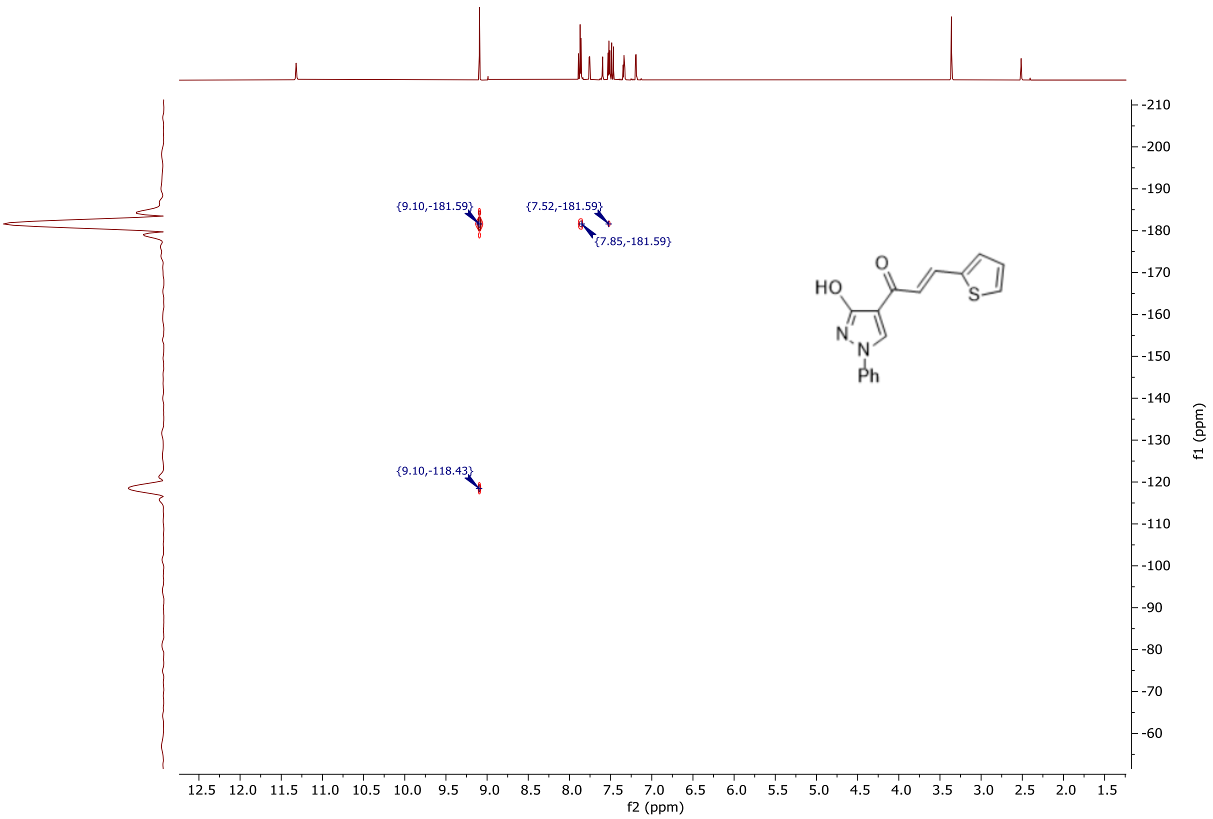

Figure S97. (2*E*)-1-(3-Hydroxy-1-phenyl-1*H*-pyrazol-4-yl)-3-(thiophen-2-yl)prop-2-en-1-one (8k). ^{1}H-^{15}N HMBC NMR spectrum (71 MHz, DMSO-d_6)

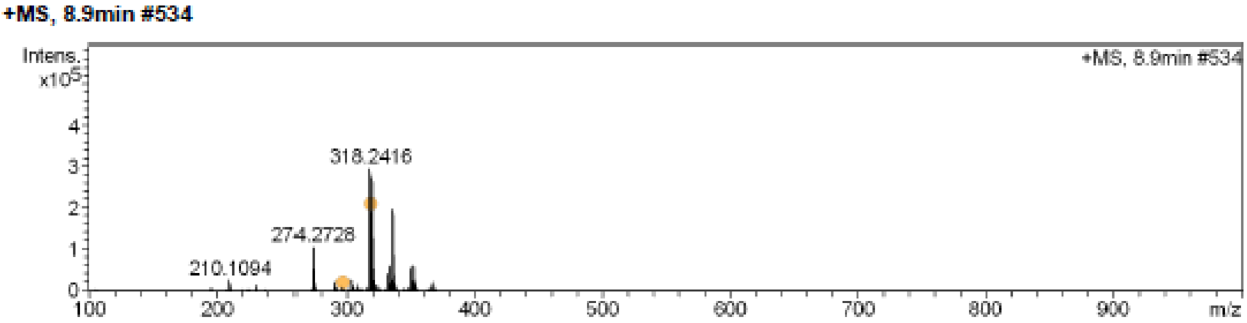

Meas. m/z	#	Ion Formula	m/z	err [ppm]	mSigma	# Sigma	Score	rdb	e¯ Conf	N-Rule
297.0683	1	C16H13N2O2S	297.0692	-3.0	34.6	1	100.00	11.5	even	ok
319.0512	1	C16H12N2NaO2S	319.0512	-0.2	6.8	1	100.00	11.5	even	ok

Figure S98. (2*E*)-1-(3-Hydroxy-1-phenyl-1*H*-pyrazol-4-yl)-3-(thiophen-2-yl)prop-2-en-1-one (8k). HRMS (ESI-TOF).

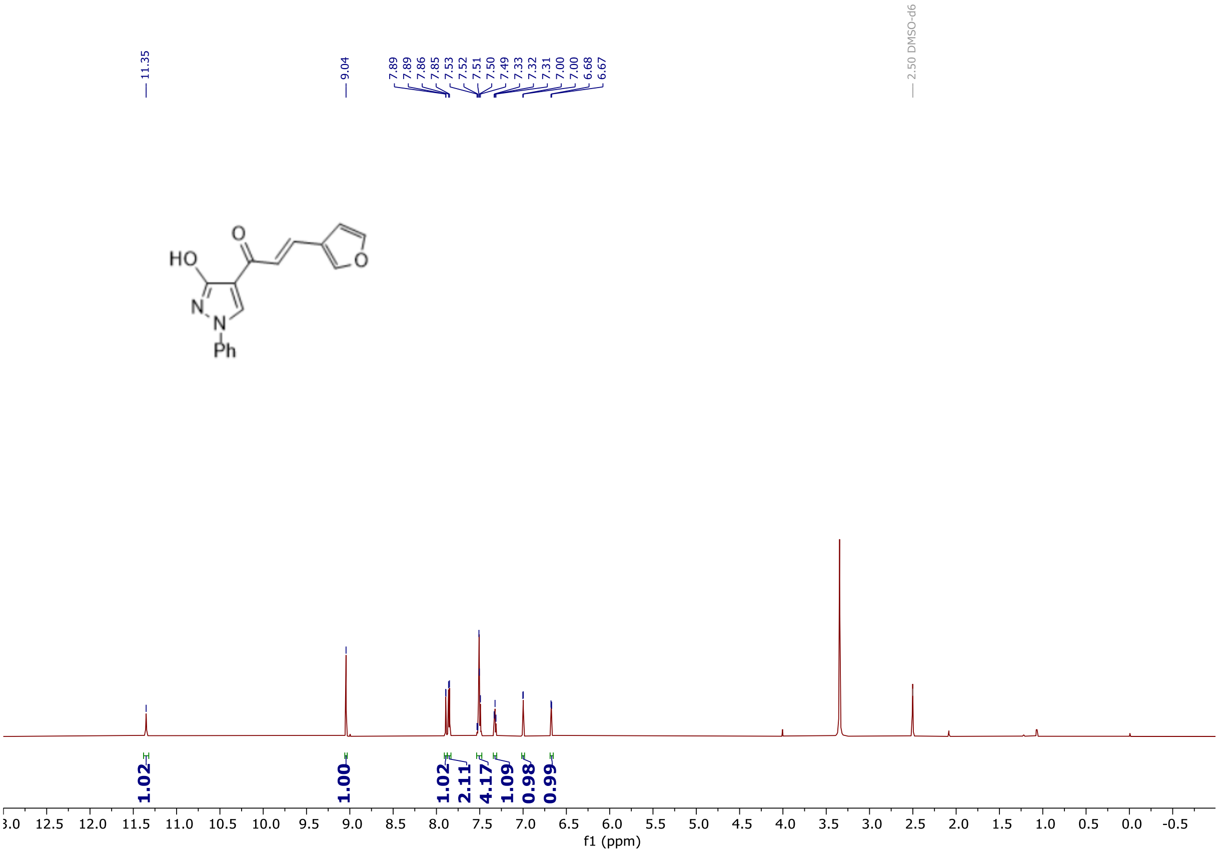

Figure S99. (2*E*)-3-(Furan-3-yl)-1-(3-hydroxy-1-phenyl-1*H*-pyrazol-4-yl)prop-2-en-1-one (8l). ^{1}H NMR spectrum (700 MHz, DMSO-*d*$_6$)

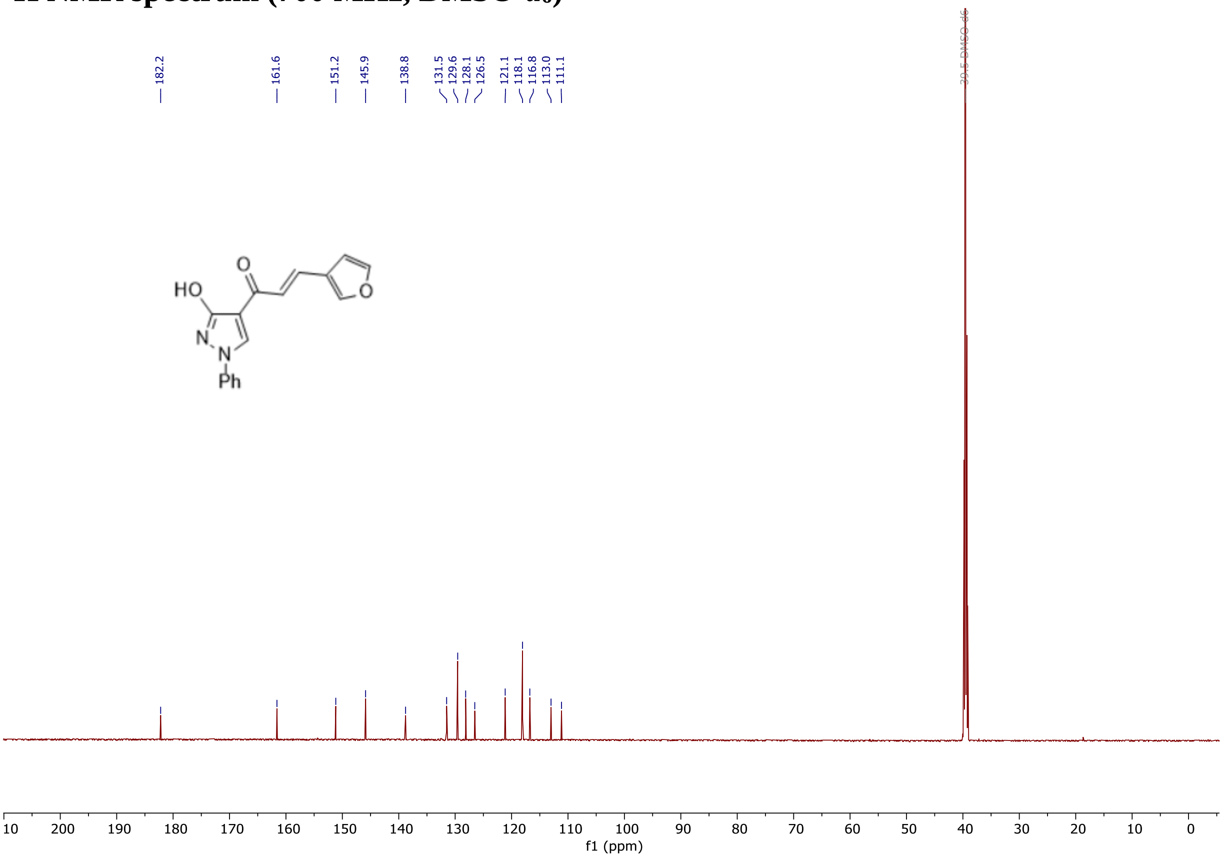

Figure S100. (2*E*)-3-(Furan-3-yl)-1-(3-hydroxy-1-phenyl-1*H*-pyrazol-4-yl)prop-2-en-1-one (8l). ^{13}C NMR spectrum (176 MHz, DMSO-*d*$_6$)

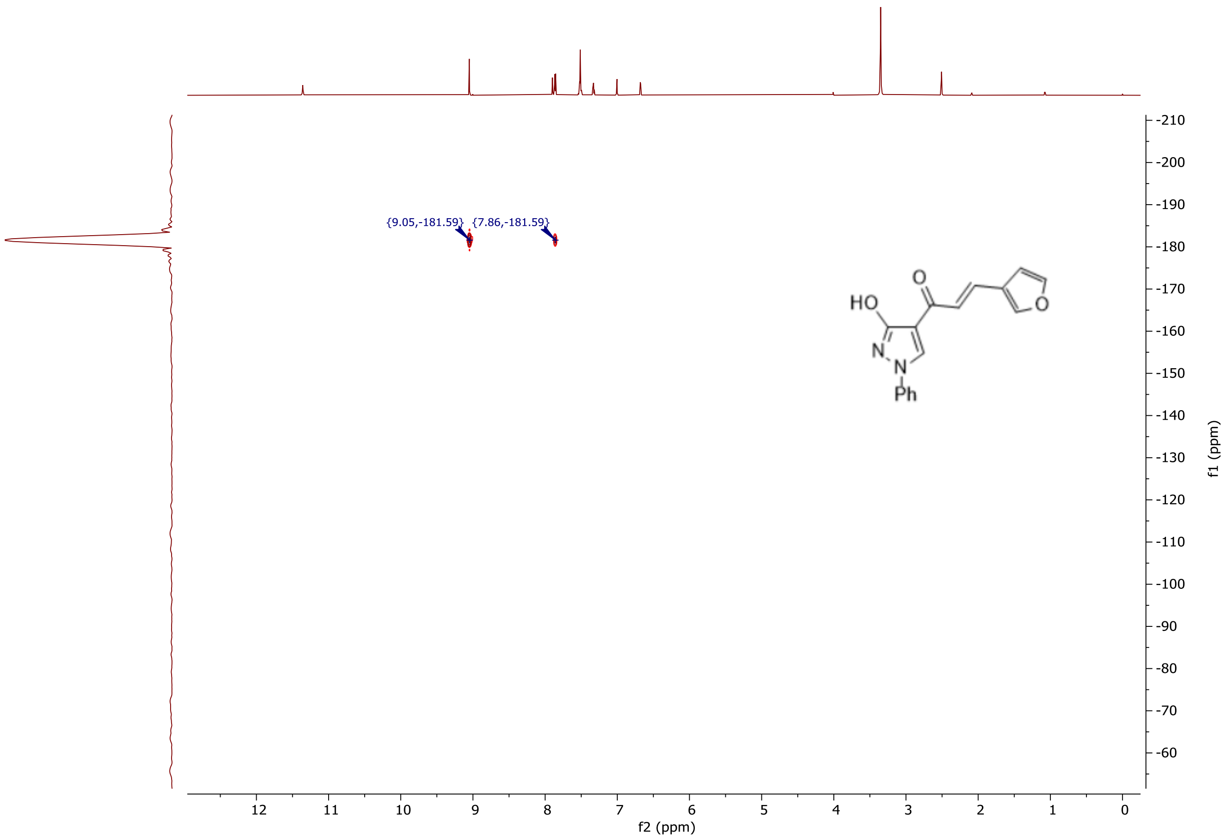

Figure S101. (2*E*)-3-(Furan-3-yl)-1-(3-hydroxy-1-phenyl-1*H*-pyrazol-4-yl)prop-2-en-1-one (8l). ^{1}H-^{15}N HMBC NMR spectrum (71 MHz, DMSO-d_6)

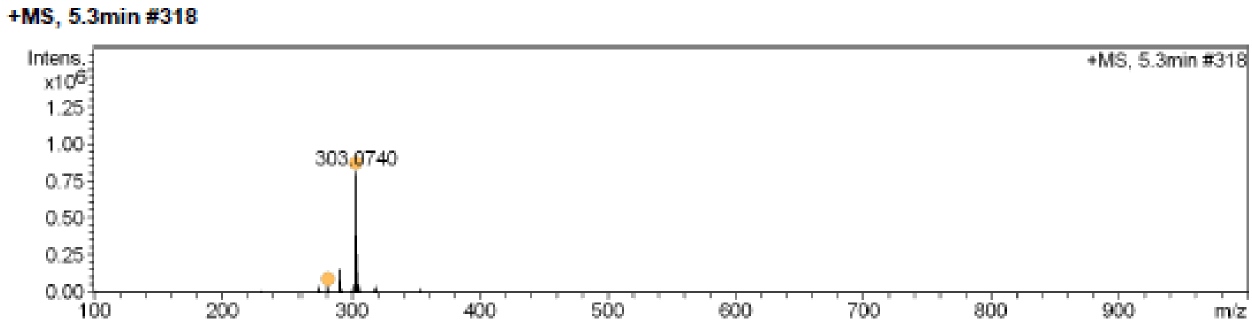

Meas. m/z	#	Ion Formula	m/z	err [ppm]	mSigma	# Sigma	Score	rdb	e⁻ Conf	N-Rule
281.0904	1	C16H13N2O3	281.0921	-5.9	9.6	1	100.00	11.5	even	ok
303.0740	1	C16H12N2NaO3	303.0740	-0.2	19.6	1	100.00	11.5	even	ok

Figure S102. (2*E*)-3-(Furan-3-yl)-1-(3-hydroxy-1-phenyl-1*H*-pyrazol-4-yl)prop-2-en-1-one (8l). HRMS (ESI-TOF).

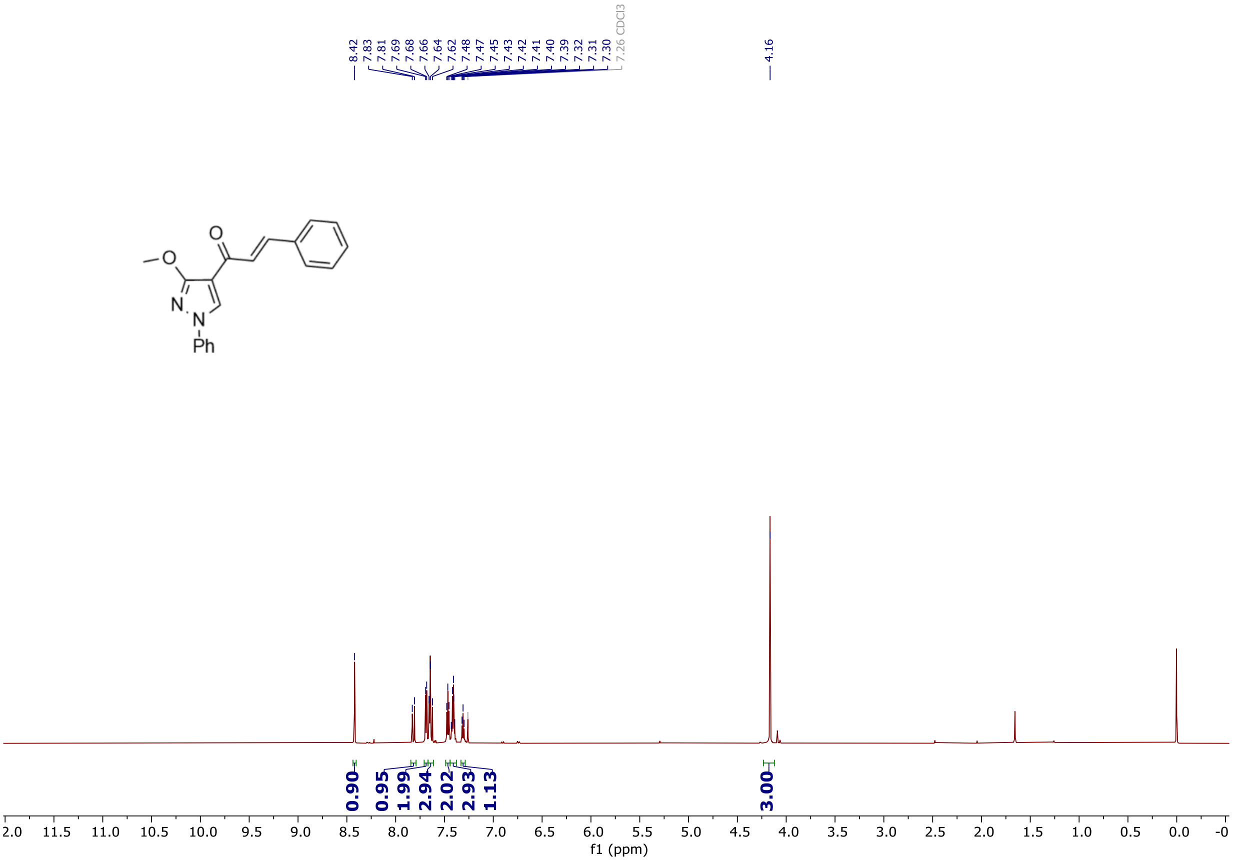

Figure S103. (2*E*)-1-(3-Methoxy-1-phenyl-1*H*-pyrazol-4-yl)-3-phenylprop-2-en-1-one (9a). ^{1}H NMR spectrum (700 MHz, $CDCl_3$)

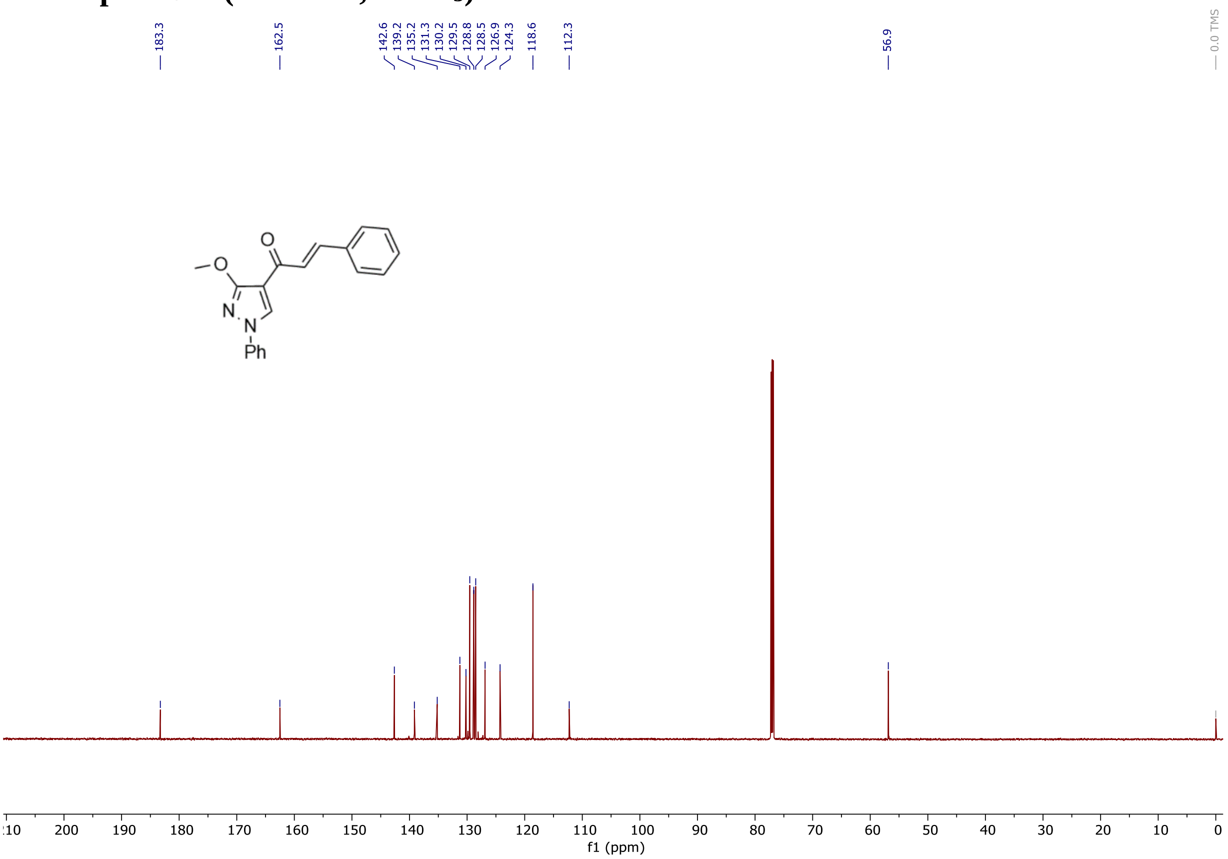

Figure S104. (2*E*)-1-(3-Methoxy-1-phenyl-1*H*-pyrazol-4-yl)-3-phenylprop-2-en-1-one (9a). ^{13}C NMR spectrum (176 MHz, $CDCl_3$)

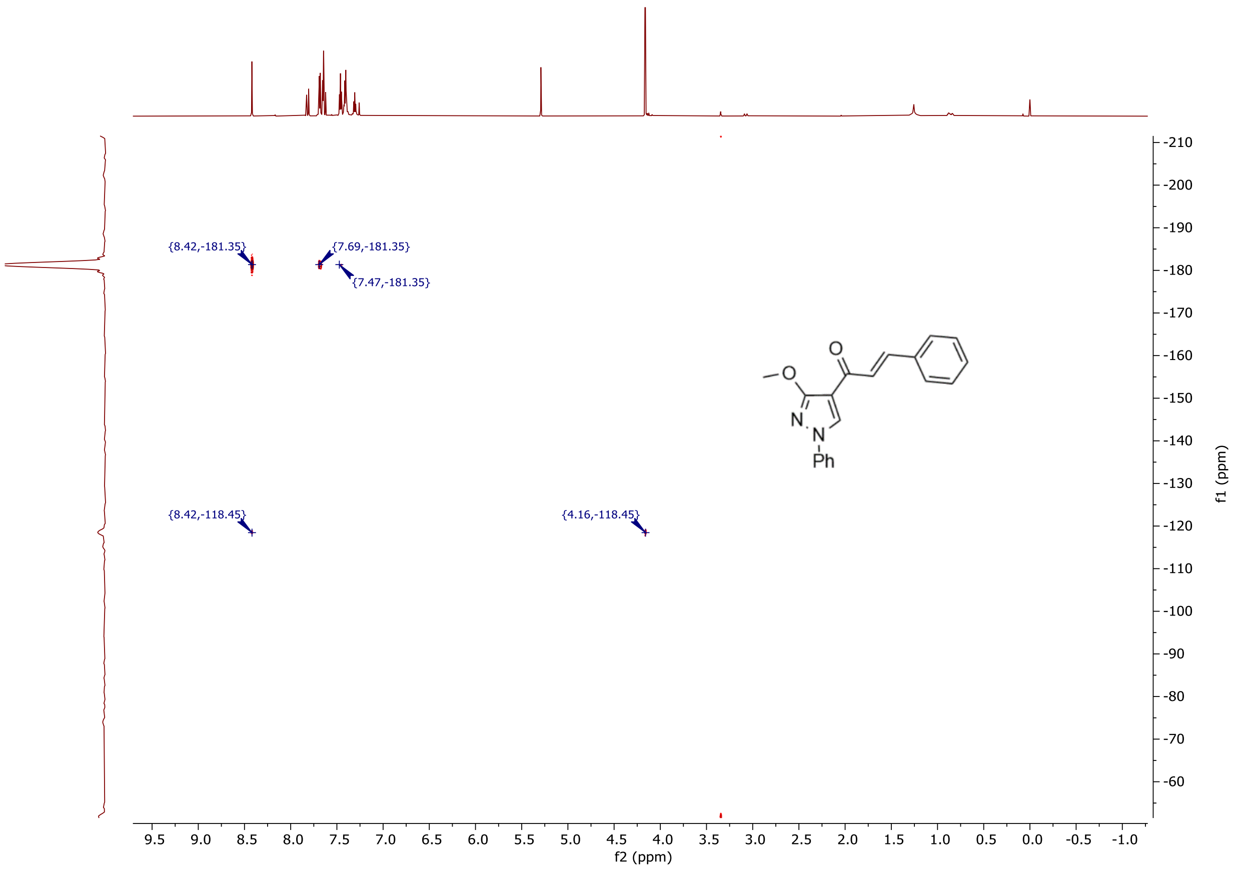

Figure S105. (2*E*)-1-(3-Methoxy-1-phenyl-1*H*-pyrazol-4-yl)-3-phenylprop-2-en-1-one (9a). ^{1}H-^{15}N HMBC NMR spectrum (71 MHz, $CDCl_3$)

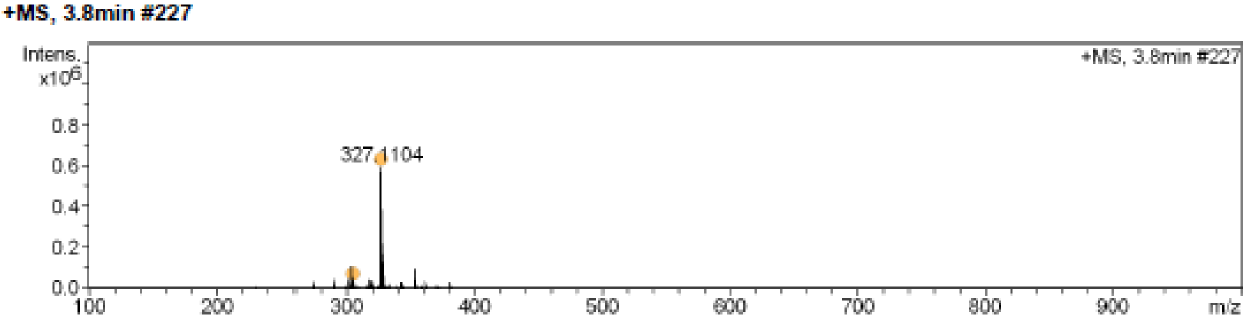

Meas. m/z	#	Ion Formula	m/z	err [ppm]	mSigma	# Sigma	Score	rdb	e¯ Conf	N-Rule
305.1281	1	C19H17N2O2	305.1285	-1.3	43.6	1	100.00	12.5	even	ok
327.1104	1	C19H16N2NaO2	327.1104	-0.1	8.8	1	100.00	12.5	even	ok

Figure S106. (2*E*)-1-(3-Methoxy-1-phenyl-1*H*-pyrazol-4-yl)-3-phenylprop-2-en-1-one (9a). HRMS (ESI-TOF).

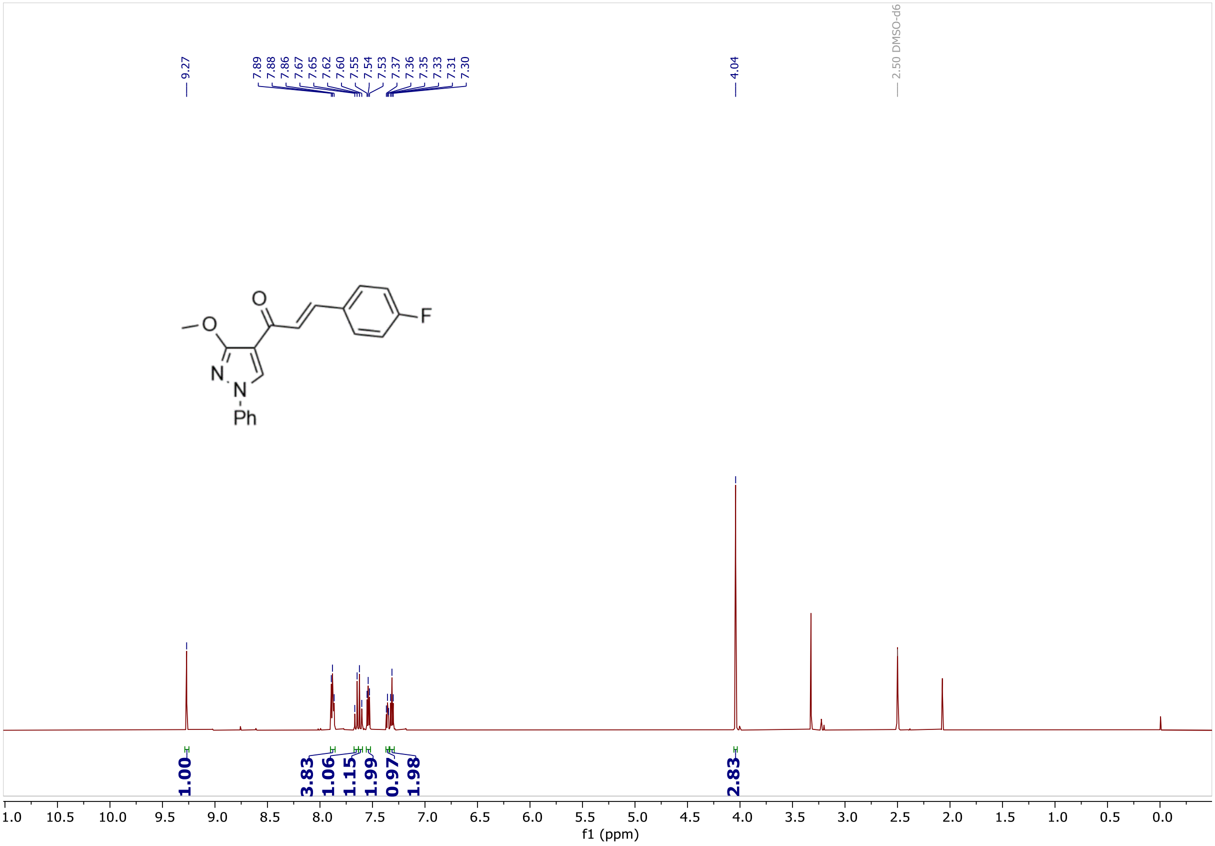

Figure S107. (2*E*)-3-(4-Fluorophenyl)-1-(3-methoxy-1-phenyl-1*H*-pyrazol-4-yl)prop-2-en-1-one (9b). ^{1}H NMR spectrum (700 MHz, DMSO-d_6)

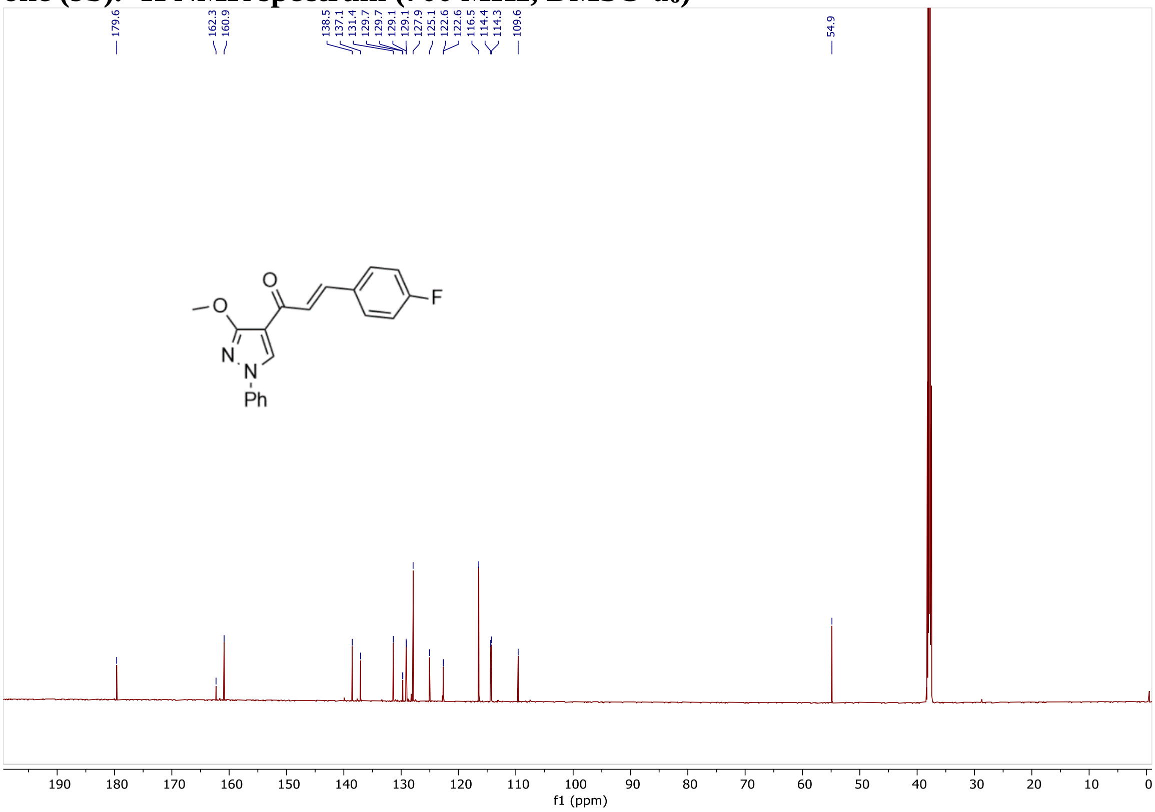

Figure S108. (2*E*)-3-(4-Fluorophenyl)-1-(3-methoxy-1-phenyl-1*H*-pyrazol-4-yl)prop-2-en-1-one (9b). ^{13}C NMR spectrum (176 MHz, DMSO-d_6)

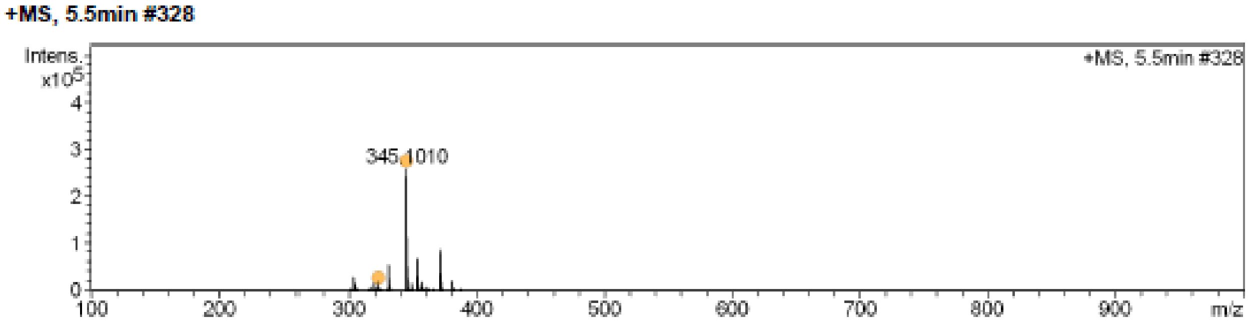

Meas. m/z	#	Ion Formula	m/z	err [ppm]	mSigma	# Sigma	Score	rdb	e⁻ Conf	N-Rule
323.1191	1	C19H16FN2O2	323.1190	0.2	18.2	1	100.00	12.5	even	ok
345.1010	1	C19H15FN2NaO2	345.1010	0.0	3.2	1	100.00	12.5	even	ok

Figure S109. (2*E*)-3-(4-Fluorophenyl)-1-(3-methoxy-1-phenyl-1*H*-pyrazol-4-yl)prop-2-en-1-one (9b). HRMS (ESI-TOF).

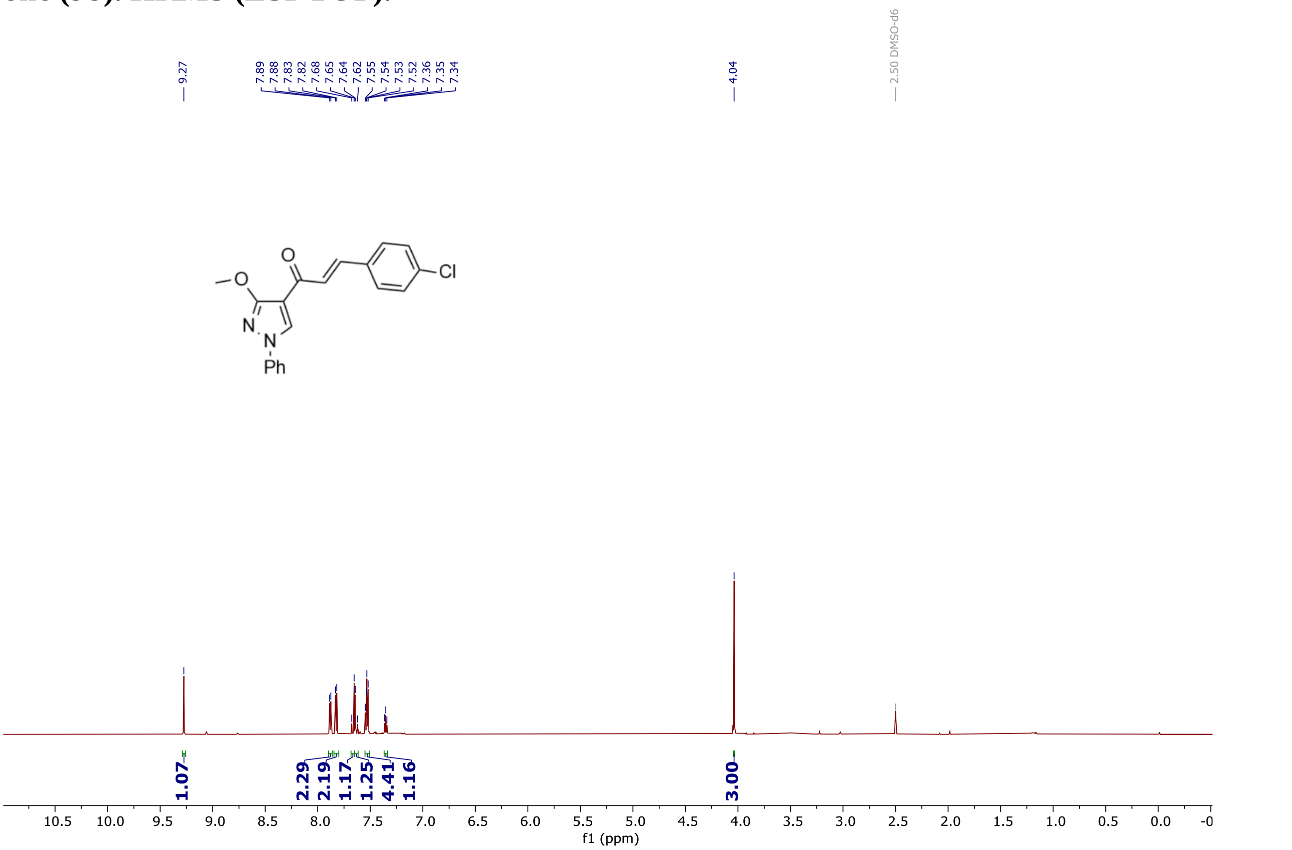

Figure S110. (2*E*)-3-(4-Chlorophenyl)-1-(3-methoxy-1-phenyl-1*H*-pyrazol-4-yl)prop-2-en-1-one (9c). ^{1}H NMR spectrum (700 MHz, DMSO-d_6)

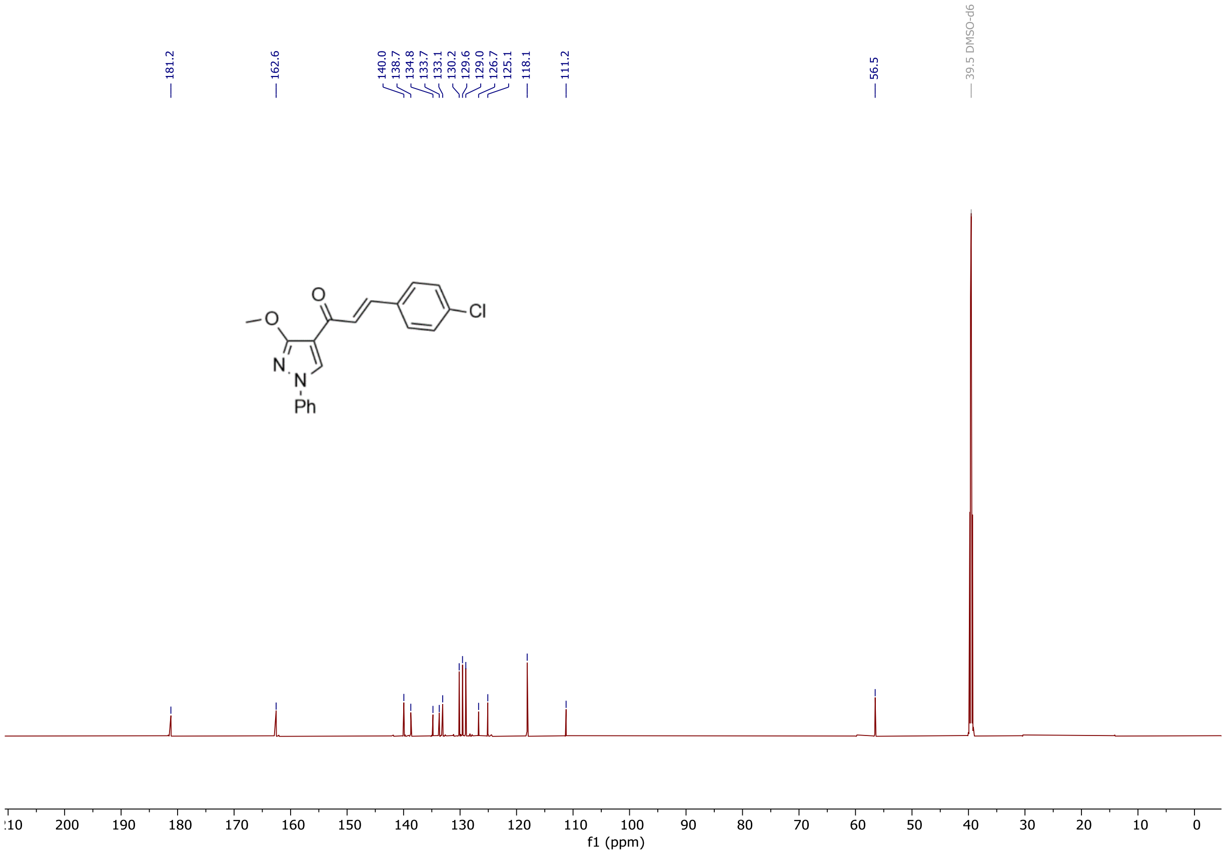

Figure S111. **(2*E*)-3-(4-Chlorophenyl)-1-(3-methoxy-1-phenyl-1*H*-pyrazol-4-yl)prop-2-en-1-one (9c).** ^{13}**C NMR spectrum (176 MHz, DMSO-*d*$_6$)**

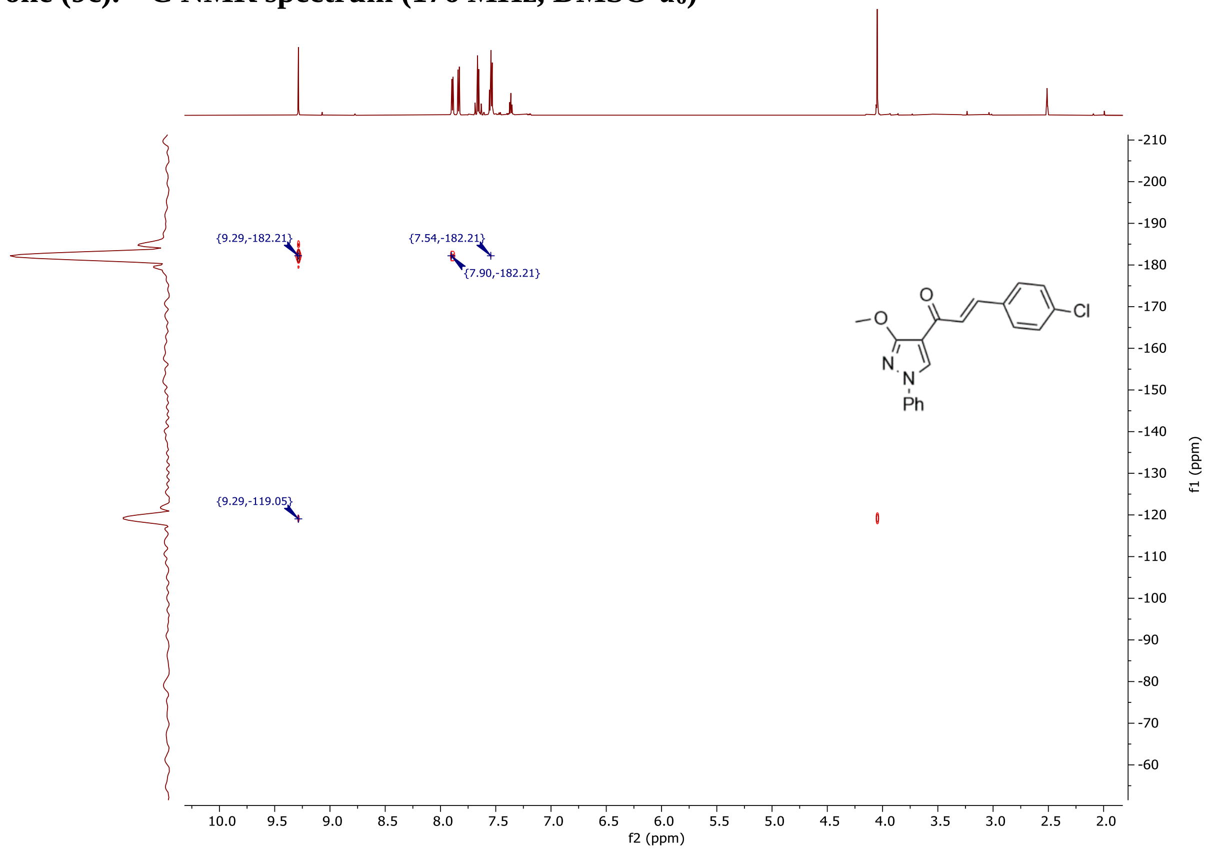

Figure S112. **(2*E*)-3-(4-Chlorophenyl)-1-(3-methoxy-1-phenyl-1*H*-pyrazol-4-yl)prop-2-en-1-one (9c).** ^{1}**H-**^{15}**N HMBC NMR spectrum (71 MHz, DMSO-*d*$_6$)**

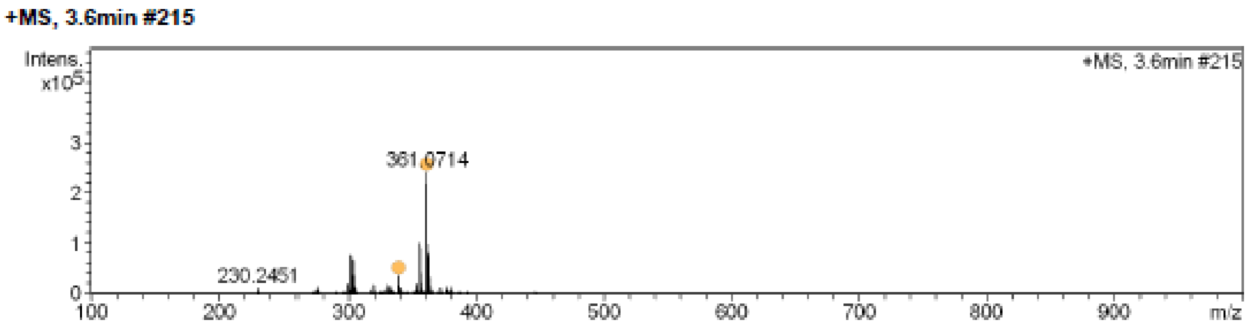

Meas. m/z	#	Ion Formula	m/z	err [ppm]	mSigma	# Sigma	Score	rdb	e⁻ Conf	N-Rule
339.0892	1	C19H16ClN2O2	339.0895	-1.0	13.6	1	100.00	12.5	even	ok
361.0714	1	C19H15ClN2NaO2	361.0714	-0.0	6.3	1	100.00	12.5	even	ok

Figure S113. (2*E*)-3-(4-Chlorophenyl)-1-(3-methoxy-1-phenyl-1*H*-pyrazol-4-yl)prop-2-en-1-one (9c). HRMS (ESI-TOF).

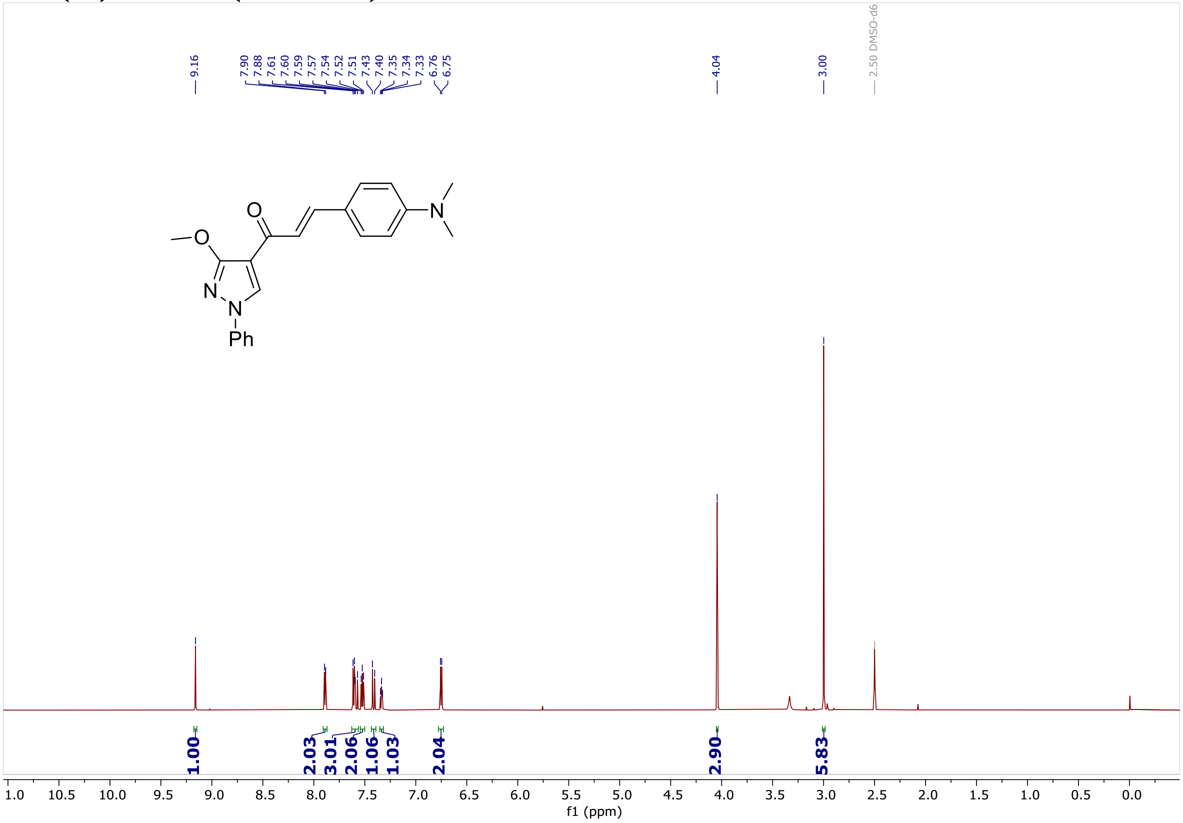

Figure S114. (2*E*)-3-[4-(Dimethylamino)phenyl]-1-(3-methoxy-1-phenyl-1*H*-pyrazol-4-yl)prop-2-en-1-one (9d). ^{1}H NMR spectrum (700 MHz, DMSO-d_6)

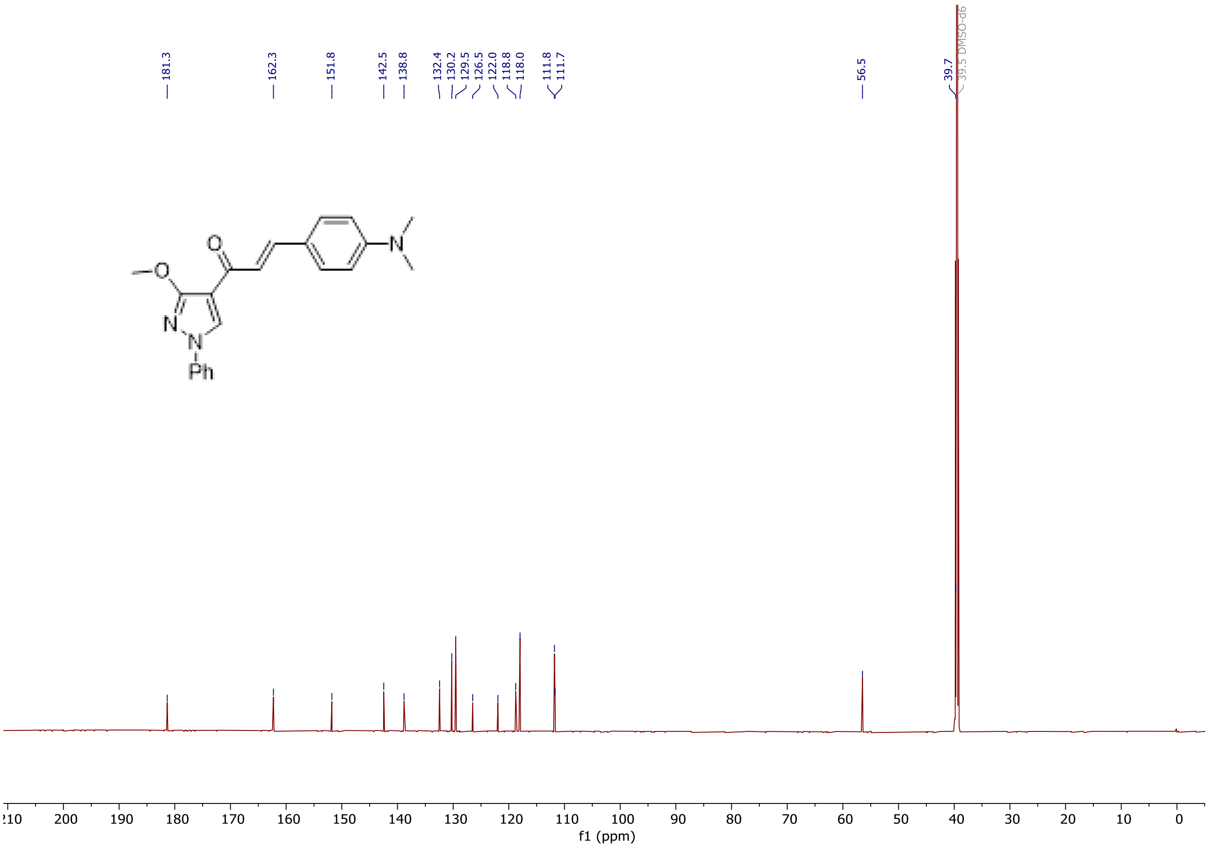

Figure S115. (2*E*)-3-[4-(Dimethylamino)phenyl]-1-(3-methoxy-1-phenyl-1*H*-pyrazol-4-yl)prop-2-en-1-one (9d). ^{13}**C NMR spectrum (176 MHz, DMSO-*d*$_6$)**

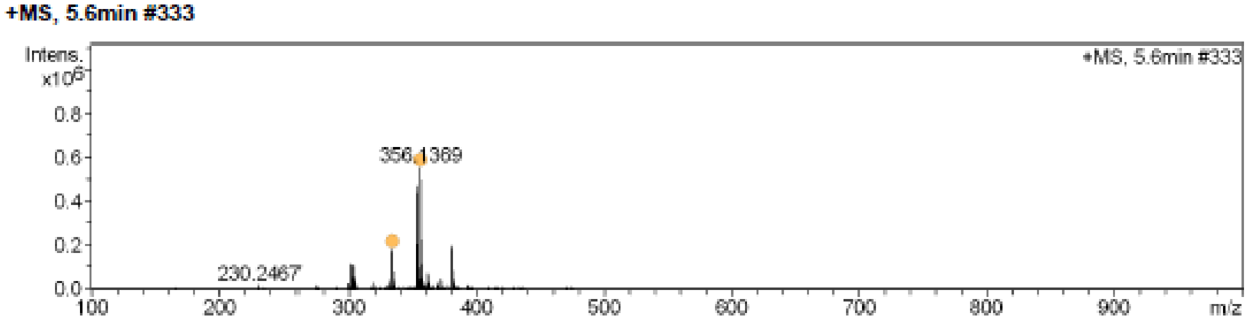

Meas. m/z	#	Ion Formula	m/z	err [ppm]	mSigma	# Sigma	Score	rdb	e⁻ Conf	N-Rule
334.1541	1	C20H20N3O2	334.1550	-2.6	14.2	1	100.00	12.5	even	ok
356.1369	1	C20H19N3NaO2	356.1369	-0.2	2.9	1	100.00	12.5	even	ok

Figure S116. (2*E*)-3-[4-(Dimethylamino)phenyl]-1-(3-methoxy-1-phenyl-1*H*-pyrazol-4-yl)prop-2-en-1-one (9d). HRMS (ESI-TOF).

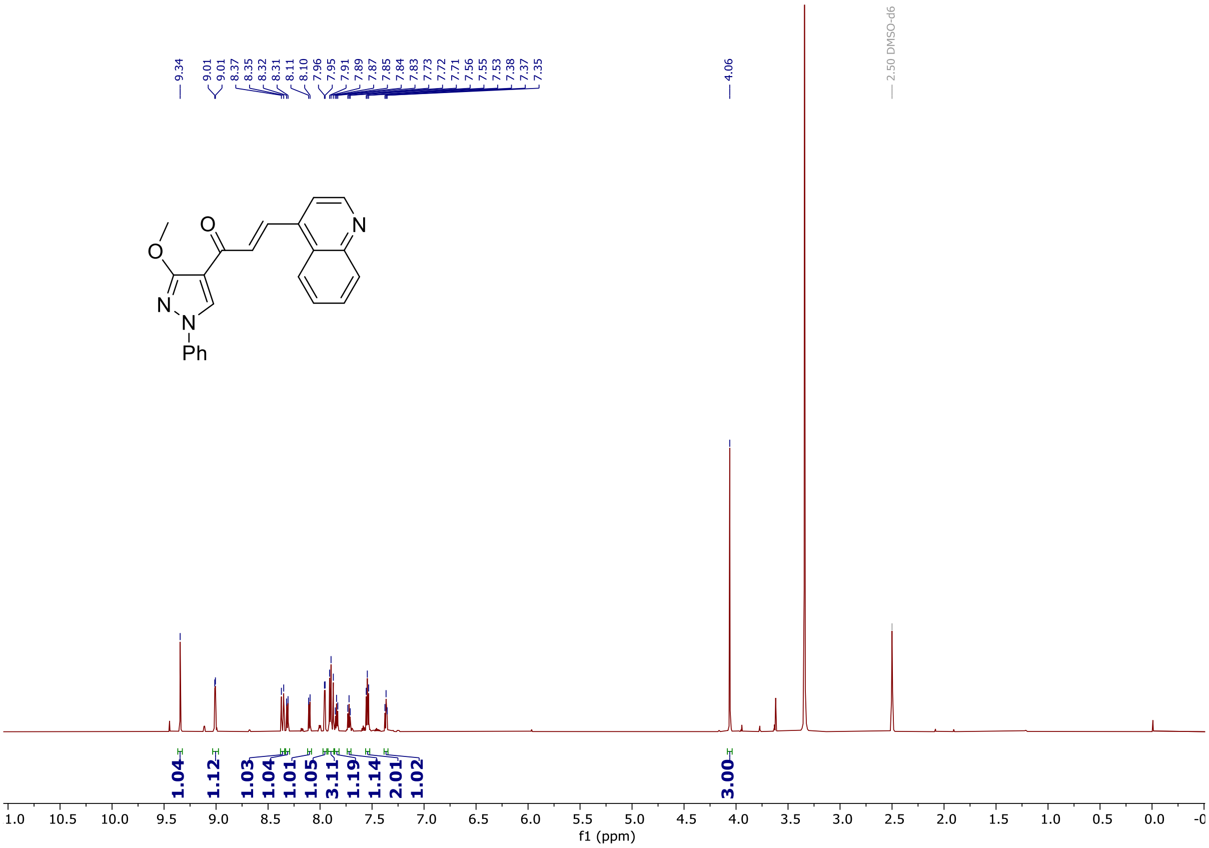

Figure S117. (2*E*)-1-(3-Methoxy-1-phenyl-1*H*-pyrazol-4-yl)-3-(quinolin-4-yl)prop-2-en-1-one (9e). ^{1}H NMR spectrum (700 MHz, DMSO-d_6)

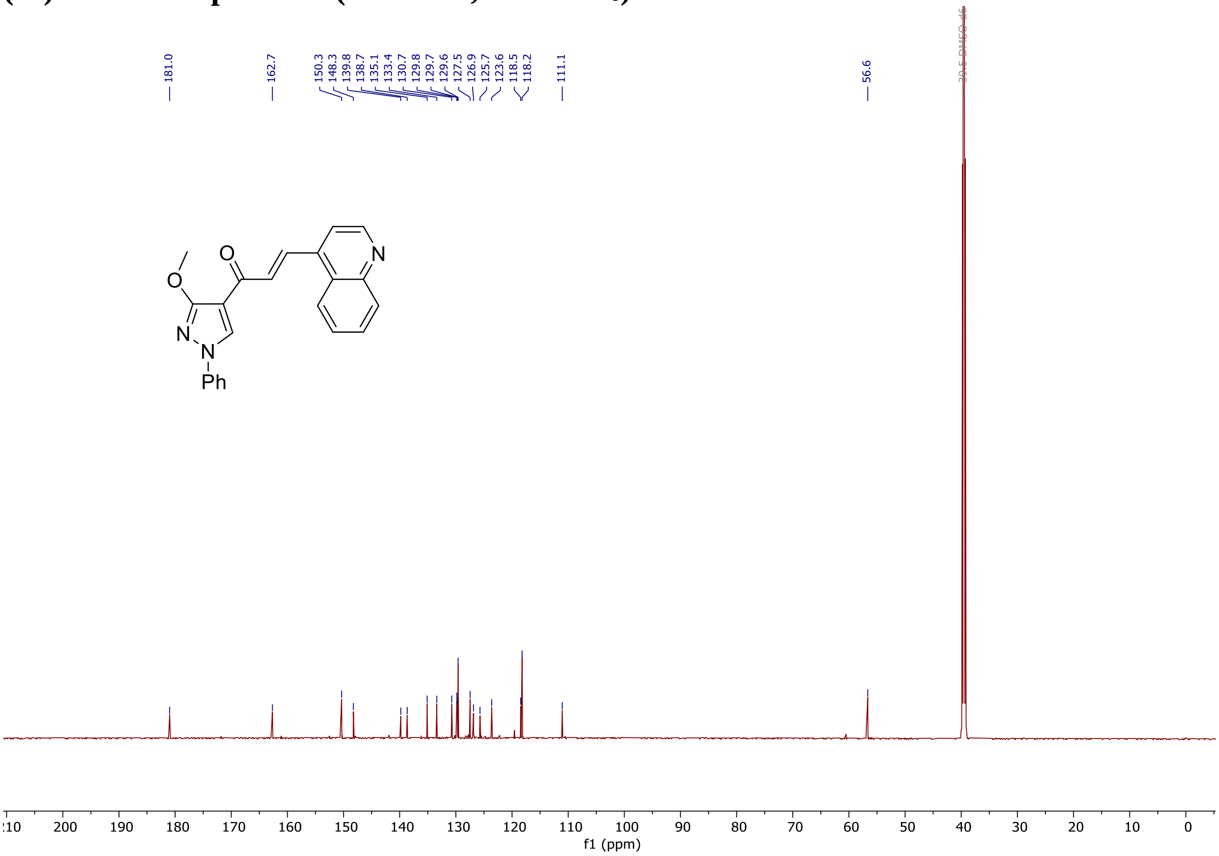

Figure S118. (2*E*)-1-(3-Methoxy-1-phenyl-1*H*-pyrazol-4-yl)-3-(quinolin-4-yl)prop-2-en-1-one (9e). ^{13}C NMR spectrum (176 MHz, DMSO-d_6)

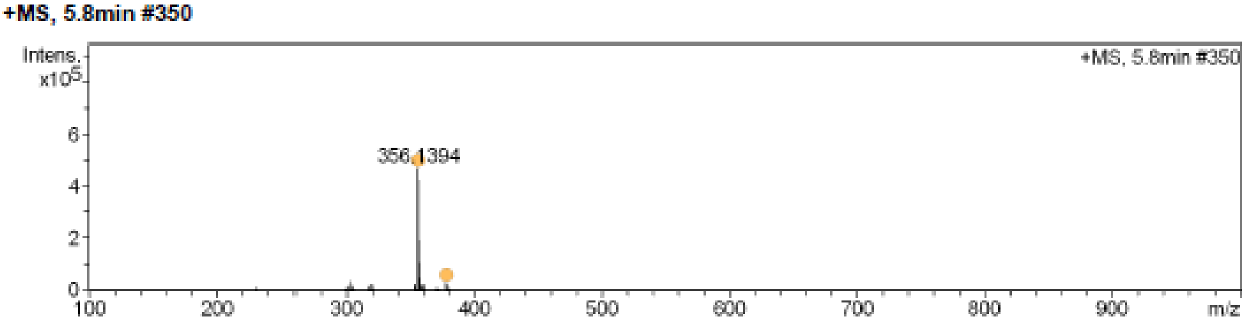

Meas. m/z	#	Ion Formula	m/z	err [ppm]	mSigma	# Sigma	Score	rdb	e⁻ Conf	N-Rule
356.1394	1	C22H18N3O2	356.1394	0.1	2.4	1	100.00	15.5	even	ok
378.1206	1	C22H17N3NaO2	378.1213	-1.7	6.8	1	100.00	15.5	even	ok

Figure S119. (2*E*)-1-(3-Methoxy-1-phenyl-1*H*-pyrazol-4-yl)-3-(quinolin-4-yl)prop-2-en-1-one (9e). HRMS (ESI-TOF).

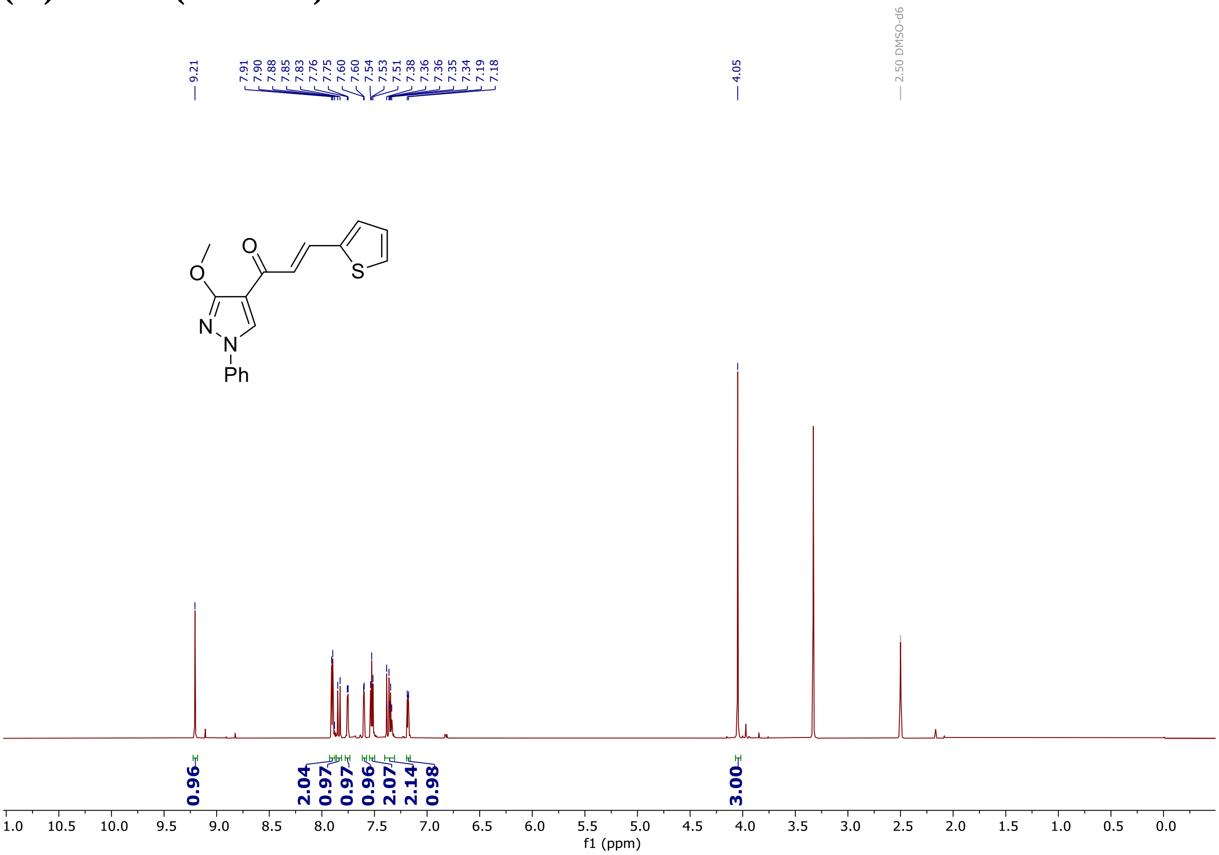

Figure S120. (2*E*)-1-(3-Methoxy-1-phenyl-1*H*-pyrazol-4-yl)-3-(thiophen-2-yl)prop-2-en-1-one (9f). ^{1}H NMR spectrum (700 MHz, DMSO-d_6)

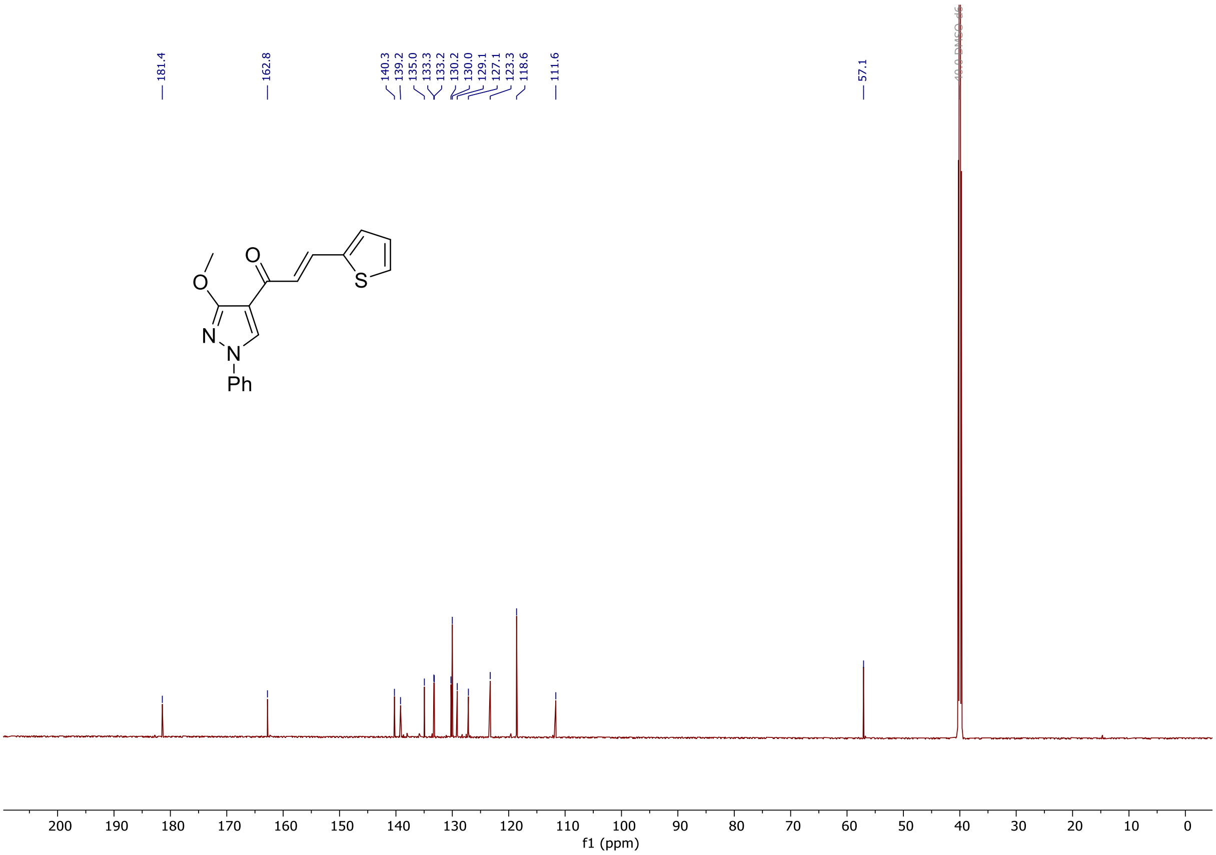

Figure S121. (2*E*)-1-(3-Methoxy-1-phenyl-1*H*-pyrazol-4-yl)-3-(thiophen-2-yl)prop-2-en-1-one (9f). ^{13}C NMR spectrum (176 MHz, DMSO-*d*$_6$)

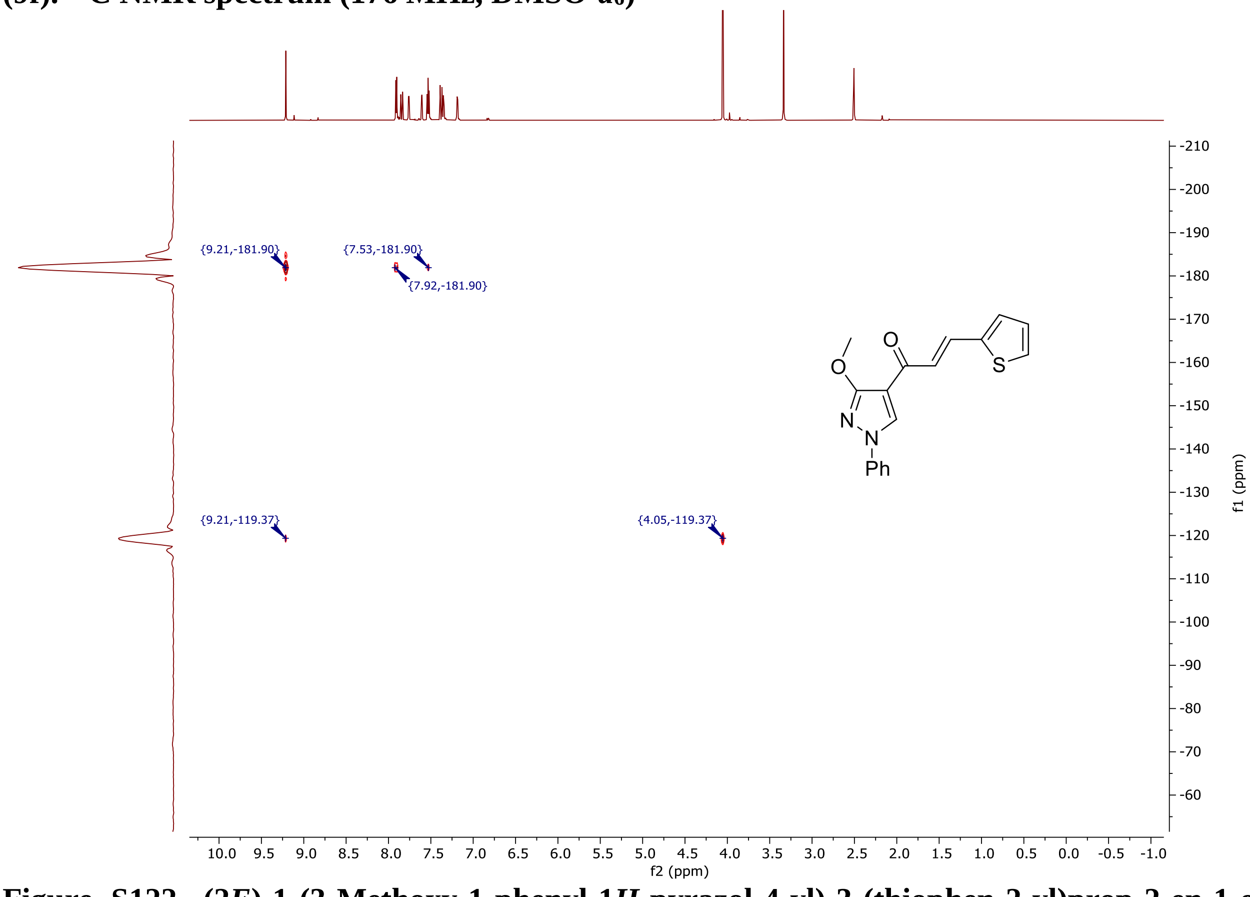

Figure S122. (2*E*)-1-(3-Methoxy-1-phenyl-1*H*-pyrazol-4-yl)-3-(thiophen-2-yl)prop-2-en-1-one (9f). ^{1}H-^{15}N HMBC NMR spectrum (71 MHz, DMSO-*d*$_6$)

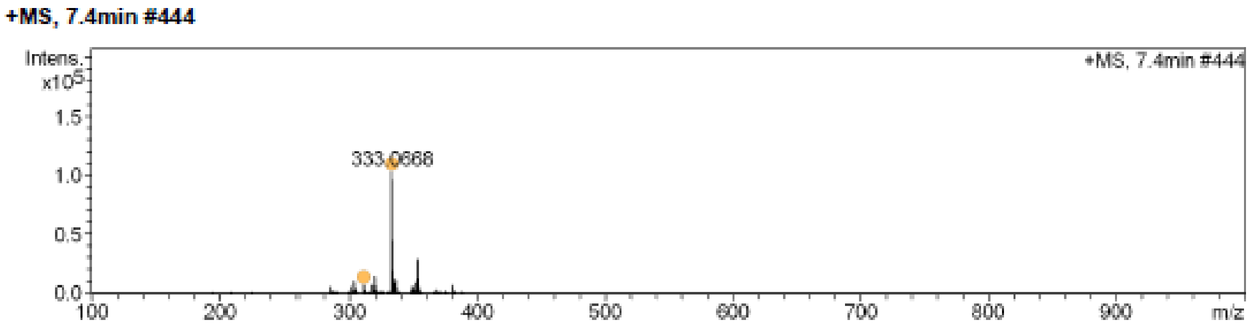

Meas. m/z	#	Ion Formula	m/z	err [ppm]	mSigma	# Sigma	Score	rdb	e¯ Conf	N-Rule
311.0851	1	C17H15N2O2S	311.0849	-0.8	75.0	1	100.00	11.5	even	ok
333.0668	1	C17H14N2NaO2S	333.0668	0.1	4.0	1	100.00	11.5	even	ok

Figure S123. (2*E*)-1-(3-Methoxy-1-phenyl-1*H*-pyrazol-4-yl)-3-(thiophen-2-yl)prop-2-en-1-one (9f). HRMS (ESI-TOF).

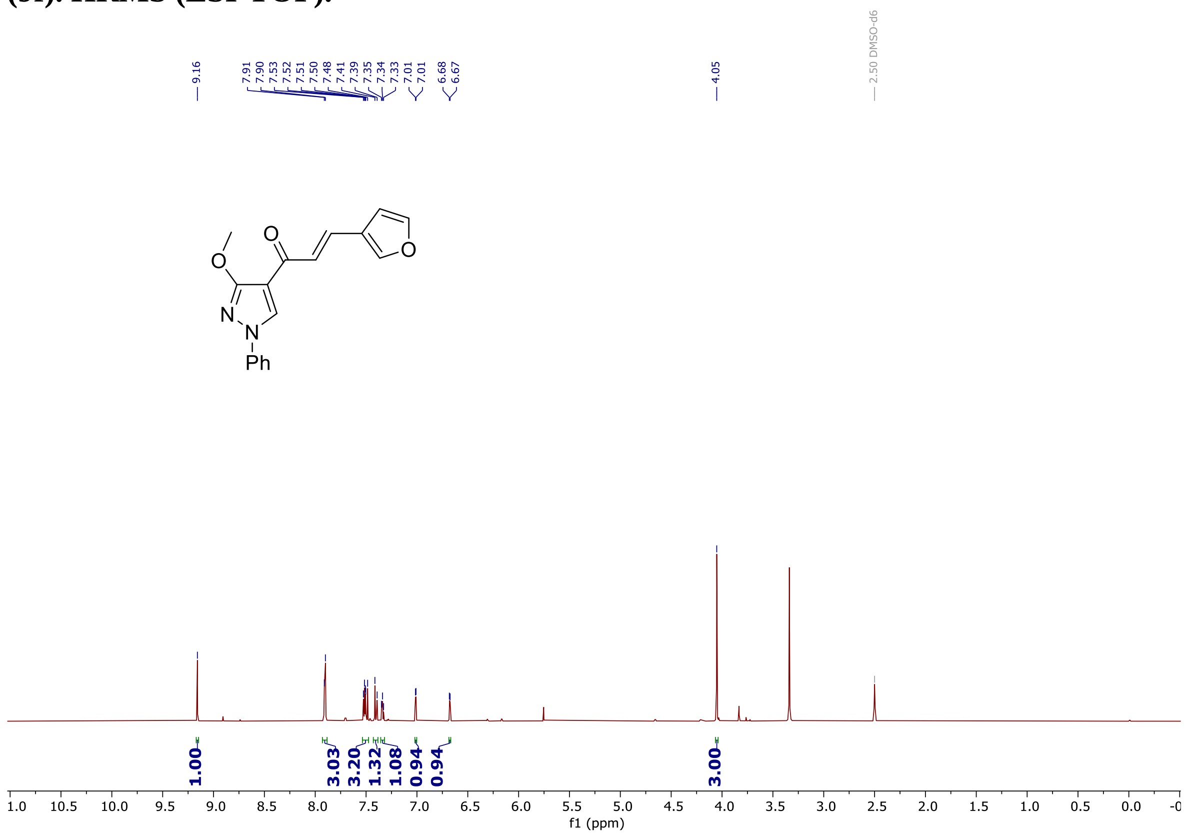

Figure S124. (2*E*)-3-(Furan-3-yl)-1-(3-methoxy-1-phenyl-1*H*-pyrazol-4-yl)prop-2-en-1-one (9g). ^{1}H NMR spectrum (700 MHz, DMSO-*d*$_6$):

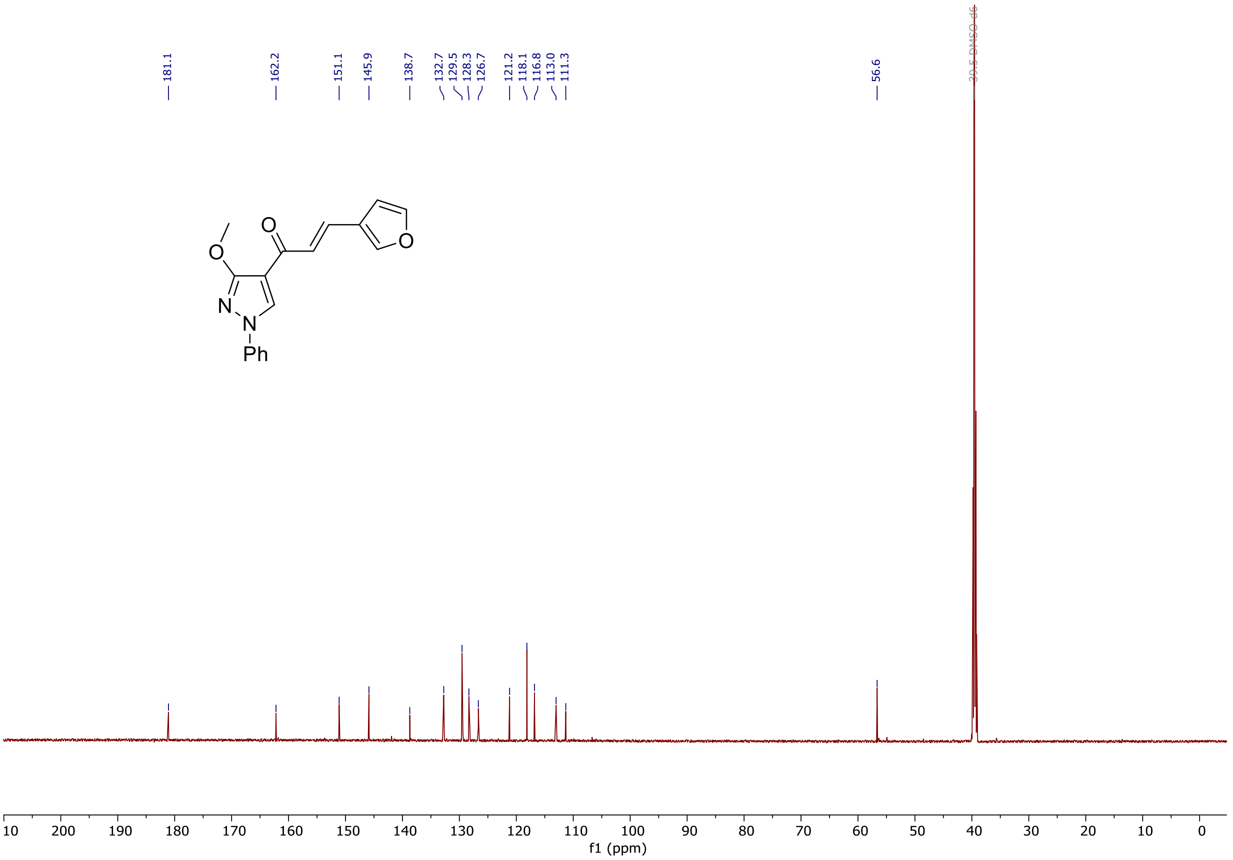

Figure S125. **(2*E*)-3-(Furan-3-yl)-1-(3-methoxy-1-phenyl-1*H*-pyrazol-4-yl)prop-2-en-1-one (9g). ^{13}C NMR spectrum (176 MHz, DMSO-d_6)**

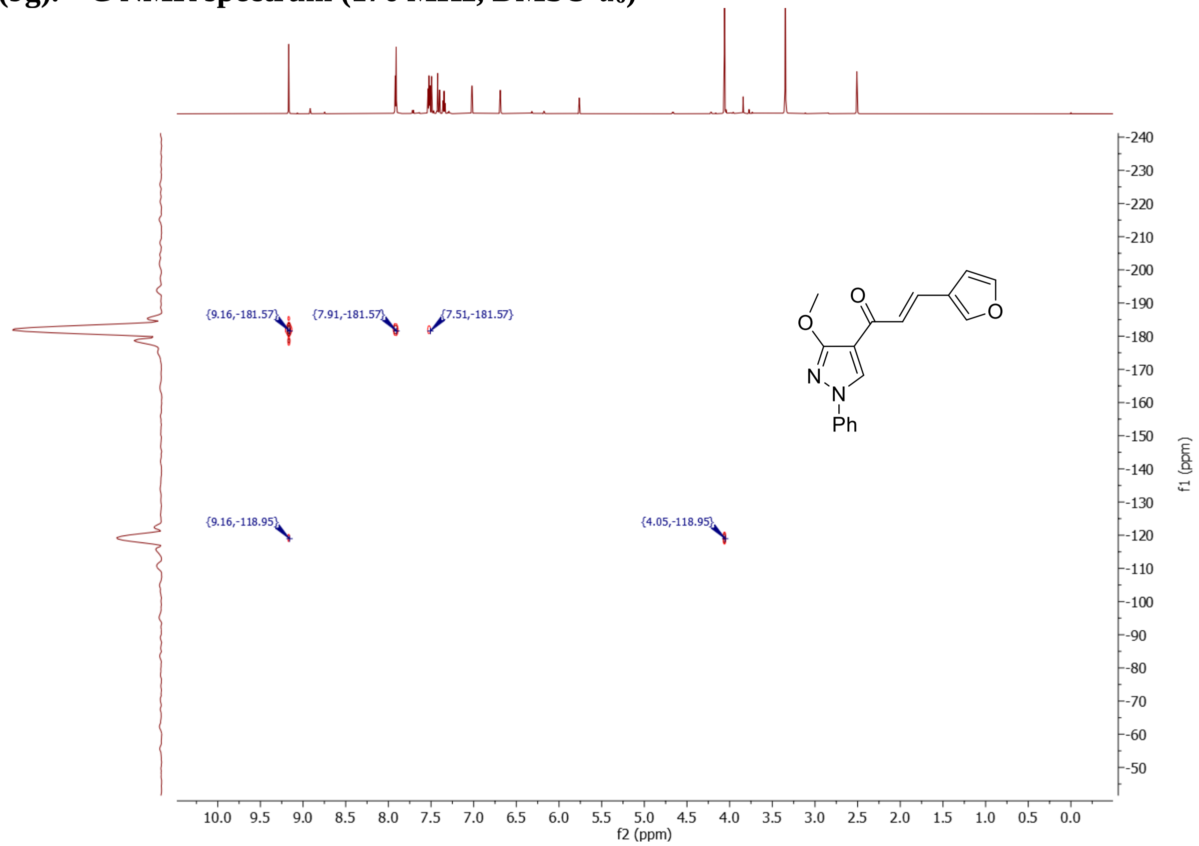

Figure S126. **(2*E*)-3-(Furan-3-yl)-1-(3-methoxy-1-phenyl-1*H*-pyrazol-4-yl)prop-2-en-1-one (9g). ^{1}H-^{15}N HMBC NMR spectrum (71 MHz, DMSO-d_6)**

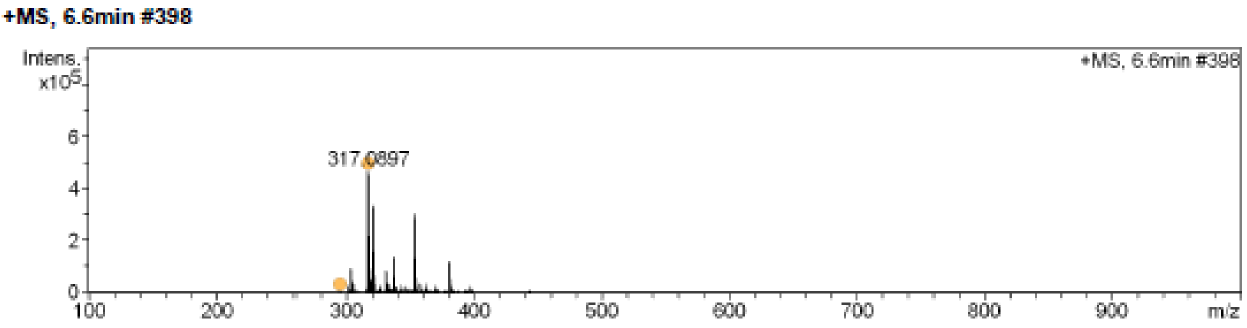

Meas. m/z	#	Ion Formula	m/z	err [ppm]	mSigma	# Sigma	Score	rdb	e¯ Conf	N-Rule
295.1057	1	C17H15N2O3	295.1077	-6.9	15.1	1	100.00	11.5	even	ok
317.0897	1	C17H14N2NaO3	317.0897	0.0	6.6	1	100.00	11.5	even	ok

Figure S127. (2*E*)-3-(Furan-3-yl)-1-(3-methoxy-1-phenyl-1*H*-pyrazol-4-yl)prop-2-en-1-one (9g). HRMS (ESI-TOF).

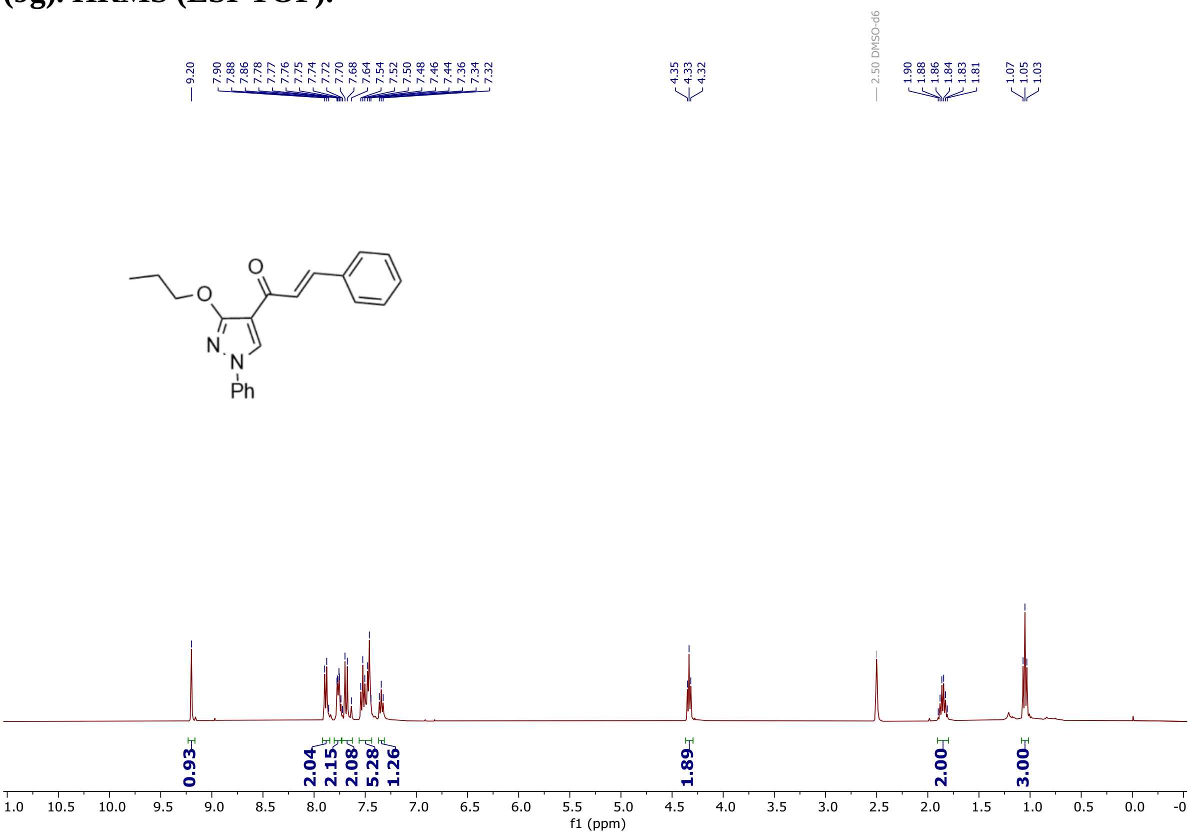

Figure S128. (2*E*)-3-Phenyl-1-(1-phenyl-3-propoxy-1*H*-pyrazol-4-yl)prop-2-en-1-one (9h). ^{1}H NMR spectrum (400 MHz, DMSO-d_6)

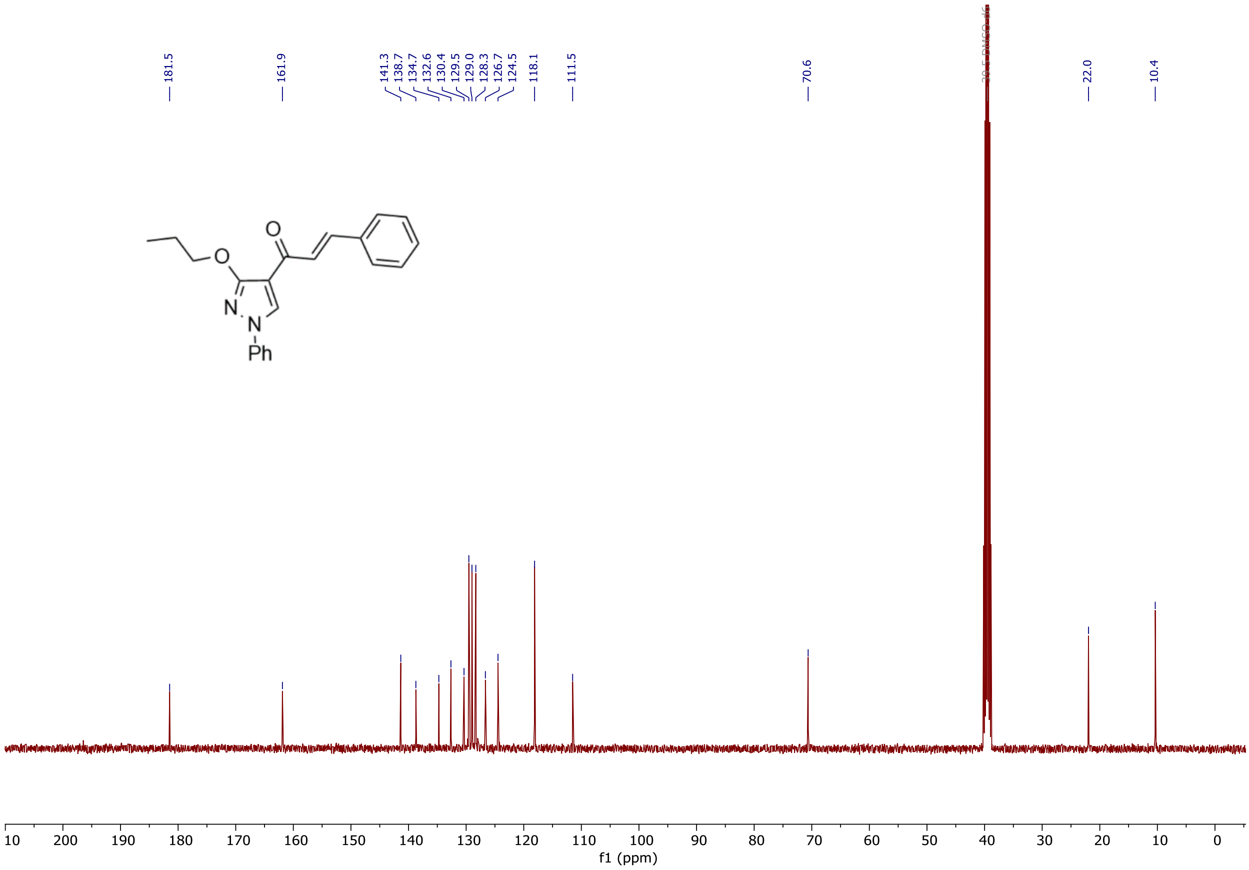

Figure S129. (2*E*)-3-Phenyl-1-(1-phenyl-3-propoxy-1*H*-pyrazol-4-yl)prop-2-en-1-one (9h). ^{13}C NMR spectrum (101 MHz, DMSO-d_6)

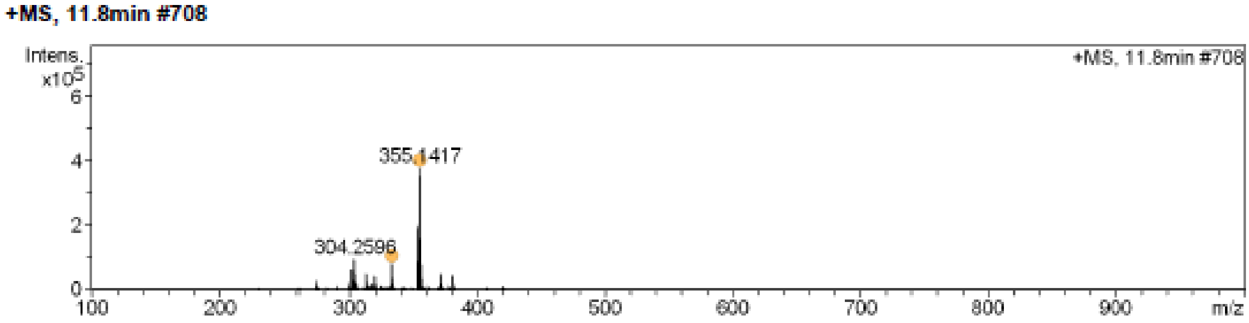

Meas. m/z	#	Ion Formula	m/z	err [ppm]	mSigma	# Sigma	Score	rdb	e⁻ Conf	N-Rule
333.1591	1	C21H21N2O2	333.1598	-2.0	4.0	1	100.00	12.5	even	ok
355.1417	1	C21H20N2NaO2	355.1417	-0.0	1.8	1	100.00	12.5	even	ok

Figure S130. (2*E*)-3-Phenyl-1-(1-phenyl-3-propoxy-1*H*-pyrazol-4-yl)prop-2-en-1-one (9h). HRMS (ESI-TOF).

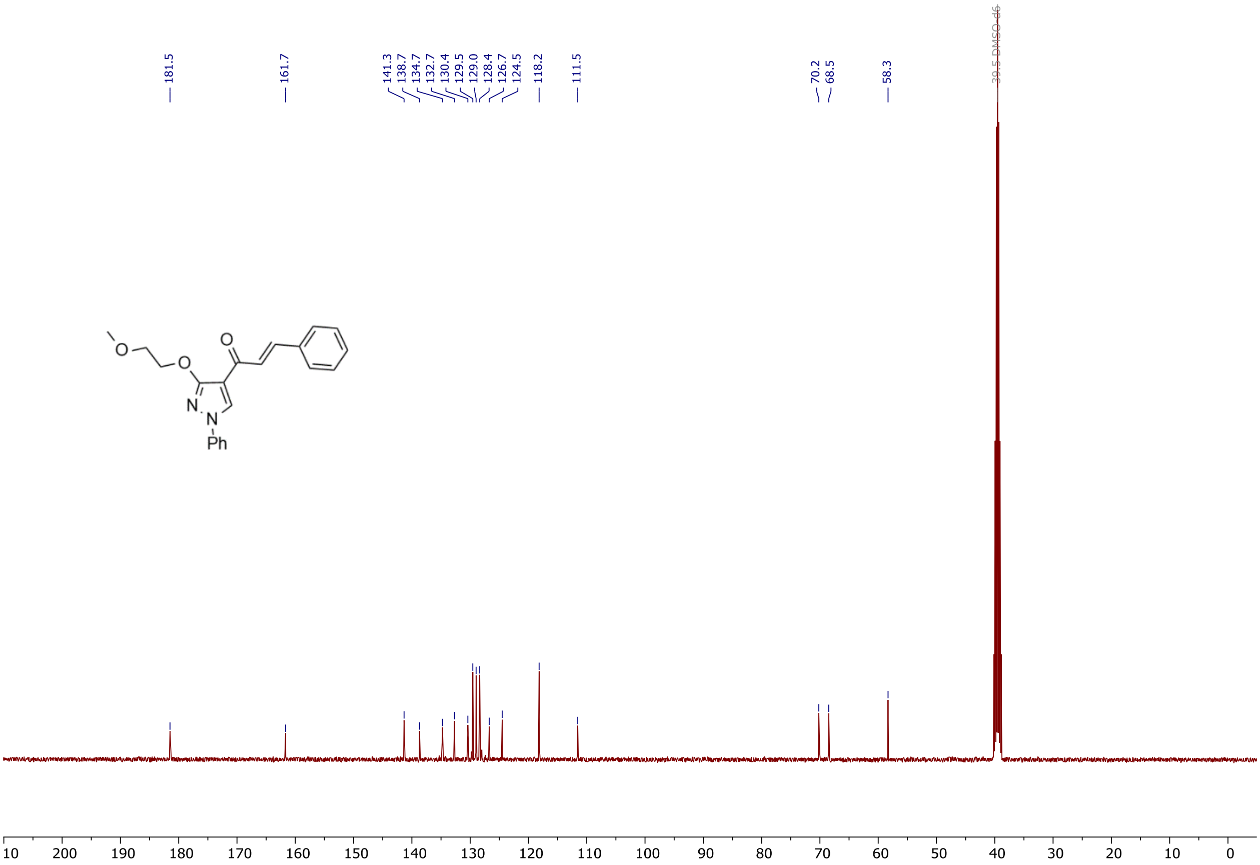

Figure S131. (2*E*)-1-[3-(2-Methoxyethoxy)-1-phenyl-1*H*-pyrazol-4-yl]-3-phenylprop-2-en-1-one (9i). ^{1}H NMR spectrum (400 MHz, DMSO-*d*$_6$)

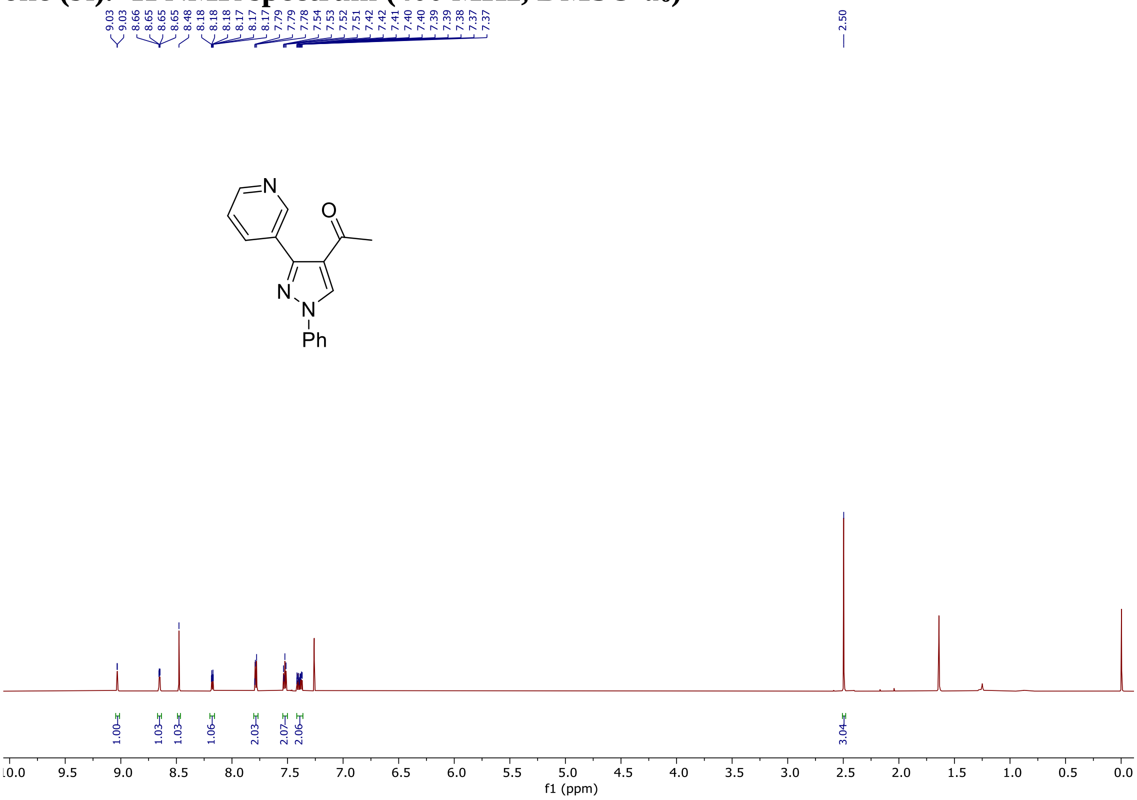

Figure S132. (2*E*)-1-[3-(2-Methoxyethoxy)-1-phenyl-1*H*-pyrazol-4-yl]-3-phenylprop-2-en-1-one (9i). ^{13}C NMR spectrum (101 MHz, DMSO-*d*$_6$)

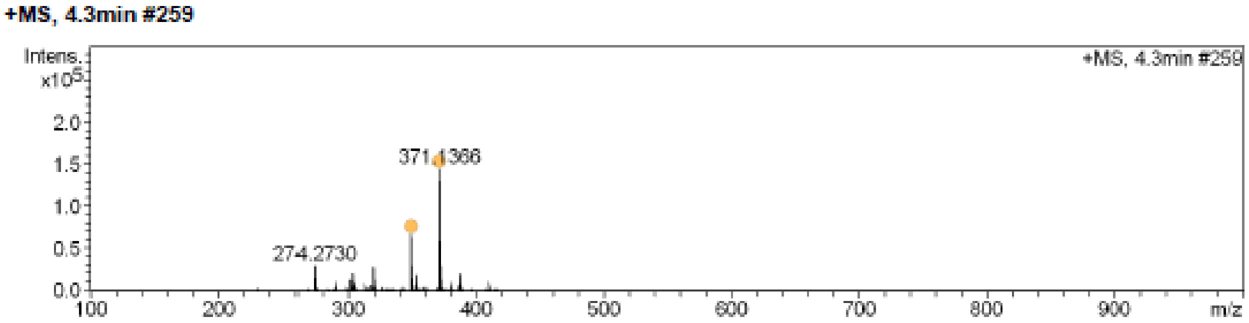

Meas. m/z	#	Ion Formula	m/z	err [ppm]	mSigma	# Sigma	Score	rdb	e^- Conf	N-Rule
349.1550	1	C21H21N2O3	349.1547	0.9	0.8	1	100.00	12.5	even	ok
371.1366	1	C21H20N2NaO3	371.1366	-0.1	5.0	1	100.00	12.5	even	ok

Figure S133. (2*E*)-1-[3-(2-Methoxyethoxy)-1-phenyl-1*H*-pyrazol-4-yl]-3-phenylprop-2-en-1-one (9i). HRMS (ESI-TOF).

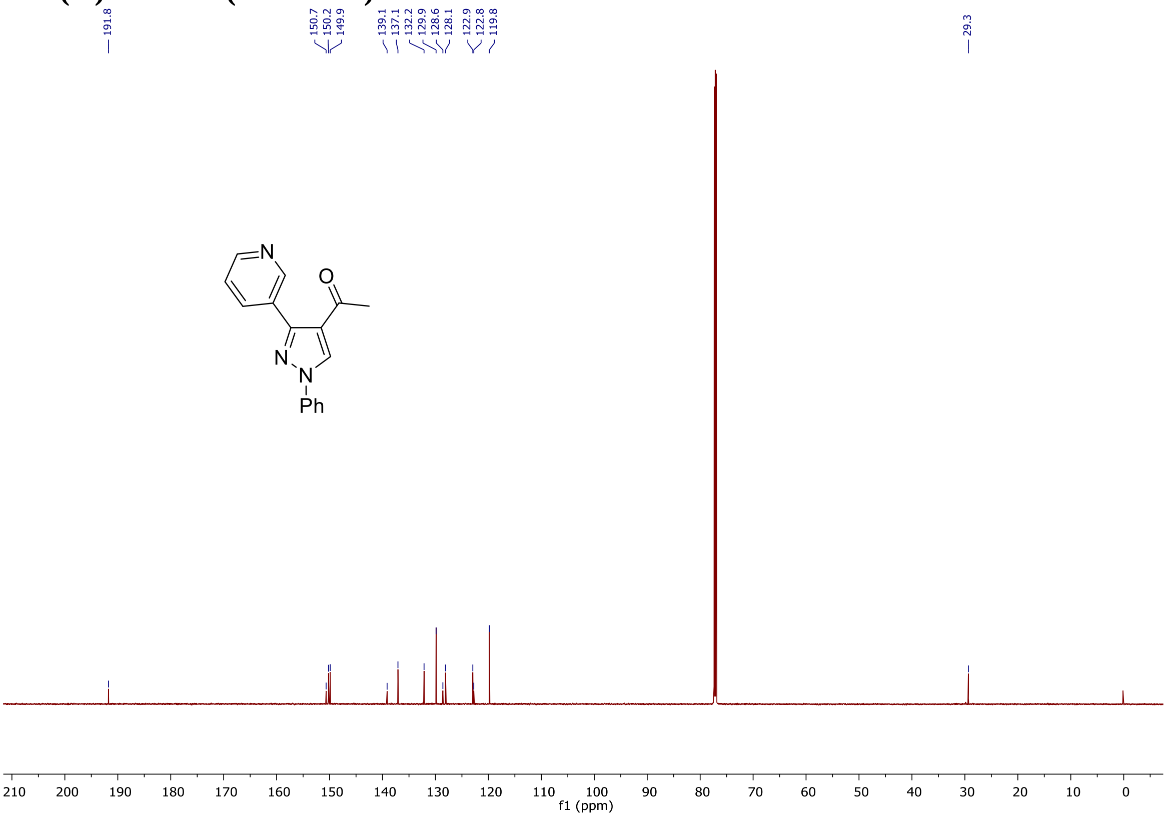

Figure S134. 1-[1-Phenyl-3-(pyridin-3-yl)-1*H*-pyrazol-4-yl]ethan-1-one (11a). ^{13}C NMR spectrum (176 MHz, $CDCl_3$)

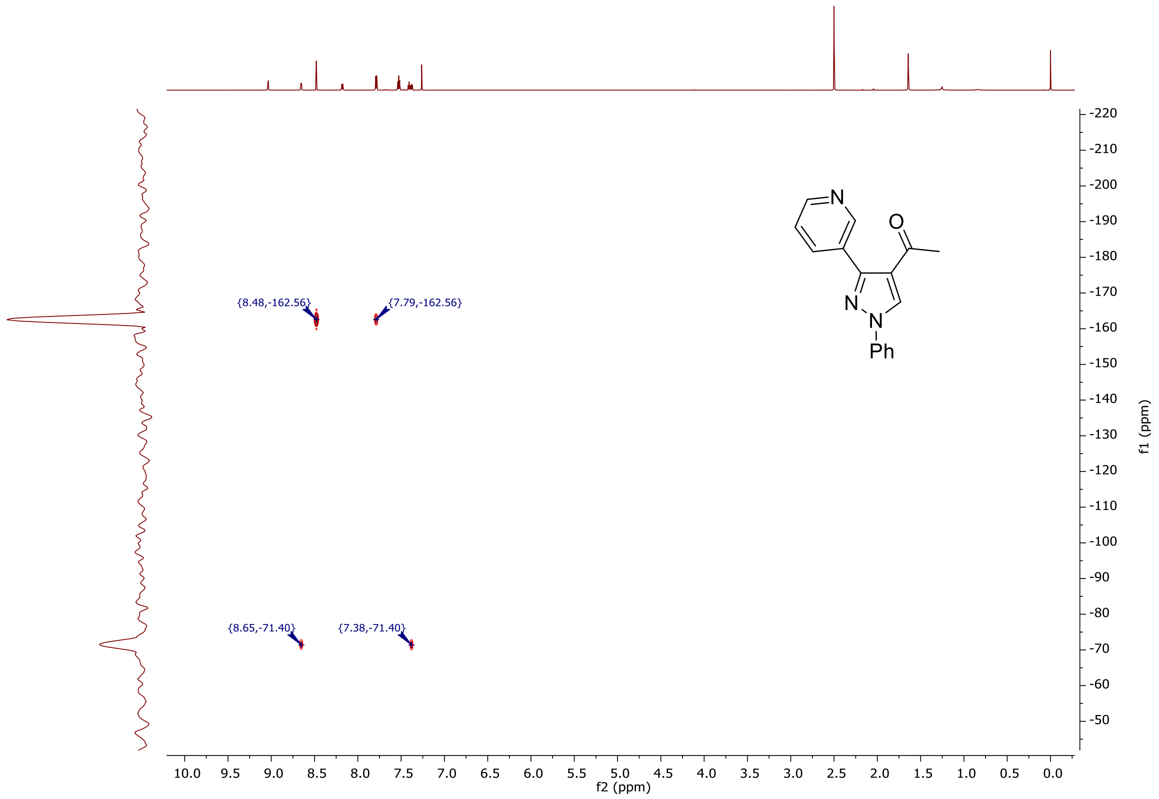

Figure S135. 1-[1-Phenyl-3-(pyridin-3-yl)-1*H*-pyrazol-4-yl]ethan-1-one (11a). ^{1}H-^{15}N HMBC NMR spectrum (71 MHz, $CDCl_3$)

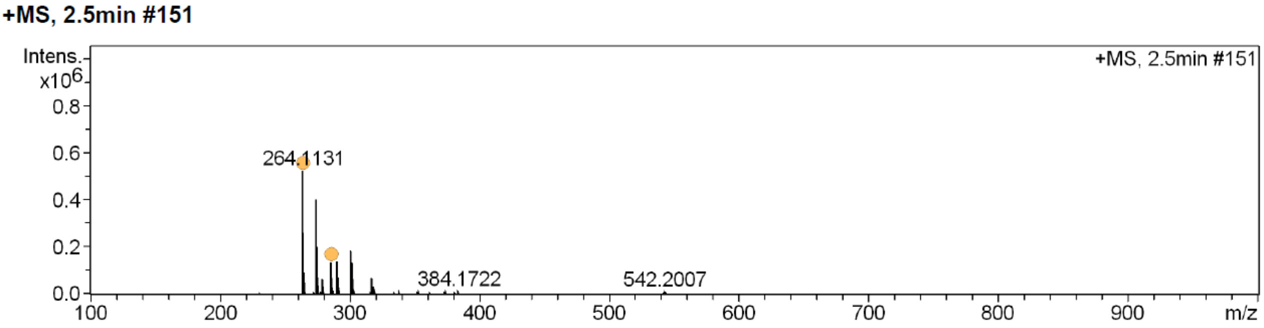

Meas. m/z	#	Ion Formula	m/z	err [ppm]	mSigma	# Sigma	Score	rdb	e⁻ Conf	N-Rule
264.1131	1	C16H14N3O	264.1131	-0.2	0.5	1	100.00	11.5	even	ok
286.0948	1	C16H13N3NaO	286.0951	1.1	4.2	1	100.00	11.5	even	ok

Figure S136. 1-[1-Phenyl-3-(pyridin-3-yl)-1*H*-pyrazol-4-yl]ethan-1-one (11a). HRMS (ESI-TOF).

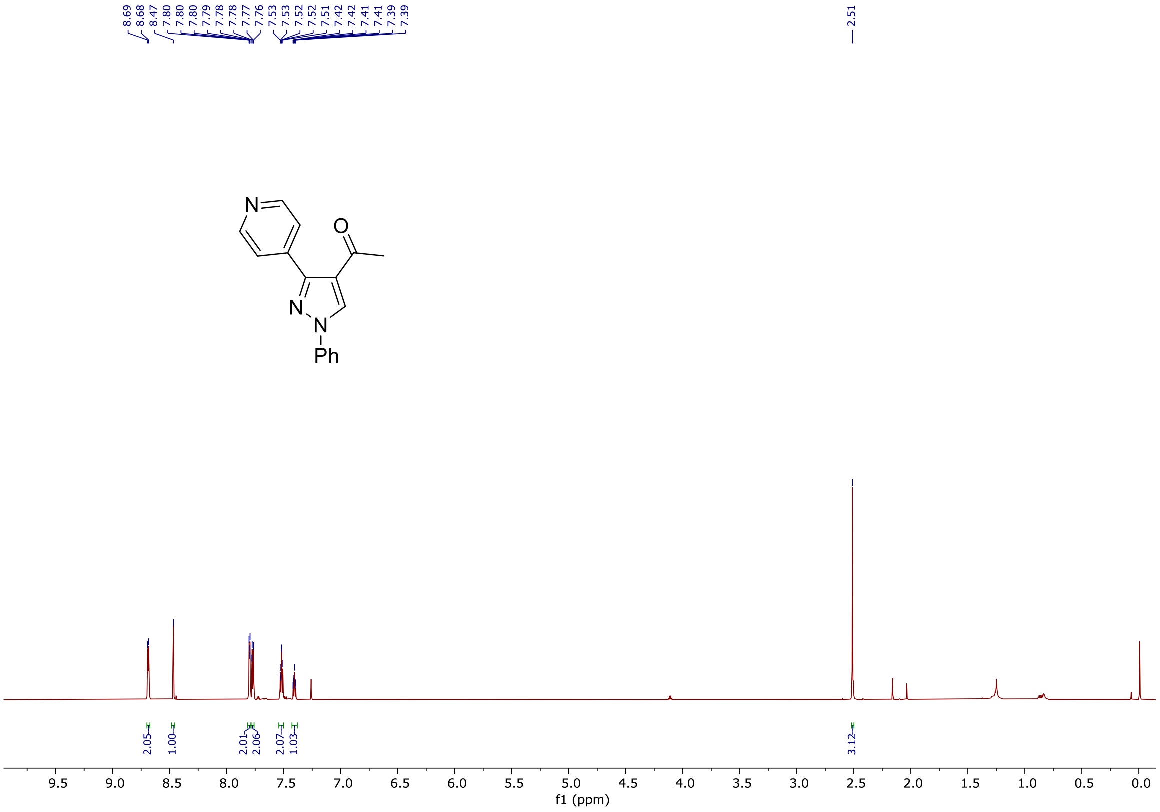

Figure S137. 1-[1-Phenyl-3-(pyridin-4-yl)-1*H*-pyrazol-4-yl]ethan-1-one (11b). ^{1}H NMR spectrum (700 MHz, $CDCl_3$)

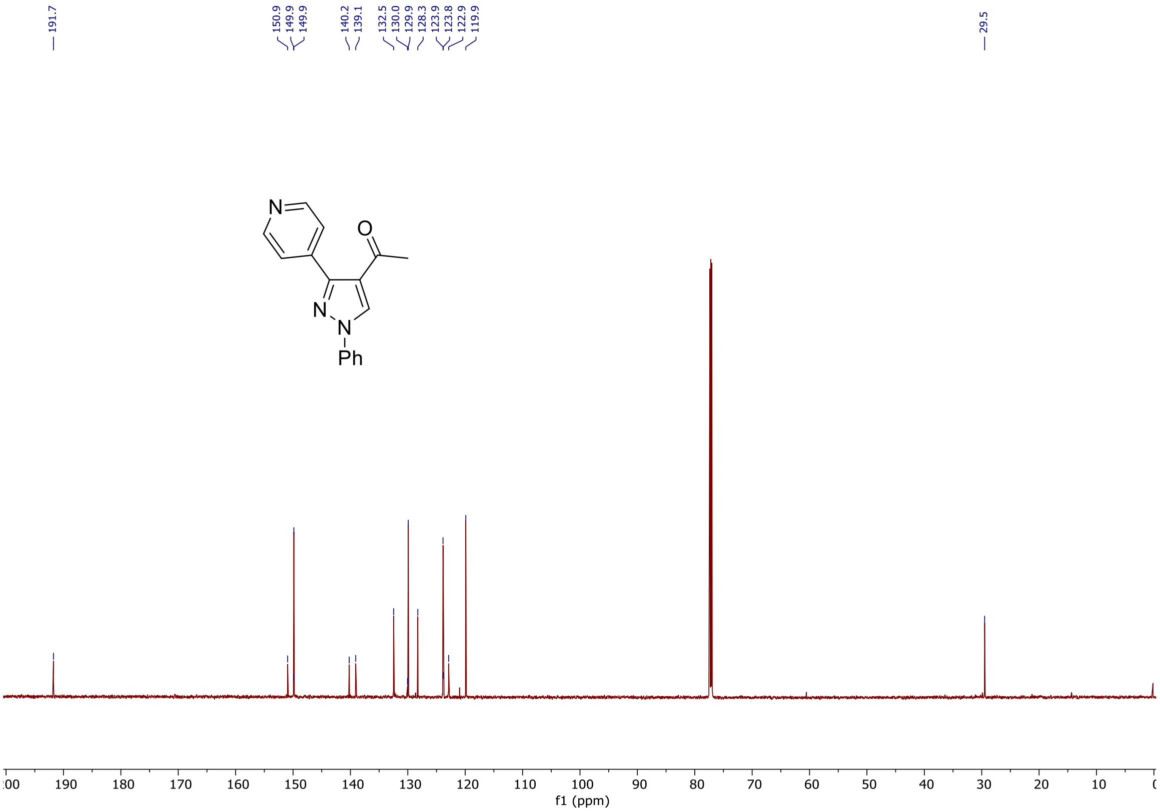

Figure S138. 1-[1-Phenyl-3-(pyridin-4-yl)-1*H*-pyrazol-4-yl]ethan-1-one (11b). ^{13}C NMR spectrum (176 MHz, $CDCl_3$)

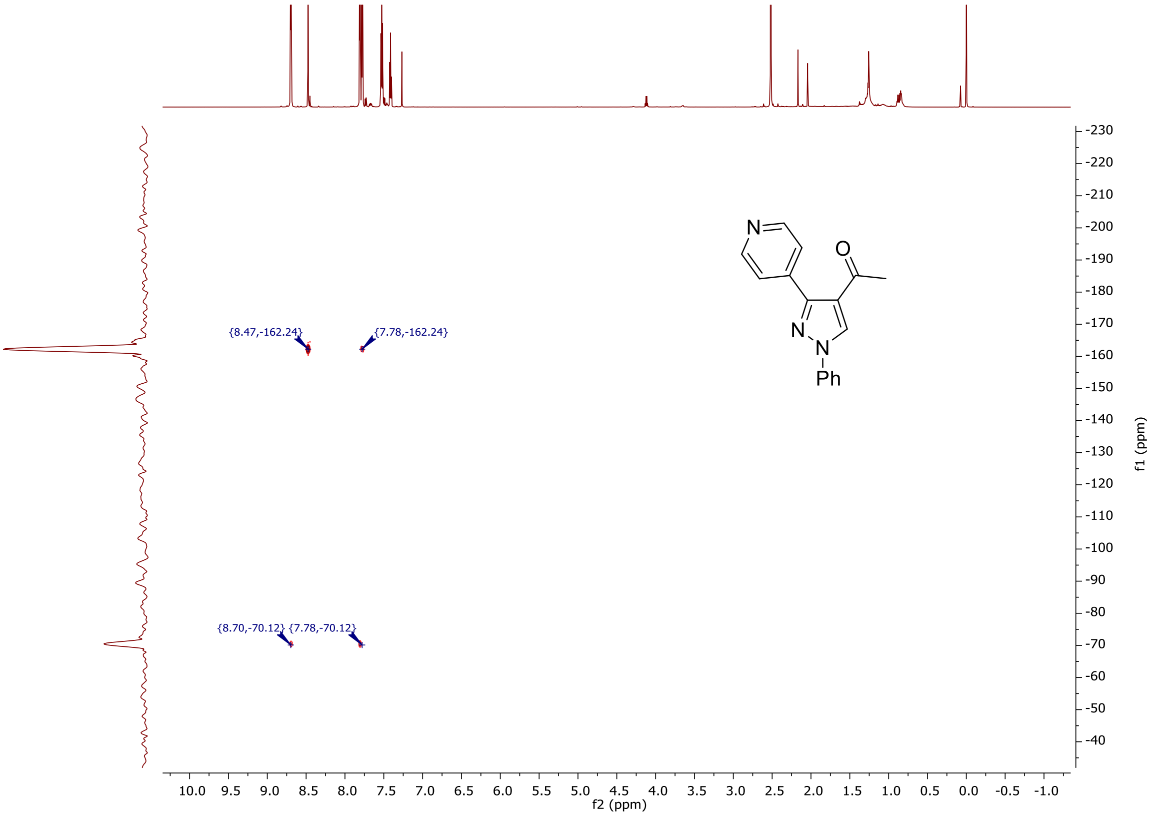

Figure S139. 1-[1-Phenyl-3-(pyridin-4-yl)-1*H*-pyrazol-4-yl]ethan-1-one (11b). ^{1}H-^{15}N HMBC NMR spectrum (71 MHz, $CDCl_3$)

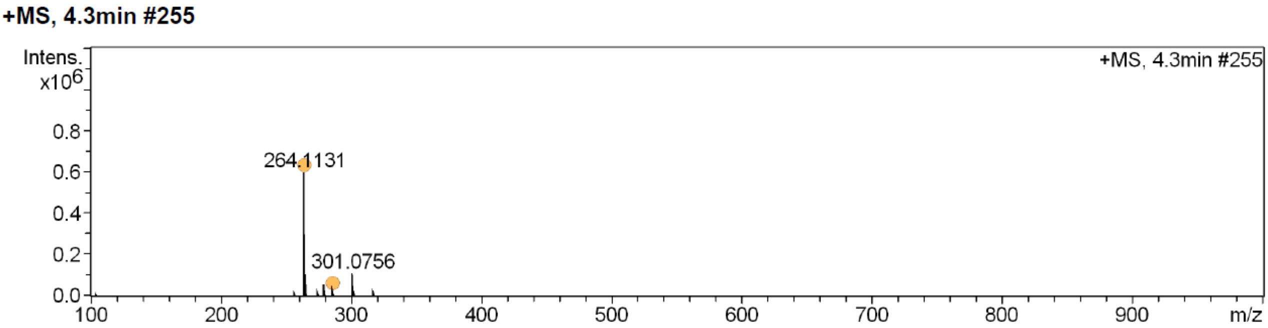

Meas. m/z	#	Ion Formula	m/z	err [ppm]	mSigma	# Sigma	Score	rdb	e⁻ Conf	N-Rule
264.1131	1	C16H14N3O	264.1131	-0.3	3.0	1	100.00	11.5	even	ok
286.0951	1	C16H13N3NaO	286.0951	0.0	10.7	1	100.00	11.5	even	ok

Figure S140. 1-[1-Phenyl-3-(pyridin-4-yl)-1*H*-pyrazol-4-yl]ethan-1-one (11b). HRMS (ESI-TOF).

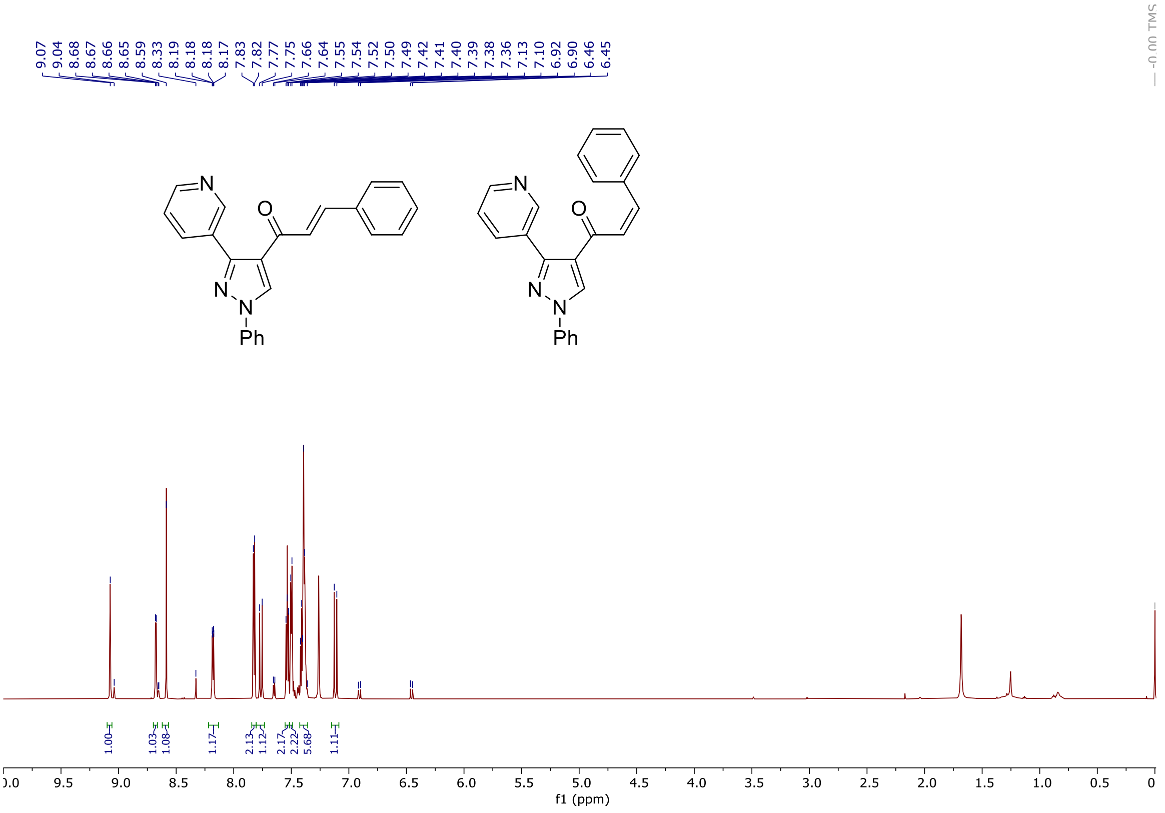

Figure S141. (2*E*/*Z*)-3-Phenyl-1-[1-phenyl-3-(pyridin-3-yl)-1*H*-pyrazol-4-yl]prop-2-en-1-one (*E*-12a, *Z*-13a). ^{1}H NMR spectrum (700 MHz, CDCl$_3$)

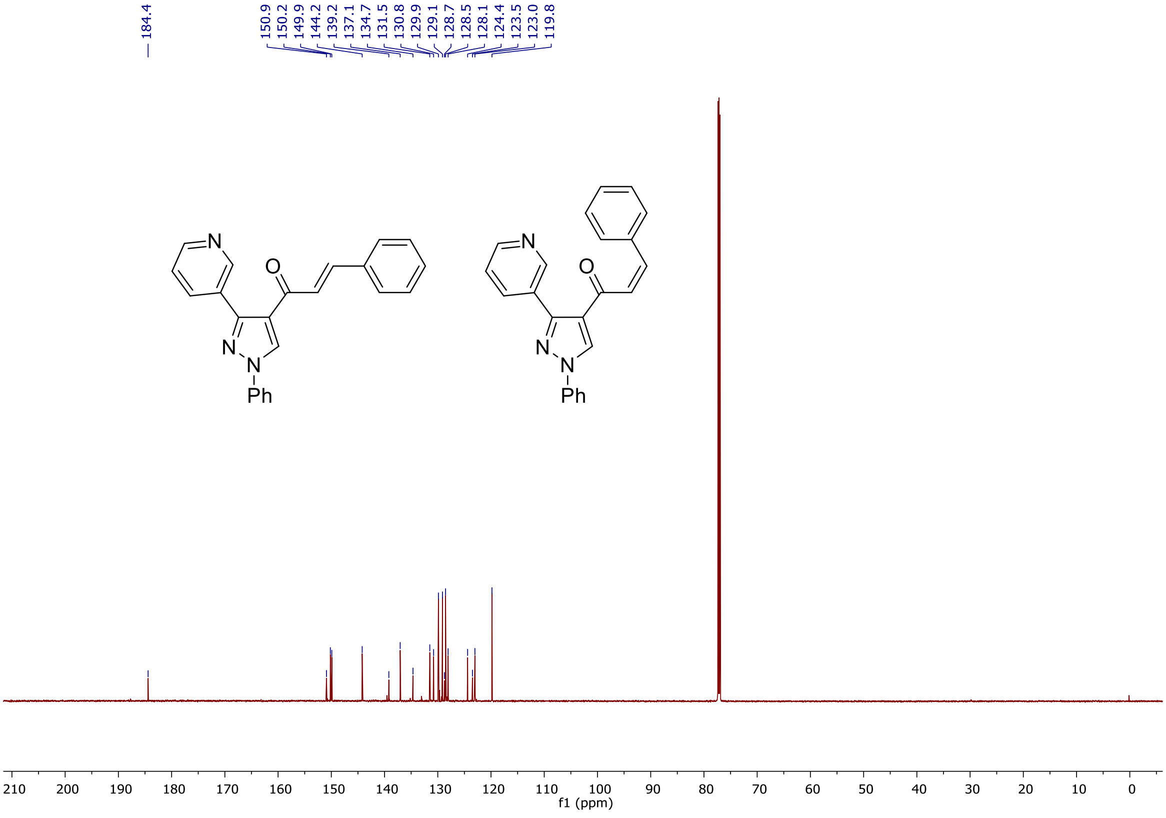

Figure S142. (2*E*/*Z*)-3-Phenyl-1-[1-phenyl-3-(pyridin-3-yl)-1*H*-pyrazol-4-yl]prop-2-en-1-one (*E*-12a, *Z*-13a). ^{13}C NMR spectrum (176 MHz, CDCl$_3$)

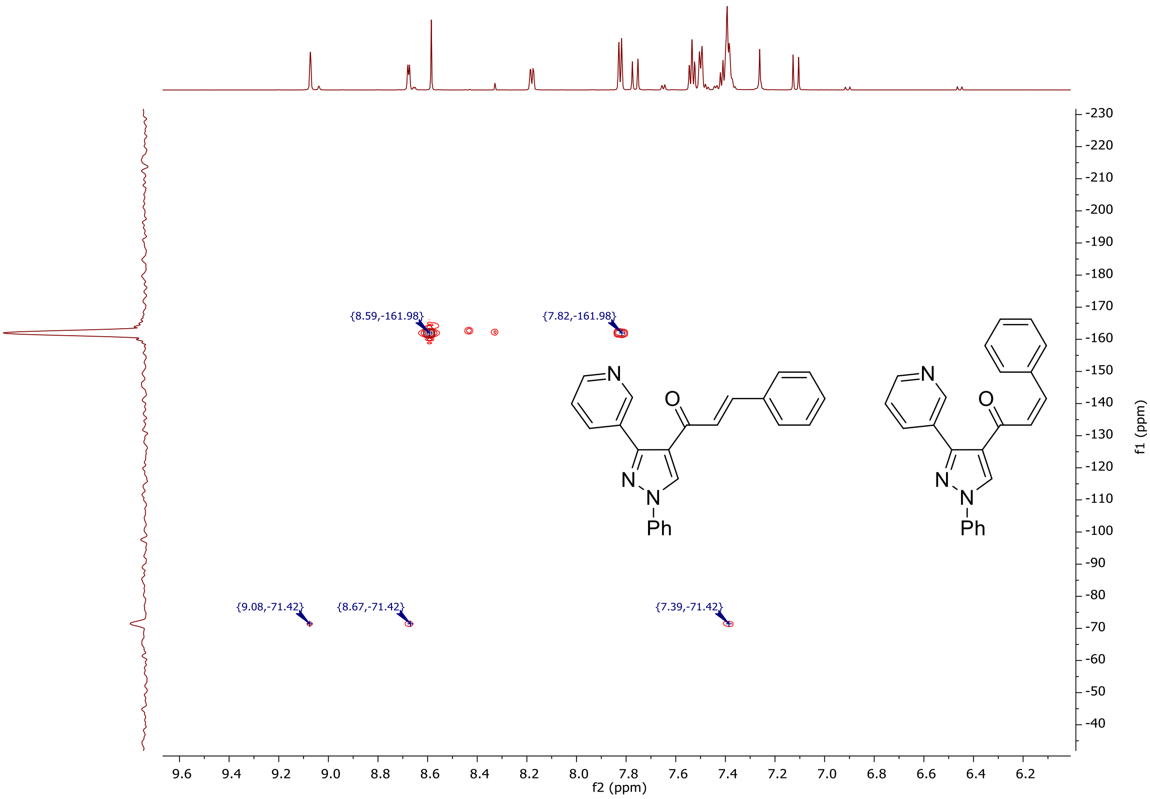

Figure S143. (2*E*/*Z*)-3-Phenyl-1-[1-phenyl-3-(pyridin-3-yl)-1*H*-pyrazol-4-yl]prop-2-en-1-one (*E*-12a, *Z*-13a). ^{1}H-^{15}N HMBC NMR spectrum (71 MHz, $CDCl_3$)

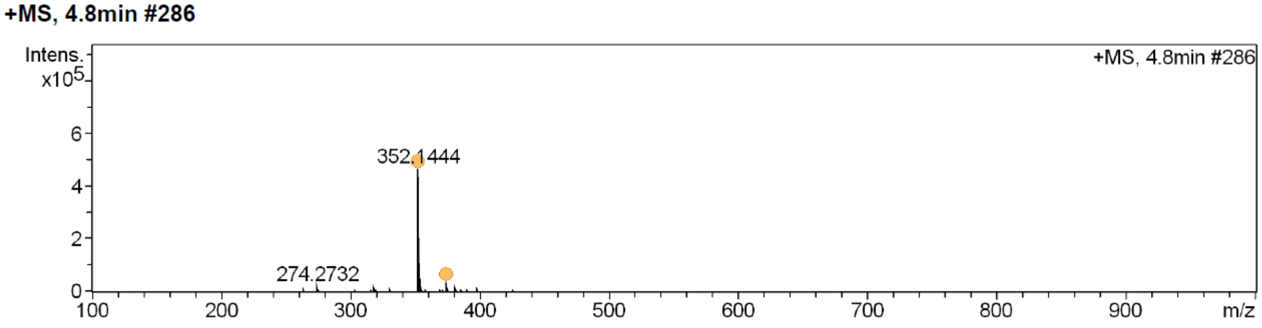

Meas. m/z	#	Ion Formula	m/z	err [ppm]	mSigma	# Sigma	Score	rdb	e⁻ Conf	N-Rule
352.1444	1	C23H18N3O	352.1444	0.0	5.8	1	100.00	16.5	even	ok
374.1261	1	C23H17N3NaO	374.1264	0.8	5.6	1	100.00	16.5	even	ok

Figure S144. (2*E*/*Z*)-3-Phenyl-1-[1-phenyl-3-(pyridin-3-yl)-1*H*-pyrazol-4-yl]prop-2-en-1-one (*E*-12a, *Z*-13a). HRMS (ESI-TOF).

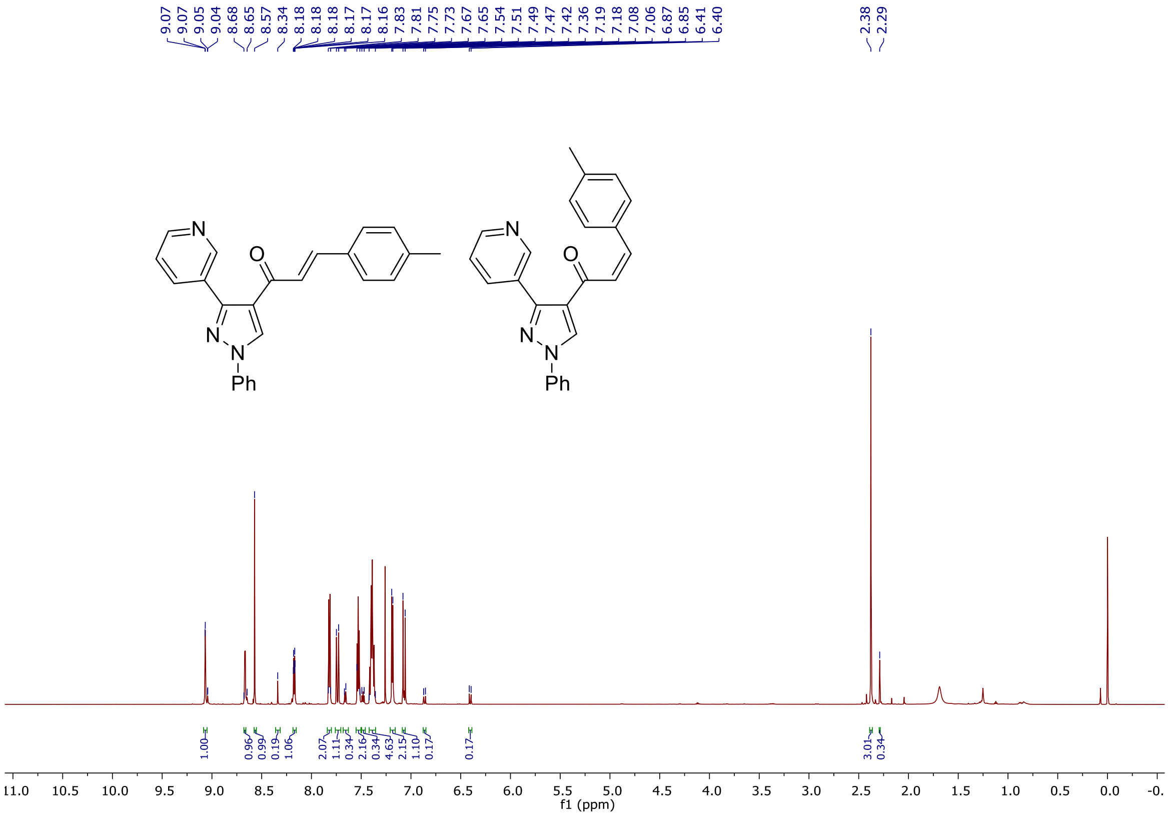

Figure S145. (2*E*/*Z*)-3-(4-Methylphenyl)-1-[1-phenyl-3-(pyridin-3-yl)-1*H*-Pyrazol-4-yl]prop-2-en-1-one (*E*-12b, *Z*-13b). ^{1}H NMR spectrum (700 MHz, CDCl$_3$)

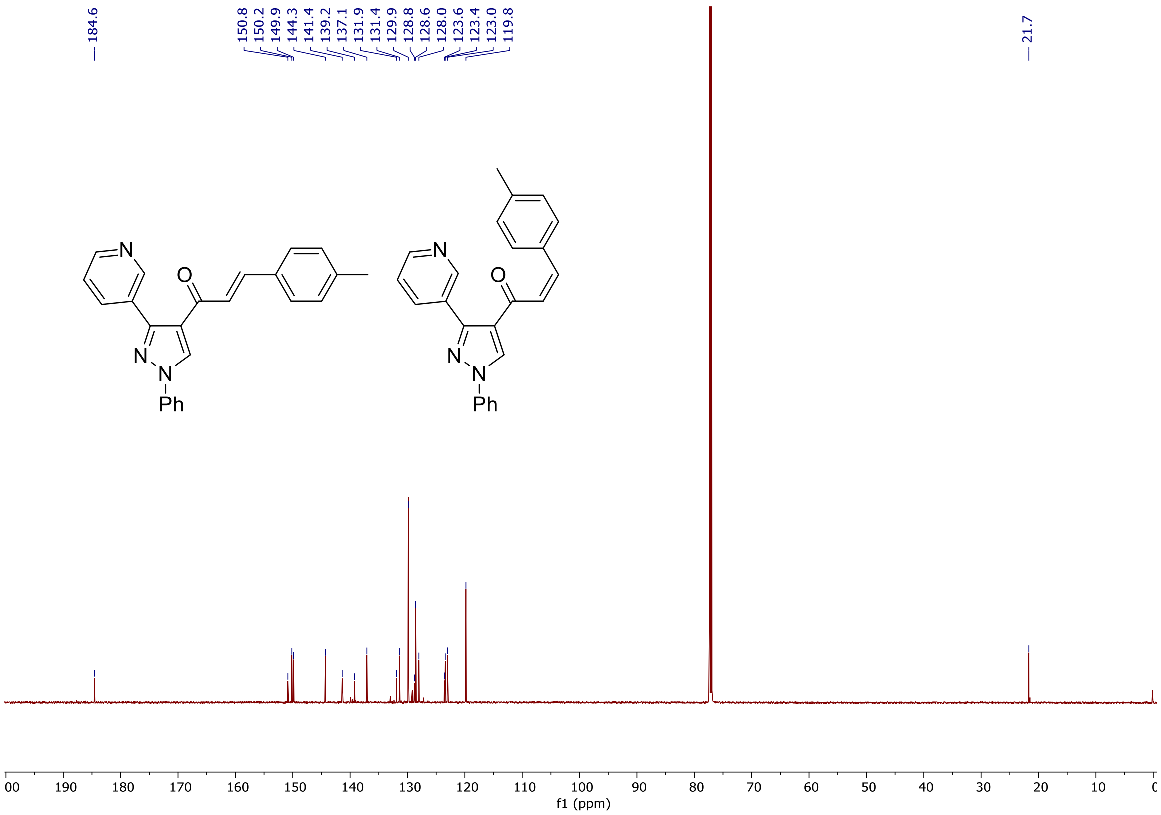

Figure S146. (2*E*/*Z*)-3-(4-Methylphenyl)-1-[1-phenyl-3-(pyridin-3-yl)-1*H*-Pyrazol-4-yl]prop-2-en-1-one (*E*-12b, *Z*-13b). ^{13}C NMR spectrum (176 MHz, CDCl$_3$)

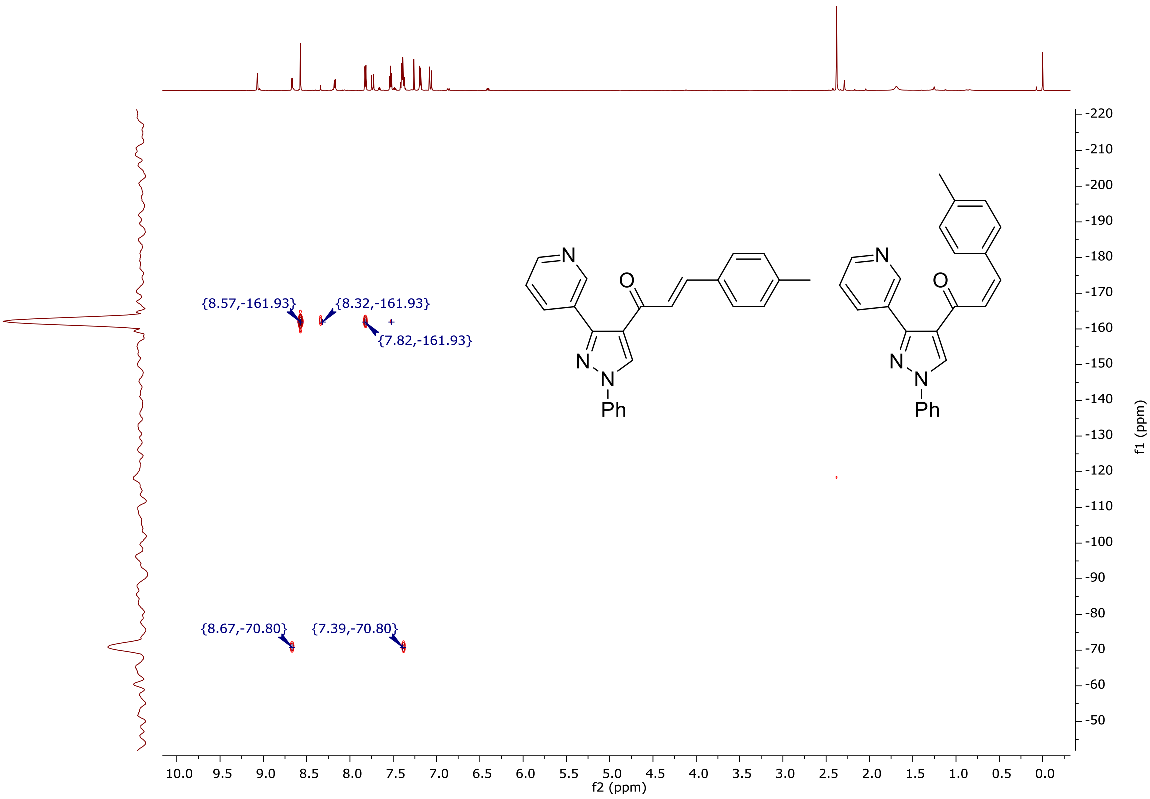

Figure S147. **(2*E*/*Z*)-3-(4-Methylphenyl)-1-[1-phenyl-3-(pyridin-3-yl)-1*H*-Pyrazol-4-yl]prop-2-en-1-one (*E*-12b, *Z*-13b). ^{1}H-^{15}N HMBC NMR spectrum (71 MHz, $CDCl_3$)**

+MS, 4.4min #263

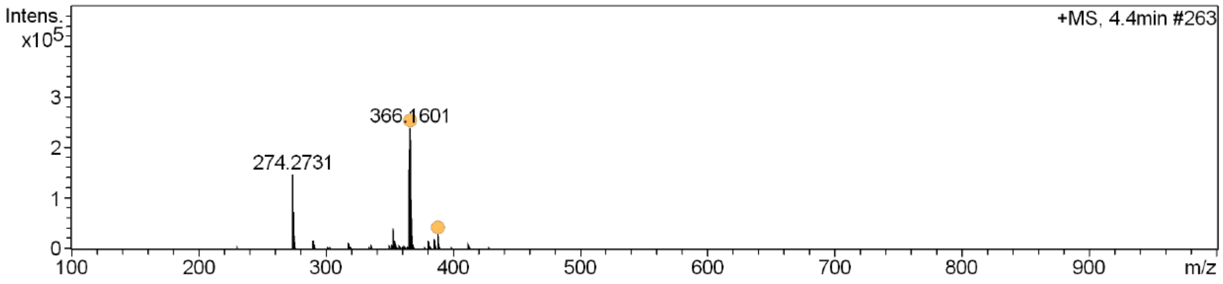

Meas. m/z	#	Ion Formula	m/z	err [ppm]	mSigma	# Sigma	Score	rdb	e⁻ Conf	N-Rule
366.1601	1	C24H20N3O	366.1601	0.1	5.9	1	100.00	16.5	even	ok
388.1433	1	C24H19N3NaO	388.1420	3.3	28.3	1	100.00	16.5	even	ok

Figure S148. **(2*E*/*Z*)-3-(4-Methylphenyl)-1-[1-phenyl-3-(pyridin-3-yl)-1*H*-Pyrazol-4-yl]prop-2-en-1-one (*E*-12b, *Z*-13b). HRMS (ESI-TOF).**

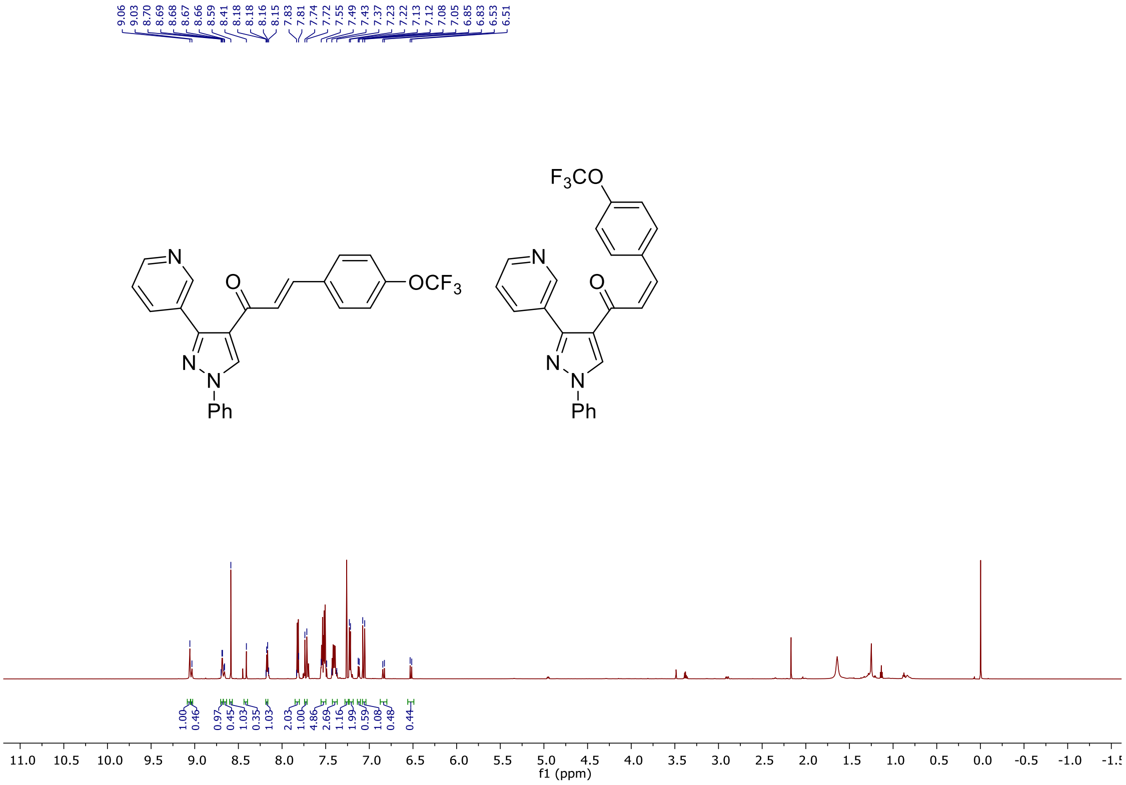

Figure S149. (2*E/Z*)-1-[1-Phenyl-3-(pyridine-3-yl)-1*H*-pyrazol-4-yl]-3-[4-(trifluoromethoxy)phenyl]prop-2-en-1-one (*E*-12c, *Z*-13c). ^{1}H NMR spectrum (700 MHz, CDCl$_3$)

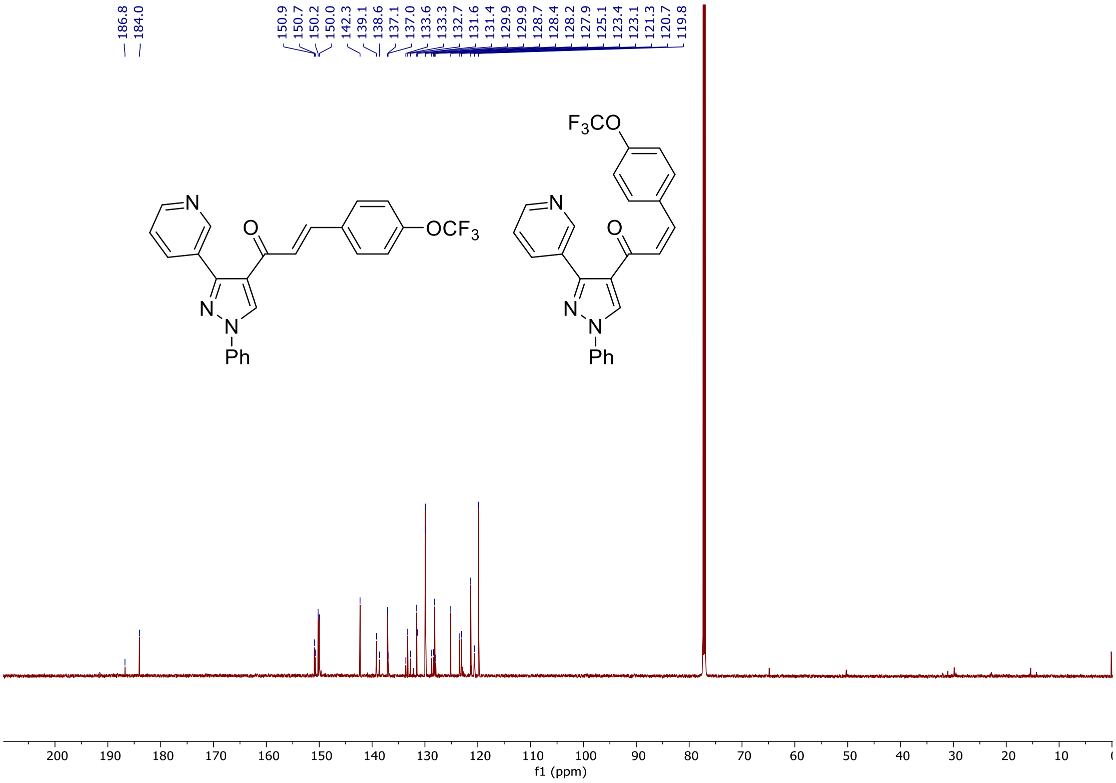

Figure S150. (2*E/Z*)-1-[1-Phenyl-3-(pyridine-3-yl)-1*H*-pyrazol-4-yl]-3-[4-(trifluoromethoxy)phenyl]prop-2-en-1-one (*E*-12c, *Z*-13c). ^{13}C NMR spectrum (176 MHz, CDCl$_3$)

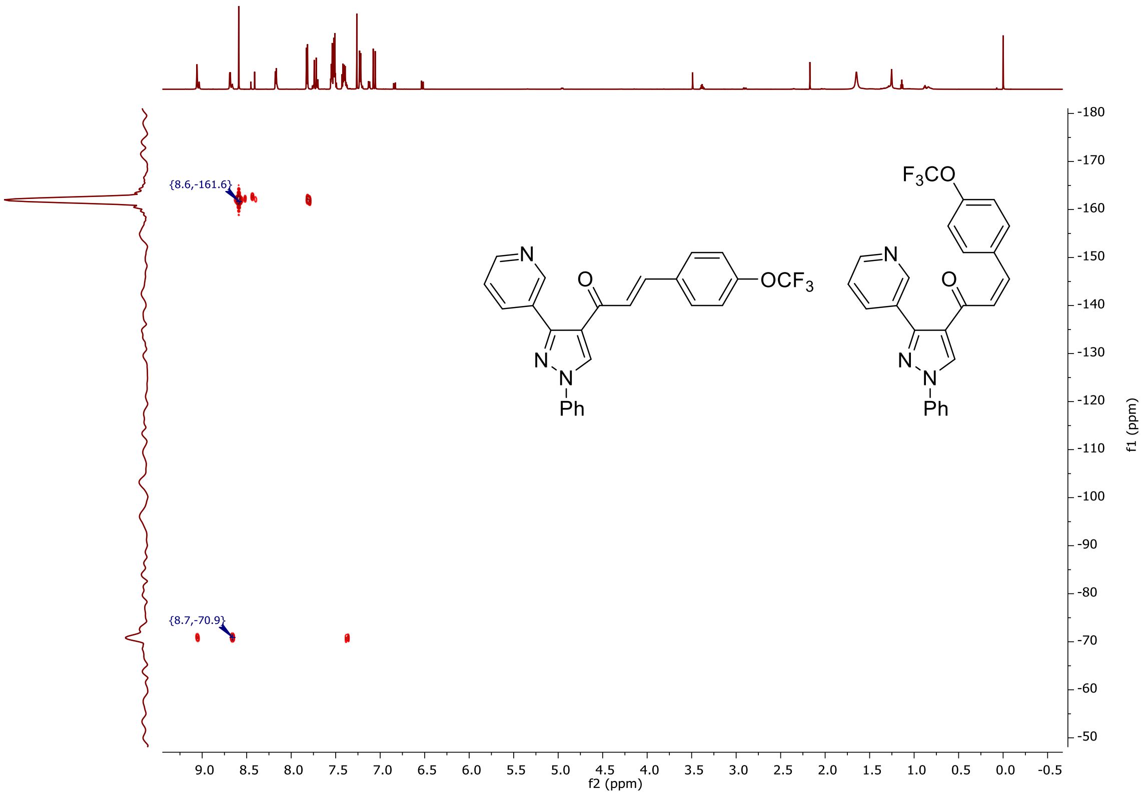

Figure S151. **(2*E*/*Z*)-1-[1-Phenyl-3-(pyridine-3-yl)-1*H*-pyrazol-4-yl]-3-[4-(trifluoromethoxy)phenyl]prop-2-en-1-one (*E*-12c, *Z*-13c). ^{1}H-^{15}N HMBC NMR spectrum (71 MHz, $CDCl_3$)**

+MS, 6.2min #374

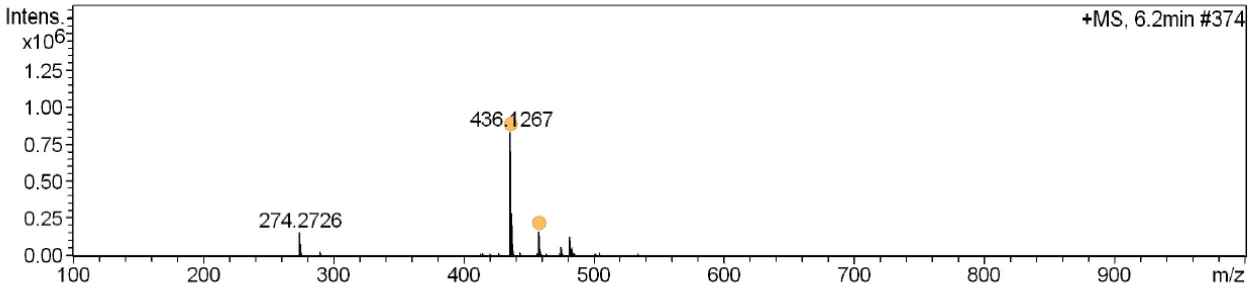

Meas. m/z	#	Ion Formula	m/z	err [ppm]	mSigma	# Sigma	Score	rdb	e⁻ Conf	N-Rule
436.1267	1	C24H17F3N3O2	436.1267	0.0	9.5	1	100.00	16.5	even	ok
458.1082	1	C24H16F3N3NaO2	458.1087	-1.0	15.2	1	100.00	16.5	even	ok

Figure S152. **(2*E*/*Z*)-1-[1-Phenyl-3-(pyridine-3-yl)-1*H*-pyrazol-4-yl]-3-[4-(trifluoromethoxy)phenyl]prop-2-en-1-one (*E*-12c, *Z*-13c). HRMS (ESI-TOF).**

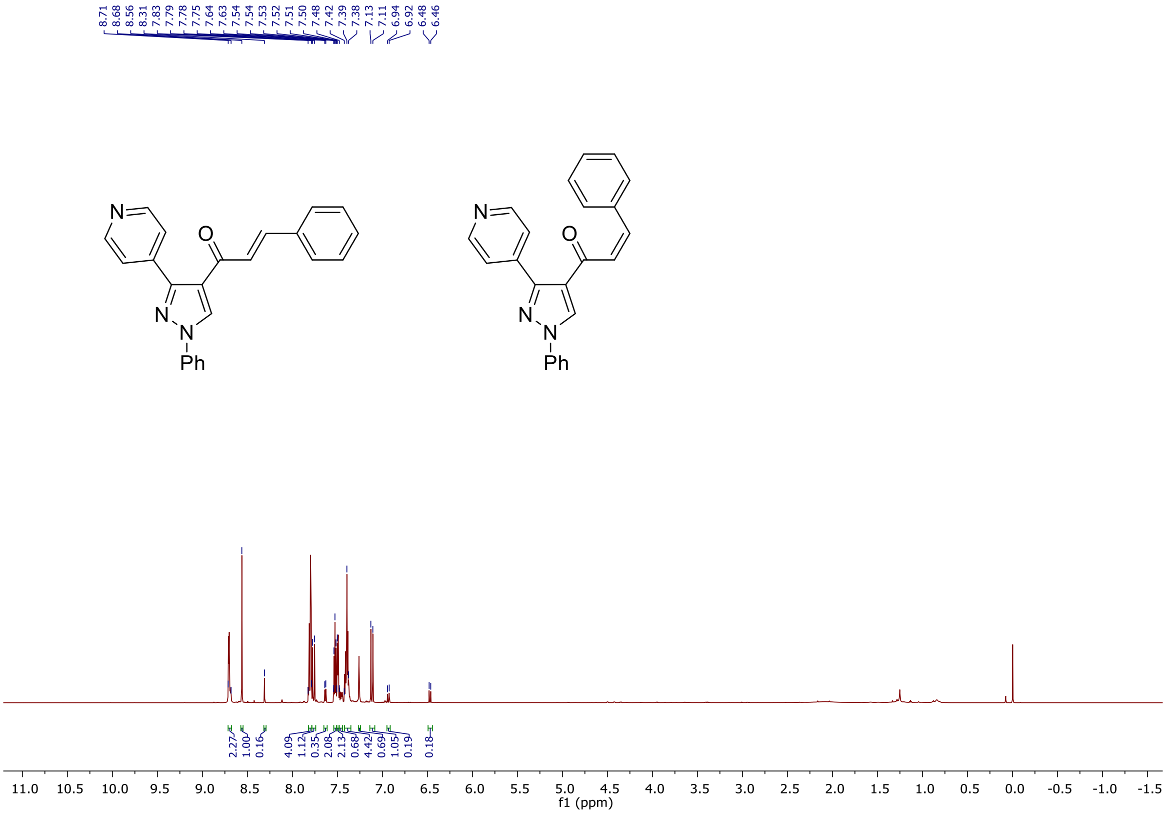

Figure S153. (2*E/Z*)-3-Phenyl-1-[1-phenyl-3-(pyridin-4-yl)-1*H*-pyrazol-4-yl]prop-2-en-1-one (*E*-12d, *Z*-13d). ^{1}H NMR spectrum (700 MHz, $CDCl_3$)

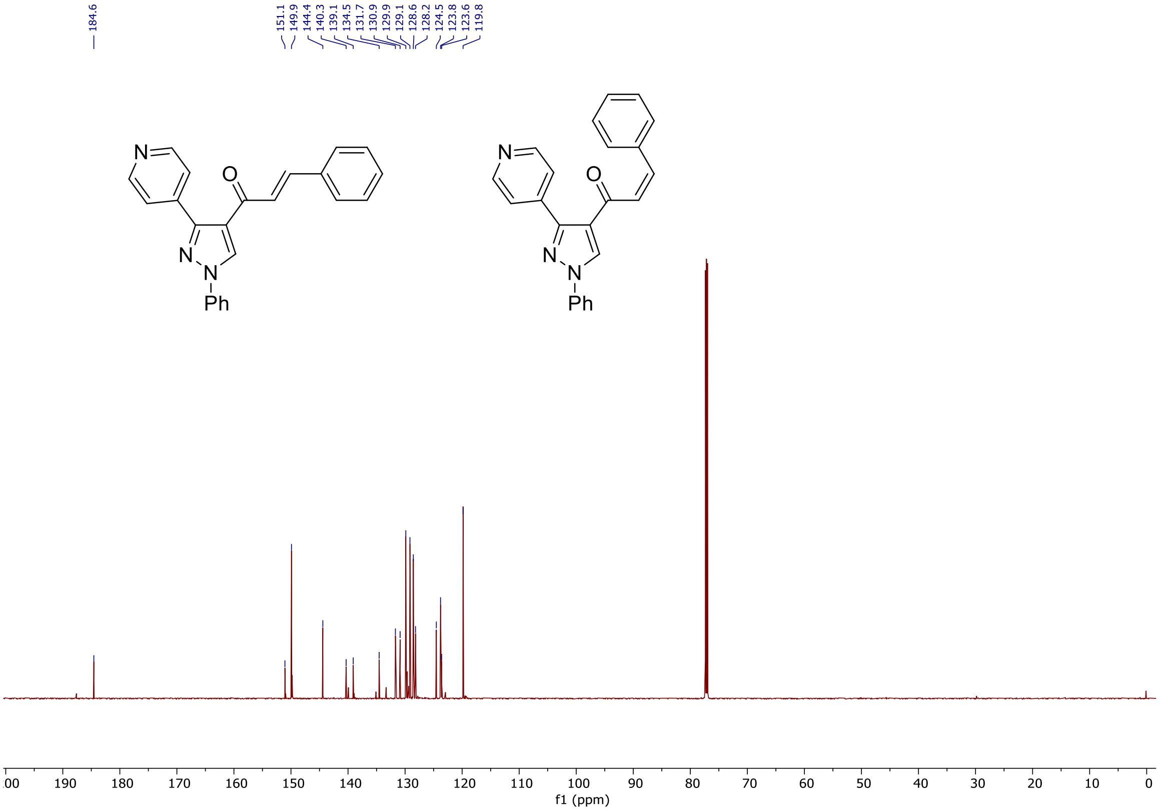

Figure S154. (2*E/Z*)-3-Phenyl-1-[1-phenyl-3-(pyridin-4-yl)-1*H*-pyrazol-4-yl]prop-2-en-1-one (*E*-12d, *Z*-13d). ^{13}C NMR spectrum (176 MHz, $CDCl_3$)

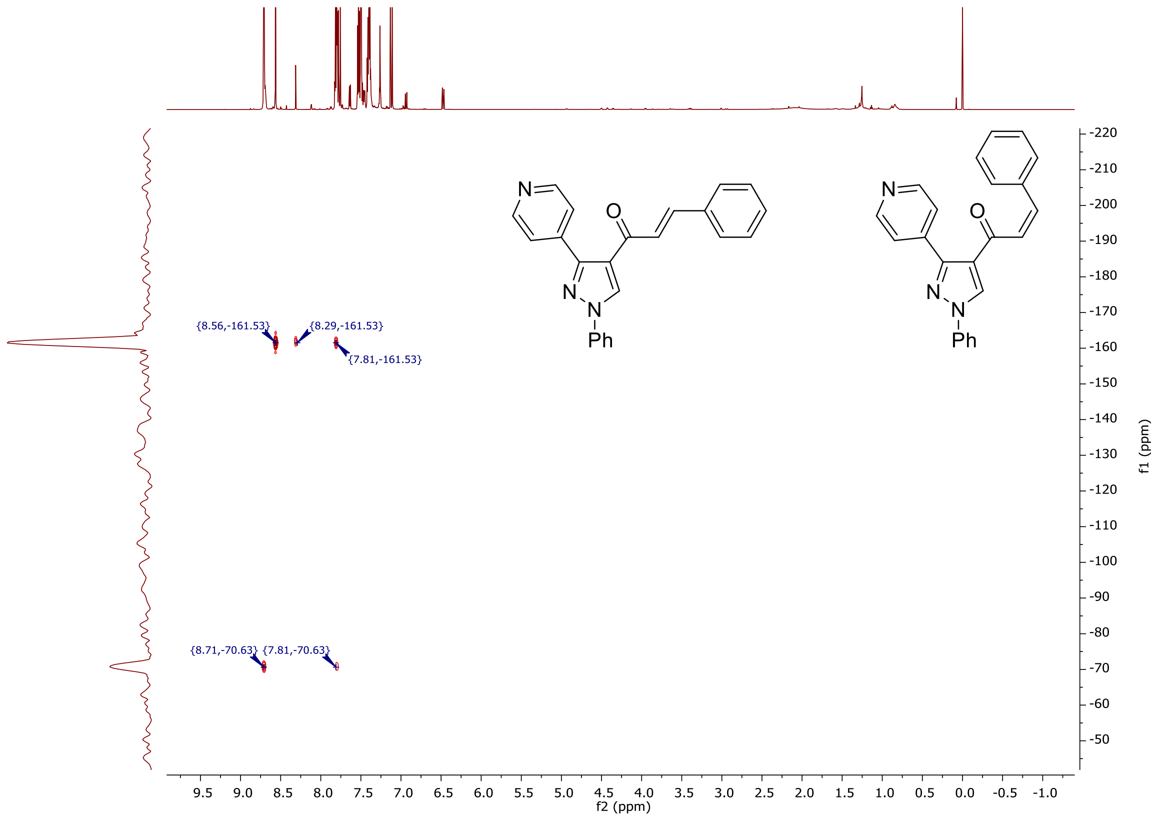

Figure S155. (2*E*/*Z*)-3-Phenyl-1-[1-phenyl-3-(pyridin-4-yl)-1*H*-pyrazol-4-yl]prop-2-en-1-one (*E*-12d, *Z*-13d). ^{1}H-^{15}N HMBC NMR spectrum (71 MHz, $CDCl_3$)

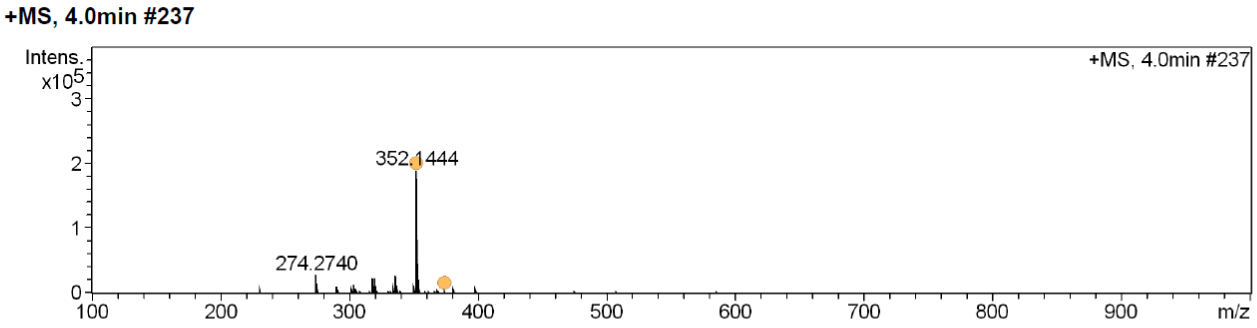

Meas. m/z	#	Ion Formula	m/z	err [ppm]	mSigma	# Sigma	Score	rdb	e⁻ Conf	N-Rule
352.1444	1	C23H18N3O	352.1444	-0.1	2.5	1	100.00	16.5	even	ok
374.1266	1	C23H17N3NaO	374.1264	-0.6	18.1	1	100.00	16.5	even	ok

Figure S156. (2*E*/*Z*)-3-Phenyl-1-[1-phenyl-3-(pyridin-4-yl)-1*H*-pyrazol-4-yl]prop-2-en-1-one (*E*-12d, *Z*-13d). HRMS (ESI-TOF).

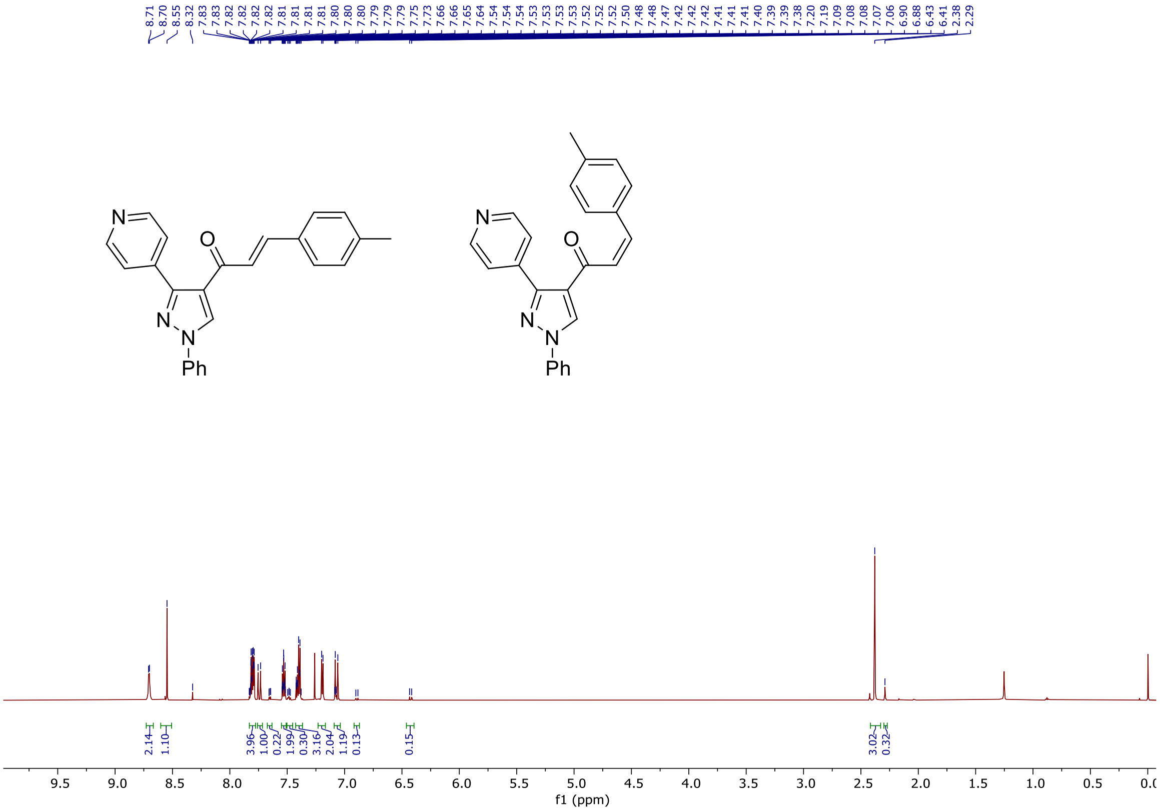

Figure S157. (2*E*/*Z*)-3-(4-Methylphenyl)-1-[1-phenyl-3-(pyridine-4-yl)-1*H*-pyrazol-4-yl]prop-2-en-1-one (*E*-12e, *Z*-13e). ^{1}H NMR spectrum (700 MHz, $CDCl_3$)

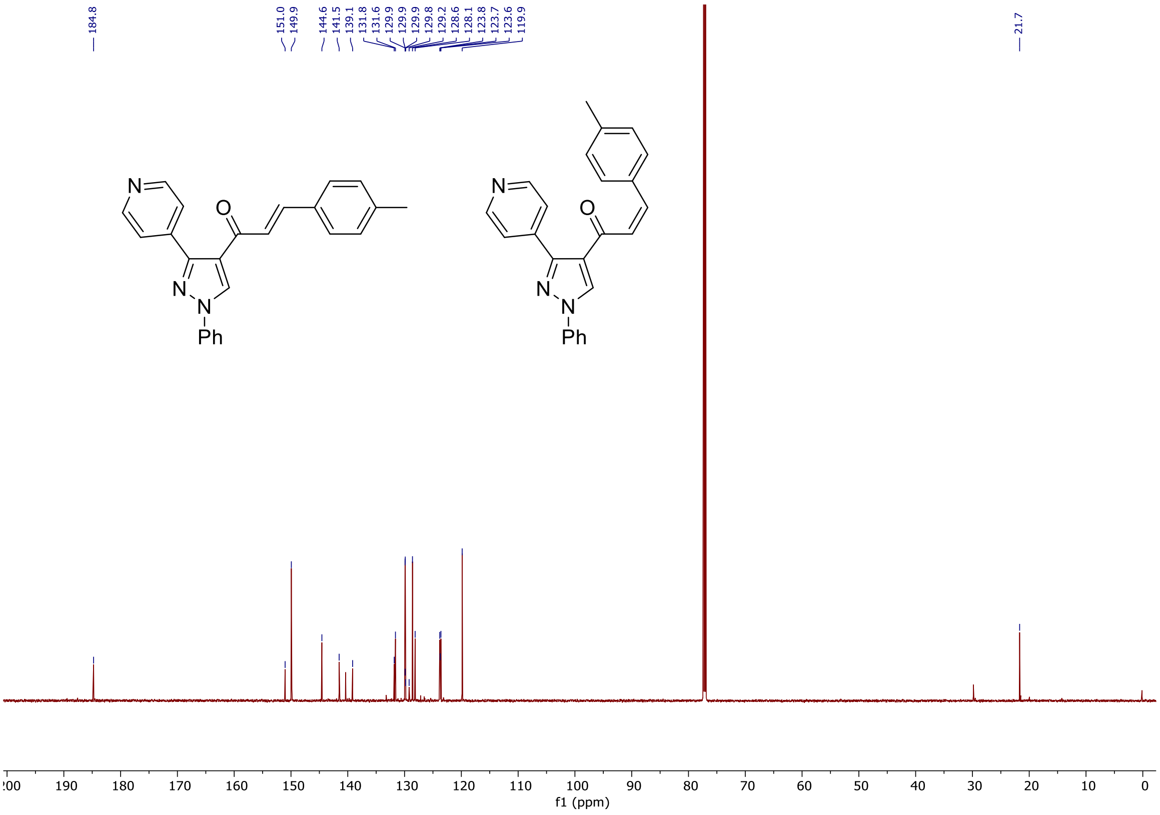

Figure S158. (2*E*/*Z*)-3-(4-Methylphenyl)-1-[1-phenyl-3-(pyridine-4-yl)-1*H*-pyrazol-4-yl]prop-2-en-1-one (*E*-12e, *Z*-13e). ^{13}C NMR spectrum (176 MHz, $CDCl_3$)

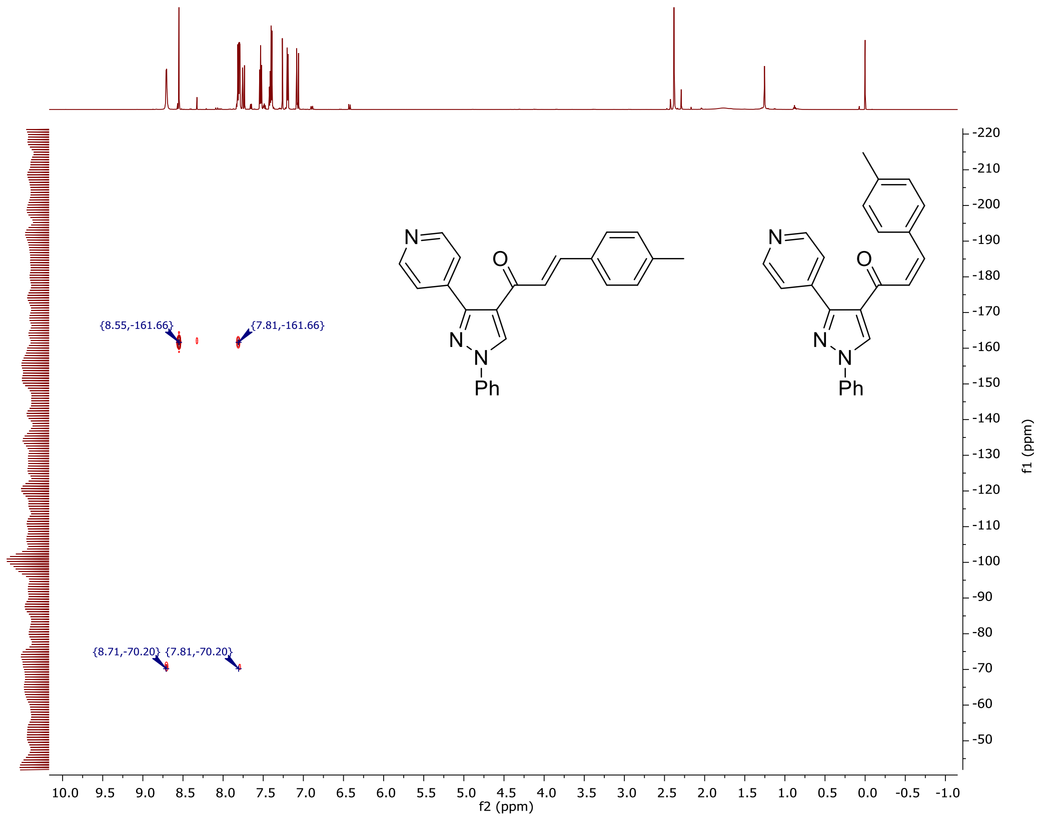

Figure S159. **(2*E*/*Z*)-3-(4-Methylphenyl)-1-[1-phenyl-3-(pyridine-4-yl)-1*H*-pyrazol-4-yl]prop-2-en-1-one (*E*-12e, *Z*-13e). ^{1}H-^{15}N HMBC NMR spectrum (71 MHz, $CDCl_3$)**

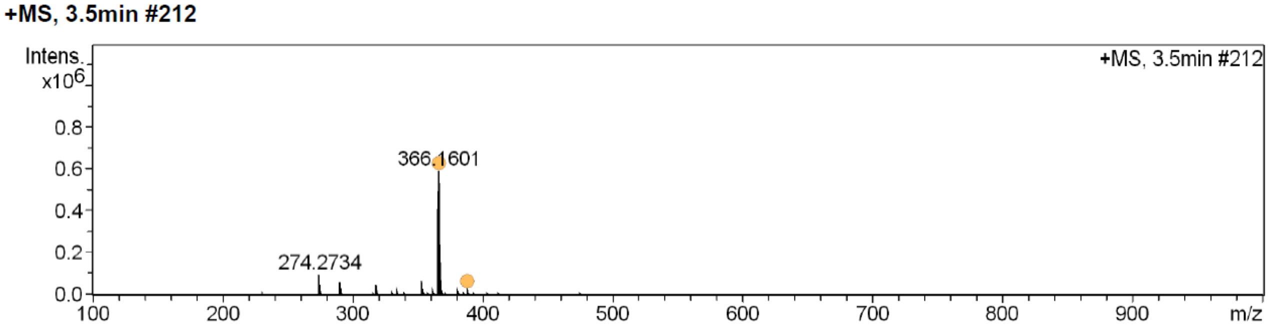

Meas. m/z	#	Ion Formula	m/z	err [ppm]	mSigma	# Sigma	Score	rdb	e⁻ Conf	N-Rule
366.1601	1	C24H20N3O	366.1601	-0.0	9.5	1	100.00	16.5	even	ok
388.1422	1	C24H19N3NaO	388.1420	-0.4	2.7	1	100.00	16.5	even	ok

Figure S160. **(2*E*/*Z*)-3-(4-Methylphenyl)-1-[1-phenyl-3-(pyridine-4-yl)-1*H*-pyrazol-4-yl]prop-2-en-1-one (*E*-12e, *Z*-13e). HRMS (ESI-TOF).**

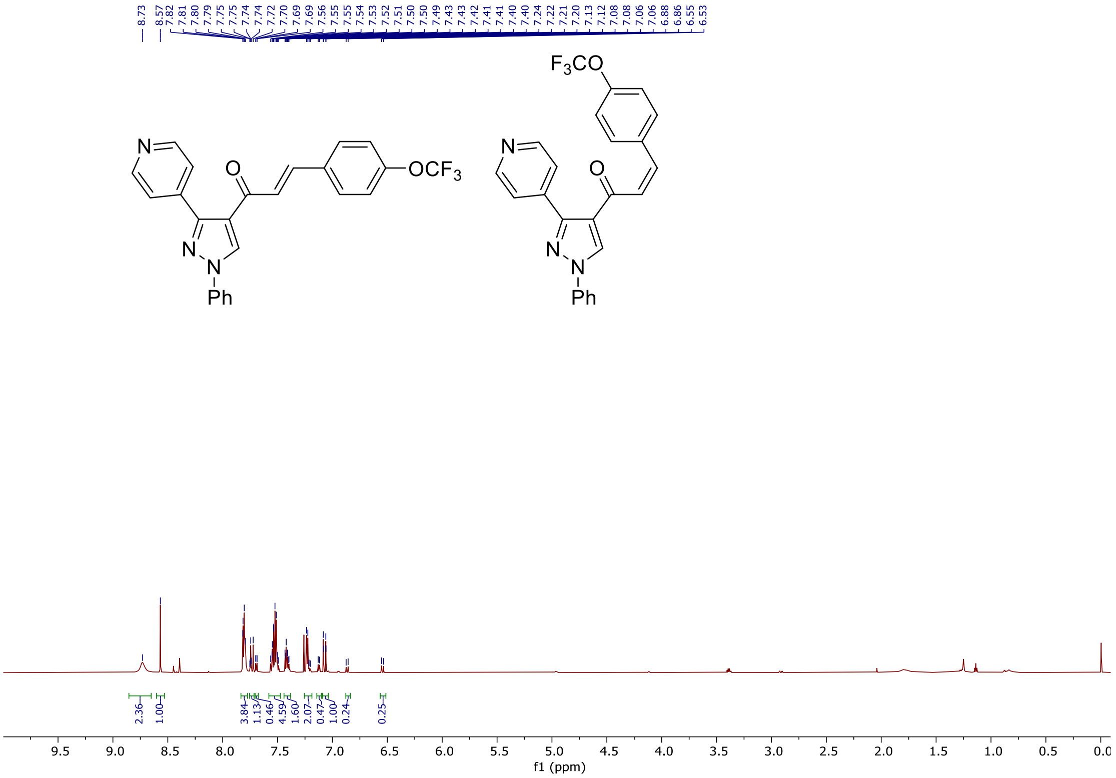

Figure S161. (2*E*/*Z*)-1-[1-Phenyl-3-(pyridine-4-yl)-1*H*-pyrazol-4-yl]-3-[4-(trifluoromethoxy)phenyl]prop-2-en-1-one spectrum (*E*-12f, *Z*-13f). ^{1}H NMR spectrum (700 MHz, $CDCl_3$)

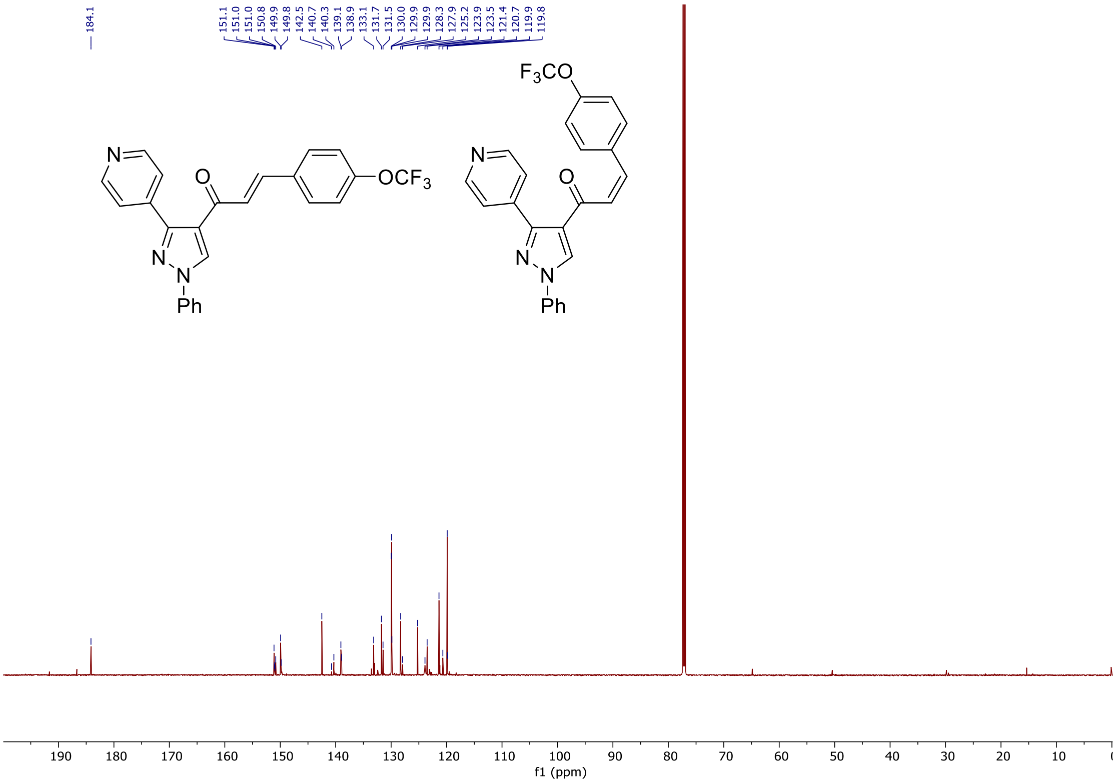

Figure S162. (2*E*/*Z*)-1-[1-Phenyl-3-(pyridine-4-yl)-1*H*-pyrazol-4-yl]-3-[4-(trifluoromethoxy)phenyl]prop-2-en-1-one spectrum (*E*-12f, *Z*-13f). ^{13}C NMR spectrum (176 MHz, $CDCl_3$)

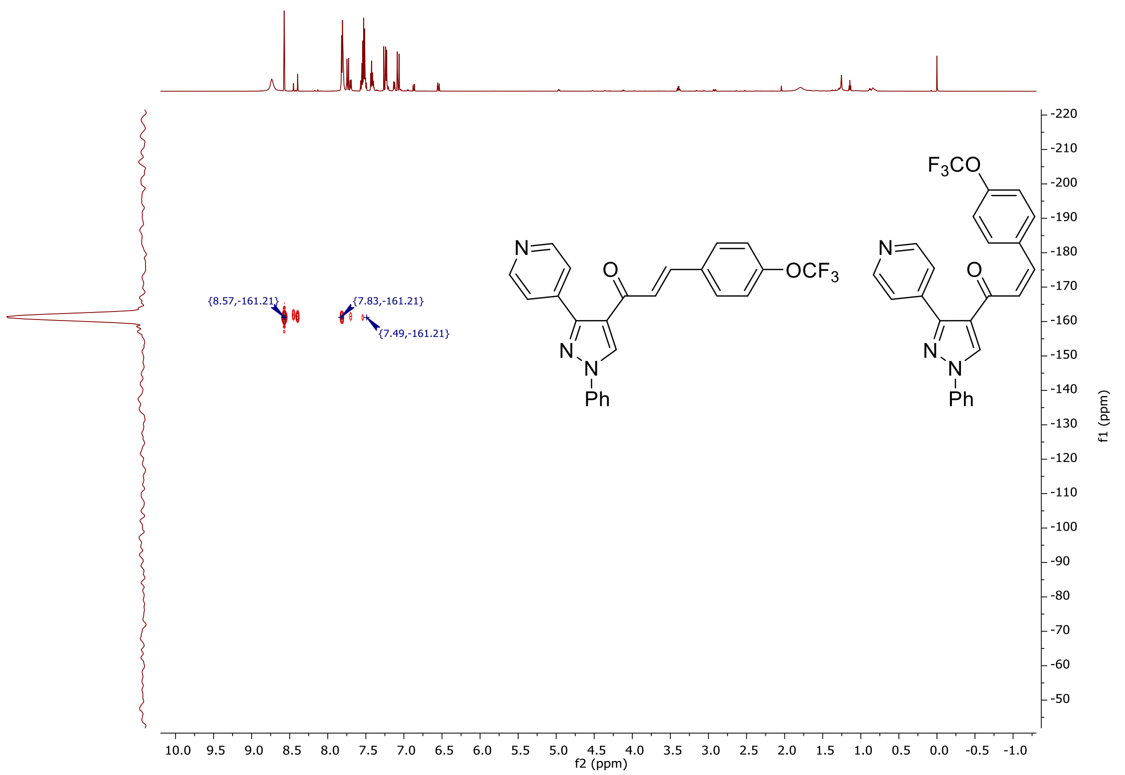

Figure S163. (2*E*/*Z*)-1-[1-Phenyl-3-(pyridine-4-yl)-1*H*-pyrazol-4-yl]-3-[4-(trifluoromethoxy)phenyl]prop-2-en-1-one (*E*-12f, *Z*-13f). ^{1}H-^{15}N HMBC NMR spectrum (71 MHz, $CDCl_3$)

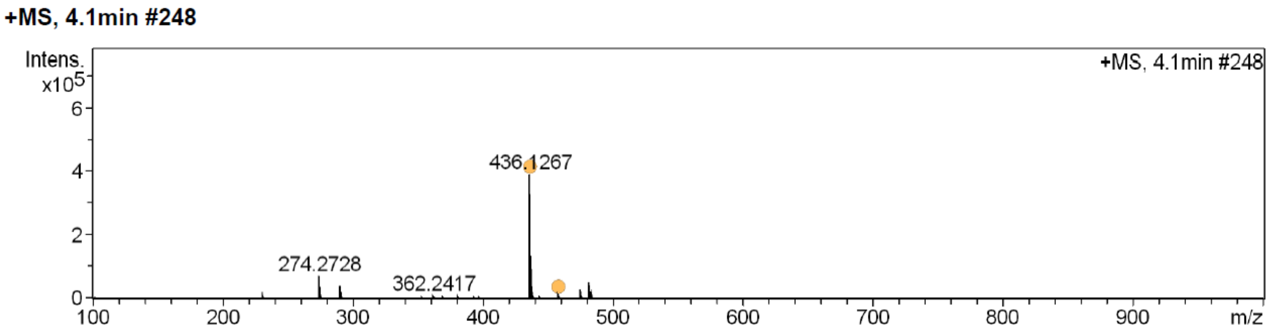

Meas. m/z	#	Ion Formula	m/z	err [ppm]	mSigma	# Sigma	Score	rdb	e⁻ Conf	N-Rule
436.1267	1	C24H17F3N3O2	436.1267	0.0	12.5	1	100.00	16.5	even	ok
458.1083	1	C24H16F3N3NaO2	458.1087	-0.9	35.7	1	100.00	16.5	even	ok

Figure S164. (2*E*/*Z*)-1-[1-Phenyl-3-(pyridine-4-yl)-1*H*-pyrazol-4-yl]-3-[4-(trifluoromethoxy)phenyl]prop-2-en-1-one (*E*-12f, *Z*-13f). HRMS (ESI-TOF).

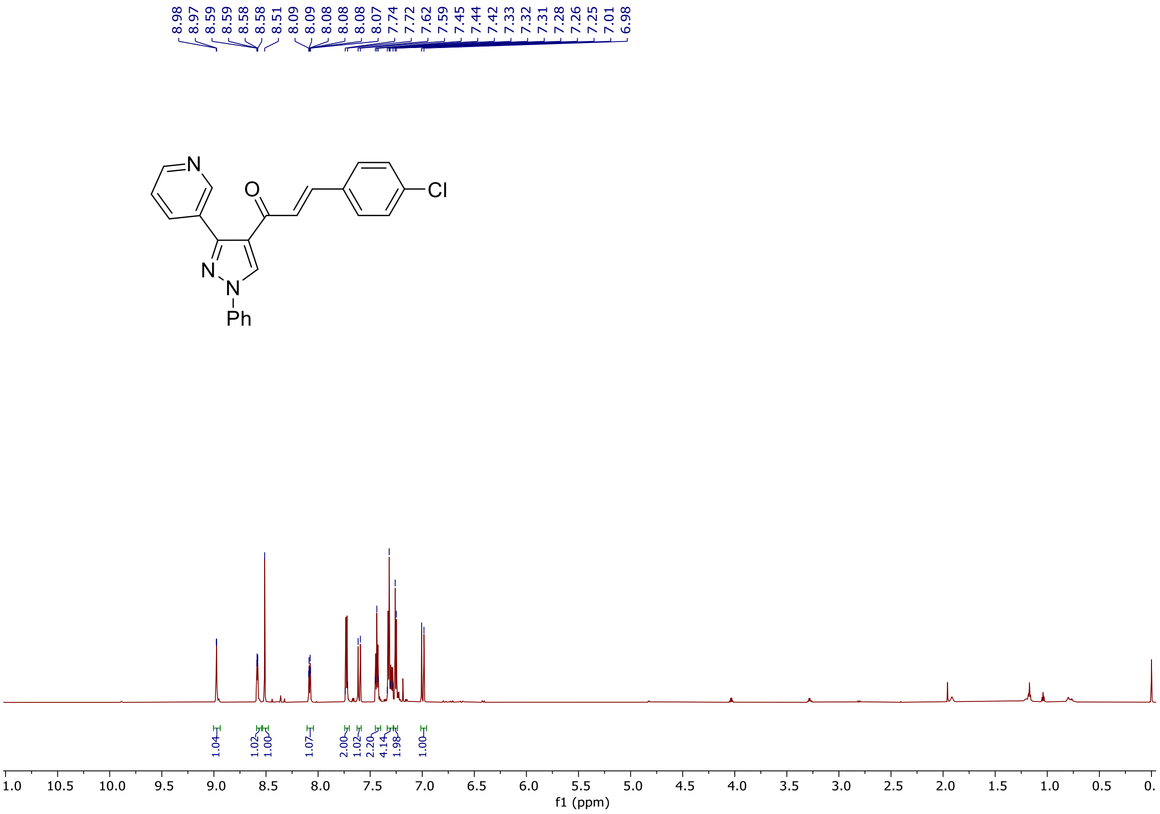

Figure S165. (2*E*)-3-(4-Chlorophenyl)-1-[1-phenyl-3-(pyridin-3-yl)-1*H*-pyrazol-4-yl]prop-2-en-1-one (12g). ^{1}H NMR spectrum (700 MHz, CDCl$_3$)

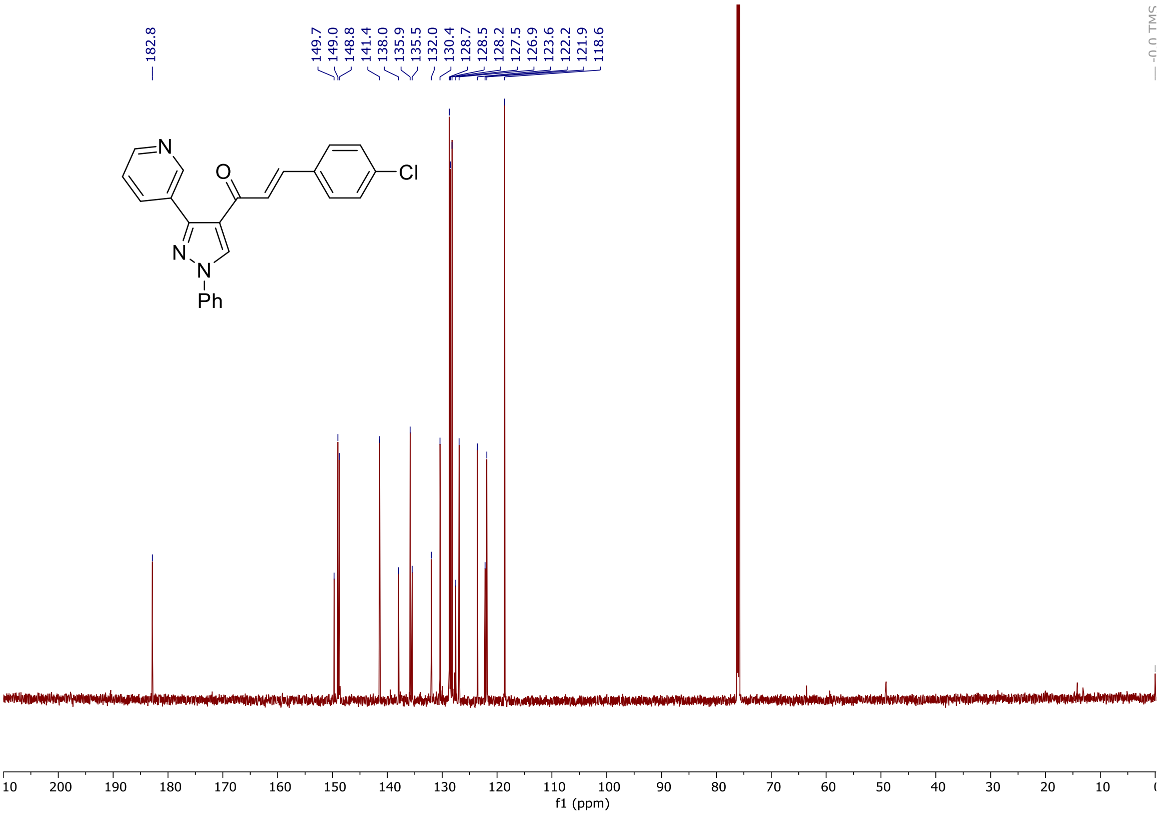

Figure S166. (2*E*)-3-(4-Chlorophenyl)-1-[1-phenyl-3-(pyridin-3-yl)-1*H*-pyrazol-4-yl]prop-2-en-1-one (12g). ^{13}C NMR spectrum (176 MHz, CDCl$_3$)

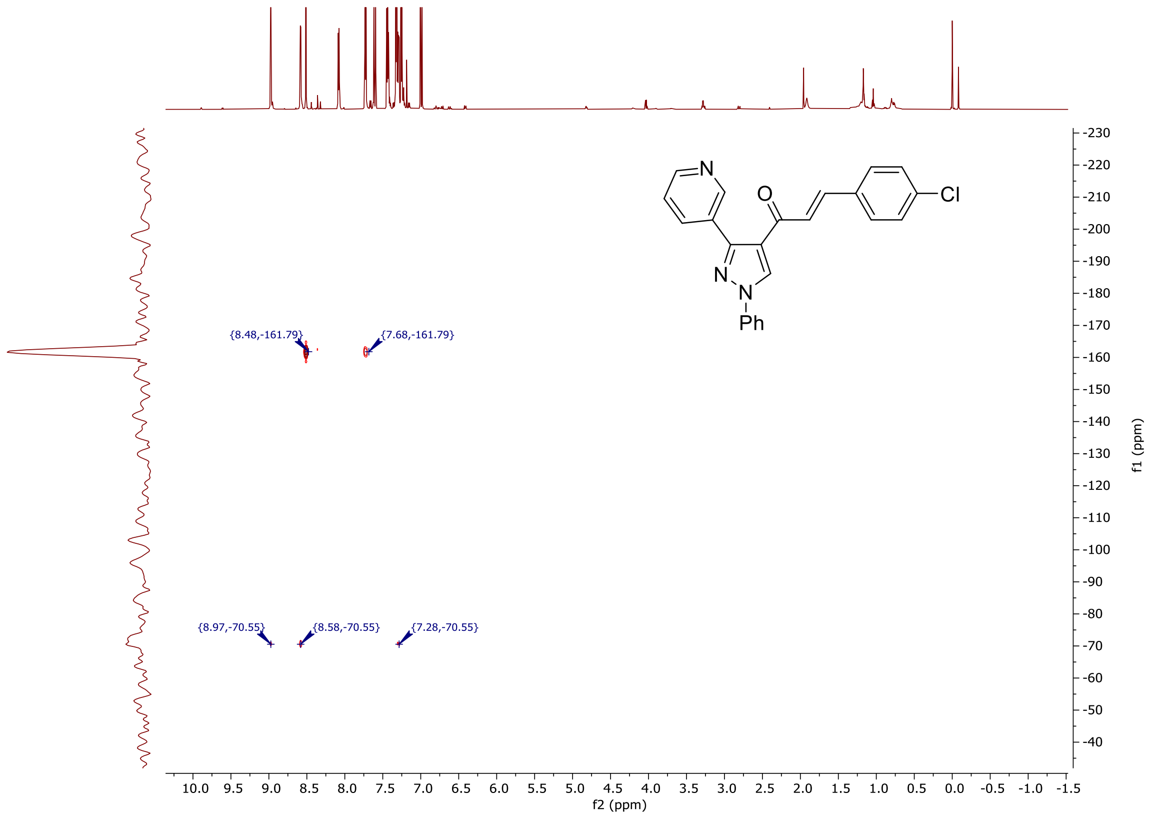

Figure S167. (2*E*)-3-(4-Chlorophenyl)-1-[1-phenyl-3-(pyridin-3-yl)-1*H*-pyrazol-4-yl]prop-2-en-1-one (12g). ^{1}H-^{15}N HMBC NMR spectrum (71 MHz, $CDCl_3$)

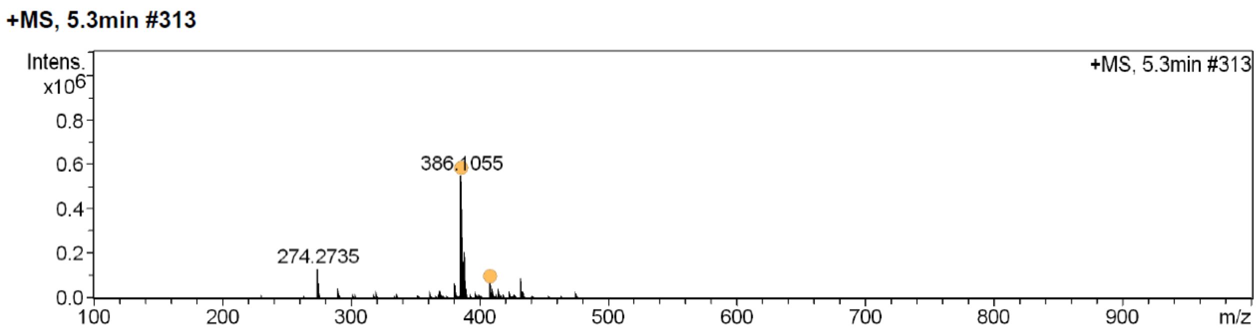

Meas. m/z	#	Ion Formula	m/z	err [ppm]	mSigma	# Sigma	Score	rdb	e⁻ Conf	N-Rule
386.1055	1	C23H17ClN3O	386.1055	-0.1	14.7	1	100.00	16.5	even	ok
408.0874	1	C23H16ClN3NaO	408.0874	0.1	52.5	1	100.00	16.5	even	ok

Figure S168. (2*E*)-3-(4-Chlorophenyl)-1-[1-phenyl-3-(pyridin-3-yl)-1*H*-pyrazol-4-yl]prop-2-en-1-one (12g). HRMS (ESI-TOF).

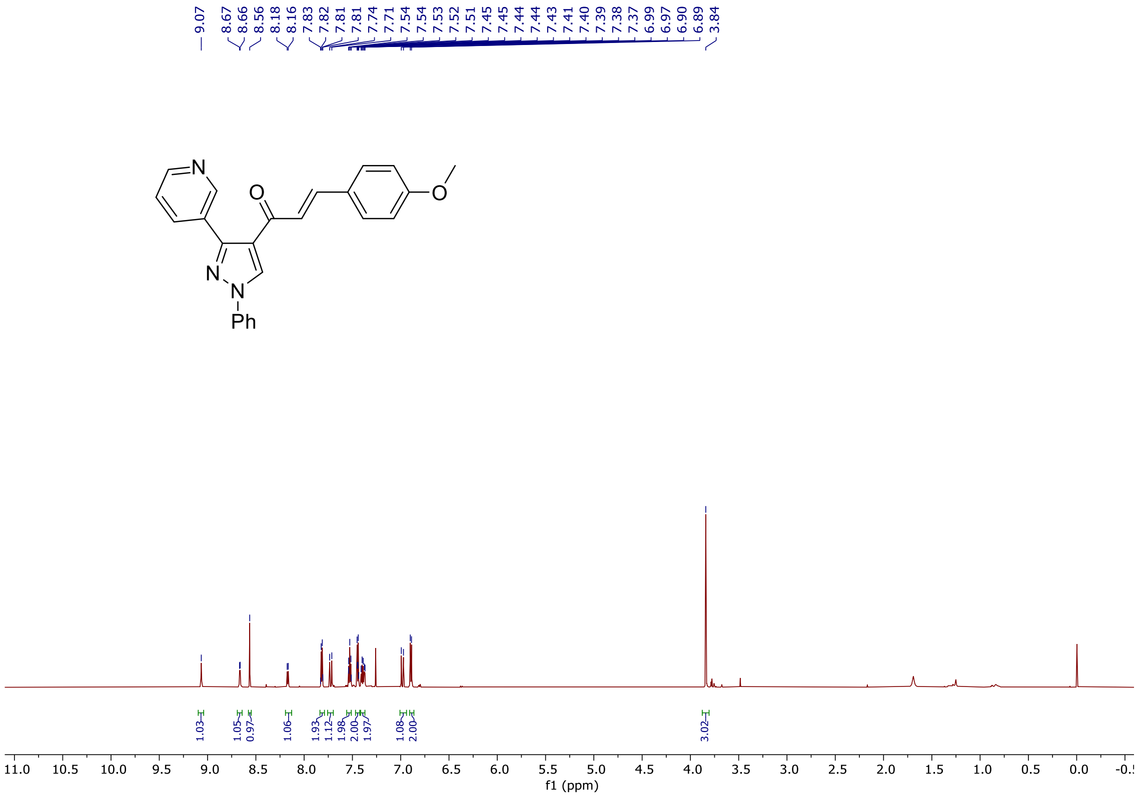

Figure S169. (2*E*)-3-(4-Methoxyphenyl)-1-[1-phenyl-3-(pyridin-3-yl)-1*H*-pyrazol-4-yl]prop-2-en-1-one (12h). ^{1}H NMR spectrum (700 MHz, $CDCl_3$)

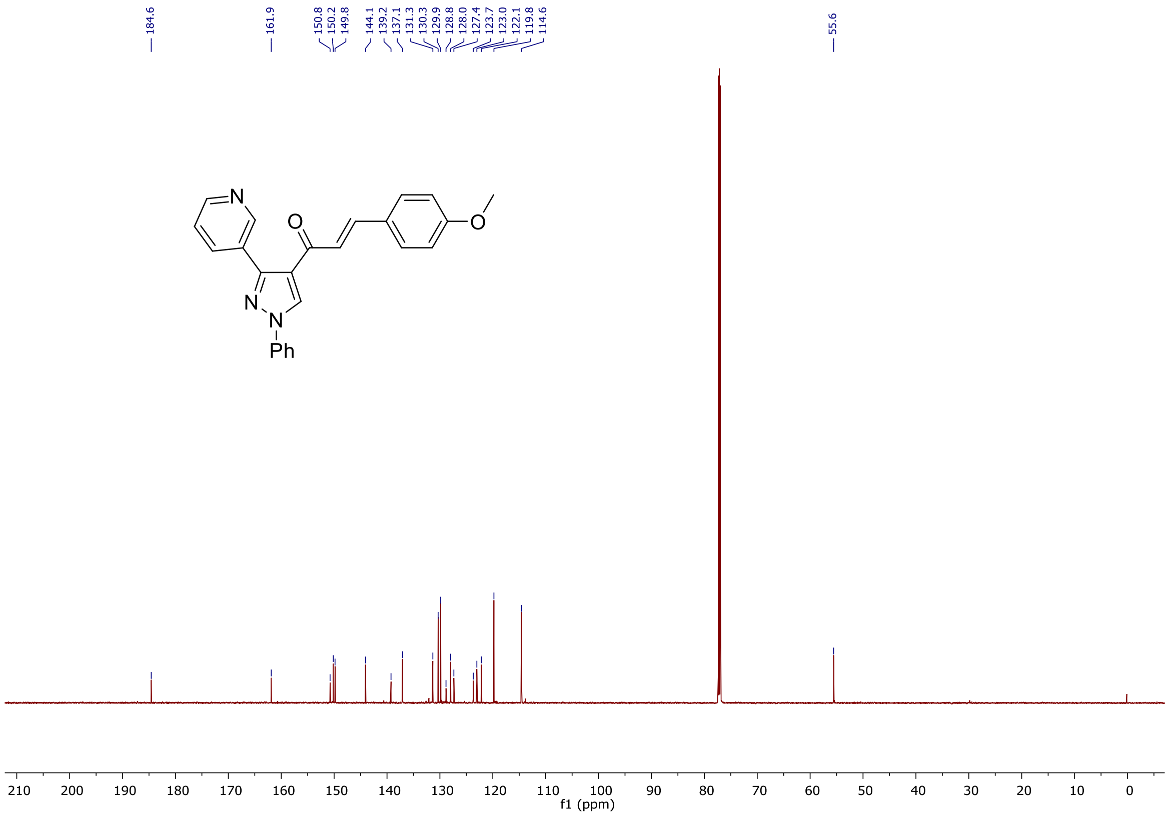

Figure S170. (2*E*)-3-(4-Methoxyphenyl)-1-[1-phenyl-3-(pyridin-3-yl)-1*H*-pyrazol-4-yl]prop-2-en-1-one (12h). ^{13}C NMR spectrum (176 MHz, $CDCl_3$)

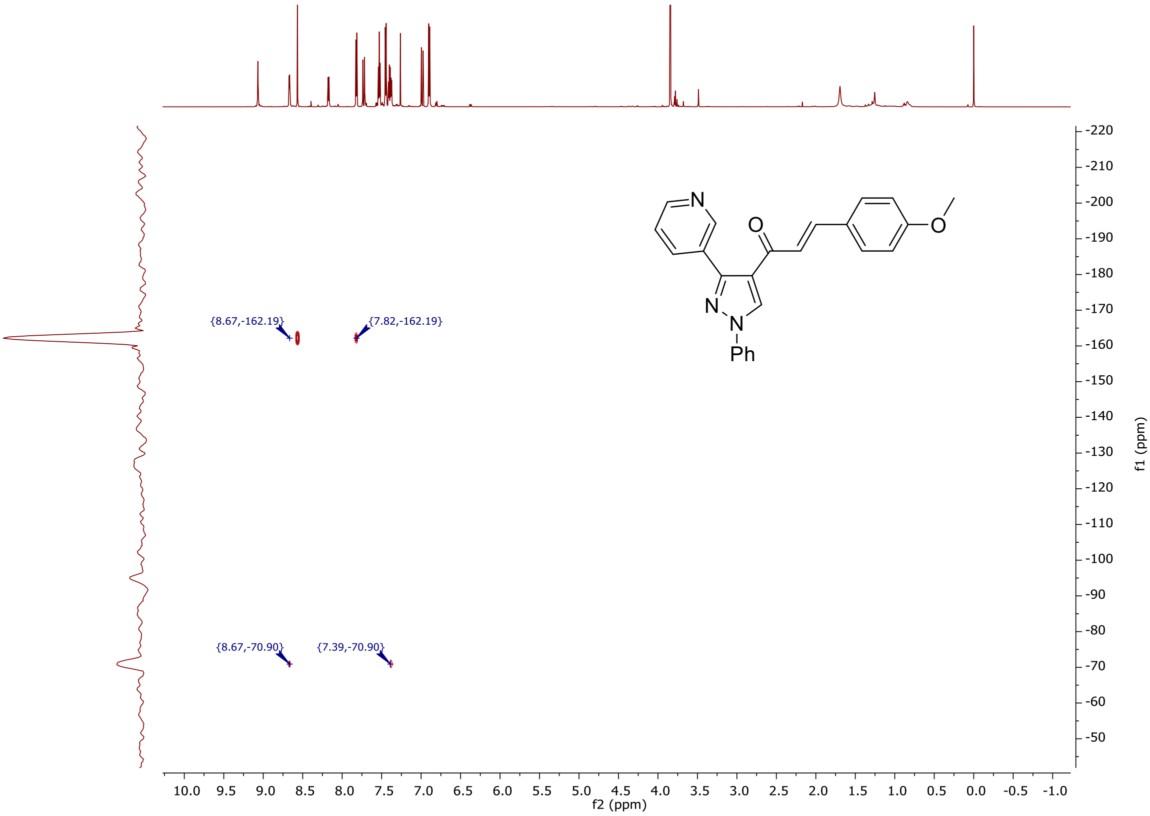

Figure S171. (2*E*)-3-(4-Methoxyphenyl)-1-[1-phenyl-3-(pyridin-3-yl)-1*H*-pyrazol-4-yl]prop-2-en-1-one (12h). ^{1}H-^{15}N HMBC NMR spectrum (71 MHz, $CDCl_3$)

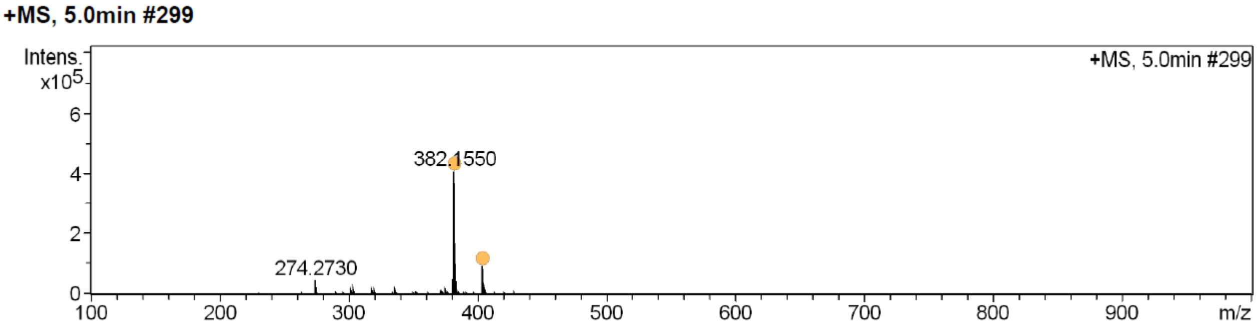

Meas. m/z	#	Ion Formula	m/z	err [ppm]	mSigma	# Sigma	Score	rdb	e^- Conf	N-Rule
382.1550	1	C24H20N3O2	382.1550	0.1	3.3	1	100.00	16.5	even	ok
404.1369	1	C24H19N3NaO2	404.1369	-0.1	5.7	1	100.00	16.5	even	ok

Figure S172. (2*E*)-3-(4-Methoxyphenyl)-1-[1-phenyl-3-(pyridin-3-yl)-1*H*-pyrazol-4-yl]prop-2-en-1-one (12h). HRMS (ESI-TOF).

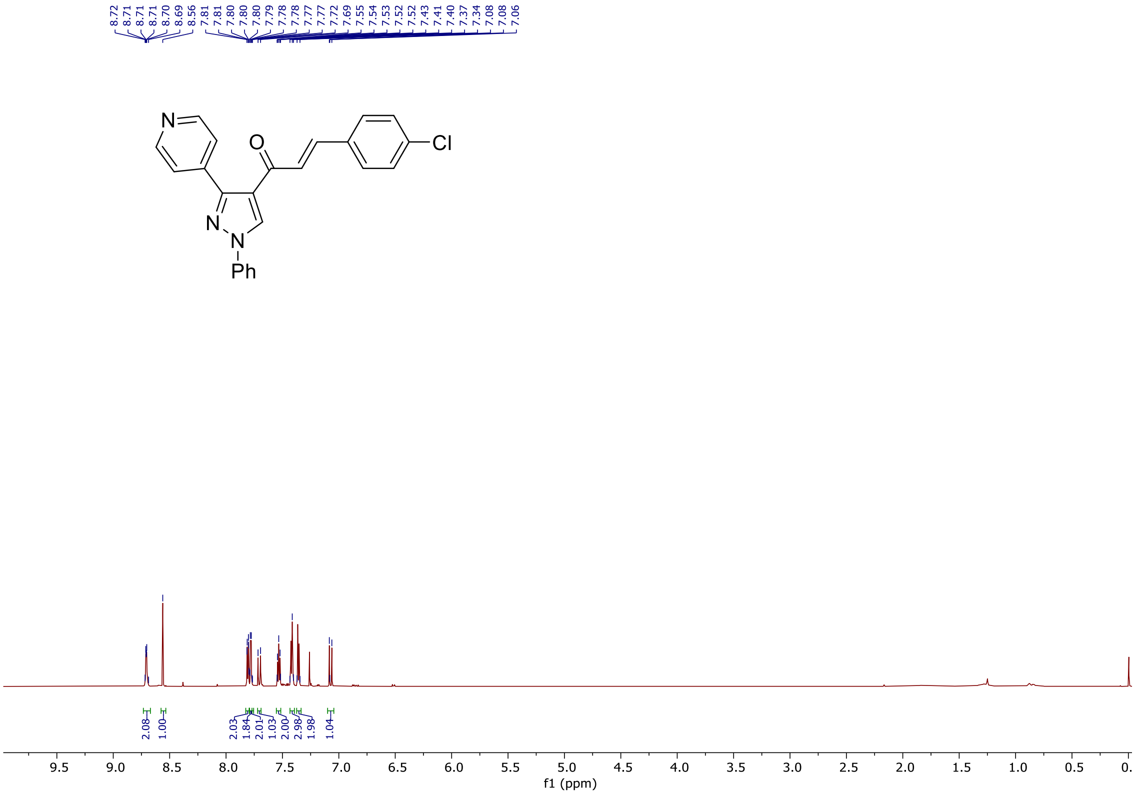

Figure S173. (2*E*)-3-(4-Chlorophenyl)-1-[1-phenyl-3-(pyridin-4-yl)-1*H*-pyrazol-4-yl]prop-2-en-1-one (12i). ^{1}H NMR spectrum (700 MHz, $CDCl_3$)

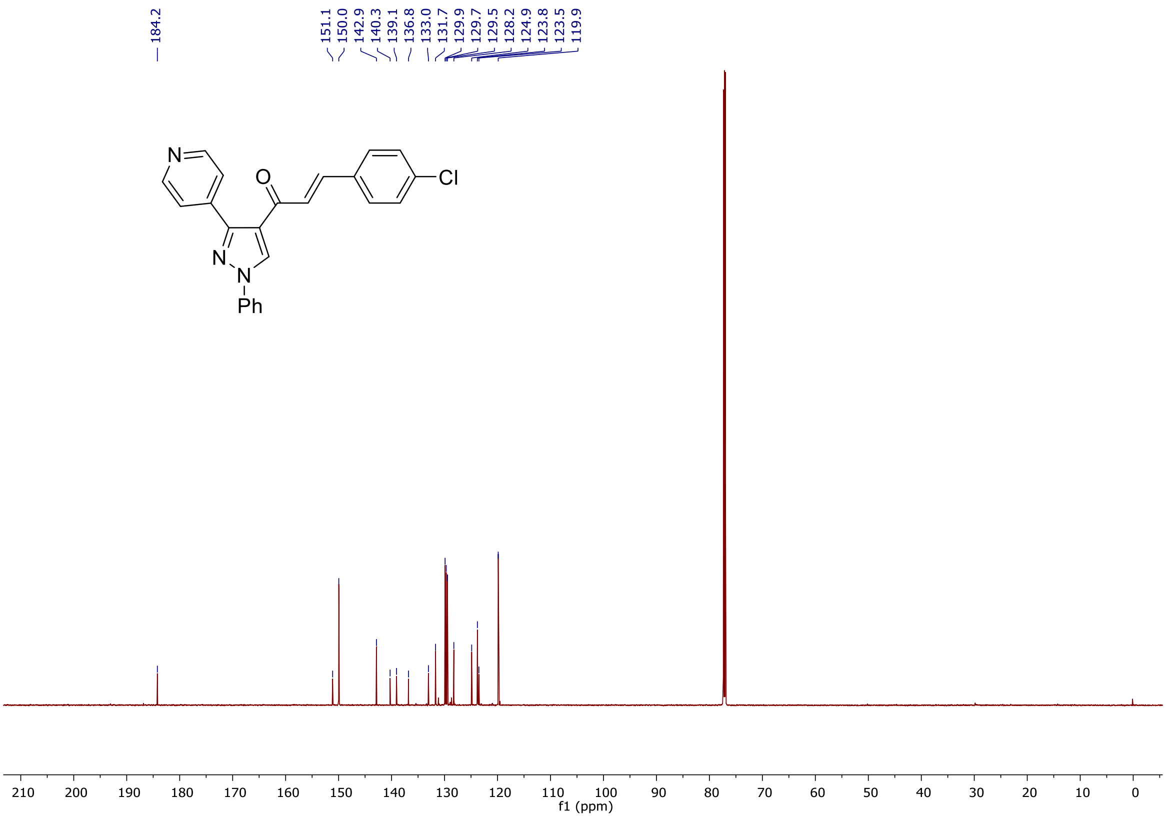

Figure S174. (2*E*)-3-(4-Chlorophenyl)-1-[1-phenyl-3-(pyridin-4-yl)-1*H*-pyrazol-4-yl]prop-2-en-1-one (12i). ^{13}C NMR spectrum (176 MHz, $CDCl_3$)

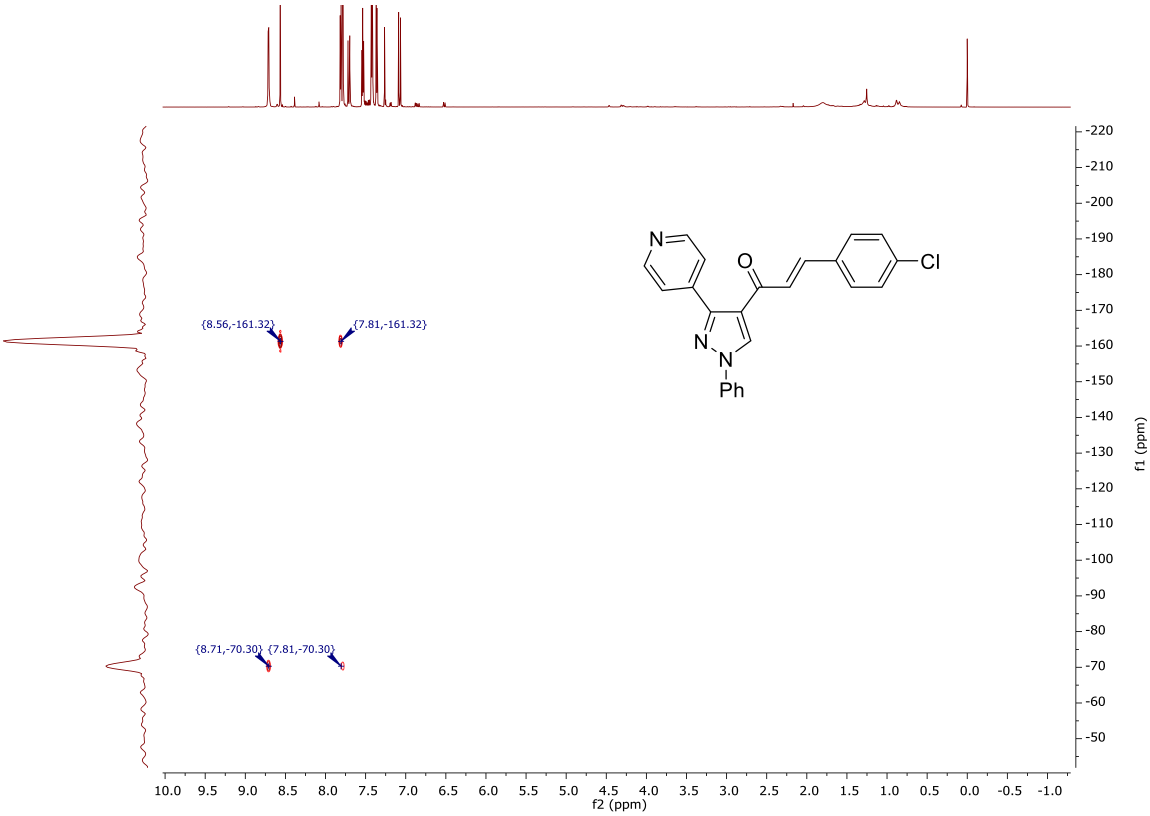

Figure S175. (2*E*)-3-(4-Chlorophenyl)-1-[1-phenyl-3-(pyridin-4-yl)-1*H*-pyrazol-4-yl]prop-2-en-1-one (12i). ^{1}H-^{15}N HMBC NMR spectrum (71 MHz, CDCl$_3$)

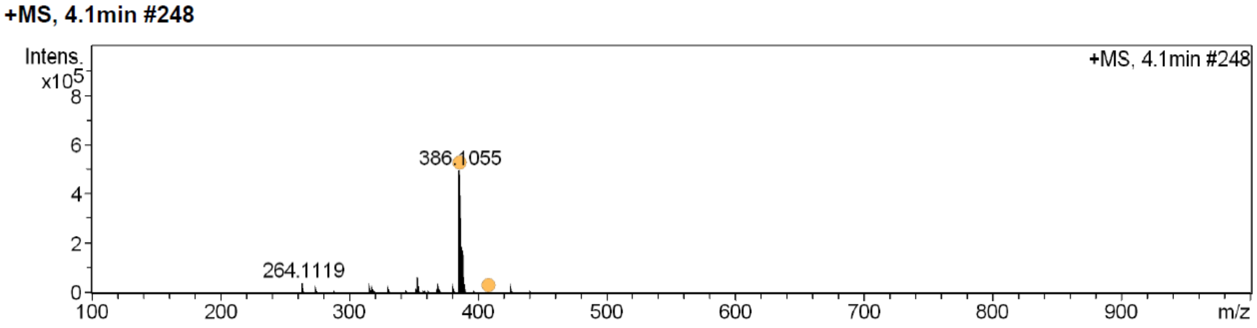

Meas. m/z	#	Ion Formula	m/z	err [ppm]	mSigma	# Sigma	Score	rdb	e$^-$ Conf	N-Rule
386.1055	1	C23H17ClN3O	386.1055	0.2	4.2	1	100.00	16.5	even	ok
408.0864	1	C23H16ClN3NaO	408.0874	2.5	182.6	1	100.00	16.5	even	ok

Figure S176. (2*E*)-3-(4-Chlorophenyl)-1-[1-phenyl-3-(pyridin-4-yl)-1*H*-pyrazol-4-yl]prop-2-en-1-one (12i). HRMS (ESI-TOF).

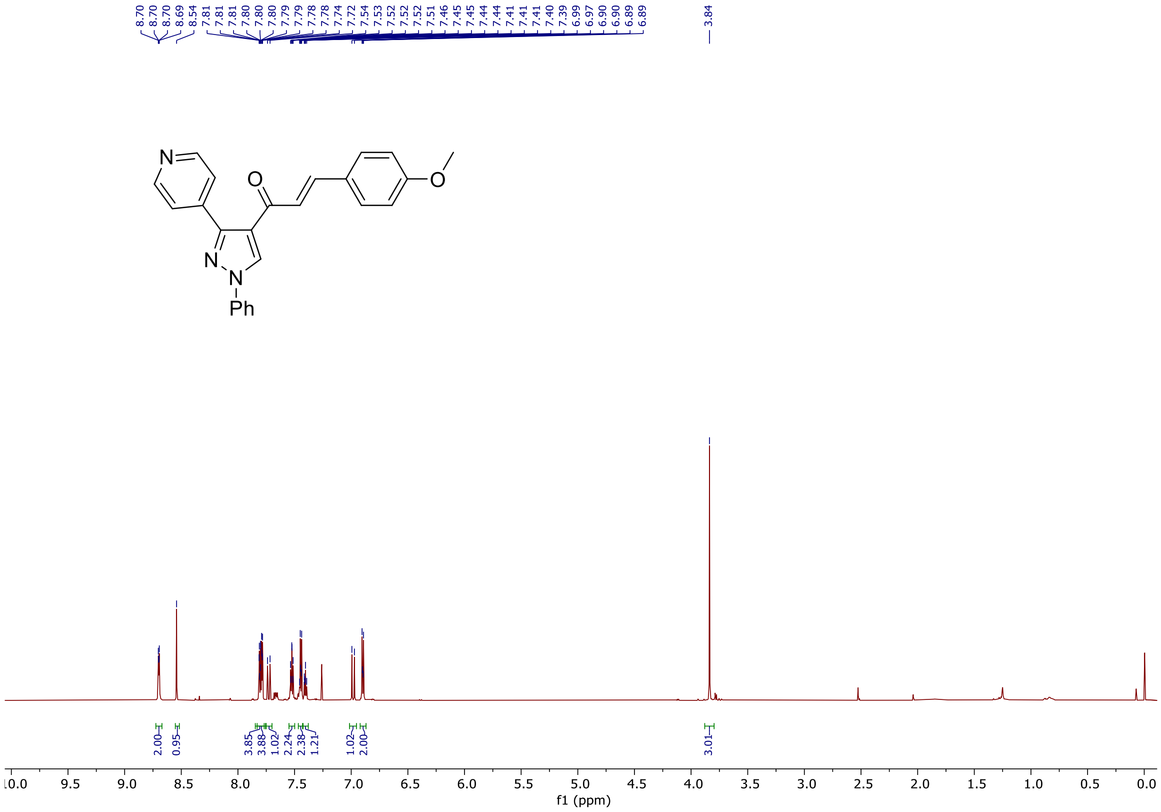

Figure S177. (2*E*)-3-(4-Methoxyphenyl)-1-[1-phenyl-3-(pyridin-4-yl)-1*H*-pyrazol-4-yl]prop-2-en-1-one (12j). ^{1}H NMR spectrum (700 MHz, $CDCl_3$)

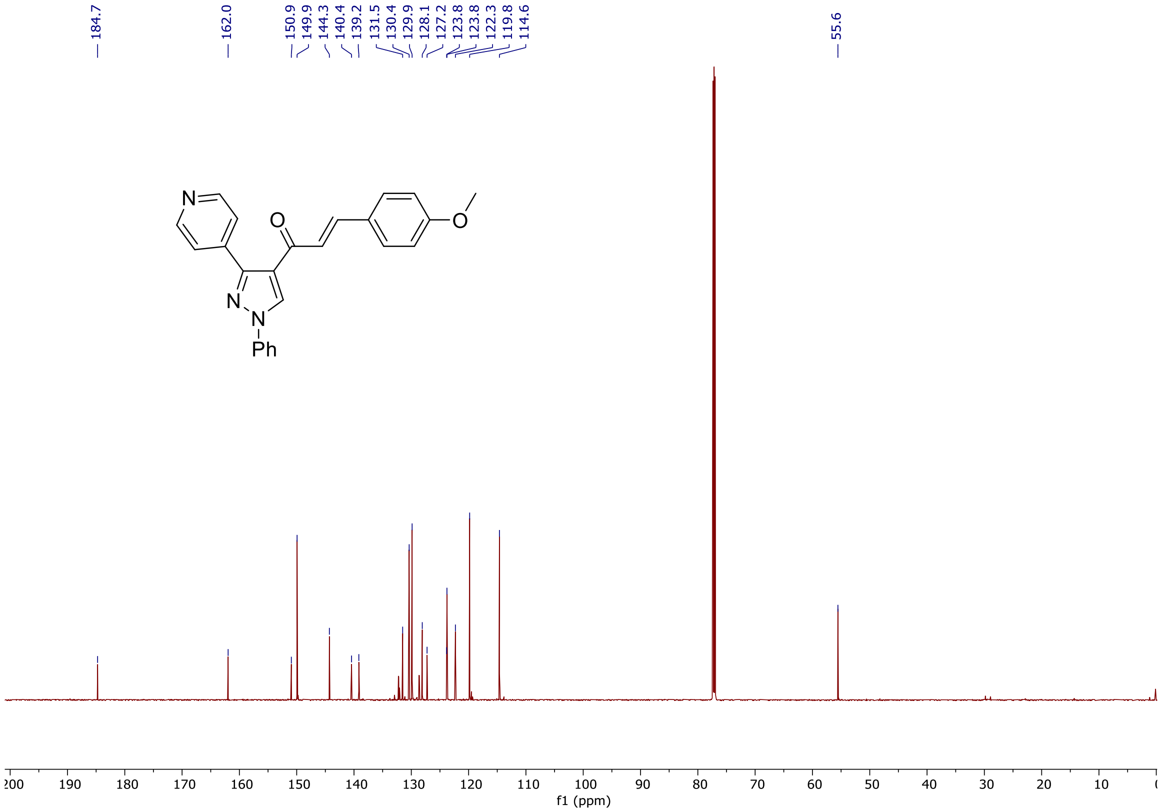

Figure S178. (2*E*)-3-(4-Methoxyphenyl)-1-[1-phenyl-3-(pyridin-4-yl)-1*H*-pyrazol-4-yl]prop-2-en-1-one (12j). ^{13}C NMR spectrum (176 MHz, $CDCl_3$)

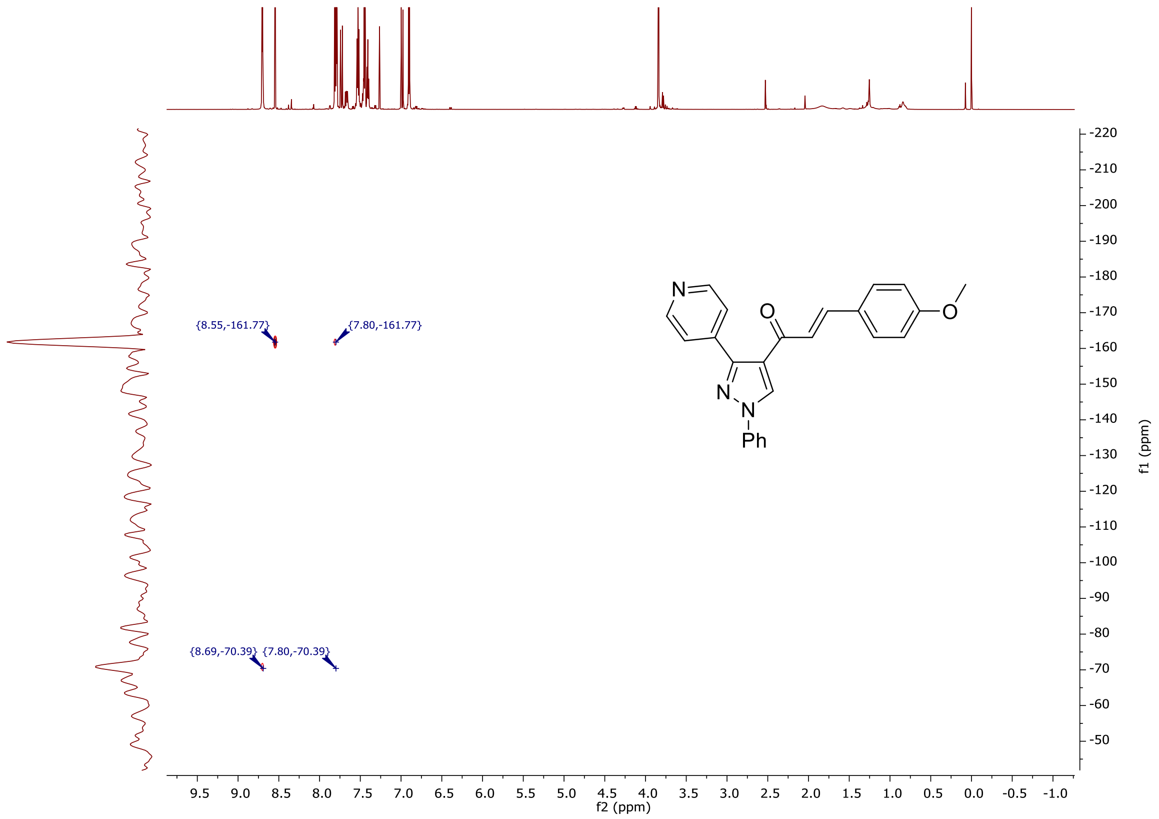

Figure S179. (2*E*)-3-(4-Methoxyphenyl)-1-[1-phenyl-3-(pyridin-4-yl)-1*H*-pyrazol-4-yl]prop-2-en-1-one (12j). ^{1}**H-**^{15}**N HMBC NMR spectrum (71 MHz, CDCl$_3$)**

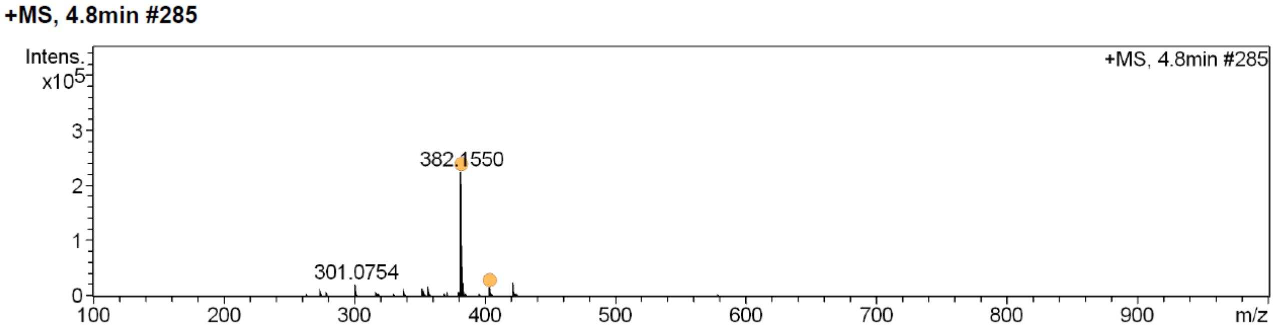

Meas. m/z	#	Ion Formula	m/z	err [ppm]	mSigma	# Sigma	Score	rdb	e¯ Conf	N-Rule
382.1550	1	C24H20N3O2	382.1550	-0.1	4.9	1	100.00	16.5	even	ok
404.1357	1	C24H19N3NaO2	404.1369	3.2	21.2	1	100.00	16.5	even	ok

Figure S180. (2*E*)-3-(4-Methoxyphenyl)-1-[1-phenyl-3-(pyridin-4-yl)-1*H*-pyrazol-4-yl]prop-2-en-1-one (12j). HRMS (ESI-TOF).

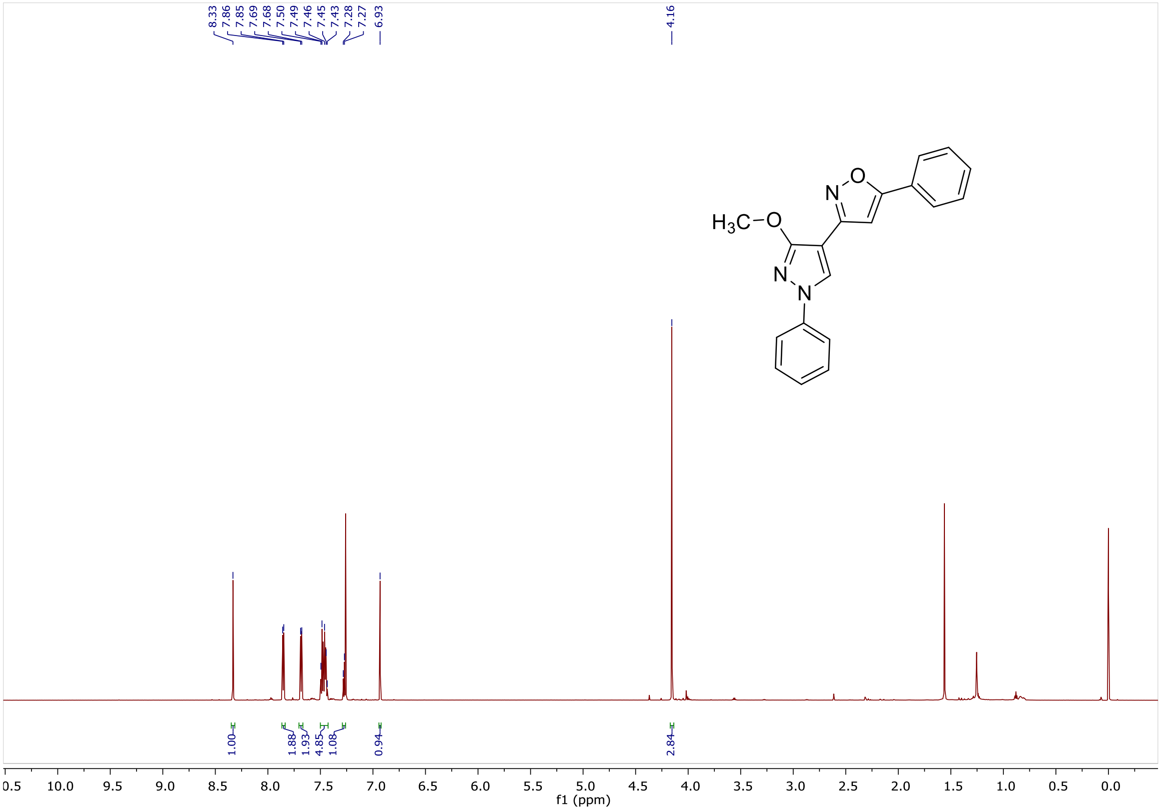

Figure S181. 3-(3-Methoxy-1-phenyl-1*H*-pyrazol-4-yl)-5-phenyl-1,2-oxazole (14). ^{1}H NMR spectrum (700 MHz, $CDCl_3$)

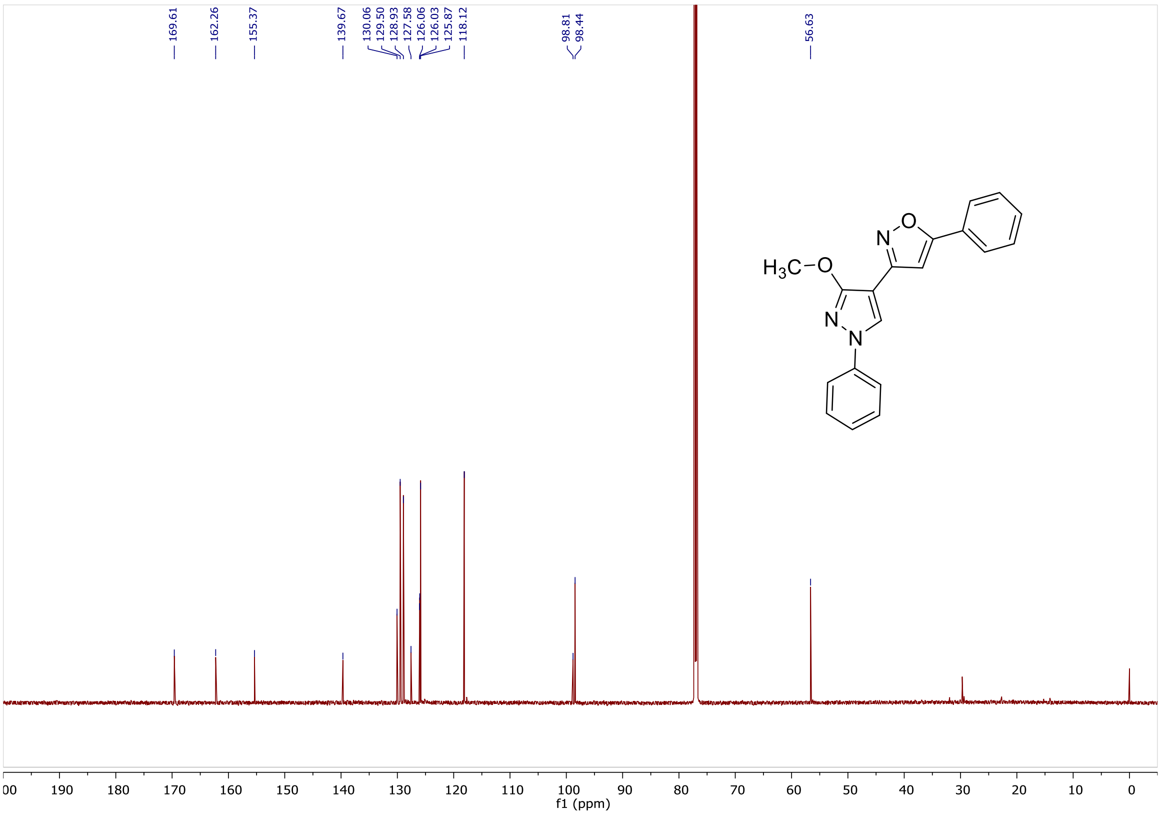

Figure S182. 3-(3-Methoxy-1-phenyl-1*H*-pyrazol-4-yl)-5-phenyl-1,2-oxazole (14). ^{13}C NMR spectrum (176 MHz, $CDCl_3$)

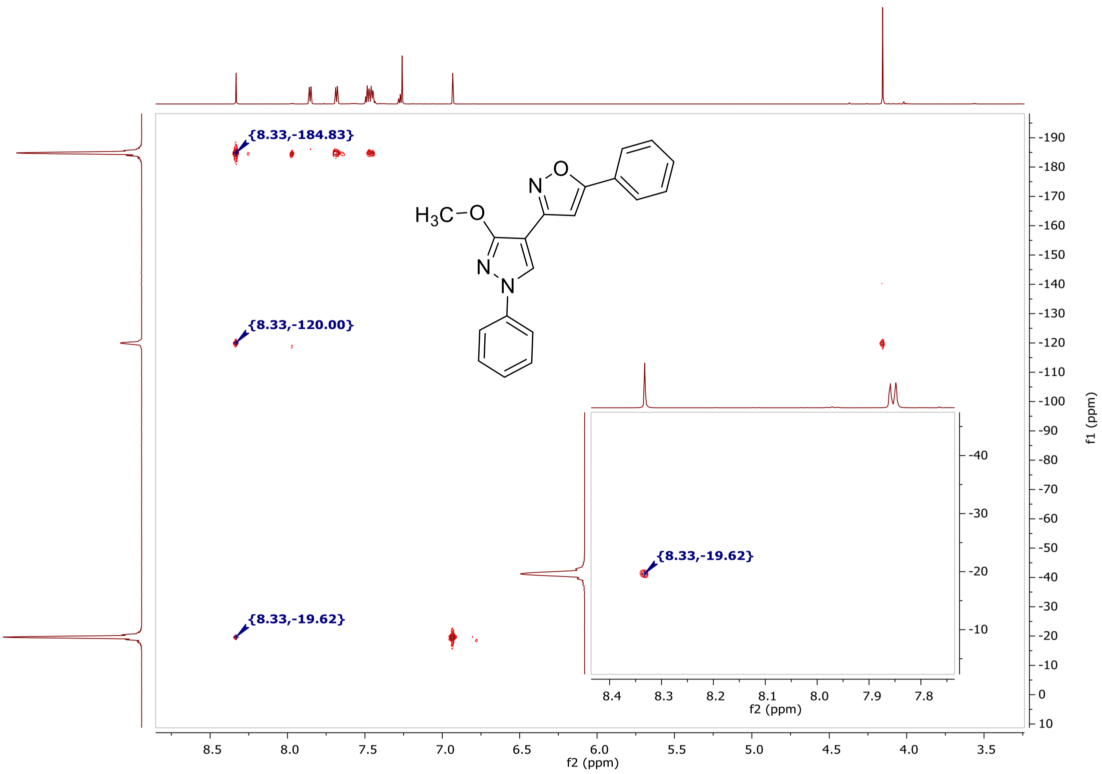

Figure S183. 3-(3-Methoxy-1-phenyl-1*H*-pyrazol-4-yl)-5-phenyl-1,2-oxazole (14). ^{1}H-^{15}N HMBC NMR spectrum (71 MHz, $CDCl_3$)

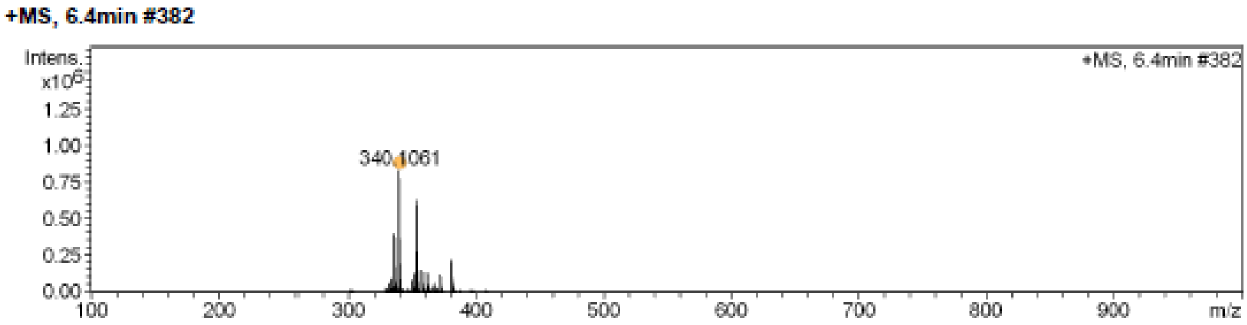

Meas. m/z	#	Ion Formula	m/z	err [ppm]	mSigma	# Sigma	Score	rdb	e⁻ Conf	N-Rule
340.1061	1	C19H15N3NaO2	340.1056	-1.3	17.1	2	100.00	13.5	even	ok

Figure S184. 3-(3-Methoxy-1-phenyl-1*H*-pyrazol-4-yl)-5-phenyl-1,2-oxazole (14). HRMS (ESI-TOF).

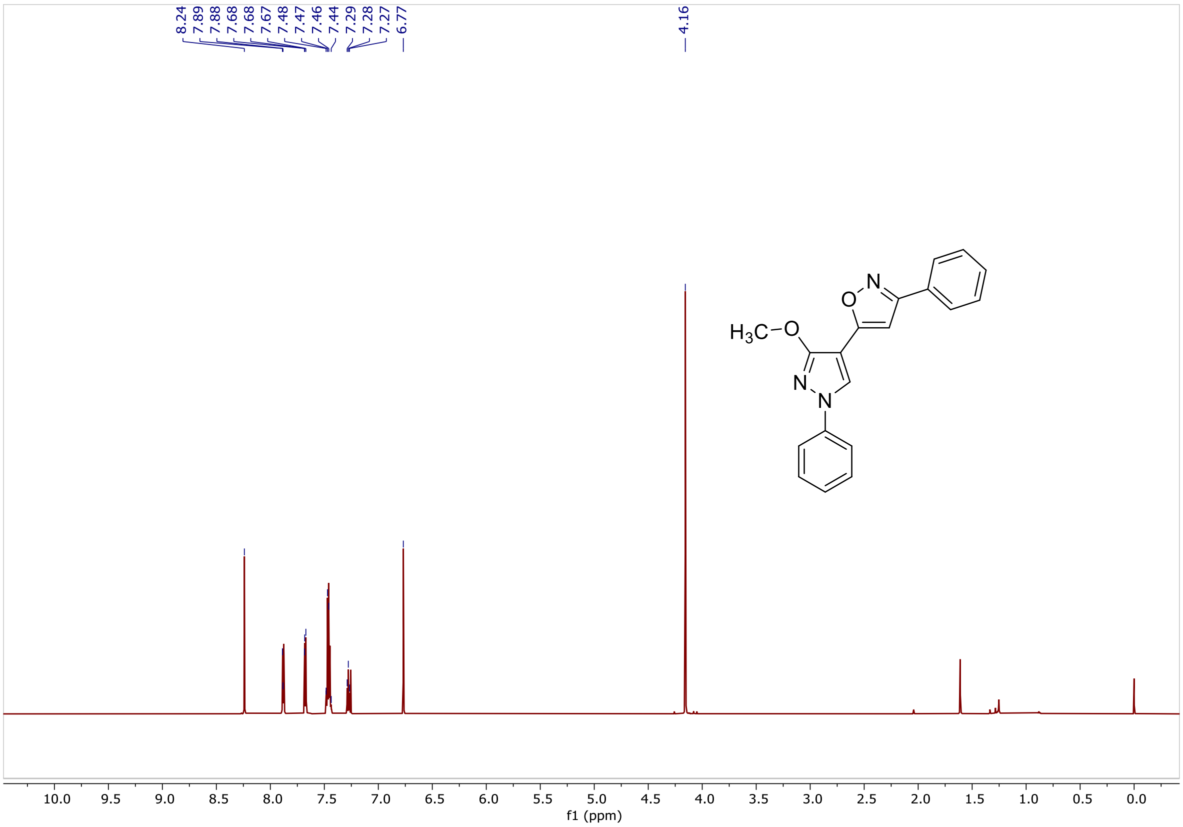

Figure S185. 5-(3-Methoxy-1-phenyl-1*H*-pyrazol-4-yl)-3-phenyl-1,2-oxazole (15). ^{1}H NMR spectrum (700 MHz, CDCl$_3$)

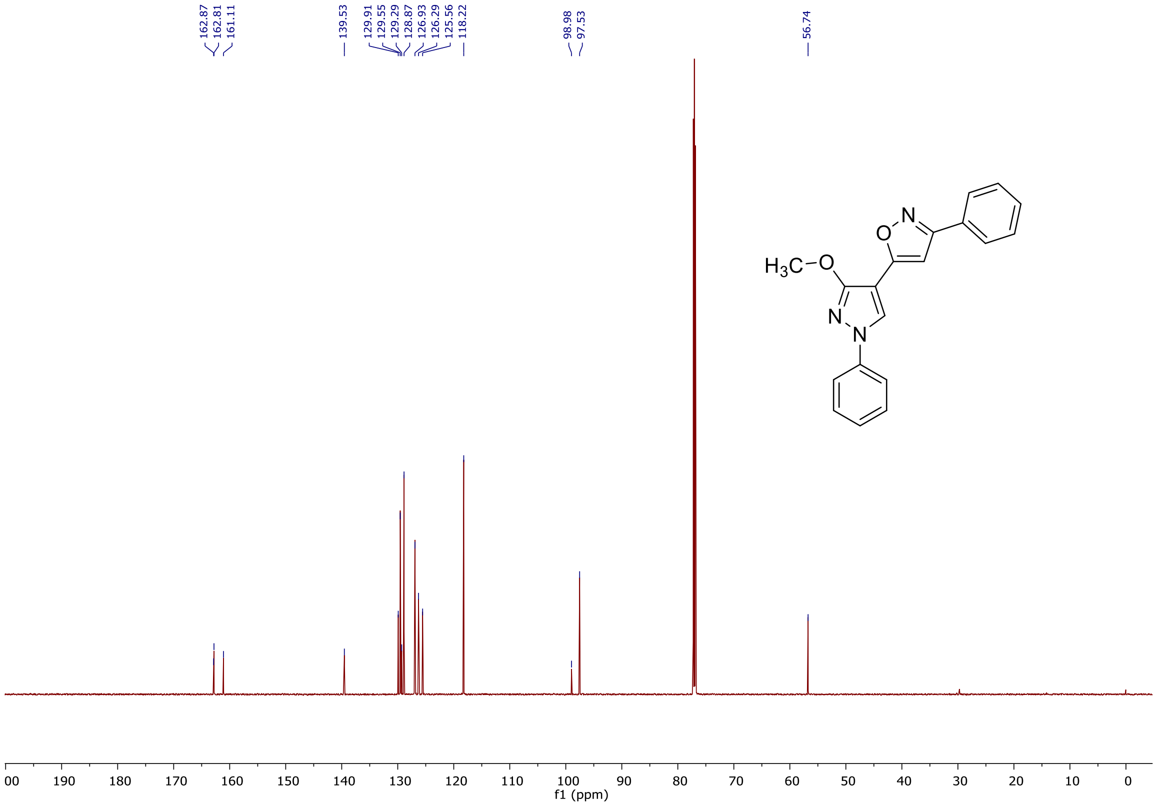

Figure S186. 5-(3-Methoxy-1-phenyl-1*H*-pyrazol-4-yl)-3-phenyl-1,2-oxazole (15). ^{13}C NMR spectrum (176 MHz, CDCl$_3$)

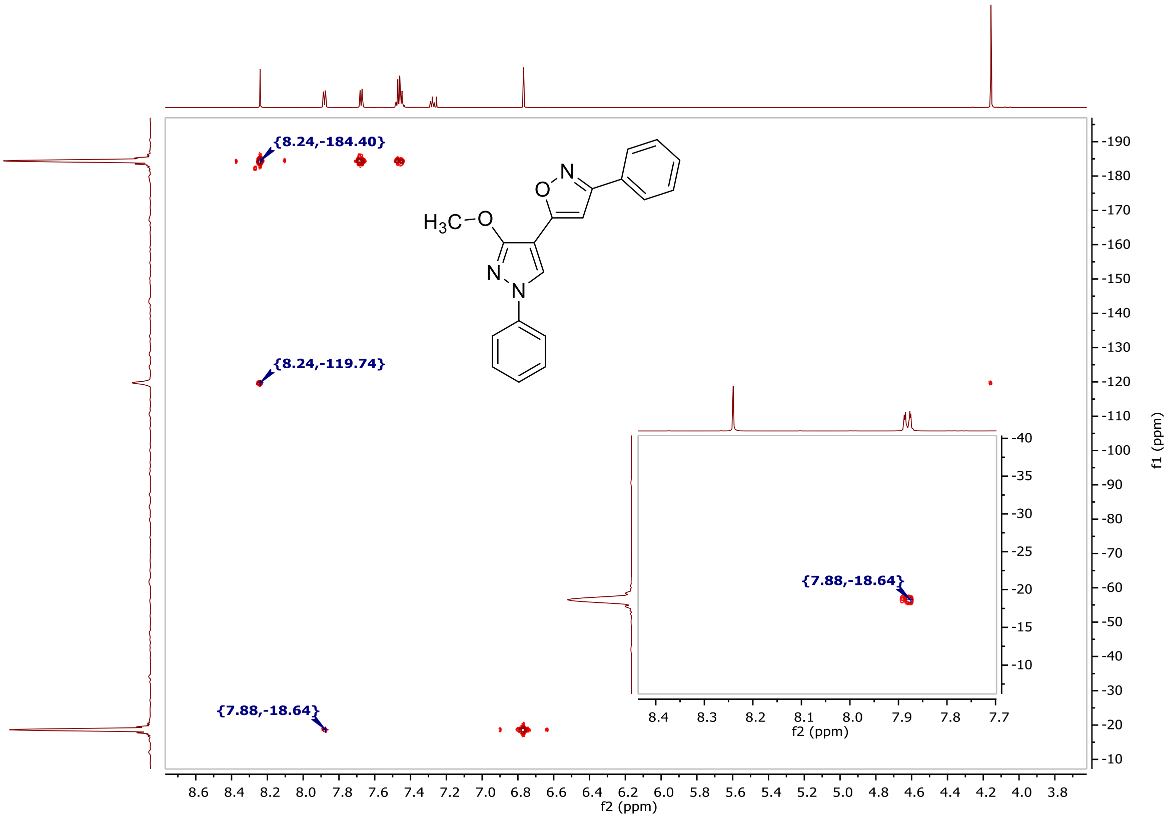

Figure S187. 5-(3-Methoxy-1-phenyl-1*H*-pyrazol-4-yl)-3-phenyl-1,2-oxazole (15). ^{1}H-^{15}N HMBC NMR spectrum (71 MHz, $CDCl_3$)

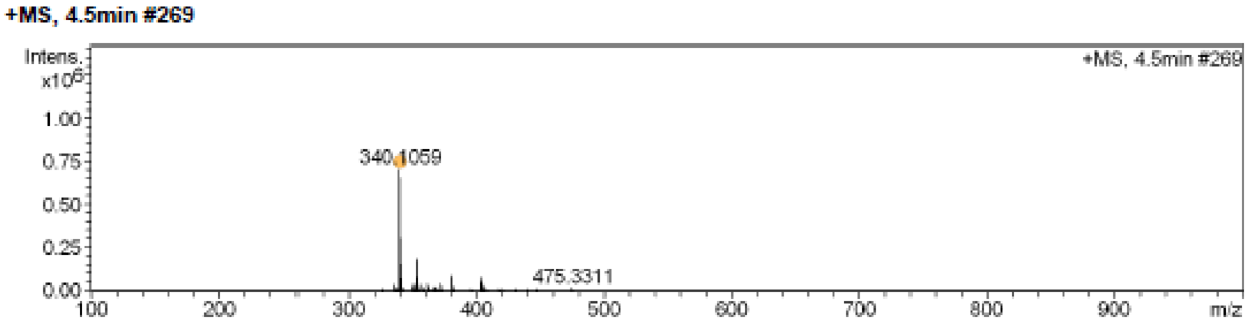

Meas. m/z	#	Ion Formula	m/z	err [ppm]	mSigma	# Sigma	Score	rdb	e⁻ Conf	N-Rule
340.1059	1	C19H15N3NaO2	340.1056	-0.9	12.0	1	100.00	13.5	even	ok

Figure S188. 5-(3-Methoxy-1-phenyl-1*H*-pyrazol-4-yl)-3-phenyl-1,2-oxazole (15). HRMS (ESI-TOF).

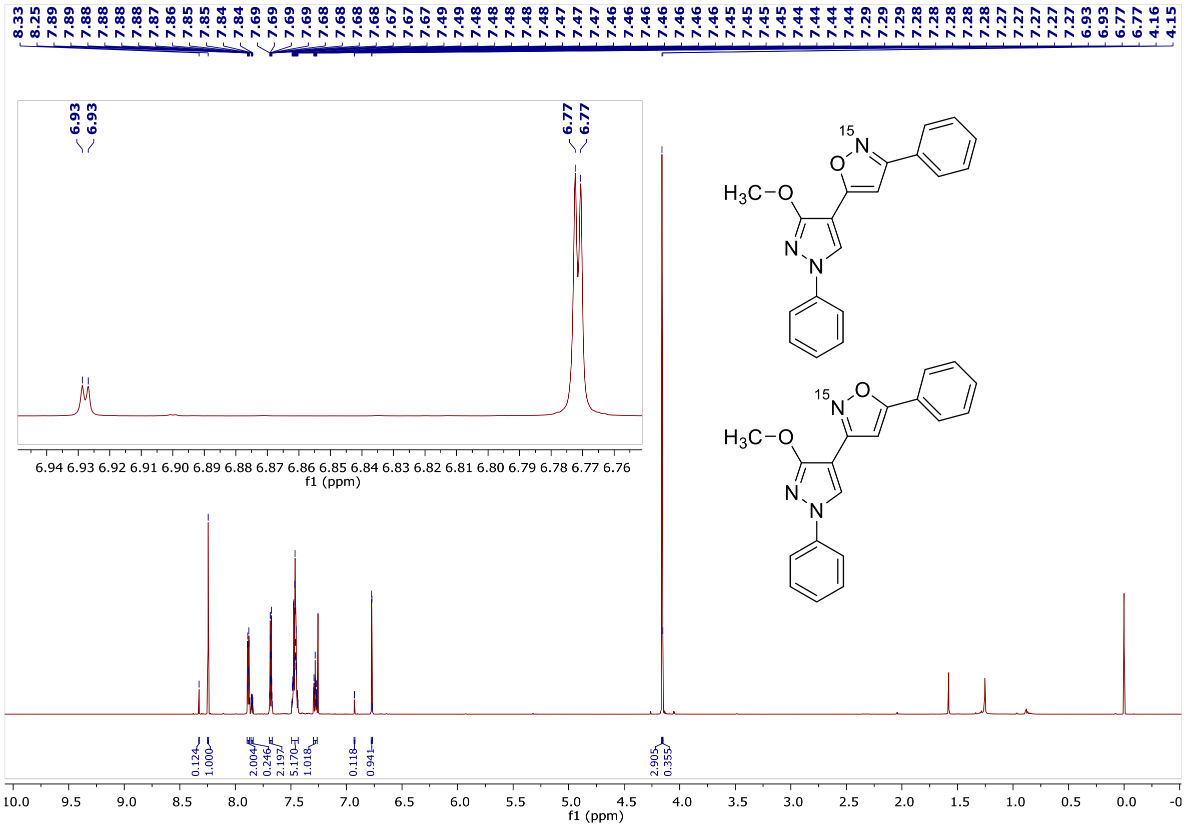

Figure S189. 3(5)-(3-Methoxy-1-phenyl-1*H*-pyrazol-4-yl)-(5)3-phenyl-^{15}N-1,2-oxazole (mixture of 16, 17). ^{1}H NMR spectrum (700 MHz, $CDCl_3$)

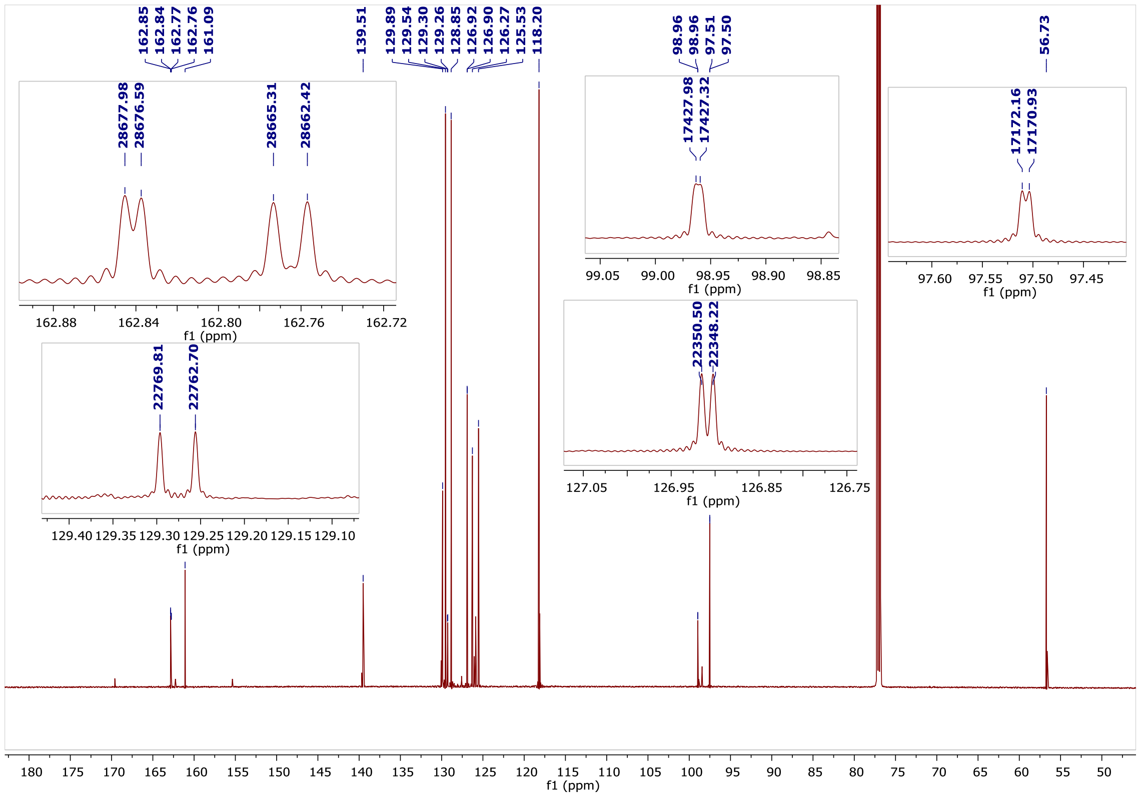

Figure S190. 3(5)-(3-Methoxy-1-phenyl-1*H*-pyrazol-4-yl)-(5)3-phenyl-^{15}N-1,2-oxazole (mixture of 16, 17), signals of major regioisomer 16. ^{13}C NMR spectrum (176 MHz, $CDCl_3$)

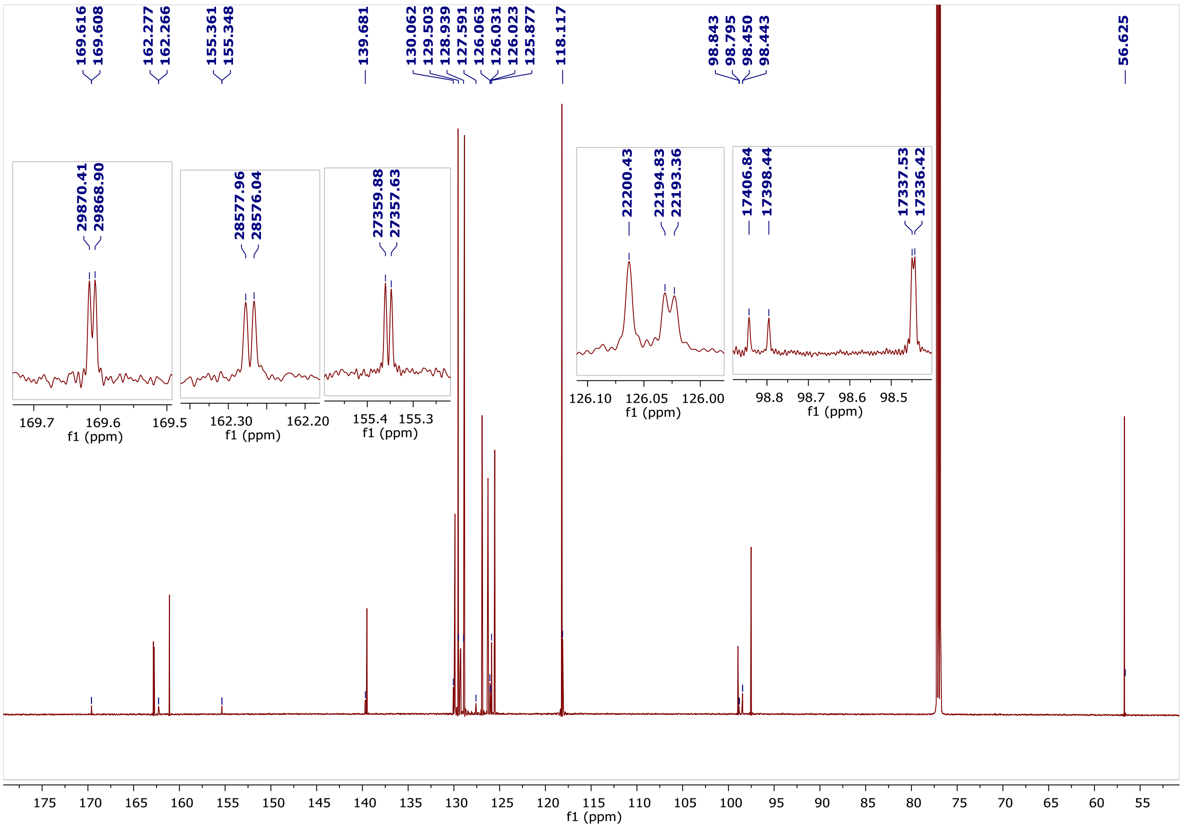

Figure S191. 3(5)-(3-Methoxy-1-phenyl-1*H*-pyrazol-4-yl)-(5)3-phenyl-^{15}N-1,2-oxazole (mixture of 16, 17), signals of minor regioisomer 17. ^{13}C NMR spectrum (176 MHz, $CDCl_3$)

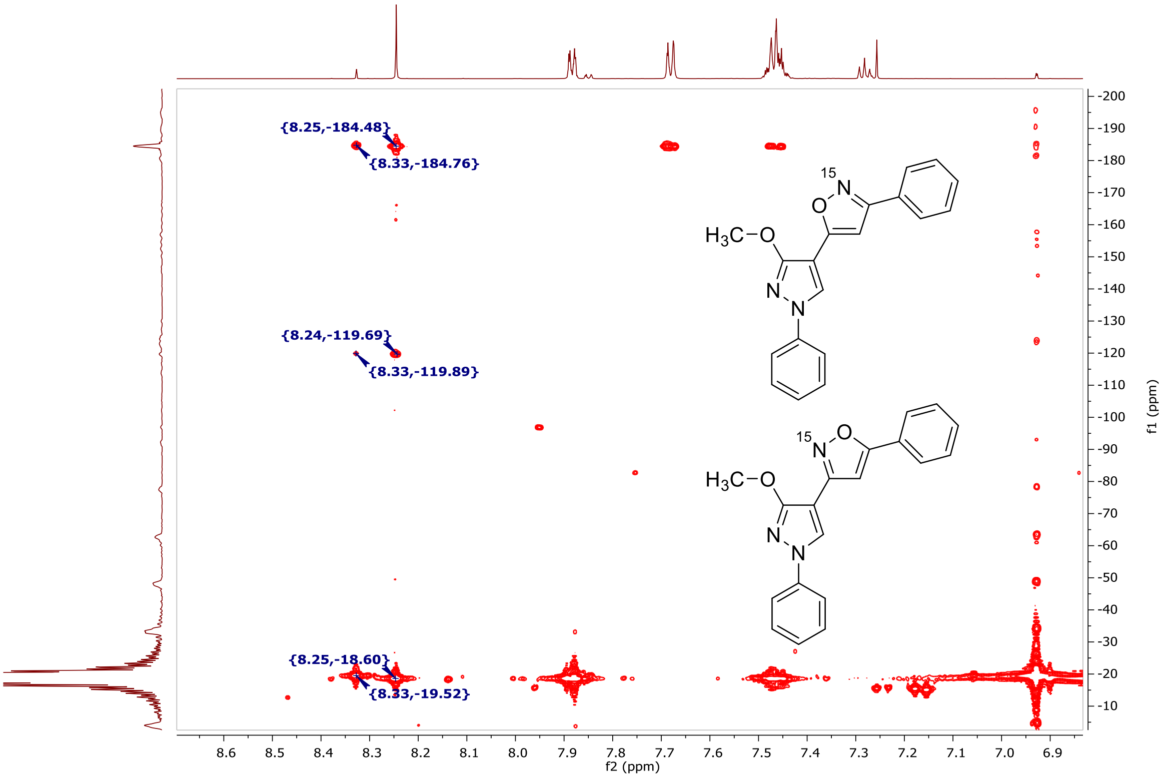

Figure S192. 3(5)-(3-Methoxy-1-phenyl-1*H*-pyrazol-4-yl)-(5)3-phenyl-^{15}N-1,2-oxazole (mixture of 16, 17). ^{1}H-^{15}N HMBC NMR spectrum (71 MHz, $CDCl_3$)

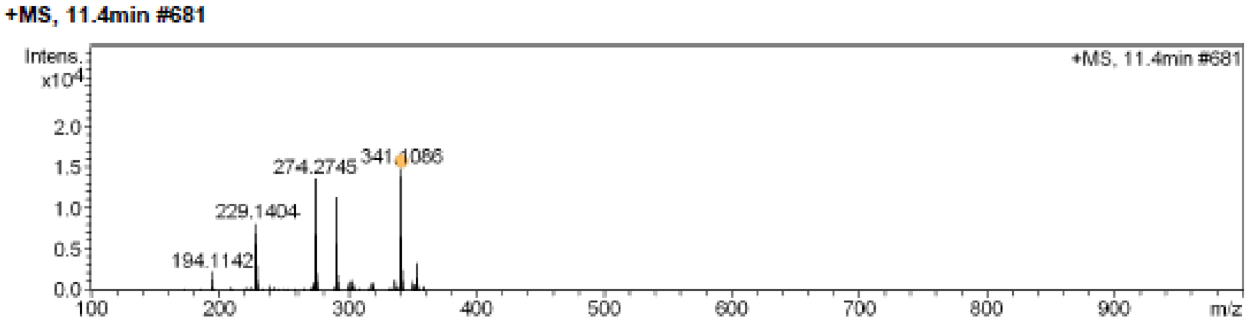

Meas. m/z	#	Ion Formula	m/z	err [ppm]	mSigma	# Sigma	Score	rdb	e⁻ Conf	N-Rule
341.1086	1	C19H15N3NaO2	340.1056	-0.2	731.9	6	0.00	13.5	even	ok

Figure S193. 3(5)-(3-Methoxy-1-phenyl-1*H*-pyrazol-4-yl)-(5)3-phenyl-^{15}N-1,2-oxazole (mixture of 16, 17). HRMS (ESI-TOF).

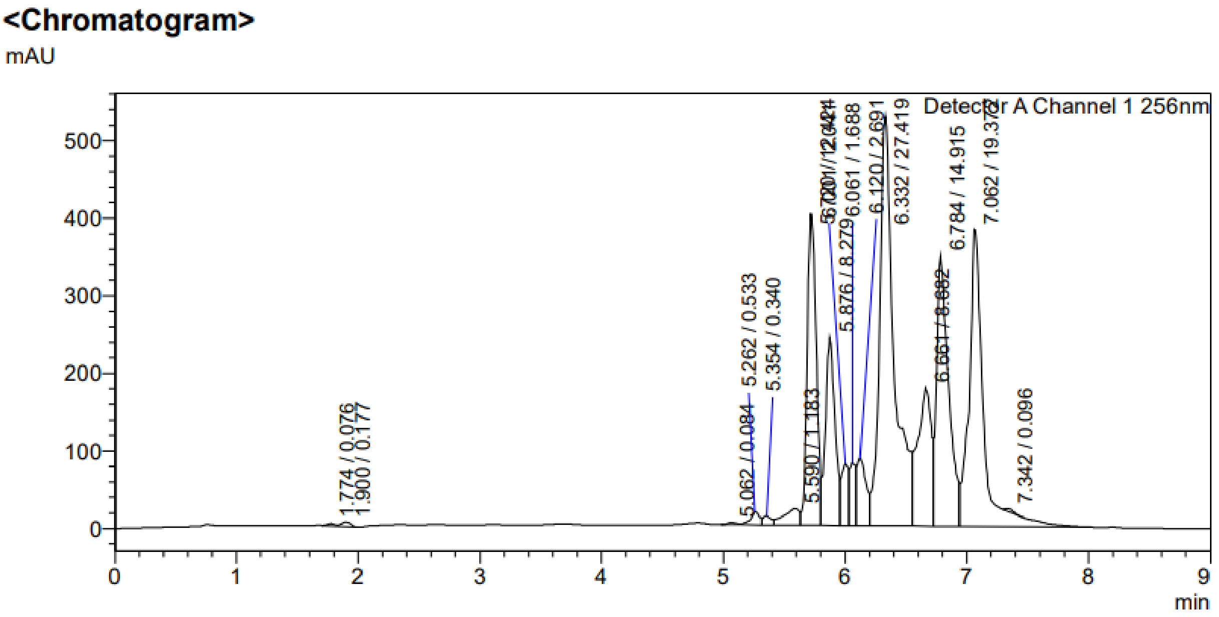

Figure S194. HPLC data of crude reaction mixture of compounds 14,15.

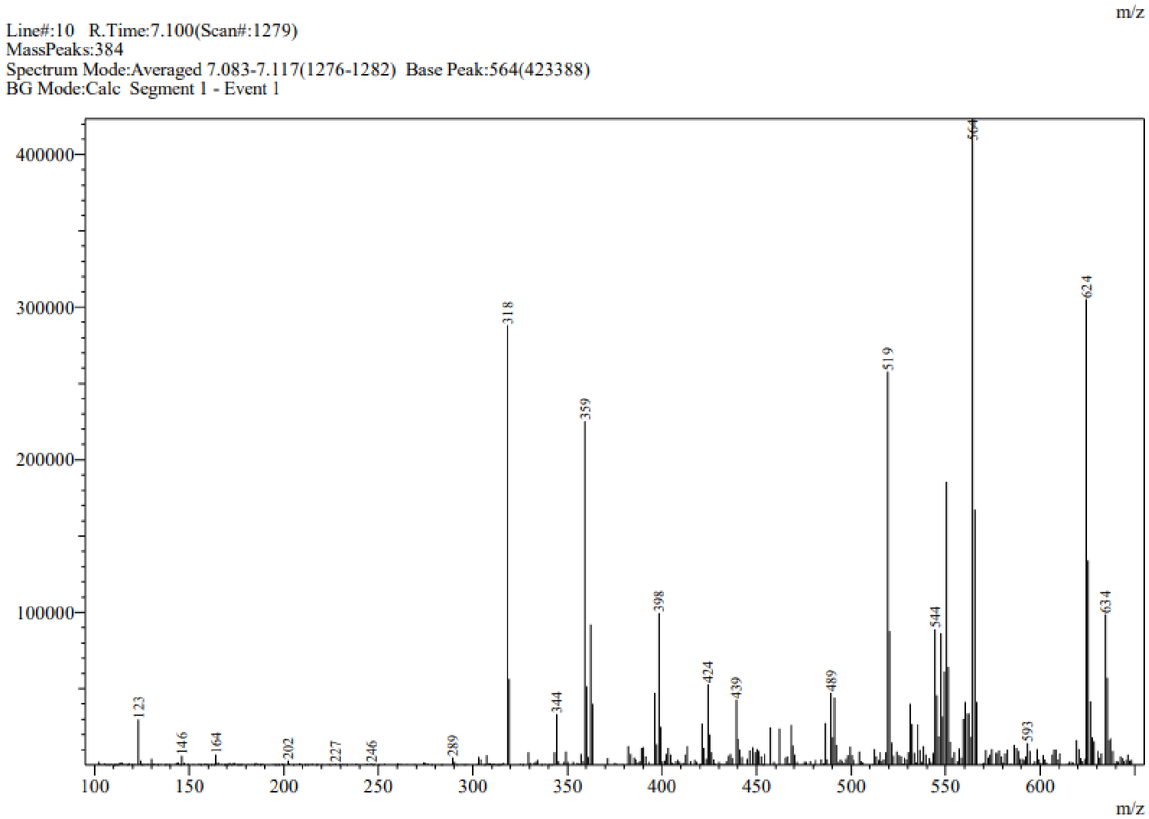

Figure S195. MS data of crude reaction mixture of compounds 14,15 (peak retention time 7.1 min.).

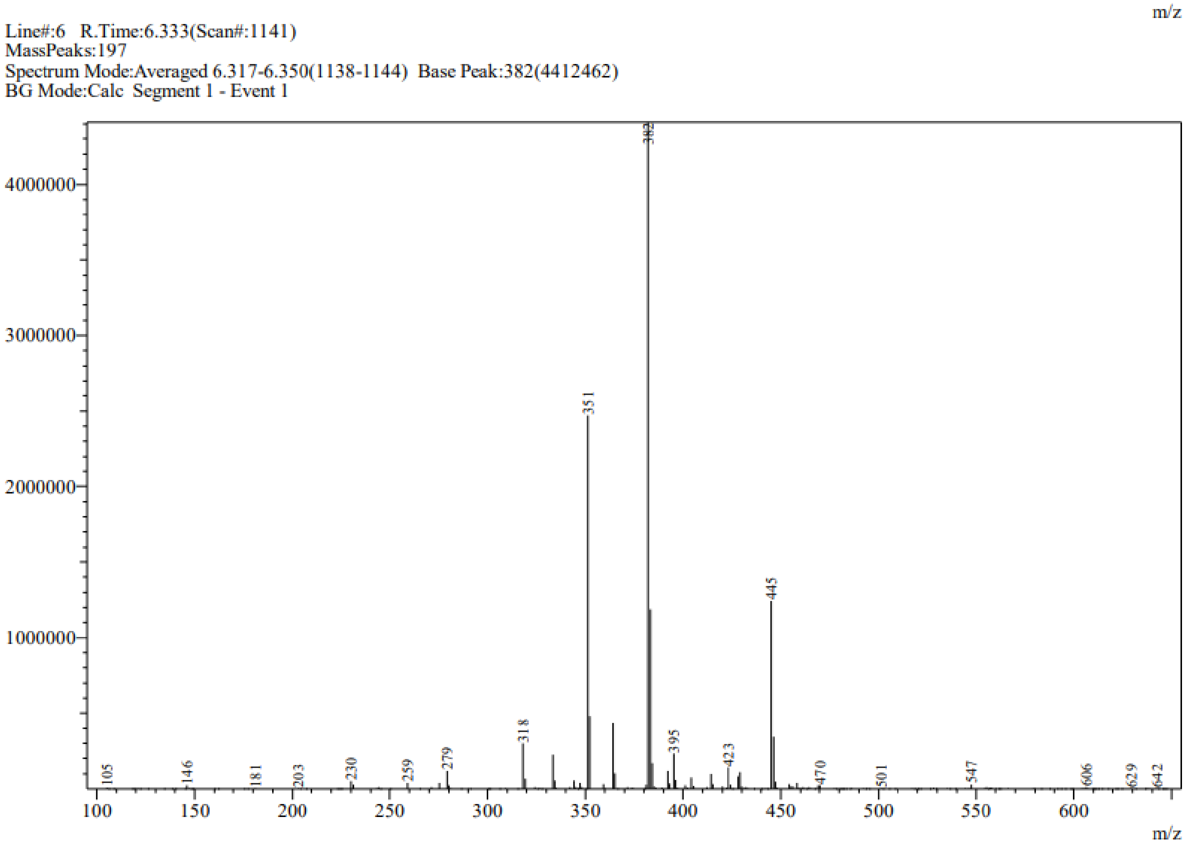

Figure S196. MS data of crude reaction mixture of compounds 14,15 (peak retention time 6.3 min.).

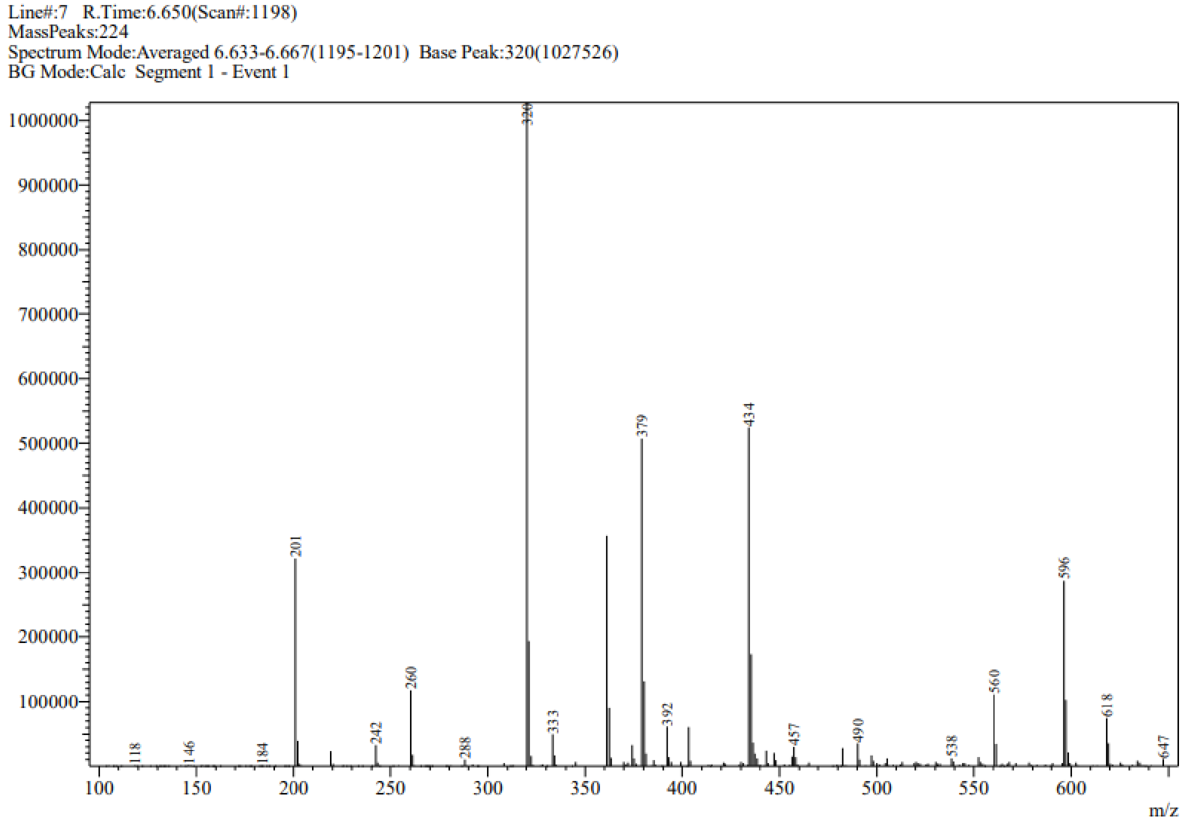

Figure S197. MS data of crude reaction mixture of compounds 14,15 (peak retention time 6.6 min.).

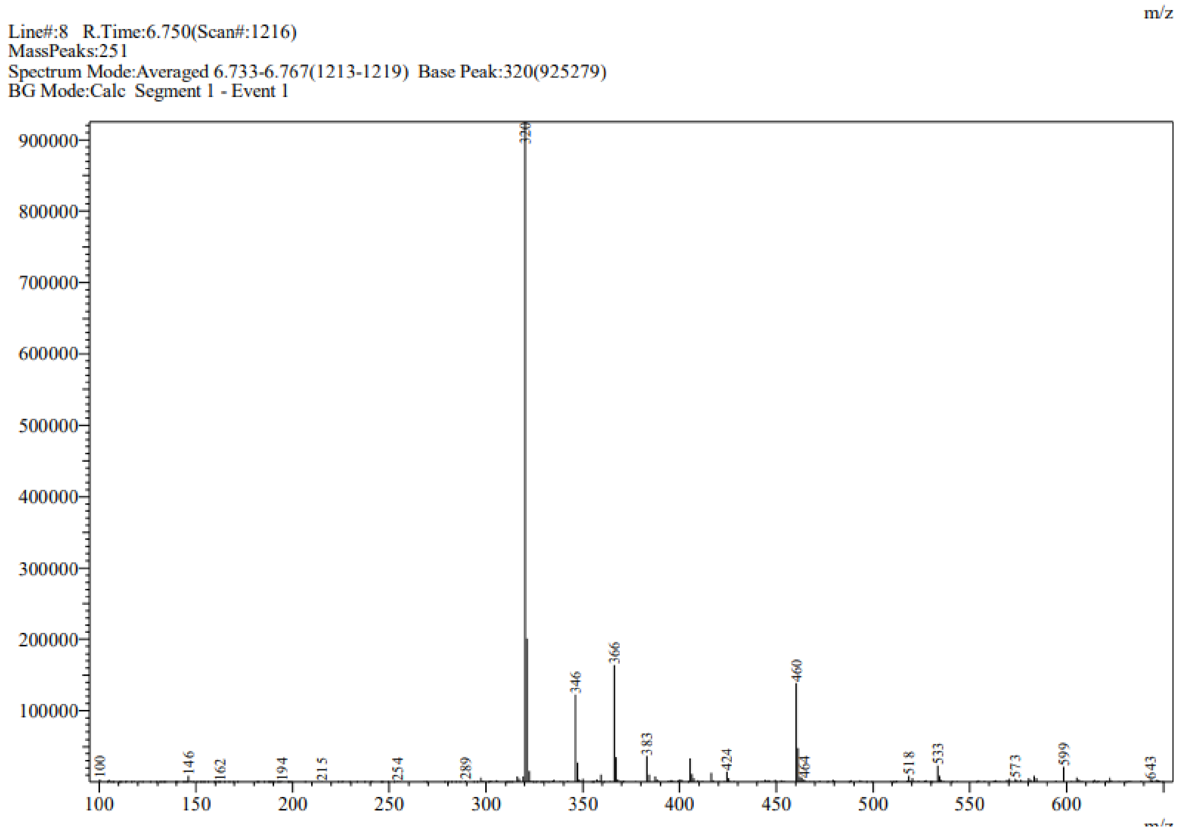

Figure S198. MS data of crude reaction mixture of compounds 14,15 (peak retention time 6.7 min.).
